# Mechanistic Analysis of Alkyne Haloboration: A DFT, MP2, and DLPNO-CCSD(T) Study

**DOI:** 10.1021/acs.jpca.3c00607

**Published:** 2023-07-25

**Authors:** Jakub Stošek, Hugo Semrád, Ctibor Mazal, Markéta Munzarová

**Affiliations:** Department of Chemistry, Faculty of Science, Masaryk University, Kotlářská 2, 611 37 Brno, Czech Republic

## Abstract

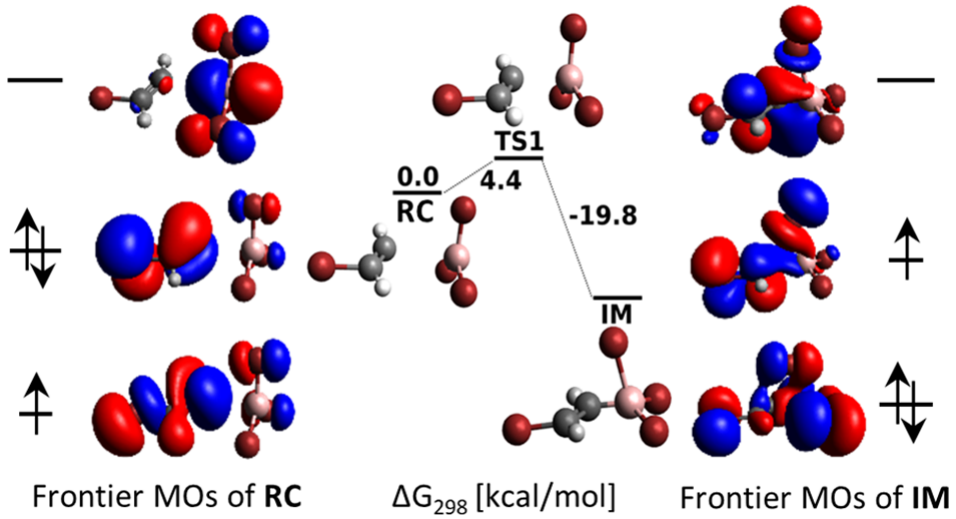

Stereocontrol of the alkyne haloboration reaction has received attention in many experimental but few theoretical studies. Here we present a detailed quantum-chemical study of mechanisms leading to *Z* versus *E* isomers of haloboration products, considering acetylene and propyne combined with BCl_3_, BBr_3_, and BI_3_. Calculations using B3LYP-D3, MP2, and DLPNO-CCSD(T) methods are used to study polar reactions between the alkyne and BX_3_ in the absence and presence of an additional halide anion whose content in the reaction mixture can be controlled experimentally. The formation of *anti*-haloboration products via radical mechanisms is also explored, namely, by adding BX_3_ to (*Z*)-halovinyl radical. For the *anti*-haloboration of propyne, the radical route is prohibited by the regiochemistry of the initiating halopropenyl radical, while the polar route is unlikely due to a competitive allene generation. In contrast, energetically accessible routes exist for both *syn*- and *anti*-bromoboration of acetylene; hence, careful control of reaction conditions is necessary to steer the stereochemical outcome. Methodologically, MP2 results correspond better to the DLPNO-CCSD(T) energies than the B3LYP-D3 results in terms of both reaction barrier heights and relative ordering of energetically close stationary points.

## 1 Introduction

Haloboration is the addition of a boron–halogen bond across an unsaturated moiety. Its importance in organic chemistry stems from the fact that it introduces two highly valuable groups in the same step: a halide and a boron unit.^1^ Extensive studies by Suzuki^2^ demonstrated the usefulness of haloboration in organic syntheses due to its regio- and stereoselectivity. Experimental studies report haloboration adducts bearing the halogen at the more substituted carbon.^3^ The first experimental report on haloboration is from 1964 by Lappert and Prokai, who studied a variety of alkynes in combination with several substituted boranes, including BBr_3_ and BCl_3_.^4^ In terms of reactivity, terminal alkynes undergo haloboration much more easily than internal alkynes, and BBr_3_ facilitates the transformation with respect to BCl_3_.

**Scheme 1:**
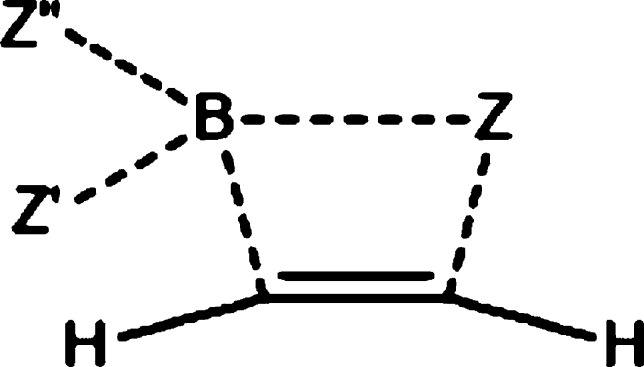
Four-Center Transition State

Regarding stereochemical behavior, higher alkyne haloboration with either BBr_3_ or BCl_3_ led to the *Z* adduct at low temperature and to a mixture of *Z* and *E* adducts at high temperature. However, in the case of acetylene, the reaction provided exclusively an *E* adduct. Lappert and Prokai suggested two possible explanations. The first assumed that the mechanism of the addition to acetylene is different and cannot involve a four-center transition state (TS) (Scheme 1) as proposed for higher alkynes. The second one suggested kinetic reaction control for higher alkynes but thermodynamic control for acetylene.

In 1973, a detailed study by Blackborow reported on the bromoboration of phenylacetylene and hex-1-yne with BBr_3_, resulting in mixtures of *Z* and *E* adducts.^5^ From the relative proportion of stereoisomers as a function of reaction conditions, the author concluded that different kinetically controlled mechanisms lead to the formation of the different stereoisomers. The *E* adduct formation was expected to be preferred in solvents more efficiently stabilizing polar transition states. However, in 1986 Wrackmeyer studied reaction of hex-3-yne with BBr_3_ and showed that the *Z*/*E* ratio could develop over several days, indicating a slow *Z* to *E* isomerization of bromoboration adducts.^6^ Similar findings were obtained for iodoboration of hex-3-yne by Siebert et al., who observed rapid formation of the *Z* adduct, which underwent slow isomerization to the *E* counterpart.^7,8^ On the other hand, in their work on chloroboration of propyne and but-1-yne at −78 °C, the same group reported *E* adduct formation without any reference to an isomerization step.^3^ Despite the rich variety of stereochemical behavior depending on reactant identity and conditions, the first mechanistic study of haloboration was done only in 2012 by Wang and Uchiyama.^9^

The latter study suggested that the reaction stereoselectivity could be understood via a mechanism opened by a *syn* addition followed by a steric *Z*/*E* conversion. Both steps were characterized by a four-center transition state involving a C–C multiple bond and a B–X single bond. The authors concluded, among others, that BBr_3_ can catalyze the isomerization of the primarily formed *Z* adduct. The free energy barrier found for such isomerization was quite high (30.0 kcal/mol) but comparable to that of *syn* addition (25.9 kcal/mol) and still lower than the barrier of a thermal process (54.6 kcal/mol). Interestingly, a mechanism that would involve successive addition and elimination of hydrogen bromide was discarded in that study on the basis of the result obtained for hydrogen chloride in analogous chloroboration.^9^

Recently, the mechanism of the acetylene bromoboration reaction has been reinvestigated in our group. Motivation for doing so stemmed from a 1994 work by one of us (C.M.) and M. Vaultier, who developed a highly stereoselective synthesis of 1-(dialkoxyboryl)-1,3-dienes based on acetylene bromoboration.^10^ The origin of the stereocontrol was explored in a joint experimental and theoretical study whose aim was to identify both polar and radical mechanisms compatible with acetylene bromoboration outcomes as a function of reaction conditions.^11,12^

In the first step of the isomerization mechanism introduced in our previous work, a bromine radical attacks the double bond of the *Z* adduct at the “boron end”. The bromine radical can come from homolysis of HBr that seems to be an inevitable impurity in BBr_3_ or from reaction of BBr_3_ with oxygen, traces of which are capable of promoting radical chain pathways in reactions of many boron derivatives.^13−15^ The addition of bromine radical is followed by rotation about the C–C single bond by ca. 180°, and the last step of the isomerization is the release of Br radical from the carbon at the “boron end” to form the *E* adduct.^16^ In addition, our results indicated that apart from isomerization of the *Z* adduct, two alternative routes to the *E* adduct exist, namely, radical and polar direct *anti* addition pathways. In the first step of the radical reaction mechanism, Br radical reacts with acetylene to get a bromovinyl radical that attacks the BBr_3_ molecule, affording a radical intermediate. It affords the *E* adduct after release of a bromine radical from boron. The first step of the polar reaction mechanism consists in BBr_3_ addition to the acetylene multiple bond to give a zwitterion, whose positively charged carbon is in the second step attacked by Br^–^ anion from experimentally inherently present HBr, affording an intermediate that gives the *E* adduct after release of Br^–^ anion from boron.^12^

In our previous work, other halogens and propyne as a model higher terminal alkyne were not studied, and in the work of Wang and Uchiyama, they were studied only to a limited extent, i.e., in the context of isomerization using the second BX_3_ molecule and a four-center TS. In the current work, we explore polar and radical mechanisms leading to *Z* and/or *E* adducts in extended scopes. In terms of reactants, interactions of acetylene and propyne with BCl_3_, BBr_3_, and BI_3_ are compared. The goal of this work is a comparison of thermodynamic (Δ_r_*G*) and kinetic (Δ*G*^⧧^) characteristics of addition reactions for acetylene and propyne in combination with different haloboranes. This will give more general insight into the relative reactivity and the stereochemical behavior.

The paper is organized as follows: we first discuss polar additions to acetylene, second polar additions to propyne, and finally the respective radical mechanisms. Discussions concerning polar additions to both acetylene and propyne are further divided into parts concerning the *syn* additions and the halide-anion-catalyzed *anti* additions. Throughout this work, DLPNO-CCSD(T)/cc-pV5Z-PP//MP2/6-31+G*_SVP energy profiles are presented in the main text. MP2 and B3LYP-D3 Gibbs free energy profiles with the 6-31+G*_SVP and def2TZVPP bases are given in the Supporting Information. Benchmarking of methods and basis sets is discussed in section 2.2.

## 2 Computational Details and Method Performance

### 2.1 Computational Details

The starting structures of BX_3_ (X = Cl, Br, I), acetylene, propyne, and van der Waals complexes were built in the program Avogadro.^17^ For these structures, geometry optimization was performed using the methods specified below. The guesses of all transition states were estimated using the single coordinate driving (SCD) method. These guesses were then optimized, followed by the frequency analysis. To verify the optimized transition states, intrinsic reaction coordinate (IRC) calculations followed by optimizations of local minima were done.

Structures were optimized at the MP2^18−23^ level of theory as implemented in Gaussian 09, revision D.01,^24^ except for Figures 10c, 12c,f, 15b,c, and the reactant complex in (Figure 13c), where Gaussian 16, revision C.01^25^ was employed. Single-point calculations of electronic energies on optimized structures were performed by means of the DLPNO-CCSD(T) method^26−29^ using ORCA, version 5.0.3.^30^ Benchmark single-point calculations were carried out at the B3LYP^31−33^ and MP2 levels of theory as implemented in Gaussian 09, revision D.01, except for Figures 10c, 12c,f, 15b, and the reactant complex in (Figure 13c), where Gaussian 16, revision C.01^25^ was employed. These are presented in the Supporting Information.

The GD3BJ dispersion correction of Grimme^34^ was employed in all DFT calculations, below denoted as B3LYP-D3. For Br and I, SVP basis sets of Ahlrichs^35,36^ were used in combination with the 6-31+G* basis set of Pople^37−44^ for H, B, C, and Cl (further labeled as 6-31+G*_SVP basis set). Additionally, MP2 calculations were performed with the Def2TZVPP extended basis set,^45,46^ while B3LYP-D3 all-electron results for iodine compounds were compared with the small-core ECP46MWB pseudopotential approach^47^ with the corresponding recommended orbital basis set of DZP quality.

The DLPNO-CCSD(T) single-point calculations were performed with the cc-pV5Z orbital basis and cc-pV5Z/C auxiliary basis of Dunning et al.,^48−50^ with the exception of iodine atoms in iodo derivatives. For the latter, the cc-pV5Z-PP orbital basis and cc-pVQZ-PP/C auxiliary basis of Dunning et al. along with the SK-MCDHF-RSC pseudopotential^51^ were emloyed. This basis set combination was used in order to overcome SCF convergence problems.

Implicit SCRF modeling of solvation using CH_2_Cl_2_ was employed in all calculations with the “pcm” (Gaussian) or “cpcm” option (ORCA).

### 2.2 Method Performance

As suggested by a reviewer, our original approach (MP2 level for structures and energies) was replaced by a combined methodology: single-point DLPNO-CCSD(T)/cc-pV5Z electronic energy calculations were performed using stationary-point geometries, zero-point energy (ZPE), and thermal corrections from the MP2/6-31G*_SVP method. The DLPNO-CCSD(T) method is known to provide highly accurate reaction barriers, with its applicability being independent of the nature of the reaction.^52^ Using the relatively big cc-pV5Z basis set renders the BSSE error negligible, and essentially “complete basis set” energies can be achieved. The method is, however, too demanding for wide-scope potential energy surface (PES) explorations. Instead, MP2 or B3LYP-D3 approaches are conventionally used to provide lower-level structures for higher-level energy evaluations.

Key decisions thus regarded the MP2 or B3LYP-D3 method choice for local minima and transition state structure calculations. Direct comparison of MP2 or B3LYP-D3 versus CCSD(T) structures was not possible since analytic gradients, necessary for efficient geometry optimizations, are not available for the DLPNO-CCSD(T) method in ORCA. We thus adopted an indirect approach comparing MP2 or B3LYP-D3 versus CCSD(T) total activation barriers and relative energies of intermediates. This is done in Figures S1–S6. In all of the latter, MP2 energy profiles—compared to B3LYP profiles—follow better the CCSD(T) energy differences between the reactants and the transition states. Figure S5 reveals the highest methodological (MP2 or B3LYP-D3) sensitivity of the reaction barriers. MP2 results, in the sense of highest TS energy with respect to reactants, are overestimated by at most 3 kcal/mol, while B3LYP-D3 results are underestimated by up to 7 kcal/mol with respect to the DLPNO-CCSD(T) reference. Furthermore, MP2 predicts in agreement with CCSD(T) a single-step profile for the chloro derivative, while B3LYP-D3 predicts the existence of an intermediate. Based on these results, we consider the MP2 method superior to the B3LYP-D3 one and employ it throughout this work for structure, ZPE, and thermal correction calculations.

Regarding basis set choice for MP2 structure determination, the combined 6-31+G*_SVP basis motivated by the study of Wang and Uchyiama^9^ is compared with the larger Def2TZVPP basis in Figures S1 and S3. The smaller 6-31+G*_SVP basis overestimates the MP2 reaction barriers by at most 3 kcal/mol (in Figures S1a) and is thus considered a suitable compromise between price and accuracy for geometry determination.

## 3 Results and Discussion

### 3.1 Polar Additions to Acetylene

#### 3.1.1 *syn*-Haloboration

**Figure 1:**
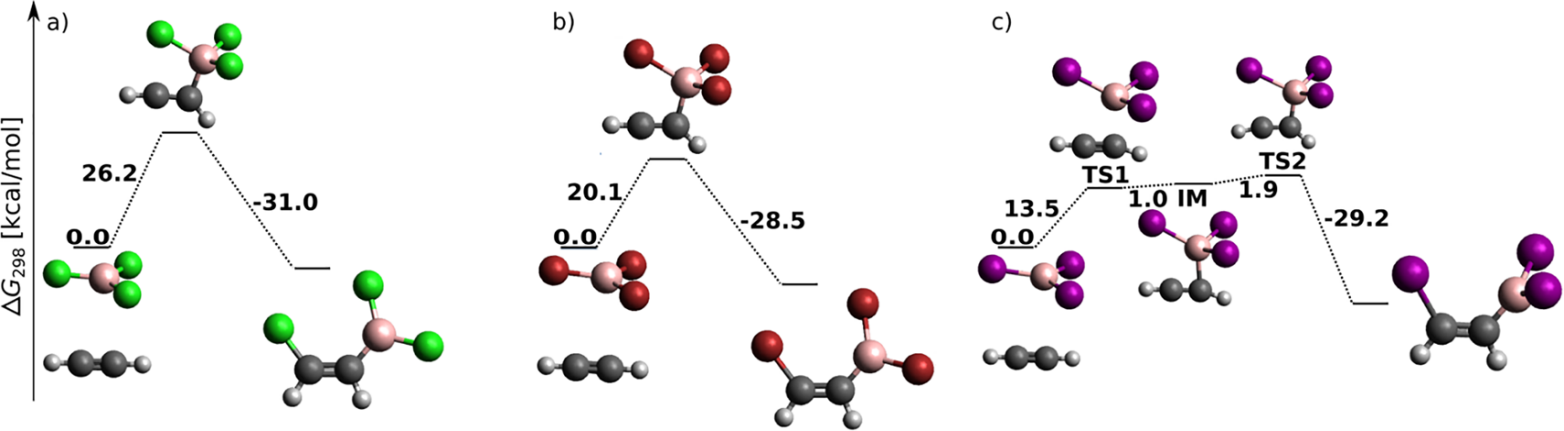
BX_3_*syn* addition to acetylene (H, white; B, pink; C, black; Cl, green; Br, red; I, purple) at the DLPNO-CCSD(T)/cc-pV5Z//MP2/6-31+G*_SVP level of theory: sum of electronic and thermal free energies (in kcal/mol) of local minima and transition structures.

An uncatalyzed *syn*-haloboration of acetylene is an electrophilic addition whose preliminary stage is a π-hole−π-electron interaction between boron and C_2_H_2_.^53^ The resulting loose van der Waals complex was chosen as the starting point of the reaction energy profile in Figure 1. In general, the total energy barrier decreases with increasing halogen proton number, in agreement with the increasing Lewis acidity of BX_3_.^54^ The reaction proceeds for BCl_3_ and BBr_3_ toward the product in a single step through a four-center TS (cf. Figure 1a,b). For BI_3_, a weakly bonded π-adduct intermediate (IM) was localized at the MP2 (not B3LYP-D3) level (cf. Figure S1), in agreement with the results of Wang and Uchyiama.^9^ The DLPNO-CCSD(T) results on MP2 geometries presented here, however, confirm that even for BI_3_, *syn*-haloboration proceeds in a single step.

#### 3.1.2 *anti*-Haloboration Catalyzed by X^–^

**Figure 2:**
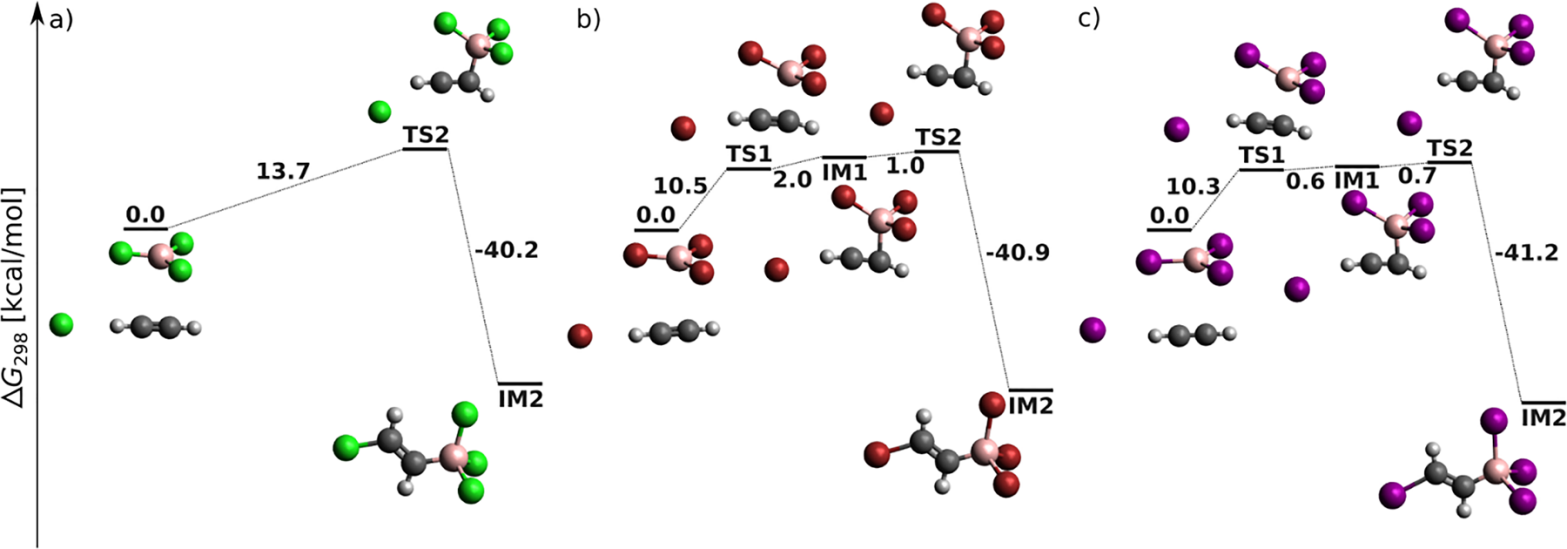
Opening of acetylene *anti*-haloboration catalyzed by X^–^ at the DLPNO-CCSD(T)/cc-pV5Z//MP2/6-31+G*_SVP level of theory: sum of electronic and thermal free energies in kcal/mol.

Figure 2 displays reaction profiles for the opening steps of acetylene *anti*-haloboration in the presence of an X^–^ catalyst. Formally, it is a termolecular reaction. However, since BX_3_ is used as the solvent (i.e., BX_3_ is in excess), the reaction is effectively a bimolecular process. The mechanism starts with the formation of the zwitterionic intermediate IM1.

First, BX_3_ adds to the triple bond to give a zwitterion whose positively charged carbon is attacked by the halide anion. The zwitterionic structure represents a shallow local minimum on the PES for X = I, and the total activation barrier decreases from 16 kcal/mol (X = Cl) to 10 kcal/mol (X = I). In the second step, halide anion binds to the positively charged carbon to give intermediate IM2 characterized by a quaternary boron atom. Because of steric hindrance and electrostatic repulsion, X^–^ approaches from the opposite side of acetylene than does the BX_3_ molecule. This results in the *E* configuration at the double bond of IM2 within a single-step reaction.

**Figure 3:**
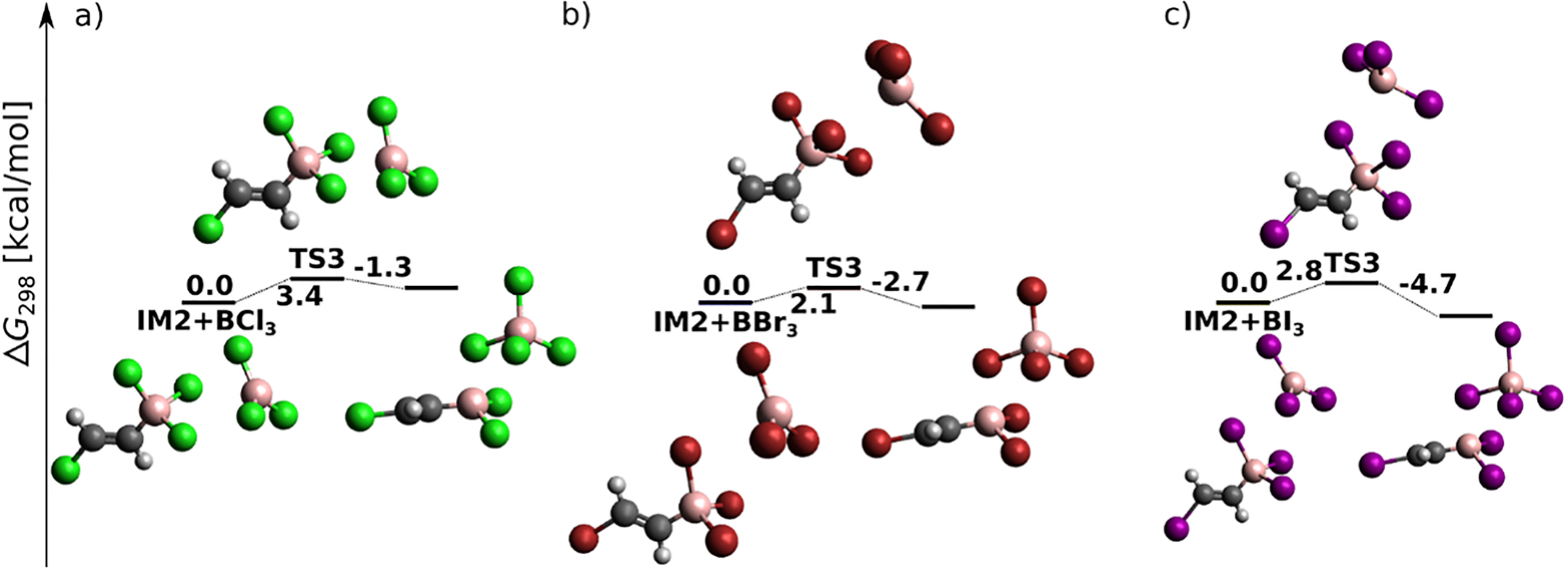
Closing step of acetylene *anti*-haloboration catalyzed by X^–^ at the DPLNO–CCSD(T)/cc-pV5Z//MP2/6-31+G*_SVP level—sum of electronic and thermal free energies.

**Scheme 2:**
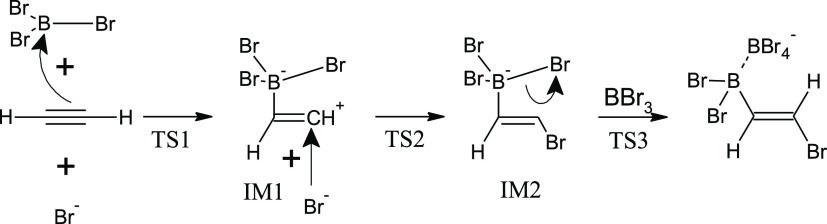
Proposed Mechanism of Acetylene *anti*-Bromoboration Catalyzed by Br^–^

In the last step, quaternary boron releases X^–^ upon assistance of another molecule of BX_3_ to give the *E* adduct and BX_4_^–^.^55,56^ Our results for the corresponding Gibbs free energy profiles are shown in Figure 3, and the complete mechanism is summarized in Scheme 2. Explicit solvation of X^–^ with BX_3_ is essential for splitting the B–X bond of IM2; in the absence of BX_3_, none of the PES scans performed provided any TS estimate, and the potential energy grew continuously. From this we conclude that the Lewis acidic orbital role of BX_3_ is crucial for the leaving halide stability while the electrostatic ion-pair-stabilizing role of BX_3_ is only of secondary importance. The results demonstrate that in the case of chloroboration, unlike for bromo- and iodoboration, the halide-leaving step is endergonic. This is consistent with a weaker Lewis acidity of BCl_3_ as compared to BBr_3_ and BI_3_. For Gibbs free energy profiles at the B3LYP-D3 and MP2 levels, see Figure S4a–c.

### 3.2 Polar Additions to Propyne

#### 3.2.1 *syn*-Haloboration

Before discussing chemical aspects of propyne *syn*-haloboration, we use its reaction mechanism for a method-comparison purpose. The reason for choosing this profile in particular is the high methodological (MP2 or B3LYP-D3) sensitivity of the reaction barriers. Unlike for the rest of the mechanisms, method-comparison data in Figure S5 are reported as electronic energies, i.e., they include neither ZPE or thermal contributions to enthalpy nor entropy contributions to Gibbs free energy. The main reason for doing so are high computational demands for obtaining the numerical Hessian at the DLPNO-CCSD(T) level of theory.

Figure S5 compares electronic energy profiles of propyne *syn*-chloro- and *syn*-bromoboration at the following levels of theory: MP2 electronic energies calculated on MP2-localized stationary-point structures are compared with DLPNO-CCSD(T) energies for the same (i.e., MP2-localized) structures. Similarly, B3LYP-D3 electronic energies calculated on B3LYP-D3-localized stationary points are compared with DLPNO-CCSD(T) energies for B3LYP-D3-localized structures. MP2 total activation barriers, in the sense of highest TS energy with respect to reactants, are overestimated by at most 3 kcal/mol with respect to the CCSD(T) reference. In case of B3LYP, they are underestimated by as much as 7 kcal/mol. Furthermore, MP2 correctly predicts the energies of bromo intermediate IM to lie below those of the neighboring transition states. In contrast, B3LYP predicts the existence of an intermediate for the chloro derivative, but the comparative CCSD(T) calculation shows that IM lies higher in energy than TS1, i.e., that the route from TS2 toward the reactant is barrierless. Based on these results, we consider the MP2/6-31+G*_SVP method superior to the B3LYP-D3/6-31+G*_SVP metod for the purpose of geometry optimization. CCSD(T) calculations are employed throughout this work for energy evaluation. Comparative MP2 and B3LYP energy data are given in the Supporting Information.

**Figure 4:**
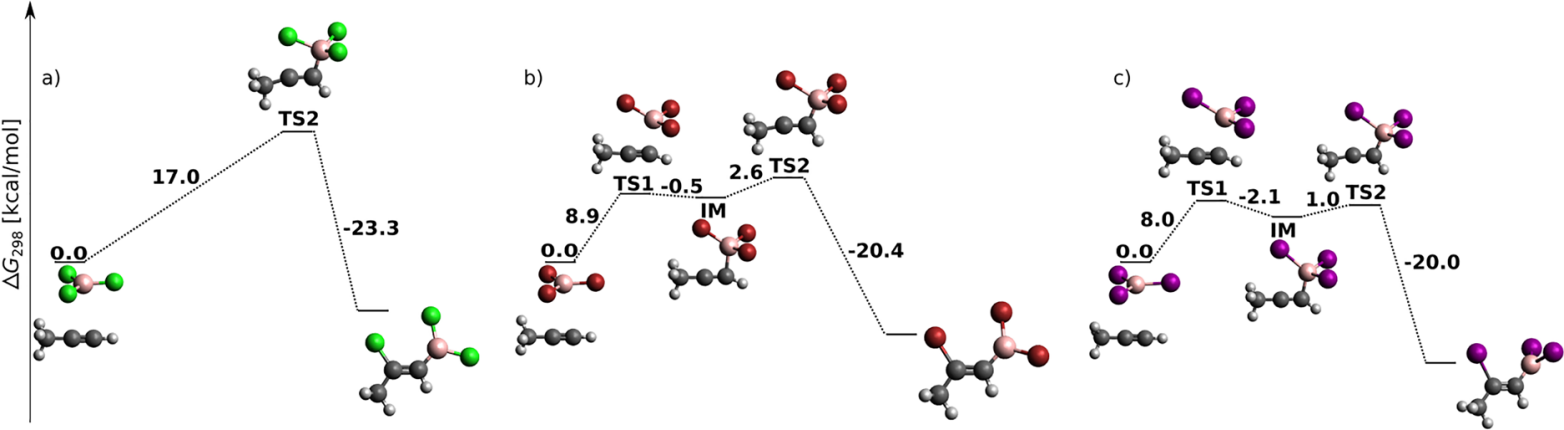
*Syn* addition of BX_3_ to propyne at the DLPNO-CCSD(T)/cc-pV5Z//MP2/6-31+G*_SVP level—sum of electronic and thermal free energies (in kcal/mol) of local minima and transition structures.

While *syn* addition to acetylene was (except for one case) a single-step reaction (cf. Figure 1), *syn*-haloboration of propyne is for most systems and methods a two-step reaction (cf. Figure 4). In terms of halogen influence, the total energy barrier decreases, in agreement with the increasing Lewis acidity of BX_3_. The presence of the additional CH_3_ group in propyne apparently strengthens disperse interactions between the halogen lone pairs and the π bonding electrons. As a result, the intermediate IM is (except for two cases) a local minimum on the PES, and the total energy difference between reactants and TS2 is smaller for each propyne haloboration than for the respective acetylene haloboration. For Gibbs free energy profiles at the B3LYP-D3 and MP2 levels of theory, see Figure S6a–c.

#### 3.2.2 *anti*-Haloboration Catalyzed by X^–^

**Figure 5:**
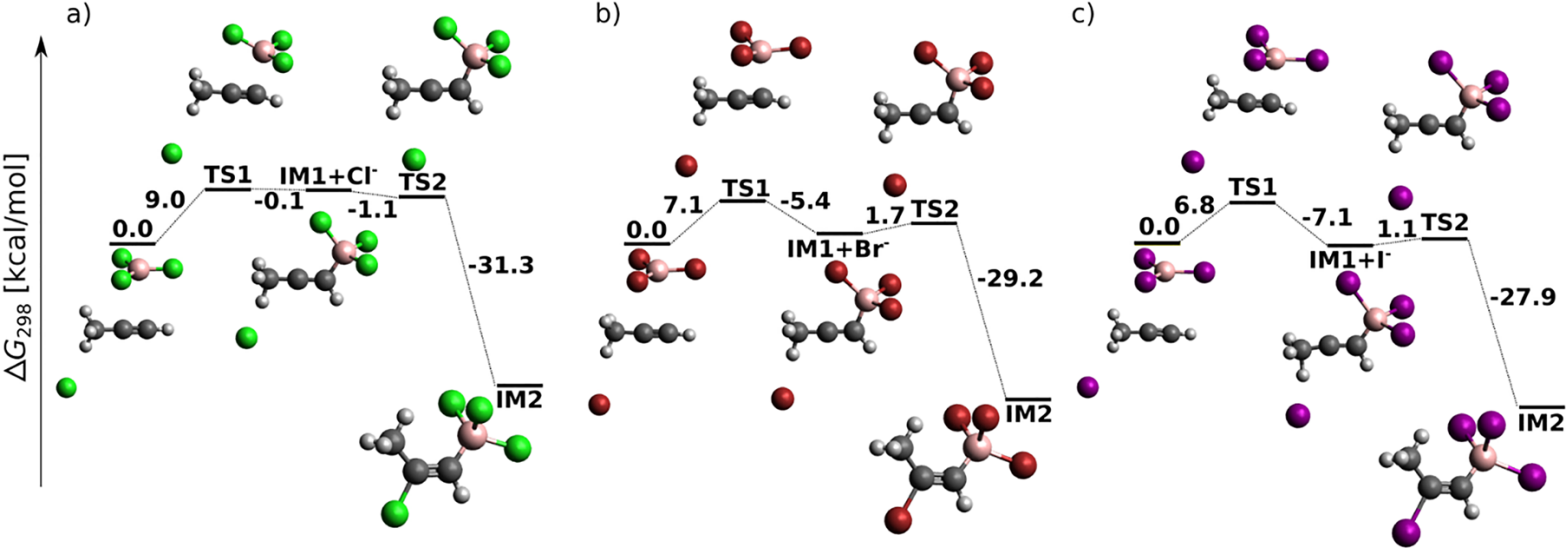
Opening of the halide-catalyzed propyne *anti*-haloboration mechanism—sums of electronic and thermal free energies at the DLPNO-CCSD(T)/cc-pV5Z//MP2/6-31+G*_SVP level of theory.

The first step of propyne *anti*-haloboration is analogous to its *syn* counterpart: boron attaches to the less substituted carbon of the triple bond. The structure of the resulting IM1 differs from its acetylene analogue by the additional CH_3_ group. Consequently, the approaching halide anion can act not only as a nucleophile but also as a base to abstract one of the CH_3_ protons, whose acidity is enhanced by the neighboring unsaturated positively charged carbon (vide infra). The reaction profile for the nucleophilic attack is given in Figure 5, which demonstrates the formation of the *anti*-haloborated propyne in two steps through the propyne–BX_3_ complex IM1. Note the continuously decreasing relative energy of the second transition state, TS2, with increasing halogen proton number. For Gibbs free energy profiles at the B3LYP-D3 and MP2 levels of theory, see Figure S8.

**Figure 6:**
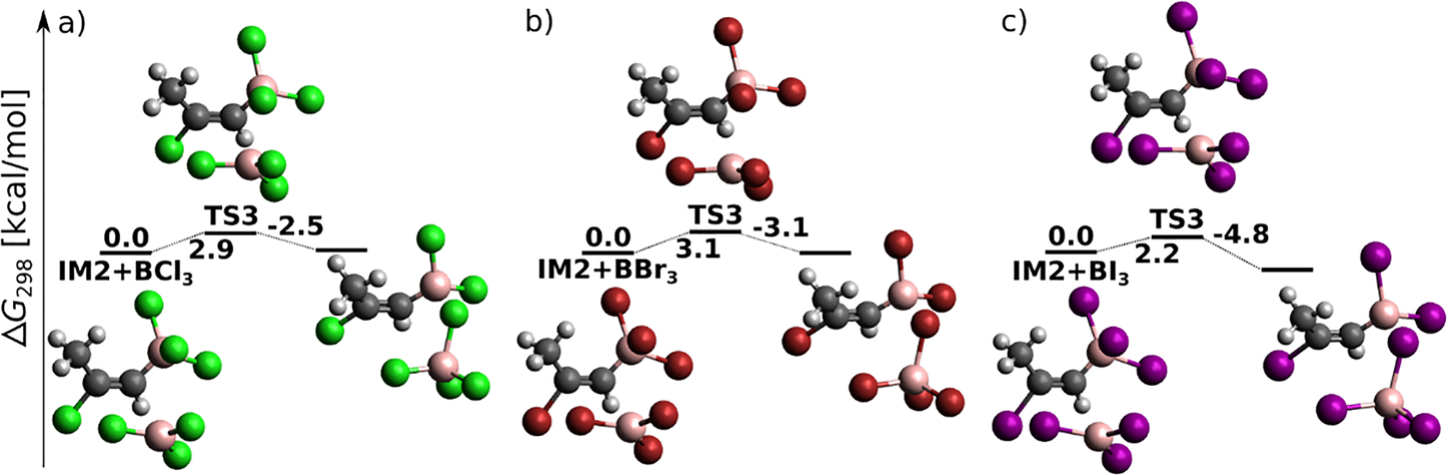
Closing of the halide-catalyzed propyne *anti*-haloboration mechanism—sums of electronic and thermal free energies at the DLPNO-CCSD(T)/cc-pV5Z//MP2/6-31+G*_SVP level of theory.

To abstract a halide anion from the quaternary boron in IM2, the participation of another molecule of BX_3_ is needed (Figure 6). While the MP2 results depicted in Figure S9 predict Δ_r_*G*° to change from +2 to −0.6 kcal/mol in going from X = Cl to X = I, B3LYP-D3 results copy the same trend with slightly more negative values of Δ_r_*G*°.

#### 3.2.3 Proton Abstraction from Propyne–BX_3_ Complex

**Figure 7:**
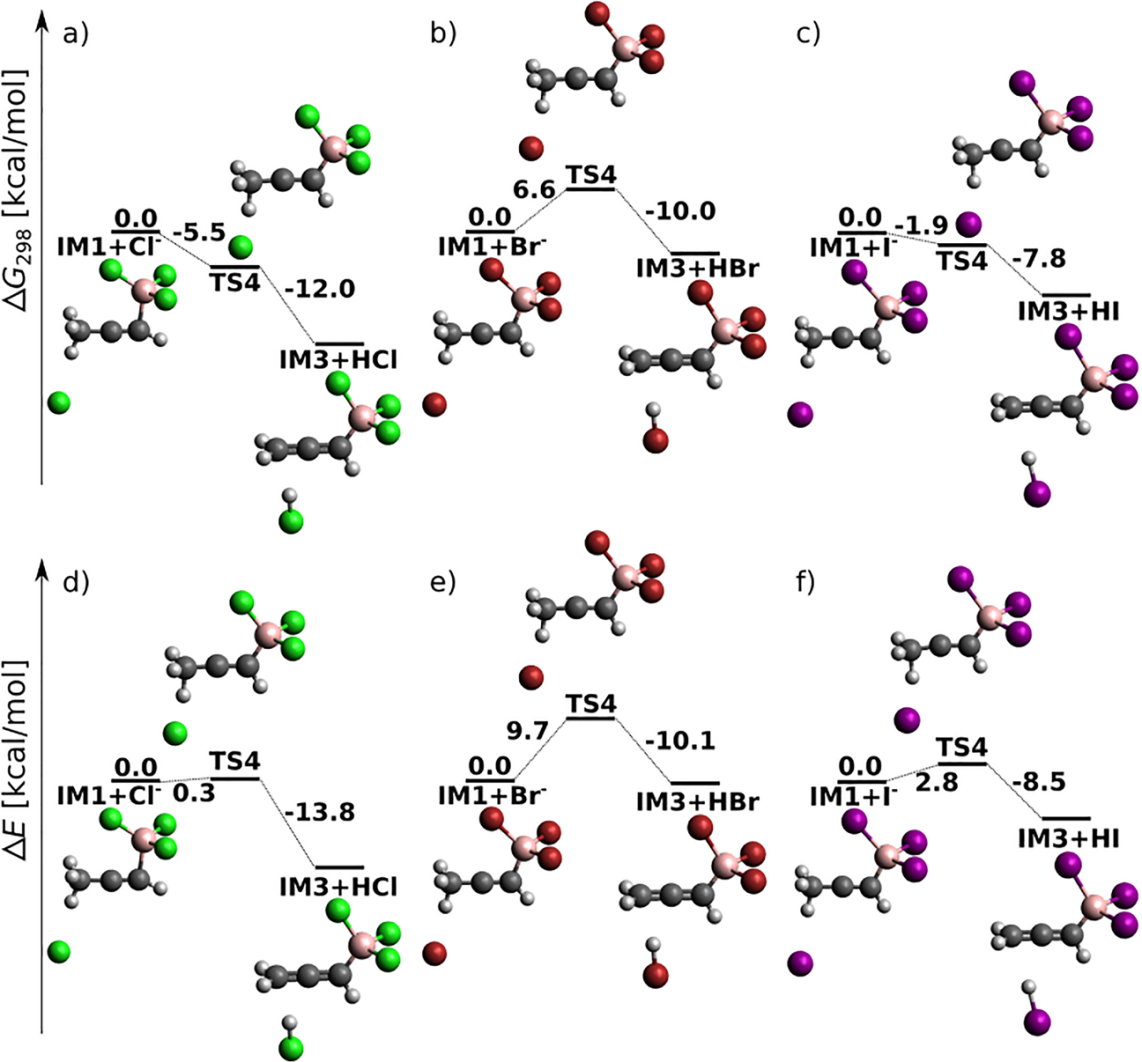
Opening of the alternative halide-catalyzed propyne *anti*-haloboration mechanism. (a–c) Sums of electronic and thermal free energies. (d–f) Electronic energies at the DLPNO-CCSD(T)/cc-pV5Z//MP2/6-31+G*_SVP level of theory.

Besides the nucleophilic attack on the propyne–BX_3_ complex, X^–^ can also behave as a base that abstracts the methyl proton to provide HX and an allenic intermediate IM3 (Figure 7; for B3LYP-D3 and MP2 results, see Figure S10). Obviously, in the case of Figure 7a,c the Gibbs activation energies are negative from the reactant side. We have therefore augmented Figure 7 with electronic energy profiles, which are already free from negative activation barriers. The improvement is expected to stem from the missing vibration correction inaccuracies. The related less-positive (compared to B3LYP-D3 and MP2) electronic energy activation barriers are expected to originate in MP2 geometry problems for the weakly bound reactant complexes.

**Figure 8:**
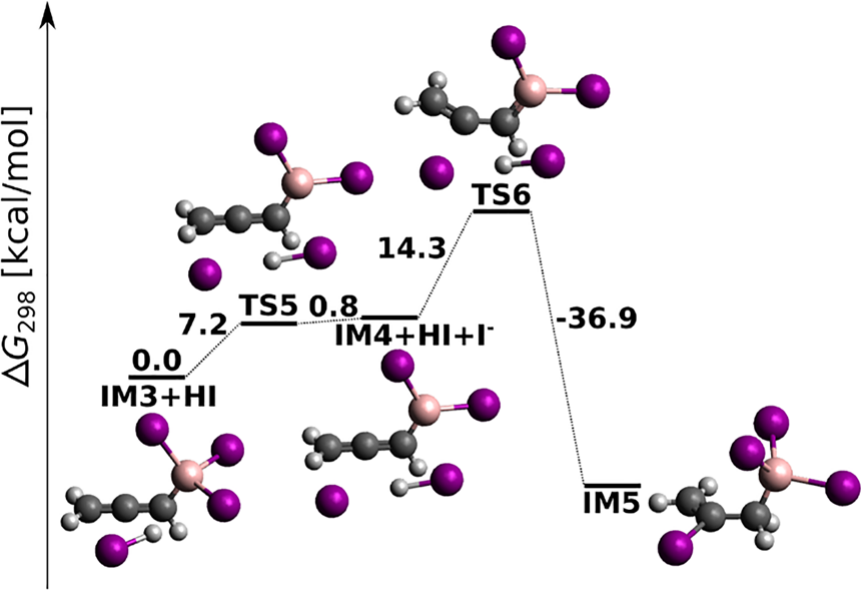
DLPNO-CCSD(T)/cc-pV5Z//MP2/6-31+G*_SVP reaction profile for the allenic π system of IM3 attacked by a HI molecule.

In order to explore reaction pathways to the *E* adduct thoroughly, for the case X^–^ = I^–^ we performed a PES scan for an interaction between the allenic π system of IM3 and a HI molecule with iodine coordinated to C2. As shown in Figure 8 (for MP2 results, see Figure S11), I^–^ can indeed bind to C2 to produce IM5, which is however chemically much distant from the desired *E* adduct. From this and from the high energy demands involved, we consider the route in Figure 7 a blind alley of *anti*-haloboration. As noted by a reviewer, BX_3_ and a base can give terminal alkyne deprotonation, but deprotonating actually the C–H and not the CH_2_R unit.^57^ Therefore, prior to the formation of the IM1 + X^–^ complex of Figure 5, BX_3_ and X^–^ can deprotonate the alkyne to provide an alkynyl trifluoroborate, which presents another competing reaction preventing the *anti*-haloboration.

### 3.3 Radical Reactions of Acetylene and Propyne

#### 3.3.1 Halovinyl Radical Formation and BX_3_ Attack

**Figure 9:**
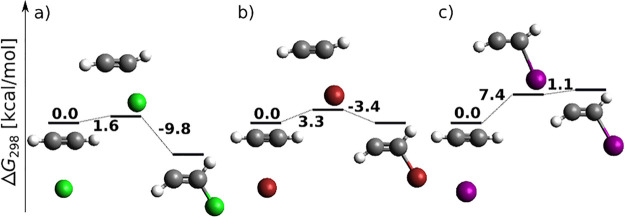
Formation of (a) (*Z*)-vinyl chloride, (b) (*Z*)-vinyl bromide, and (c) (*Z*)-vinyl iodide radical at the DLPNO-CCSD(T)/cc-pV5Z//MP2/6-31+G*_SVP level of theory—sum of electronic and thermal free energies in kcal/mol.

As discussed recently in our joint experimental and theoretical study of acetylene bromoboration,^12^ the most straightforward radical mechanism leading to (*E*)-dihalo(2-halovinyl)borane is BX_3_ addition on halovinyl radical. The latter can be formed by adding halogen radical (initially formed by HX homolysis) on acetylene. Our results for reaction Gibbs free energy profiles of (*Z*)-vinylhalide radical formation are shown in Figure 9. The standard reaction Gibbs free energy is positive for X = I. However, the following reaction with BX_3_ consumes the thermodynamically unstable (*Z*)-vinyl halide radical and thus supports the regeneration of the halogen radical that can further propagate this reaction pathway. B3LYP-D3 and MP2 results are displayed in Figure S14.

**Figure 10:**
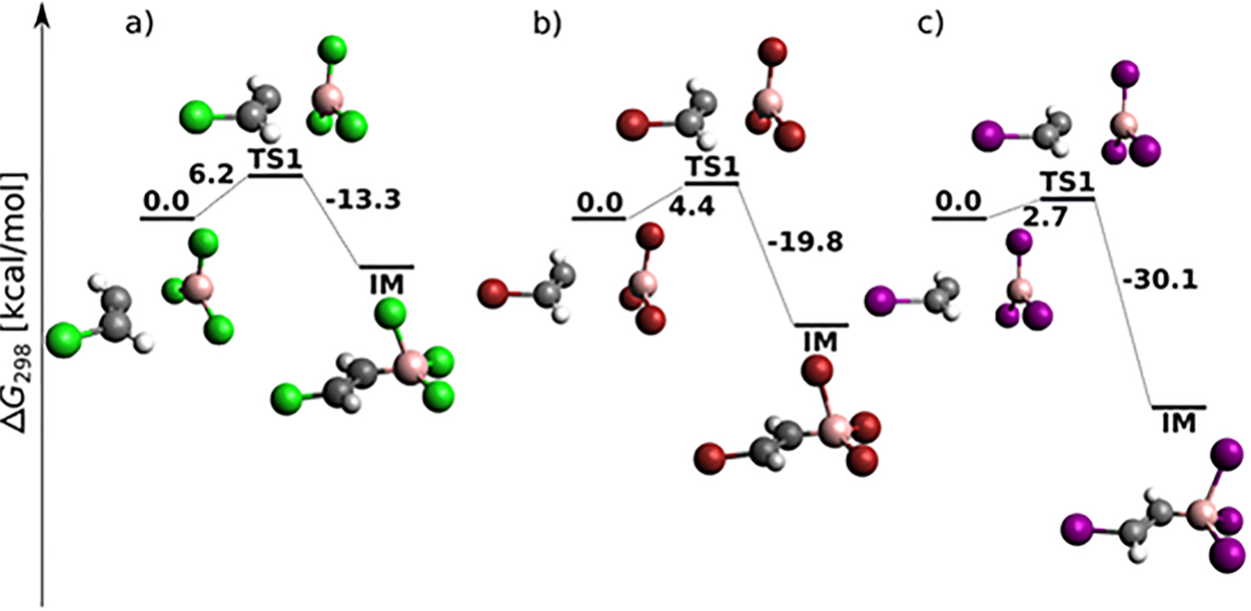
(*Z*)-Vinyl halide radical attack of BX_3_: reaction energy profiles at the DLPNO-CCSD(T)/cc-pV5Z//MP2/6-31+G*_SVP level.

The attack of the BX_3_ molecule on the (*Z*)-vinyl halide radical is shown in Figure 10. TS1 adopts *C*_*s*_ symmetry with one boron–halogen bond of BX_3_ eclipsed with the halovinyl unit and with the C^β^–C^α^–B angle of 119° for X = Cl and 118° for X = Br and I. This orientation is indicative of σ attack due to a maximum possible overlap between the sp^2^ hybrid orbital of C^α^ and the largely empty p orbital of boron. The latter is symmetrical with respect to the halovinyl plane, which prevents π attack by the C=C bond. The transition state transforms into IM via C–B bond formation and BX_3_ unit deformation, which reveals an interesting halogen dependence. While chlorine and bromine bend “toward” the halovinyl unit to create a triangular B–X–C arrangement, iodine bends away. Below we present a molecular orbital analysis of the halogen-bridged product formation for the case of X = Br.

**Figure 11:**
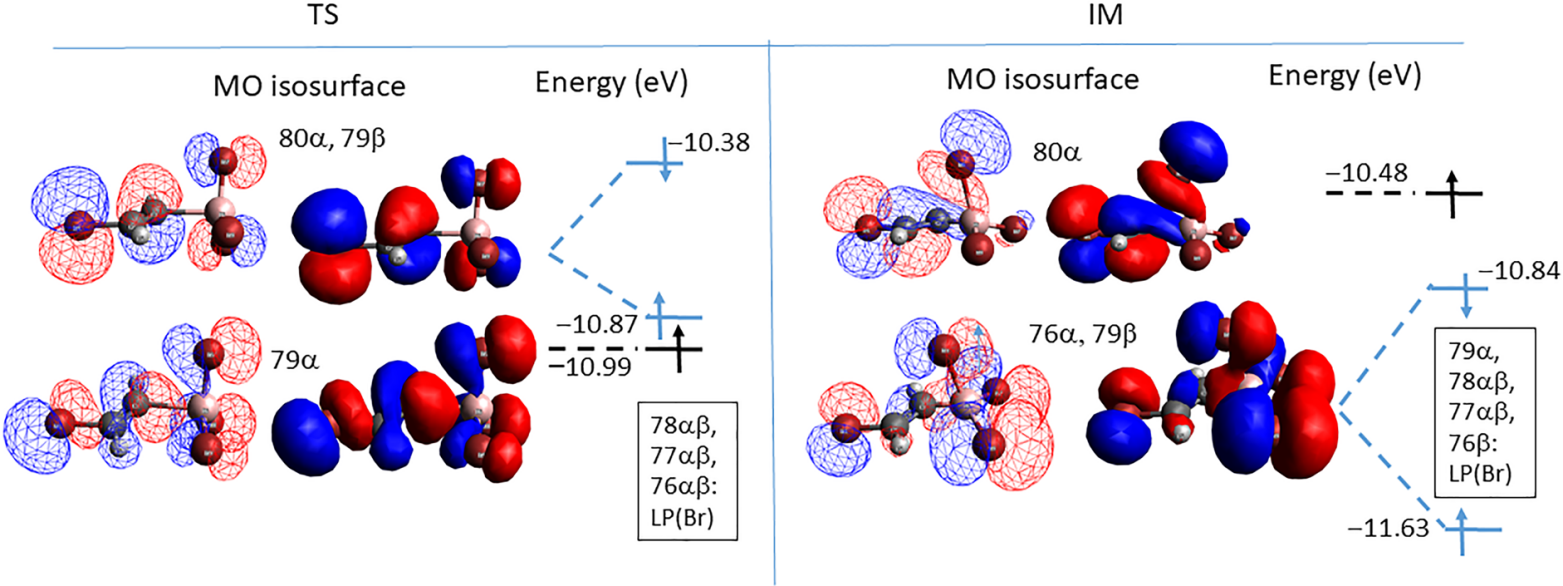
Frontier molecular orbitals of TS1 and IM for X = Br at the UHF/6-31+G*_SVP level. The character of orbitals listed in the rectangular field is dominantly that of linear combinations of Br lone pairs.

Key orbital interactions underlying Figure 10b are shown in Figure 11. Due to strong spin polarization, the singly occupied molecular orbital (SOMO) is represented by the second-highest orbital of α spin, 79α. According to the sums of net Mulliken basis function populations, the SOMO is dominated by an in-plane sp^2^ hybrid of C^α^ pointing at B and weakly binding H^α^ (34%). Further contributions are from the p orbitals of Br at C^β^ (24%) and from the two out-of-plane BBr_3_ bromines (each 10%). In summary, the 79α SOMO is characteristic of an early TS with most of the unpaired spin preserved in the vinyl bromide unit. The role of the (80α, 79β) pair is merely that of a π antibonding interaction between the vinyl bromide and BBr_3_ lone pairs.

When TS transforms into IM, its structure rearranges as follows: The C^α^–B distance contracts significantly (from 2.44 to 1.57 Å) while the C=C bond lengthens slightly (from 1.28 to 1.38 Å). The BBr_3_ unit undergoes pyramidalization and reorientates, giving rise to a triangular C^α^–Br–B arrangement with Br located over the newly formed C^α^–B bond (we denote this Br atom as Br^α^). Symmetry breaking enables an intermixing of previously orthogonal MOs, delivering the majority of spin in the SOMO (80α) to the C^β^ end (34% at Br^β^ and 25% at C^β^), while 29% is concentrated at Br^α^ and only 8% remains at C^α^. The (76α, 79β) orbital pair represents a doubly occupied MO with significant bonding character between the vinyl bromide and BBr_3_ units. Indeed, even though lone pairs of two “in-plane” BBr_3_ bromines dominate in the (76α, 79β) pair of Figure 11 (each contributing with 27%), “parallel” p orbitals of Br^α^ and C^α^ contribute as well (11% and 5%, respectively) and act as a pathway for π back-bonding.

**Scheme 3:**
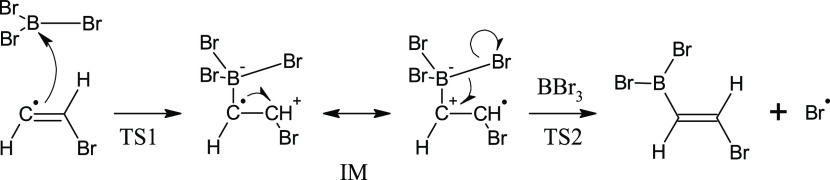
Mechanism of (*Z*)-Vinyl Bromide Radical Attack of BBr_3_ and Following Formation of *anti*-Haloborated Product

The content of Figure 11 can be related to a literature-based mechanistic view of borane reactivity toward radicals. According to Renaud et al.,^13^ homolytic substitution at boron does not proceed with C-centered radicals, but heteroatom-centered radicals react efficiently with organoboranes. This difference was attributed to the Lewis base character of the heteroatom-centered radicals, since the first step of the homolytic substitution is the formation of a Lewis acid–base complex.^13^ The double bond of the vinyl bromide radical can be considered as an alternative source of a Lewis base character, and the mechanism described in Scheme 3 can be constructed. In terms of TS geometry, our results contradict Scheme 3, which implies a π attack by electrons of the double bond. On the other hand, beyond the TS, π electrons of the C=C bond are symmetry-allowed and do participate in the delocalization from C^α^ to boron (cf. MO 80α of IM). Computed data also agree with Scheme 3 in that they predict the unpaired electron to remain localized at C^α^ in the TS and shift to C^β^ afterward. Finally, Scheme 3 correctly describes the subsequent shift of electron density from the B–Br bond on C^α^ (a π back-donation from Br^α^ to C^α^) and a spin density concentration at Br^α^. In summary, our results agree with Scheme 3 except for a σ-attack step that is missing at the beginning of Scheme 3 but consistent with our computation.

#### 3.3.2 *anti*-Haloborated Product Formation

**Figure 12:**
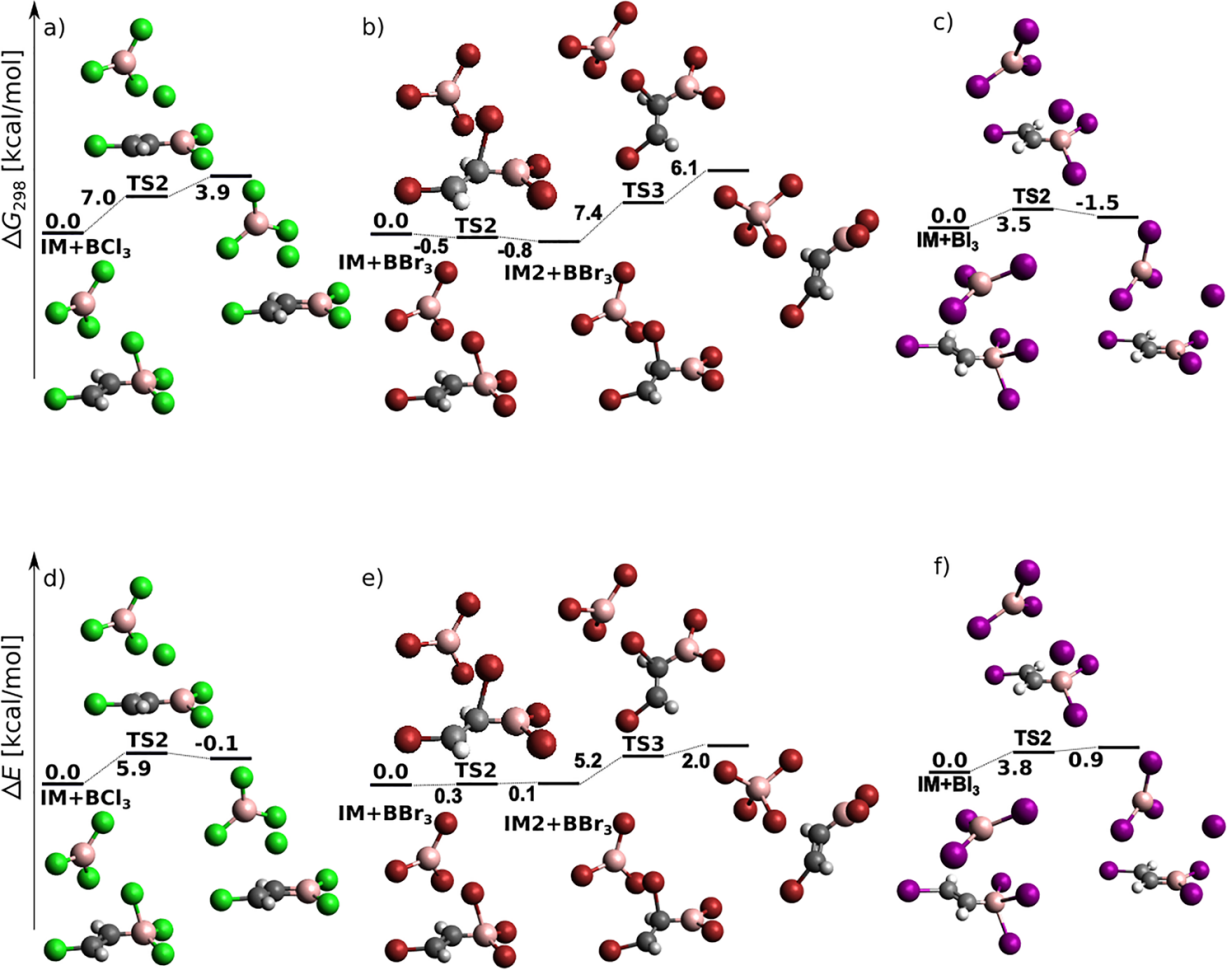
*anti*-Haloborated product formation following (*Z*)-vinyl halide radical attack of BX_3_ at the DLPNO-CCSD(T)/cc-pV5Z//MP2/6-31+G*_SVP level of theory. (a–c) Sum of electronic and thermal free energies in kcal/mol. (d–f) Electronic energies.

Figure 12 displays energy profiles for the final step of Scheme 3 (splitting of the B–X bond and *E* adduct formation) for X = Cl and Br. Clearly, in Figure 12a,b the Gibbs activation energies are sizably negative from the product side (and slightly negative from the reactant side in Figure 12b). We have therefore augmented Figure 12 with electronic energy profiles, for which the problem becomes less severe but partially persists. While the improvement is expected to stem from the missing vibration correction inaccuracies, persisting unphysical behavior is expected to originate in MP2 geometry problems for the weakly bound product complexes.

Two possible reaction paths are covered: in Figure 12a, halogen leaves from the boron atom in just a loose coordination to the BX_3_ molecule, which preserves a close-to-planar geometry. In Figure 12b, a two-step mechanism is followed: halogen first skips to the α-carbon, and from there it leaves as a part of a tetrahedral BBr_4_ radical. From the point of view of activation barriers, a direct splitting of halogen from boron is preferred. This endergonic step is again triggered by a coupled propagation reaction in which halogen radical attacks a fresh acetylene molecule. MP2 energy profiles are displayed in Figure S16. Energy profiles for direct splitting of bromine from boron at additional levels of theory are displayed in Figure S17.

#### 3.3.3 Radical Reactions of Propyne Providing Haloborated Products

**Figure 13:**
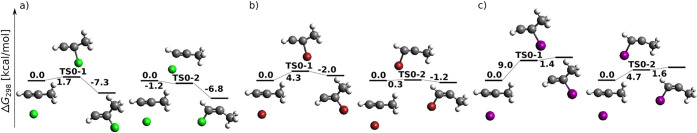
Comparison of (a) (*Z*)-2-chloropropen-1-yl and (*Z*)-1-chloropropen-2-yl radical formation, (b) (*Z*)-2-bromopropen-1-yl radical and (*Z*)-1-bromopropen-2-yl radical formation, and (c) (*Z*)-2-iodopropen-1-yl and (*Z*)-1-iodopropen-2-yl radical formation at the DLPNO-CCSD(T)/cc-pV5Z//MP2/6-31+G*_SVP level of theory—sum of electronic and thermal free energies in kcal/mol.

Unlike for acetylene, a halogen radical attack on propyne can result in two possible products: a (*Z*)-2-halopropen-1-yl radical and a (*Z*)-1-halopropen-2-yl radical. Experimentally, however, halogen atom is known to attack the less substituted carbon of the alkyne, which is understood in terms of greater stability of the resulting radical.^58^ This textbook rule is illustrated by our results for energy barriers and product stabilities in the case of Cl, Br, and I addition on propyne in Figure 13. Energy barriers for Cl, Br, and I addition on the terminal carbon are in all cases lower than in the case of the central carbon. Additionally, the (*Z*)-1-halopropen-2-yl radical is in all cases the more stable one. Propyne attack reactions are increasingly endergonic for heavier halogens but are expected to be triggered by radical consumption in further steps of the mechanism. MP2 energy profiles are displayed in Figure S18.

**Figure 14:**
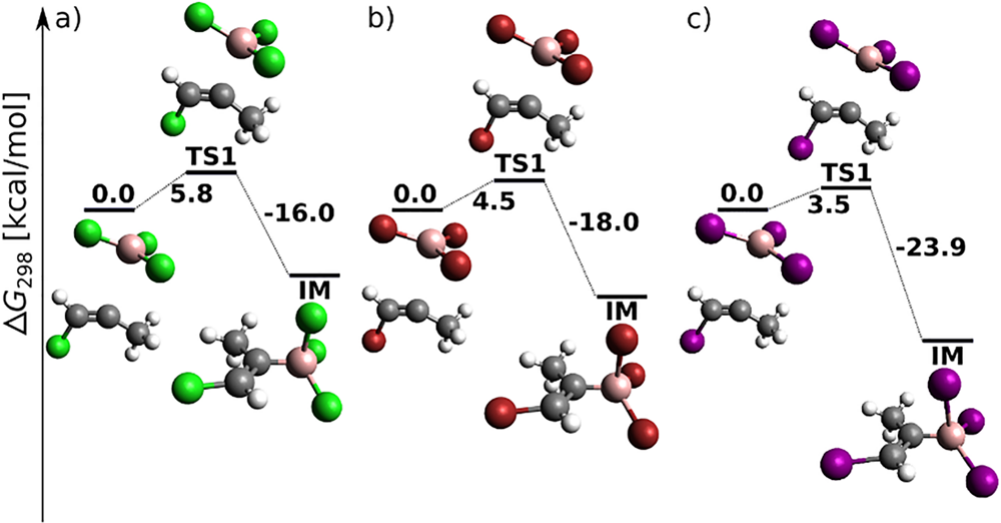
Halopropene radical addition on BX_3_ at the DLPNO-CCSD(T)/cc-pV5Z//MP2/6-31+G*_SVP level of theory—sum of electronic and thermal free energies in kcal/mol.

**Figure 15:**
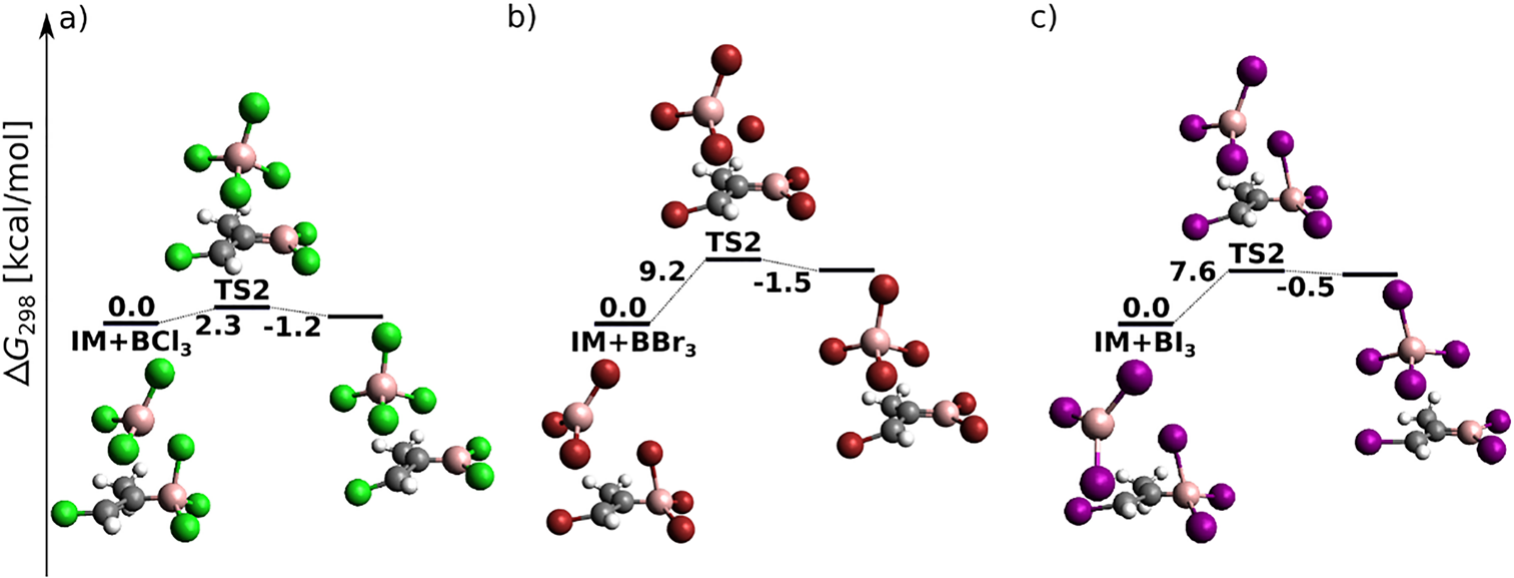
Excessive halogen splitting following halopropene radical addition on BX_3_ at the DLPNO-CCSD(T)/cc-pV5Z//MP2/6-31+G*_SVP level—sum of electronic and thermal free energies in kcal/mol.

**Scheme 4:**
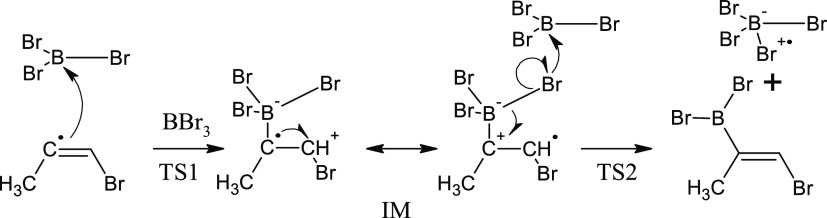
Proposed Mechanism of (*E*)-Dibromo(1-bromopropen-2-yl)borane Formation from 1-Bromopropen-2-yl Radical and BBr_3_

Based on the above-mentioned results, the consecutive reactions of the radical haloboration mechanism were only explored for the (*Z*)-1-halopropen-2-yl radicals. Our results for reaction Gibbs free energy profiles have been divided into two parts: the first step involving halopropene radical addition on BX_3_ (Figure 14; for B3LYP-D3 and MP2 results, see Figure S19) and the second step involving excessive halogen atom splitting (Figure 15; for B3LYP-D3 and MP2 results, see Figure S20 and S21). The complete mechanism is summarized in Scheme 4.

The second step of (*Z*)-1-halopropen-2-yl radical haloboration (Figure 15) is endergonic but can be triggered by a propagation step in which the BX_4_ radical attacks a fresh propyne molecule, producing (*Z*)-1-halopropen-2-yl and BX_3_. The reaction mechanism proceeds along a similar path as in the case of acetylene haloboration product formation shown in Figure 12. Note, however, that while the intermediates of Figure 12 correspond to a bridgelike arrangement of B–X–C atoms for chloro and bromo derivatives, in IM structures of Figure 15 this is so for bromo and iodo derivatives. For the remaining structures, boron adopts a genuinely tetrahedral coordination.

Obviously, due to the regiochemistry of the initiating halopropenyl radicals, only haloboration products bearing the halogen at the less substituted carbon are obtained from our calculations. However, experimental studies report haloboration adducts bearing the halogen at the more substituted carbon.^3^ From this, we conclude that the radical mechanism most probably does not operate in the case of higher alkyne haloboration.

## 4 Conclusions and Outlook

The mechanism of alkyne haloboration has been studied by means of B3LYP-D3, MP2, and DLPNO-CCSD(T) methods with an emphasis on direct pathways toward the *anti*-haloborated adduct. For X = Cl, Br, and I, interactions between BX_3_ and acetylene or propyne with and without an additional X^–^ anion, as well as between BX_3_ and halovinyl or (*Z*)-1-halopropen-2-yl radical, have been modeled. A bimolecular reaction between acetylene or propyne and the BX_3_ molecule produces the *Z* adduct through a four-center transition state in a single-step process.

In the presence of X^–^, acetylene and BX_3_ form the *E* adduct in a two-step sequence. First, BX_3_ adds to the triple bond to give a zwitterion whose positively charged carbon is attacked by the halide anion, at a total cost of 7–9 kcal/mol. In the second step, the “excessive” halide anion from the BX_3_^–^ group leaves with another solvent molecule under mild energy requirements of 2–3 kcal/mol. If BX_3_, in the presence of X^–^, is added not to acetylene but to propyne, the first step differs only in a substantial decrease in activation barriers for chloro and iodo derivatives. The process is essentially barrierless. Two possibilities for the zwitterion reactivity follow: X^–^ can attack the positive carbon as a nucleophile or as a base. The former option continues to the *E* adduct through two steps, of which the second requires additional BX_3_ molecule participation and is endergonic. The latter option provides an allenic moiety in a single exergonic step. Thus, the polar way of propyne *anti*-haloboration is unlikely due to competitive allene generation.

A similar stereochemical message as for acetylene versus propyne in polar mechanisms is given by free radical mechanisms. Halogen radical attack of acetylene produces (*Z*)-2-vinyl halide radical, which subsequently reacts with BX_3_ to provide a bromine-bridged intermediate. The *E* adduct is then formed via splitting of the excessive halogen radical by binding it on a second BX_3_ molecule (or possibly on acetylene). When it comes to propyne, the only initiation process compatible with experimental and computed data is (*Z*)-1-halopropen-2-yl radical formation. Upon BX_3_ addition, it can provide an *anti*-haloboration adduct but with regioselectivity different from that reported in experimental studies. Since the radical route for the propyne *anti*-haloboration is excluded and the polar mechanism is unlikely to proceed, it can be expected that the *Z*/*E* mixtures reported in experiments for some higher alkynes result from *Z*/*E* isomerization rather than from *anti* addition.

On the other hand, both polar and radical mechanisms are likely to produce *E*-haloborated adducts in the case of acetylene. Radical activation barriers for the rate-determining step are ca. 2.5 times lower, which along with experimental results in ref (12) indicates radical formation of (*E*)-dibromo(2-bromovinyl)borane.

Important points made by a reviewer should be considered in potential follow-up work. In the presence of excess BX_3_, the concentration of free halide in solutions of BBr_3_ will be very low. Thus, the [BX_4_]^−^ anion could be the source of halide for *anti*-haloboration. Assessing the energetic feasibility of *anti*-haloboration upon [BX_4_]^−^ (instead of X^–^) catalysis is beyond the scope of the current study but definitly deserves to be explored in future studies.

## Supporting Information Available

Computational details; results of benchmarking calculations, supplementary reaction profiles; electronic energies, thermal corrections to enthalpy and to Gibbs energy at 298 K, imaginary frequencies; optimized Cartesian coordinates of transition states, intermediates, and products from calculations (PDF).
