# Supplementary material for: Mechanistic Analysis of Alkyne Haloboration: A DFT, MP2, and DLPNO-CCSD(T) Study
Source: J Phys Chem A. 2023 Jul 25;127(30):6135–46. doi: 10.1021/acs.jpca.3c00607 (PMC10405270; doi:10.1021/acs.jpca.3c00607)
Supplement: Supplementary file 1 — jp3c00607_si_001.pdf [file jp3c00607_si_001.pdf]

**Supporting Information for:**

# Mechanistic Analysis of Alkyne Haloboration: A DFT and MP2 Study

Jakub Stošek, Hugo Semrád, Ctibor Mazal, and Markéta Munzarová

*Department of Chemistry, Faculty of Science, Masaryk University, Kotlářská 2, 611 37 Brno, Czech Republic*

## Table of Contents

**1. Quantum Chemical Calculations ........ S2**

Computational Details: General Information ........ S2

Benchmarking calculations for polar *syn*- and *anti*-additions ........ S3

B3LYP and MP2 reaction profiles for polar *anti*- and radical additions ........ S8

Absolute energy contributions referring to Figures ........ S17

Optimized Cartesian Coordinates ........ S38

**2. References ........ S145**

## Quantum Chemical Calculations

## Computational Details: General Information

Starting structures have been prepared using the Avogadro 1.1.1 software.[1] Structures have been optimized at MP2[2–7] level of theory as implemented in Gaussian09, rev. D.01[8] and Gaussian16, rev. C.01[9] for the cases of Figures 10c, 12c,f, 15b,c, and Figure 13c (reactant complex only). Single-point calculations of electronic energies on optimized structures have been performed by means of DLPNO-CCSD(T) method[10–13] using Orca, vers. 5.0.3.[14] at B3LYP-D3 and MP2 levels, Benchmark calculations have been carried out at the B3LYP[15–17] and MP2 levels of theory as implemented in Gaussian09, rev. D.01 and Gaussian16, rev. C.01 for the cases of Figures 10c, 12c,f, 15b,c, and Figure 13c (reactant complex only).

The GD3BJ dispersion correction of Grimme[18] has been employed in all DFT calculations, below denoted as B3LYP-D3. For Br and I, SVP basis sets of Ahlrichs[19,20] were used in combination with the 6-31+G* basis set of Pople[21–28] for H, B, C and Cl (further labeled as 6-31+G*_SVP basis set). Additionally, MP2 calculations have been performed with the Def2TZVPP extended basis set,[29,30] while B3LYP-D3 all-electron results for iodine compounds have been compared with the small core, ECP46MWB pseudopotential approach[31] with the corresponding recommended orbital basis set of DZP quality.

The DLPNO-CCSD(T) single-point calculations were performed with the cc-pV5Z orbital basis and cc-pV5Z/C auxiliary basis of Dunning et al.[32–34] with the exception of iodine atoms in iodo-derivatives. For the latter, the cc-pV5Z-PP orbital basis and cc-pVQZ-PP/C auxiliary basis of Dunning et al. Along with the SK-MCDHF-RSC pseudopotential[35] has been emloyed.

Implicit SCRF model of solvation using $CH_2Cl_2$ has been employed in all calculations with the “pcm” (Gaussian) or “cpcm” option (Orca).

The calculations have been run on the computers of the “MetaCentrum” Czech supercomputing center[36].

B3LYP calculations have been employed for transition state searching using the single coordinate driving method, scanning either a bond length or a bond angle. Transition state searching was performed either from reactants or the products, exploring a larger set of local minimum structures, until appropriate TS guess has been localized. Transition state optimizations as well as optimizations to all local minima have been performed at MP2 and B3LYP levels of theory as described in text. Unless stated otherwise, reaction profiles from both the MP2 and the B3LYP methods were qualitatively same. Harmonic vibrational frequency calculations were performed for all of the stationary points to determine whether they are local minima or transition structures and to derive the thermochemical corrections for the enthalpies and free energies. The correspondence of the transition states to the assigned local minima was proved by an inspection of imaginary frequencies, by a small geometry distortion along imaginary frequency directions followed by geometry optimizations, and by an IRC calculation from transition state in both directions.

## Benchmarking calculation for polar *syn*- and *anti*-additions

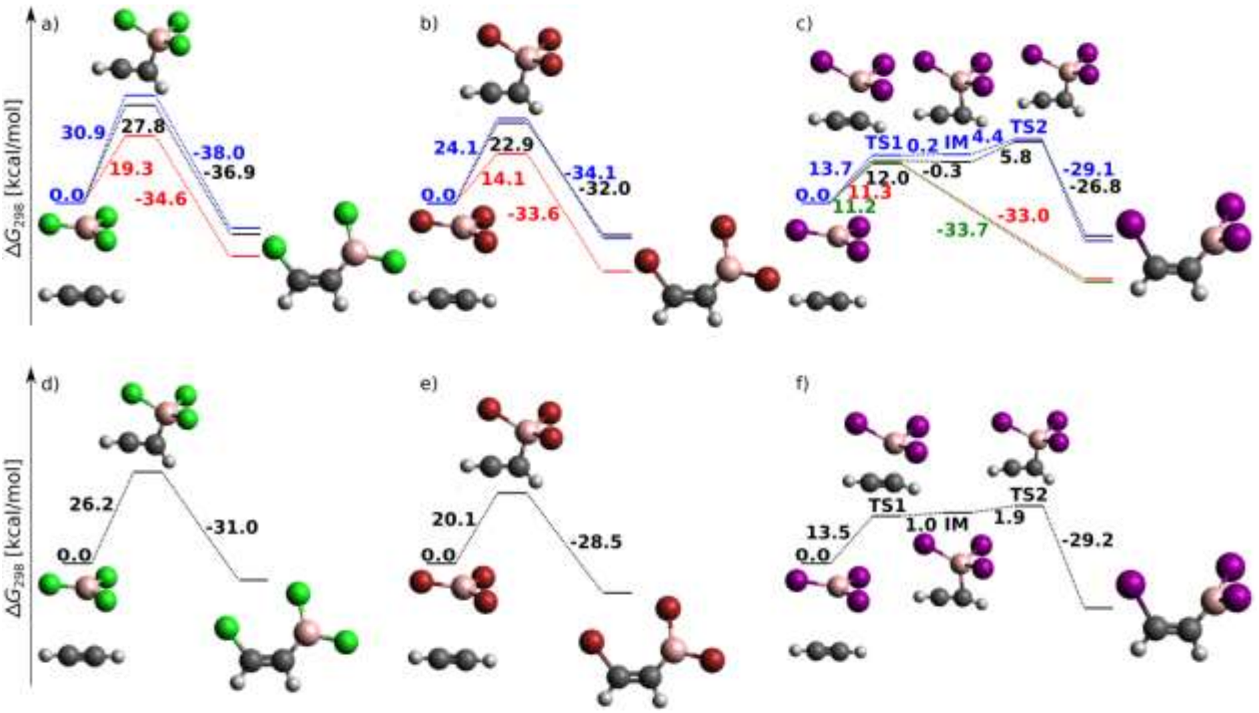


**Figure S1.** $BX_3$ *syn*-addition to acetylene (H – white, B – pink, C – black, Cl – green, Br – red, I – purple). a-c) at MP2/6-31+G*_SVP (blue), at MP2/Def2TZVPP (black), at B3LYP-D3/6-31+G*_SVP (red) and at B3LYP-D3/ECP46MWB levels (green), d-f) at DLPNO-CCSD(T)/cc-pV5Z//MP2/6-31+G*_SVP level of theory – sum of electronic and thermal free energies (in kcal/mol) of local minima and transition structures.

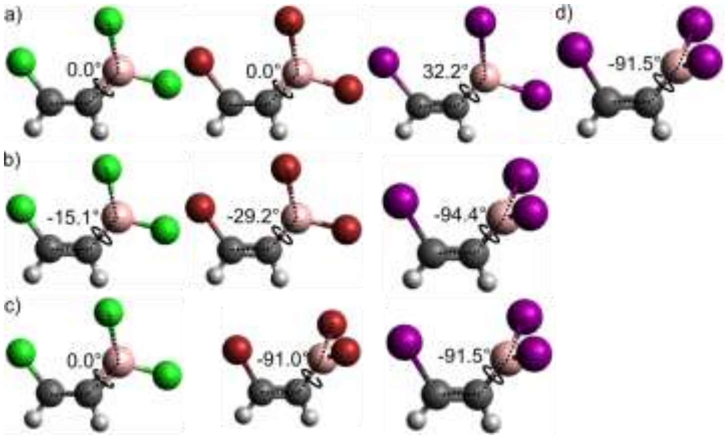


**Figure S2.** The C–C–B–X dihedral angles (indicated by the dotted lines) in the structures of *Z*-adduct for an addition of $BX_3$ to acetylene at a) B3LYP/6-31+G*_SVP, b) MP2/6-31+G*_SVP, c) MP2/Def2TZVPP and d) B3LYP/ECP46MWB levels.

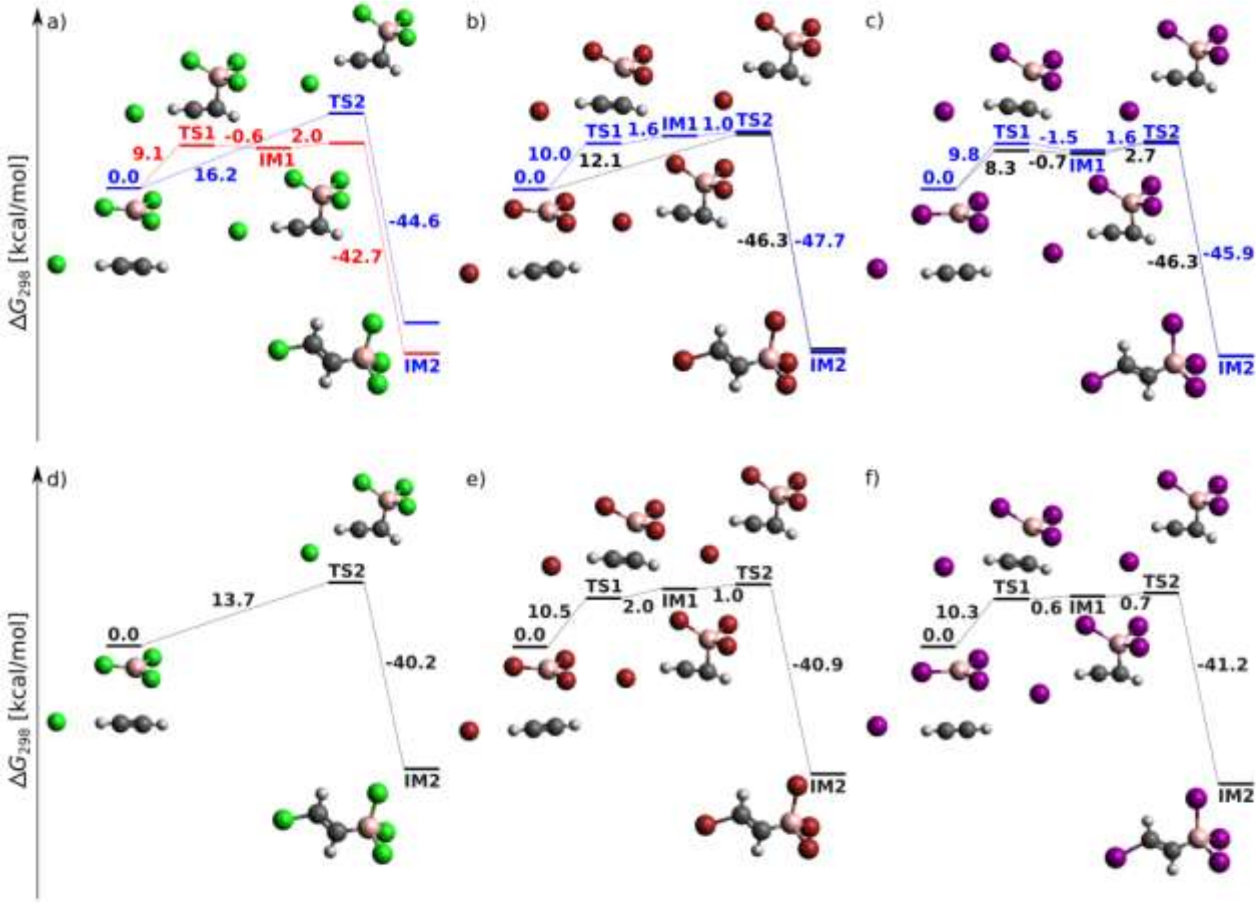


**Figure S3.** Opening of acetylene *anti*-haloboration catalyzed by X⁻. a-c) at MP2/6-31+G*_SVP (blue), MP2/Def2TZVPP (black) and B3LYP-D3/6-31+G* (red) level of theory, d-f) at DLPNO-CCSD(T)/cc-pV5Z//MP2/6-31+G*_SVP level of theory. Sum of electronic and thermal free energies in kcal/mol.

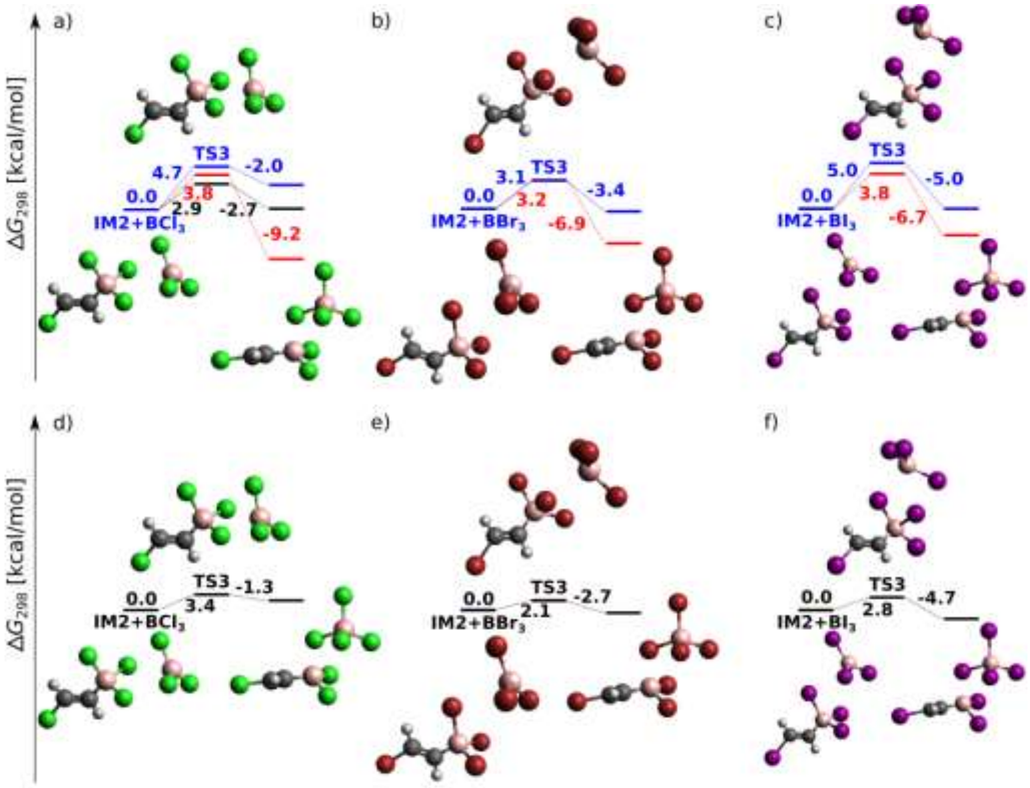


**Figure S4.** Closing step of acetylene *anti*-haloboration catalyzed by $X^{-}$. a-c) at MP2/6-31+G*_SVP (blue), MP2/Def2TZVPP (black) and B3LYP-D3/6-31+G*_SVP (red) level, d-f) at DLPNO-CCSD(T)/cc-pV5Z//MP2/6-31+G*_SVP level of theory – sum of electronic and thermal free energies.

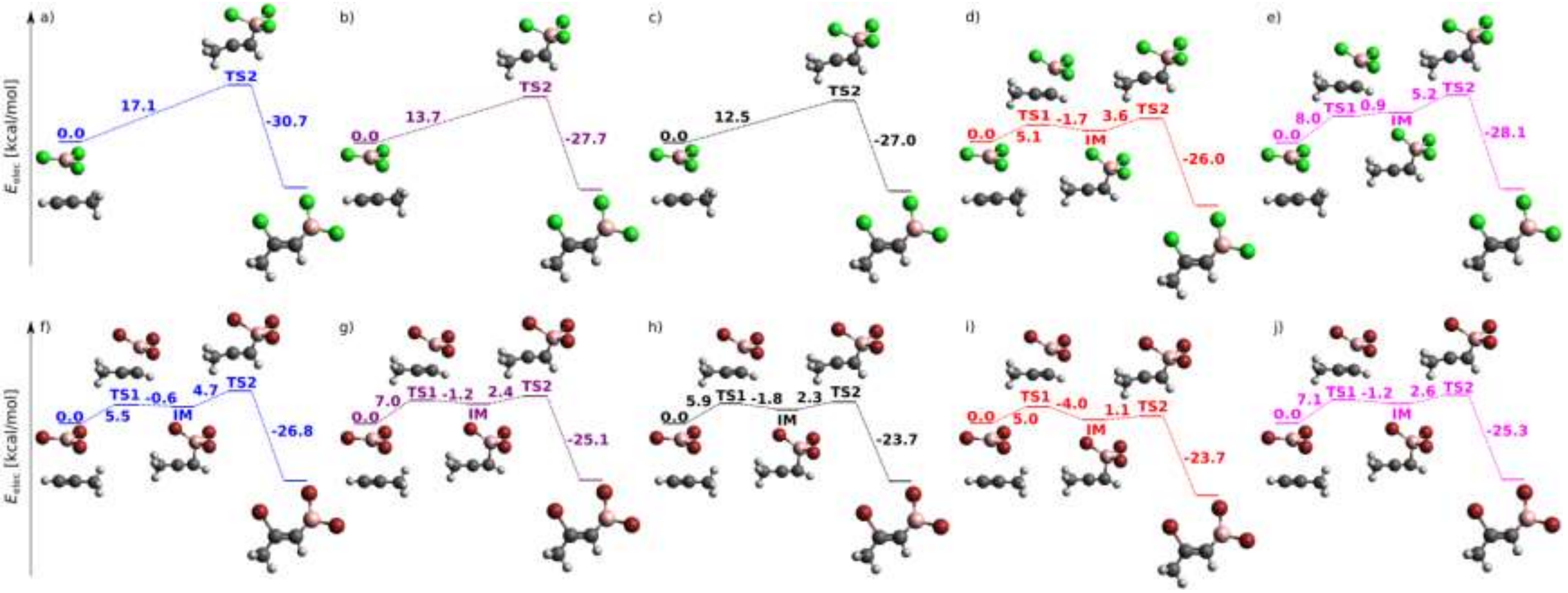


**Figure S5.** *Syn*-addition of $BX_3$ to propyne at a) MP2/6-31+G*_SVP//MP2/6-31+G*_SVP for X = Cl, b) DLPNO-CCSD(T)/cc-pVTZ//MP2/6-31+G*_SVP for X = Cl, c) DLPNO-CCSD(T)/cc-pV5Z//MP2/6-31+G*_SVP for X = Cl, d) B3LYP-D3/6-31+G*_SVP//B3LYP-D3/6-31+G*_SVP for X = Cl, e) DLPNO-CCSD(T)/cc-pVTZ//B3LYP-D3/6-31+G*_SVP for X = Cl, f) MP2/6-31+G*_SVP//MP2/6-31+G*_SVP for X = Br, g) DLPNO-CCSD(T)/cc-pVTZ//MP2/6-31+G*_SVP for X = Br, h) DLPNO-CCSD(T)/cc-pV5Z//MP2/6-31+G*_SVP for X = Br, i) B3LYP-D3/6-31+G*_SVP//B3LYP-D3/6-31+G*_SVP for X = Br and j) DLPNO-CCSD(T)/cc-pVTZ//B3LYP-D3/6-31+G*_SVP for X = Br – Electronic energies of local minima and transition structures.

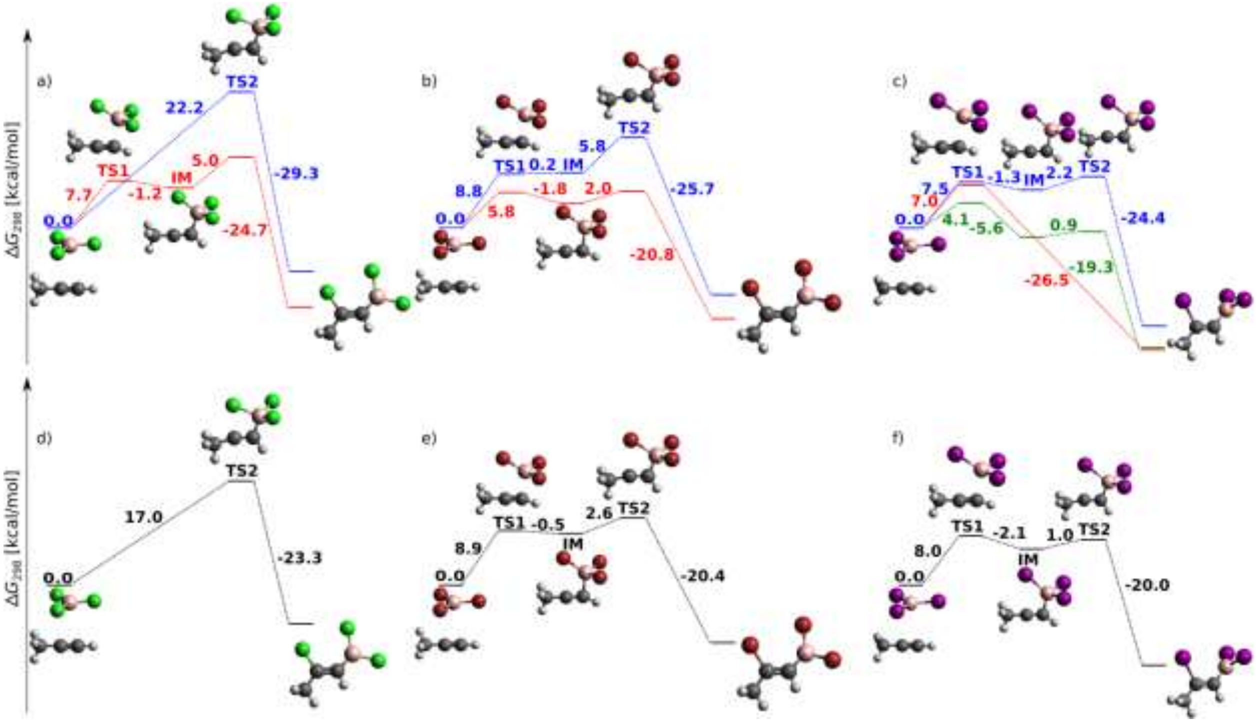


**Figure S6.** *Syn*-addition of $BX_3$ to propyne. a-c) at MP2/6-31+G*_SVP (blue), B3LYP-D3/6-31+G*_SVP (red) and at B3LYP-D3/ECP46MWB (green) levels, d-f) at DLPNO-CCSD(T)/cc-pV5Z//MP2/6-31+G*_SVP level of theory – sum of electronic and thermal free energies (in kcal/mol) of local minima and transition structures.

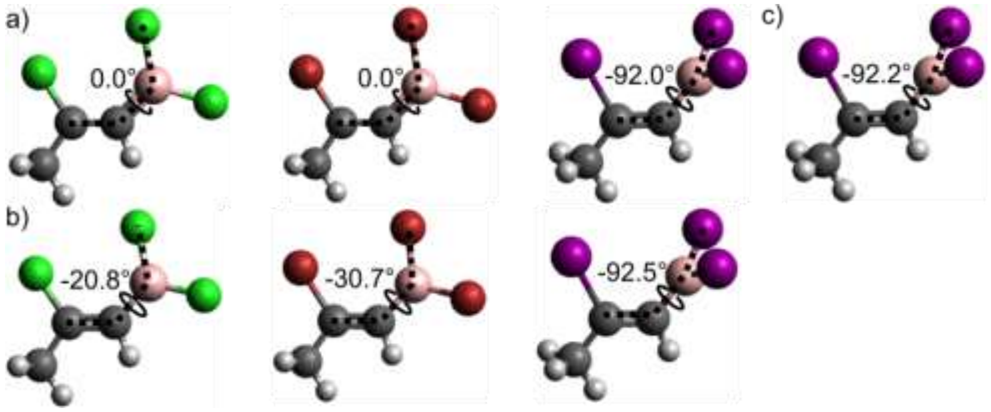


**Figure S7.** The C–C–B–X dihedral angles (indicated by the dotted lines) for products shown in Figure S5. a) B3LYP/6-31+G*_SVP, b) MP2/6-31+G*_SVP and c) B3LYP/ECP46MWB levels.

## B3LYP and MP2 reaction profiles for polar *anti*- and radical additions

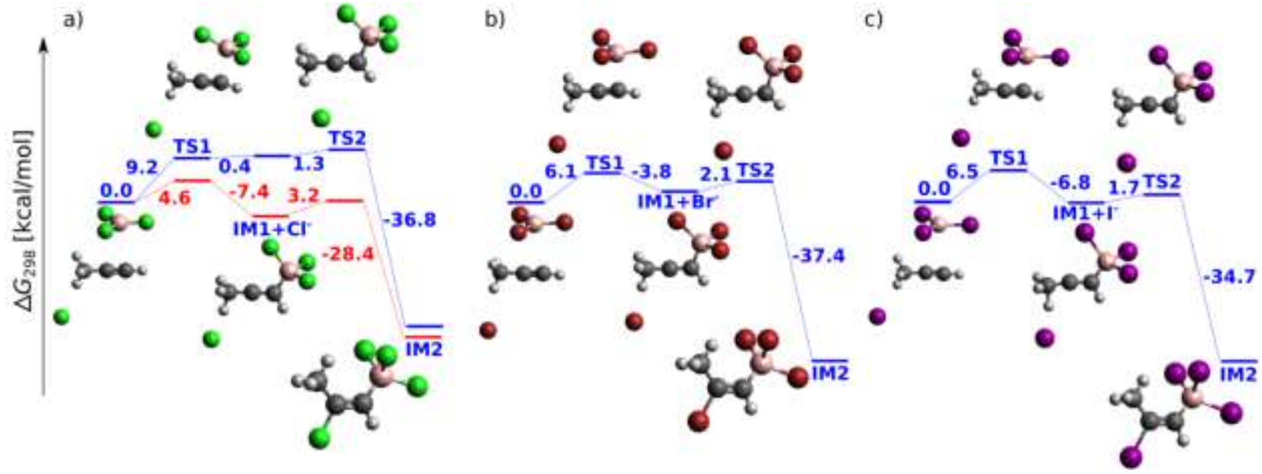


**Figure S8.** Opening of the halide-catalyzed propyne *anti*-haloboration mechanism. Sums of electronic and thermal free energies (in kcal/mol) at MP2/6-31+G*_SVP (blue) and B3LYP/6-31+G* (red) level.

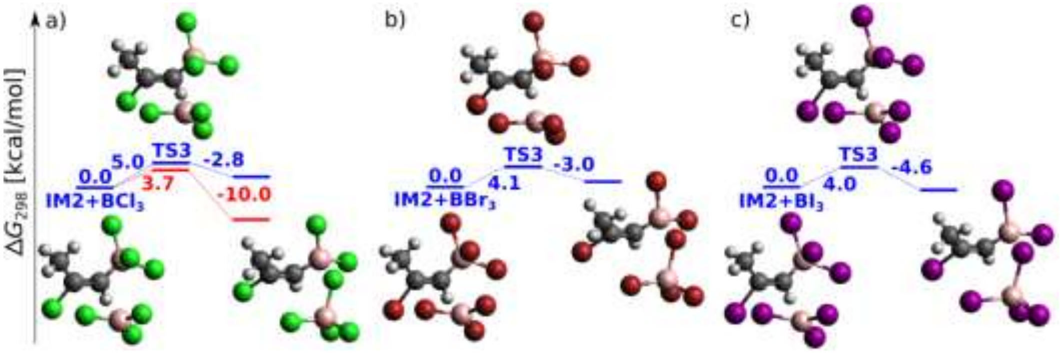


**Figure S9.** Closing of the halide-catalyzed propyne *anti*-haloboration mechanism. Sums of electronic and thermal free energies at MP2/6-31+G*_SVP (blue) and B3LYP/6-31+G* (red) level.

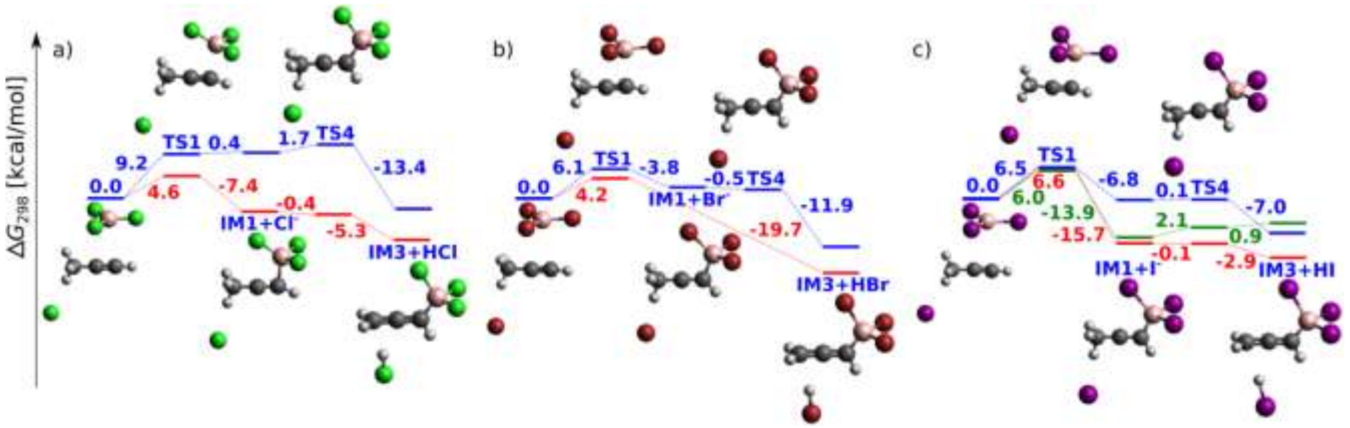


**Figure S10.** Opening of the alternative halide-catalyzed propyne *anti*-haloboration mechanism. Sums of electronic and thermal free energies at MP2/6-31+G*_SVP (blue), B3LYP-D3/6-31+G*_SVP (red), and B3LYP-D3/ECP46MWB (green) levels of theory.

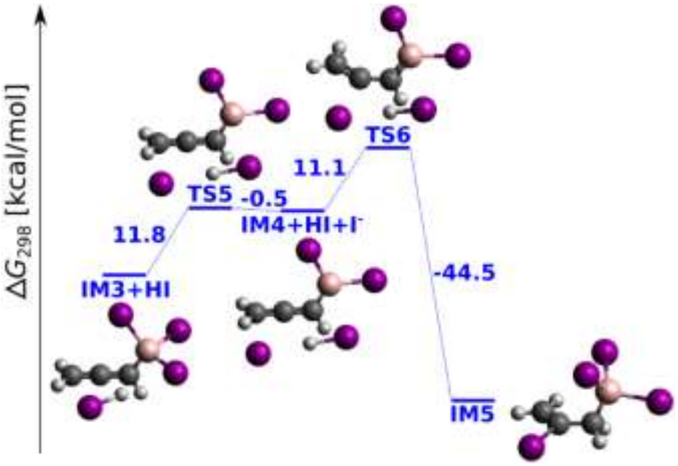


**Figure S11.** MP2/6-31+G*_SVP reaction profile for allenic π-system of IM3 attacked by HI molecule.

A continuation of the reaction profile of propyne iodoboration catalyzed by $I^-$ displayed in the Figures 8 and S11 is shown in Figure S12. The last step of the pathway is a release of $I^-$ from quaternary boron to give an intermediate IM6. Energy barriers for rotation around the C-C bond in IM5 intermediates are shown in the Figure S13. The last step of the reaction mechanism found seems to be endoergonic but we expect that the very last step – formation of *E*-adduct from IM6 (not found yet) could be exoergonic to justify this observation.

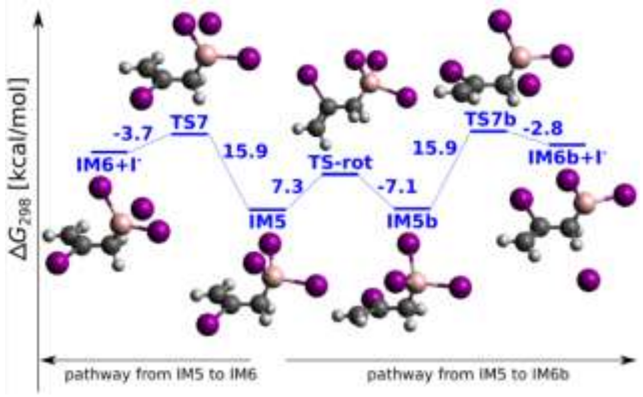


**Figure S12.** Propyne iodoboration catalyzed by $I^-$ at MP2/6-31+G*_SVP level – possible continuations of the reaction profile shown in the Figures 8 and S11.

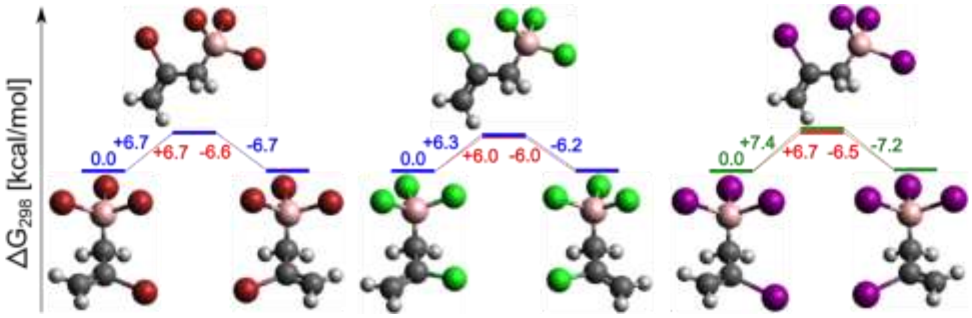


**Figure S13.** Rotation around the C-C bond in IM5 intermediates at the MP2 (blue), B3LYP (red) and at B3LYP/ECP46MWB (green) level – sum of electronic and thermal free energies (in kcal/mol) of local minima and transition structures.

The results shown in Figures 5-8 and S8-S13 for propyne *anti*-haloboration catalyzed by $X^-$ at the MP2 level of theory are summarized in the reaction mechanism shown in Scheme S1.

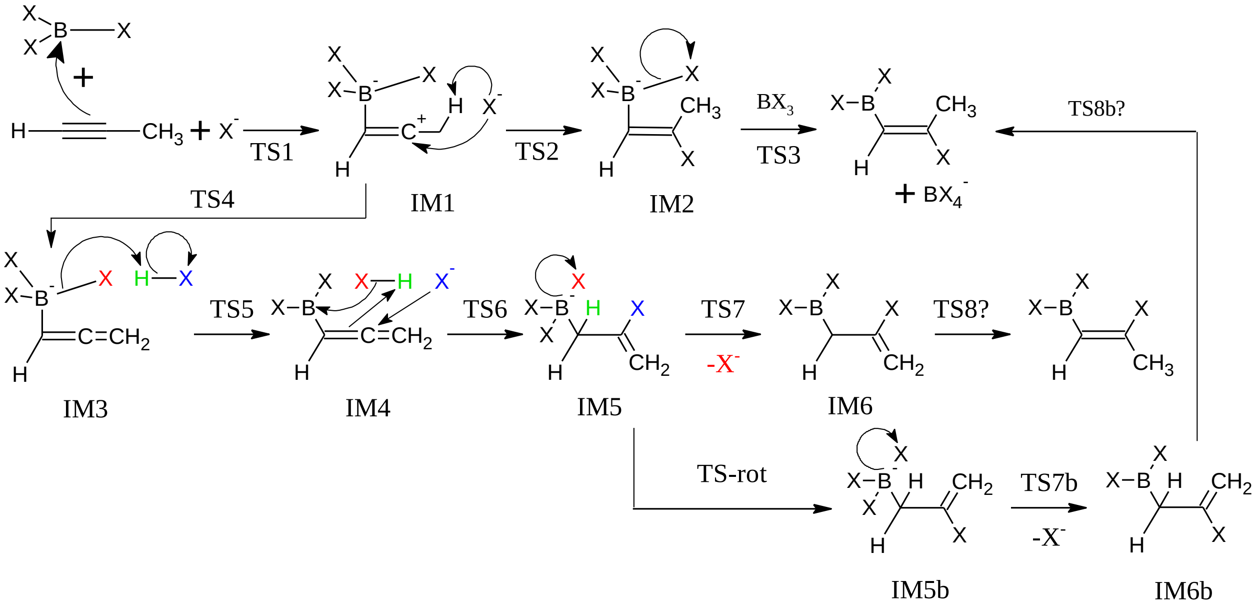


**Scheme S1.** A proposed reaction mechanism of propyne haloboration catalysed by $X^-$

The search for the rest of transition structures TS8(b) and intermediates is still in progress. We expect that the reaction continues as shown in Scheme S2. The reaction mechanisms shown in Scheme S1 and Scheme S2 could explain the observed fact that with a general terminal alkyne, haloboration leads to a *Z*-adduct at low temperature and to a mixture of *Z*- and *E*-adduct at high temperature.

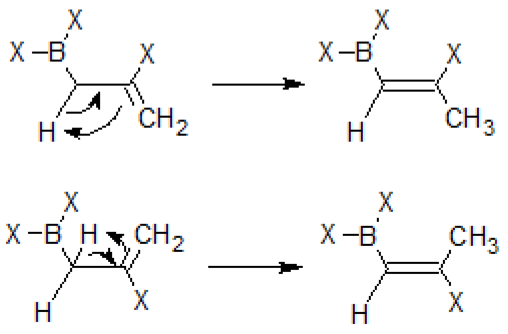


**Scheme S2.** A proposed termination of propyne haloboration catalyzed by X⁻

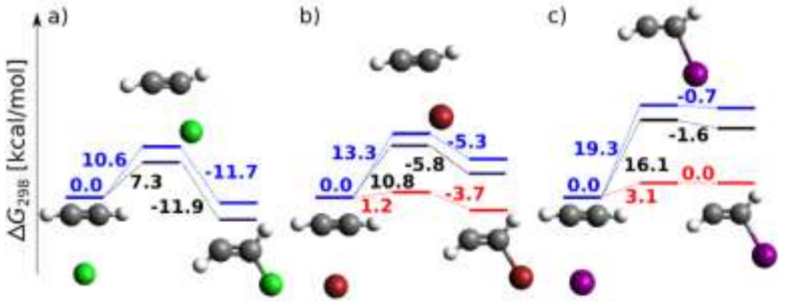


**Figure S14.** Formation of a) (*Z*)-vinylchloride, b) (*Z*)-vinylbromide and c) (*Z*)-vinyliodide radical at at MP2/6-31+G*_SVP (blue), MP2/Def2TZVPP (black) and B3LYP-D3/6-31+G*_SVP (red) level of theory. Sum of electronic and thermal free energies in kcal/mol.

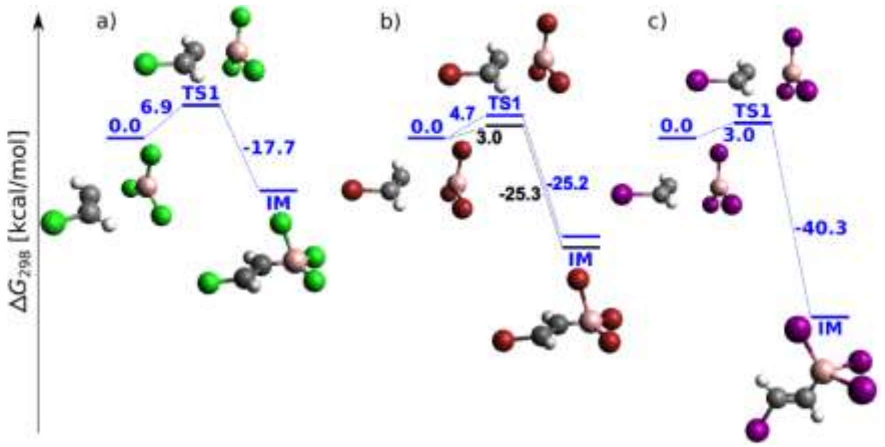


**Figure S15.** (*Z*)-vinylhalide radical attack of $BX_3$: reaction energy profiles at MP2/6-31+G*_SVP (blue) and MP2/Def2TZVPP level (black) for X = Cl, Br and I.

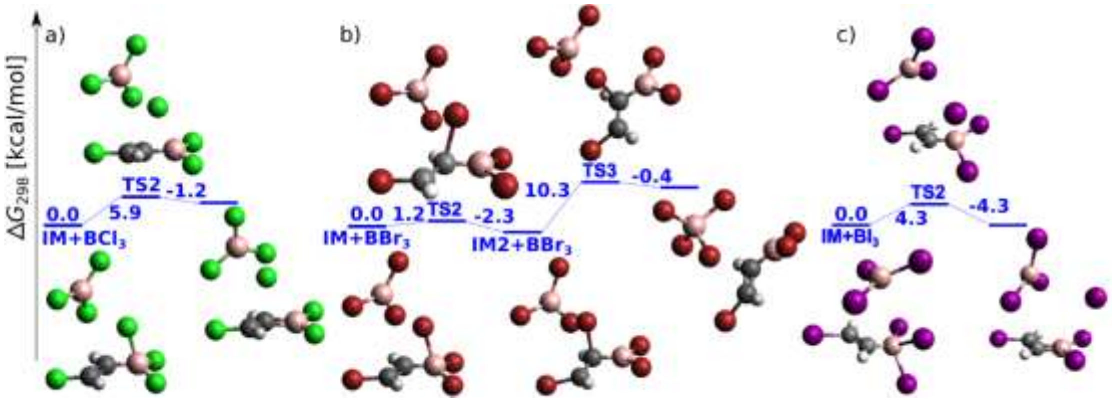


**Figure S16.** *Anti*-haloborated product formation following (*Z*)-vinylhalide radical attack of $BX_3$ at MP2/6-31+G*_SVP level of theory. A sum of electronic and thermal free energies in kcal/mol.

In the Figure S17a, thermal corrections to enthalpy and to Gibbs energy at 298 K (in Hartrees per particle) are 0.065679 and -0.006101 for complex of reactants, 0.063397 and -0.009302 for TS and 0.132384 and 0.056699 for complex of products.

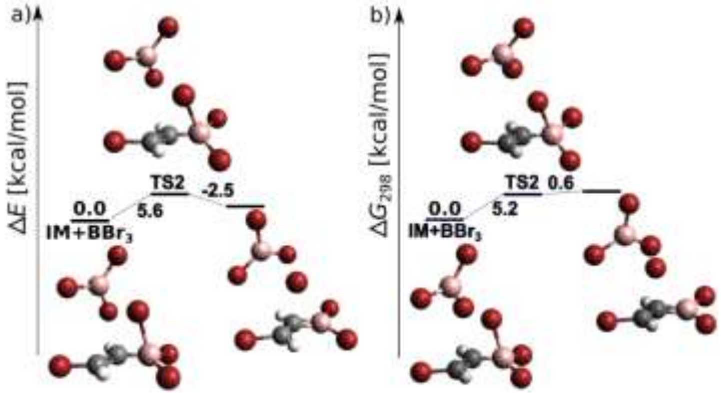


**Figure S17.** *Anti*-bromoborated product formation following (*Z*)-vinylbromide radical attack of $BBr_3$. a) DLPNO-CCSD(T)/cc-pV5Z//MP2/6-31+G*_SVP level of theory, electronic free energies in kcal/mol and b) MP2/Def2TZVPP level of theory, a sum of electronic and thermal free energies in kcal/mol.

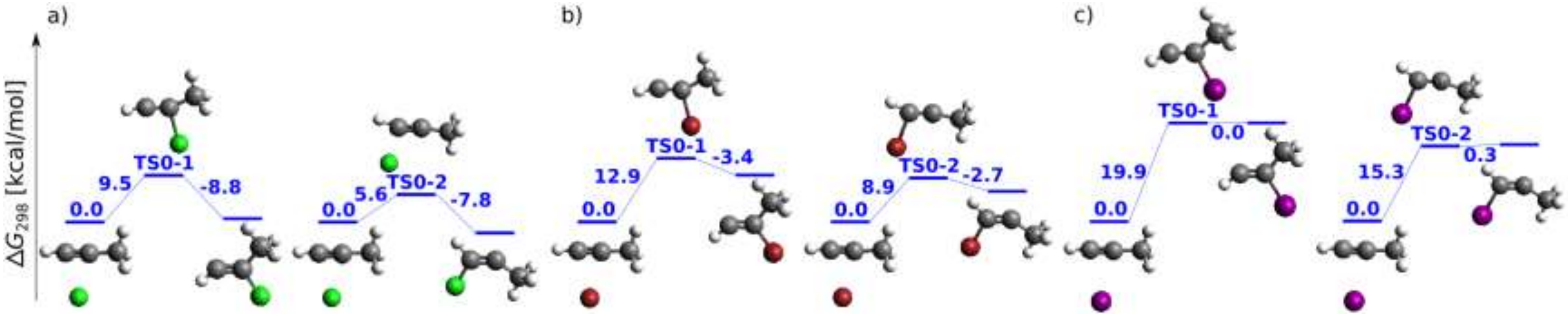


**Figure S18.** Comparison of a) (*Z*)-2-chloropropene-1-yl and (*Z*)-1-chloropropene-2-yl radical formation, b) (*Z*)-2-bromopropene-1-yl radical and (*Z*)-1-bromopropene-2-yl radical formation and c) (*Z*)-2-iodopropene-1-yl and (*Z*)-1-iodopropene-2-yl radical formation at MP2/6-31+G*_SVP level of theory. Sum of electronic and thermal free energies in kcal/mol.

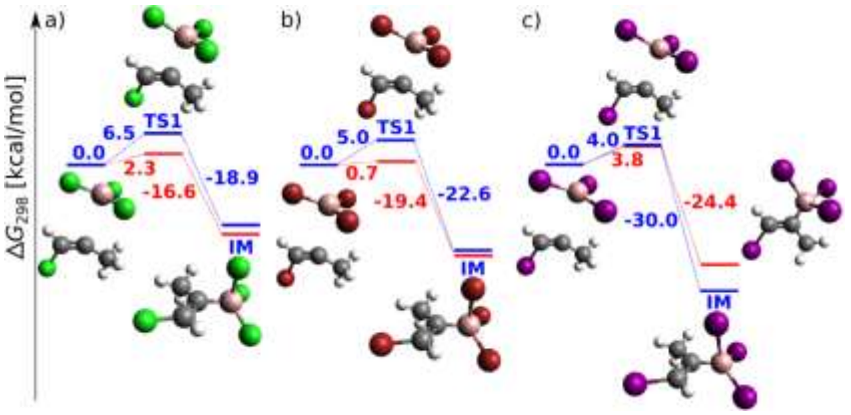


**Figure S19.** Halopropene radical addition on $BX_3$ at MP2/6-31+G*_SVP (blue) and B3LYP-D3/6-31+G*_SVP (red) level of theory. Sum of electronic and thermal free energies in kcal/mol.

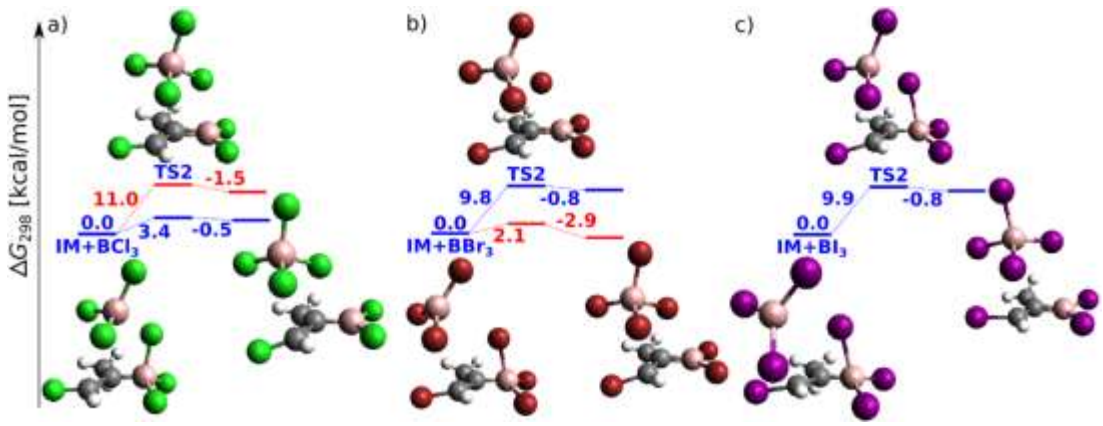


**Figure S20.** Excessive halogen splitting following halopropene radical addition on $BX_3$ at MP2/6-31+G*_SVP (blue) and B3LYP-D3/6-31+G*_SVP (red) level. Sum of electronic and thermal free energies in kcal/mol.

For the particular case of propyne radical iodoboration, the second step displayed in Figures 15 and S20 is analyzed at B3LYP-D3 level in the separate Figure S21. The reason for doing so is in a slightly modified reaction mechanism as predicted by the DFT approach: The bond between the bridging iodine and boron splits first, leaving an *E*-adduct. Only then, iodine radical binds to $BI_3$ molecule, forming the $BI_4$ radical. The complete mechanism is summarized in Scheme S3. All steps are exergonic and energy barriers are lower than at MP2 level.

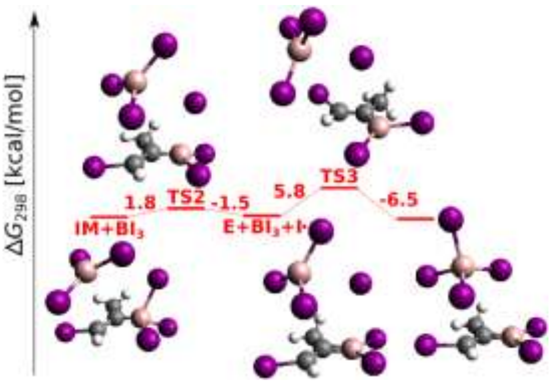


**Figure S21.** Excessive iodine splitting following iodopropene radical addition on $BI_3$ at B3LYP-D3/6-31+G*_SVP level. Sum of electronic and thermal free energies in kcal/mol.

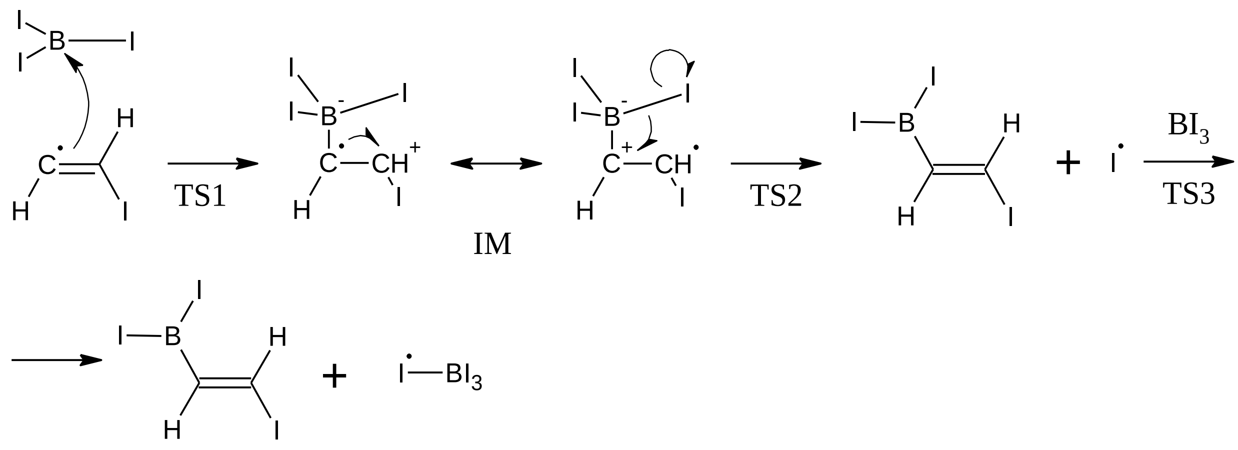


**Scheme S3.** Reaction mechanism of (*Z*)-1-iodopropene-2-yl radical iodoboration at B3LYP-D3/6-31+G*_SVP level of theory.

## Absolute energy contributions referring to Figures

**Table S1.** Electronic energies, thermal corrections to enthalpy and to Gibbs energy at 298 K (in Hartrees per particle) and imaginary frequencies (in $cm^{-1}$).

| Geometry | E(elec) | H(corr) | G(corr) | Imaginary [a] frequency |
|---|---|---|---|---|
| **Figure 1** | | | | |
| $C_2H_2$+$BCl_3$ | -1481.435285 | 0.044303 | -0.004651 | - |
| TS-Cl | -1481.403193 | 0.044297 | 0.005092 | -438.4 |
| Z-Cl | -1481.459223 | 0.048650 | 0.007412 | - |
| $C_2H_2$+$BBr_3$ | -7820.718461 | 0.043127 | -0.009069 | - |
| TS-Br | -7820.695477 | 0.043146 | -0.000104 | -357.7 |
| Z-Br | -7820.747290 | 0.047249 | 0.002249 | - |
| $C_2H_2$+$BI_3$ | -987.160460 | 0.042426 | -0.013290 | - |
| TS1 | -987.146016 | 0.042201 | -0.006030 | -212.6 |
| IM | -987.146841 | 0.043426 | -0.004883 | - |
| TS2 | -987.144101 | 0.042524 | -0.003663 | -309.0 |
| Z-I | -987.194675 | 0.045915 | -0.003025 | - |
| **Figure 2** | | | | |
| $C_2H_2$+$BCl_3$+$Cl^-$ | -1941.373657 | 0.047704 | -0.005959 | - |
| TS2-Cl | -1941.356130 | 0.047099 | -0.001046 | -74.1 |
| IM2-Cl | -1941.431474 | 0.051002 | 0.006339 | - |
| $C_2H_2$+$BBr_3$+$Br^-$ | -10393.763987 | 0.046209 | -0.014659 | - |
| TS1-Br | -10393.751954 | 0.045293 | -0.009004 | -194.1 |
| IM1-Br | -10393.752223 | 0.045477 | -0.005656 | *-10.5* |
| TS2-Br | -10393.750590 | 0.045818 | -0.006100 | -74.0 |
| IM2-Br | -10393.825710 | 0.049729 | -0.000085 | - |
| $C_2H_2$+$BI_3$+$I^-$ | -1282.363030 | 0.045471 | -0.018529 | - |
| TS1-I | -1282.350693 | 0.044769 | -0.013739 | -238.7 |
| IM1-I | -1282.353189 | 0.046143 | -0.011688 | - |
| TS2-I | -1282.352832 | 0.045215 | -0.010021 | -44.4 |

| Geometry | E(elec) | H(corr) | G(corr) | Imaginary [a] frequency |
|---|---|---|---|---|
| IM2-I | -1282.427609 | 0.048949 | -0.004664 | - |
| **Figure 3** | | | | |
| IM2+$BCl_3$ | -3345.649625 | 0.065964 | 0.001446 | - |
| TS3-Cl | -3345.645793 | 0.064626 | 0.004404 | -191.7 |
| E+$BCl_4^-$ | -3345.649833 | 0.065581 | 0.005469 | - |
| IM2+$BBr_3$ | -18137.328033 | 0.062567 | -0.007108 | *-8.9* |
| TS3-Br | -18137.323930 | 0.062162 | -0.007395 | -143.6 |
| E+$BBr_4^-$ | -18137.330475 | 0.063177 | -0.006147 | - |
| IM2+$BI_3$ | -2192.374268 | 0.062067 | -0.017663 | - |
| TS3-I | -2192.371371 | 0.060829 | -0.014897 | -148.0 |
| E+$BI_4^-$ | -2192.380727 | 0.061844 | -0.014023 | - |
| **Figure 4** | | | | |
| $C_3H_4$+$BCl_3$ | -1520.699350 | 0.075548 | 0.023834 | - |
| TS2-Cl | -1520.679490 | 0.074735 | 0.031913 | -354.8 |
| Z-Cl | -1520.722564 | 0.078523 | 0.034123 | - |
| $C_3H_4$+$BBr_3$ | -7859.982863 | 0.074334 | 0.018679 | - |
| TS1-Br | -7859.973472 | 0.073858 | 0.023883 | -201.0 |
| IM-Br | -7859.976334 | 0.074793 | 0.024966 | - |
| TS2-Br | -7859.972685 | 0.073655 | 0.026606 | -278.2 |
| Z-Br | -7860.010528 | 0.077221 | 0.028296 | - |
| $C_3H_4$+$BI_3$ | -1026.425241 | 0.073689 | 0.015233 | - |
| TS-I | -1026.415647 | 0.073263 | 0.018794 | -231.0 |
| IM-I | -1026.422818 | 0.074049 | 0.021853 | - |
| TS2-I | -1026.421275 | 0.072989 | 0.022907 | -207.8 |
| Z-I | -1026.457332 | 0.075994 | 0.024133 | - |
| **Figure 5** | | | | |
| $C_3H_4$+$BCl_3$+$Cl^-$ | -1980.635135 | 0.078300 | 0.018012 | - |
| TS1-Cl | -1980.626432 | 0.077932 | 0.024070 | -171.6 |

| Geometry | E(elec) | H(corr) | G(corr) | Imaginary [a] frequency |
|---|---|---|---|---|
| IM1+$Cl^-$ | -1980.628119 | 0.078701 | 0.024870 | - |
| TS2-Cl | -1980.629715 | 0.077576 | 0.025875 | -60.3 |
| IM2-Cl | -1980.689096 | 0.080766 | 0.032188 | - |
| $C_3H_4$+$BBr_3$+$Br^-$ | -10433.026011 | 0.077306 | 0.012894 | - |
| TS1-Br | -10433.018258 | 0.076617 | 0.017146 | -209.5 |
| IM1+$Br^-$ | -10433.026419 | 0.076174 | 0.017131 | - |
| TS2-Br | -10433.026397 | 0.075777 | 0.020278 | -23.7 |
| IM2-Br | -10433.082976 | 0.079641 | 0.026465 | - |
| $C_3H_4$+$BI_3$+$I^-$ | -1321.625723 | 0.076581 | 0.009381 | - |
| TS1-I | -1321.617845 | 0.075961 | 0.012956 | -218.2 |
| IM1+$I^-$ | -1321.629238 | 0.075722 | 0.013329 | - |
| TS2-I | -1321.629376 | 0.075000 | 0.015944 | -13.1 |
| IM2-I | -1321.683788 | 0.078922 | 0.022016 | - |
| **Figure 6** | | | | |
| IM2+$BCl_3$ | -3384.909058 | 0.095820 | 0.030230 | - |
| TS3 | -3384.905646 | 0.094503 | 0.032789 | -197.7 |
| E+$BCl_4^-$ | -3384.910196 | 0.095431 | 0.032450 | - |
| IM2+$BBr_3$ | -18176.588109 | 0.093452 | 0.018522 | - |
| TS3 | -18176.584939 | 0.092248 | 0.021463 | -142.9 |
| E+$BBr_4^-$ | -18176.591243 | 0.093173 | 0.021839 | - |
| IM2+$BI_3$ | -2231.634372 | 0.092054 | 0.010920 | - |
| TS3 | -2231.632239 | 0.090875 | 0.013469 | -142.5 |
| E+$BI_4^-$ | -2231.641607 | 0.091866 | 0.014141 | - |
| **Figure 7** | | | | |
| IM1+$Cl^-$ | -1980.628110 | 0.078695 | 0.024780 | - |
| TS4-Cl | -1980.627612 | 0.072549 | 0.021655 | -648.8 |
| IM3+HCl | -1980.649676 | 0.075669 | 0.021413 | - |
| IM1+$Br^-$ | -10433.040009 | 0.076151 | 0.017359 | - |

| Geometry | E(elec) | H(corr) | G(corr) | Imaginary frequency [a] |
|---|---|---|---|---|
| TS4-Br | -10433.024619 | 0.072590 | 0.016111 | -204.6 |
| IM3+HBr | -10433.040757 | 0.073939 | 0.015016 | - |
| IM1+I$^-$ | -1321.629172 | 0.075731 | 0.013023 | - |
| TS4-I | -1321.624768 | 0.070588 | 0.010688 | -618.3 |
| IM3+HI | -1321.638274 | 0.072621 | 0.009777 | - |
| **Figure 8** | | | | |
| IM3+HI | -1321.639880 | 0.072511 | 0.010489 | - |
| TS5 | -1321.621920 | 0.068816 | 0.007642 | -508.6 |
| IM4+HI+I$^-$ | -1321.624001 | 0.071954 | 0.007867 | - |
| TS6 | -1321.601891 | 0.071232 | 0.009197 | -203.9 |
| IM5 | -1321.680943 | 0.078541 | 0.022068 | - |
| **Figure 9** | | | | |
| $C_2H_2$+Cl· | -536.925867 | 0.032856 | -0.000402 | - |
| TS0-Cl | -536.928574 | 0.034299 | 0.003335 | -459.8 |
| $C_2H_2$Cl· | -536.947008 | 0.035387 | 0.005016 | - |
| $C_2H_2$+Br· | -2650.042401 | 0.032829 | -0.001579 | - |
| TS0-Br | -2650.041332 | 0.033772 | 0.001685 | -461.6 |
| $C_2H_2$Br· | -2650.049229 | 0.034843 | 0.003104 | - |
| $C_2H_2$+I· | -372.214116 | 0.032468 | -0.003374 | - |
| TS0-I | -372.206504 | 0.033060 | 0.000257 | -456.3 |
| $C_2H_2$I· | -372.207435 | 0.034385 | 0.001641 | - |
| **Figure 10** | | | | |
| $C_2H_2$Cl·+$BCl_3$ | -1941.162706 | 0.050396 | -0.003991 | - |
| TS1-Cl | -1941.159178 | 0.049974 | 0.002815 | -104.3 |
| IM-Cl | -1941.185200 | 0.051286 | 0.006366 | - |
| $C_2H_2$Br·+$BBr_3$ | -10393.546394 | 0.048728 | -0.009743 | - |
| TS1-Br | -10393.544391 | 0.048132 | -0.004146 | -112.3 |
| IM-Br | -10393.581736 | 0.049991 | -0.000192 | - |

| Geometry | E(elec) | H(corr) | G(corr) | Imaginary [a] frequency |
|---|---|---|---|---|
| $C_2H_2I\cdot+BI_3$ | -1282.147163 | 0.047681 | -0.013894 | - |
| TS1-I | -1282.146555 | 0.047014 | -0.009527 | -64.9 |
| IM-I | -1282.202682 | 0.049900 | -0.004264 | - |
| **Figure 12** | | | | |
| $IM+BCl_3$ | -3345.402811 | 0.066396 | 0.001429 | - |
| TS2-Cl | -3345.393348 | 0.066274 | 0.003173 | -211.9 |
| $E+BCl_3+Cl\cdot$ | -3345.393582 | 0.071054 | 0.004791 | - |
| $IM+BBr_3$ | -18137.081633 | 0.063855 | -0.010574 | - |
| TS2-Br | -18137.081203 | 0.062546 | -0.010543 | -151.7 |
| $IM2+BBr_3$ | -18137.080980 | 0.063680 | -0.013108 | - |
| TS3 | -18137.072667 | 0.062492 | -0.008397 | -164.6 |
| $E+BBr_4\cdot$ | -18137.069433 | 0.065777 | -0.005163 | - |
| $IM+BI_3$ | -2192.149293 | 0.062665 | -0.017352 | - |
| TS2-I | -2192.143159 | 0.061850 | -0.017106 | -114.3 |
| $E+BI_3+I\cdot$ | -2192.141777 | 0.062554 | -0.021565 | - |
| **Figure 13** | | | | |
| $C_3H_4+Cl\cdot$ | -576.194015 | 0.064134 | 0.027086 | - |
| TS0-1-Cl | -576.194569 | 0.064491 | 0.029917 | -437.6 |
| $C_3H_4Cl$ (1) | -576.208883 | 0.065519 | 0.031638 | - |
| TS0-2-Cl | -576.197629 | 0.064661 | 0.028198 | -317.9 |
| $C_3H_4Cl$ (2) | -576.212975 | 0.066176 | 0.031267 | - |
| $C_3H_4+Br\cdot$ | -2689.308759 | 0.063967 | 0.025272 | - |
| TS0-1-Br | -2689.305535 | 0.064202 | 0.028614 | -424.4 |
| $C_3H_4Br\cdot$ (1) | -2689.310982 | 0.065162 | 0.029940 | - |
| TS0-2-Br | -2689.310394 | 0.064317 | 0.027034 | -365.4 |
| $C_3H_4Br$ (2) | -2689.315792 | 0.065622 | 0.029281 | - |
| $C_3H_4+I\cdot$ | -411.479334 | 0.063677 | 0.023469 | - |
| TS0-1-I | -411.468854 | 0.063644 | 0.027397 | -416.8 |

| Geometry | E(elec) | H(corr) | G(corr) | Imaginary [a] frequency |
|---|---|---|---|---|
| $C_3H_4I$ (1) | -411.468874 | 0.064805 | 0.028496 | - |
| TS0-2-I | -411.474901 | 0.063801 | 0.026425 | -340.1 |
| $C_3H_4I$ (2) | -411.474832 | 0.065067 | 0.027636 | - |
| **Figure 14** | | | | |
| $C_3H_4Cl{\cdot}{+}BCl_3$ | -1980.430058 | 0.081226 | 0.025746 | - |
| TS1-Cl | -1980.425860 | 0.080639 | 0.031357 | -190.9 |
| IM-Cl | -1980.451690 | 0.080994 | 0.031267 | - |
| $C_3H_4Br{\cdot}{+}BBr_3$ | -10432.814442 | 0.079463 | 0.018286 | - |
| TS1-Br | -10432.812019 | 0.078843 | 0.023704 | -110.7 |
| IM-Br | -10432.844386 | 0.079752 | 0.026532 | - |
| $C_3H_4I{\cdot}{+}BI_3$ | -1321.416229 | 0.078305 | 0.013816 | - |
| TS1-I | -1321.414533 | 0.077726 | 0.018323 | -118.7 |
| IM-I | -1321.458363 | 0.079204 | 0.022603 | - |
| **Figure 15** | | | | |
| $IM{+}BCl_3$ | -3384.661666 | 0.095472 | 0.032719 | - |
| TS2-Cl | -3384.656695 | 0.094620 | 0.032210 | -59.5 |
| $E{+}BCl_4{\cdot}$ | -3384.656265 | 0.096203 | 0.032055 | - |
| $IM{+}BBr_3$ | -18176.344980 | 0.093536 | 0.016767 | - |
| TS2-Br | -18176.332919 | 0.092842 | 0.019993 | -190.3 |
| $E{+}BBr_4{\cdot}$ | -18176.334535 | 0.093957 | 0.020792 | - |
| $IM{+}BI_3$ | -2231.403455 | 0.092292 | 0.008805 | - |
| TS2 | -2231.393826 | 0.091152 | 0.012481 | -190.8 |
| $E{+}BI_4{\cdot}$ | -2231.397133 | 0.093386 | 0.014288 | - |
| **Figure S1** | | | | |
| $C_2H_2{+}BCl_3$, MP2/6-31+G* | -1480.811189 | 0.044303 | -0.004651 | - |
| $C_2H_2{+}BCl_3$, MP2/tzvpp[a] | -1481.181408 | 0.043803 | -0.000827 | *-4.5* |
| $C_2H_2{+}BCl_3$, MP2/tzvpp[b] | -1481.181407 | 0.043803 | -0.000840 | *-8.0* |
| $C_2H_2{+}BCl_3$, B3LYP | -1482.918421 | 0.044849 | -0.003589 | - |

| Geometry | E(elec) | H(corr) | G(corr) | Imaginary frequency [a] |
|---|---|---|---|---|
| TS-Cl, MP2/6-31+G* | -1480.770126 | 0.044297 | 0.005092 | -438.4 |
| TS-Cl, MP2/tzvpp | -1481.142647 | 0.044039 | 0.004787 | -459.3 |
| TS-Cl, B3LYP | -1482.895188 | 0.043570 | 0.004000 | -247.1 |
| Z-Cl, MP2/6-31+G* | -1480.832997 | 0.048650 | 0.007412 | - |
| Z-Cl, MP2/tzvpp | -1481.203859 | 0.048334 | 0.007225 | - |
| Z-Cl, B3LYP | -1482.952983 | 0.048052 | 0.006710 | - |
| $C_2H_2$+$BBr_3$, MP2/SVP | -7818.946807 | 0.043127 | -0.009069 | - |
| $C_2H_2$+$BBr_3$, MP2/tzvpp | -7820.135493 | 0.043706 | -0.007694 | - |
| $C_2H_2$+$BBr_3$, B3LYP | -7823.922715 | 0.043886 | -0.007397 | - |
| TS-Br, MP2/SVP | -7818.917416 | 0.043146 | -0.000104 | -357.7 |
| TS-Br, MP2/tzvpp | -7820.106551 | 0.043086 | -0.000089 | -387.6 |
| TS-Br, B3LYP | -7823.906527 | 0.042633 | -0.001102 | -129.6 |
| Z-Br, MP2/SVP | -7818.974112 | 0.047249 | 0.002249 | - |
| Z-Br, MP2/tzvpp | -7820.161193 | 0.045722 | 0.003575 | *-6.1* |
| Z-Br, B3LYP | -7823.961787 | 0.046923 | 0.000619 | - |
| $C_2H_2$+$BI_3$, MP2/SVP | -20853.864447 | 0.042426 | -0.013290 | - |
| $C_2H_2$+$BI_3$, MP2/tzvpp | -993.508235 | 0.043060 | -0.011075 | - |
| $C_2H_2$+$BI_3$, B3LYP/SVP | -20861.918919 | 0.043318 | -0.010517 | - |
| $C_2H_2$+$BI_3$, B3LYP/ECP46MWB | -136.526645 | 0.043191 | -0.011016 | - |
| TS1, MP2/SVP | -20853.849907 | 0.042201 | -0.006030 | -212.6 |
| TS1, MP2/tzvpp | -993.494572 | 0.042196 | -0.005571 | -105.4 |
| TS1, B3LYP/SVP | -20861.905365 | 0.042210 | -0.006071 | -246.2 |
| TS1, B3LYP/ECP46MWB | -136.513949 | 0.042251 | -0.005923 | -235.9 |
| IM, MP2/SVP | -20853.850747 | 0.043426 | -0.004883 | - |
| IM, MP2/tzvpp | -993.494580 | 0.043190 | -0.006018 | - |
| TS2, MP2/SVP | -20853.844996 | 0.042524 | -0.003663 | -309.0 |
| TS2, MP2/tzvpp | -993.487796 | 0.042465 | -0.003588 | -337.2 |
| Z-I, MP2/SVP | -20853.898957 | 0.045915 | -0.003025 | - |
| Z-I, MP2/tzvpp | -993.541116 | 0.045708 | -0.002246 | - |

| Geometry | E(elec) | H(corr) | G(corr) | Imaginary [a] frequency |
|---|---|---|---|---|
| Z-I, B3LYP/SVP | -20861.963813 | 0.044635 | -0.000200 | *-2.5* |
| Z-I, B3LYP/ECP46MWB | -136.570140 | 0.045546 | -0.003365 | - |
| $C_2H_2$+$BCl_3$, CCSD(T)//MP2 | -1481.435285 | 0.044303 | -0.004651 | - |
| TS-Cl, CCSD(T)//MP2 | -1481.403193 | 0.044297 | 0.005092 | -438.4 |
| Z-Cl, CCSD(T)//MP2 | -1481.459223 | 0.048650 | 0.007412 | - |
| $C_2H_2$+$BBr_3$, CCSD(T)//MP2 | -7820.718461 | 0.043127 | -0.009069 | - |
| TS-Br, CCSD(T)//MP2 | -7820.695477 | 0.043146 | -0.000104 | -357.7 |
| Z-Br, CCSD(T)//MP2 | -7820.747290 | 0.047249 | 0.002249 | - |
| $C_2H_2$+$BI_3$, CCSD(T)//MP2 | -987.160460 | 0.042426 | -0.013290 | - |
| TS1, CCSD(T)//MP2 | -987.146016 | 0.042201 | -0.006030 | -212.6 |
| IM, CCSD(T)//MP2 | -987.146841 | 0.043426 | -0.004883 | - |
| TS2, CCSD(T)//MP2 | -987.144101 | 0.042524 | -0.003663 | -309.0 |
| Z-I, CCSD(T)//MP2 | -987.194675 | 0.045915 | -0.003025 | - |
| **Figure S3** | | | | |
| $C_2H_2$+$BCl_3$+$Cl^-$, MP2 | -1940.587483 | 0.047704 | -0.005959 | - |
| $C_2H_2$+$BCl_3$+$Cl^-$, B3LYP | -1943.296628 | 0.047654 | -0.006155 | - |
| TS1-Cl | -1943.285128 | 0.045905 | -0.003121 | -119.2 |
| IM1-Cl | -1943.285126 | 0.046793 | -0.004014 | - |
| TS2-Cl, MP2 | -1940.566621 | 0.047099 | -0.001046 | -74.1 |
| TS2-Cl, B3LYP | -1943.284340 | 0.045660 | -0.001469 | -56.6 |
| IM2-Cl, MP2 | -1940.645140 | 0.051002 | 0.006339 | - |
| IM2-Cl, B3LYP | -1943.358901 | 0.050187 | 0.005033 | - |
| $C_2H_2$+$BBr_3$+$Br^-$, MP2/SVP | -10391.439930 | 0.046209 | -0.014659 | - |
| $C_2H_2$+$BBr_3$+$Br^-$, MP2/tzvpp | -10393.001052 | 0.046886 | -0.011257 | - |
| TS1-Br | -10391.429659 | 0.045293 | -0.009004 | -194.1 |
| IM1-Br | -10391.430504 | 0.045477 | -0.005656 | *-10.5* |
| TS2-Br, MP2/SVP | -10391.428419 | 0.045818 | -0.006100 | -74.0 |
| TS2-Br, MP2/tzvpp | -10392.986540 | 0.045692 | -0.006444 | -54.1 |
| IM2-Br, MP2/SVP | -10391.510514 | 0.049729 | -0.000085 | - |

| Geometry | E(elec) | H(corr) | G(corr) | Imaginary [a] frequency |
|---|---|---|---|---|
| IM2-Br, MP2/tzvpp | -10393.065721 | 0.049436 | -0.001016 | - |
| $C_2H_2$+$BI_3$+$I^-$, MP2/SVP | -27771.349078 | 0.045471 | -0.018529 | - |
| $C_2H_2$+$BI_3$+$I^-$, MP2/tzvpp | -1290.842457 | 0.045943 | -0.016180 | - |
| TS1-I, MP2/SVP | -27771.338318 | 0.044769 | -0.013739 | -238.7 |
| TS1-I, MP2/tzvpp | -1290.831821 | 0.044870 | -0.013633 | -216.1 |
| IM1-I, MP2/SVP | -27771.342755 | 0.046143 | -0.011688 | - |
| IM1-I, MP2/tzvpp | -1290.833789 | 0.045847 | -0.012747 | - |
| TS2-I, MP2/SVP | -27771.341795 | 0.045215 | -0.010021 | -44.4 |
| TS2-I, MP2/tzvpp | -1290.831461 | 0.045153 | -0.010175 | -33.8 |
| IM2-I, MP2/SVP | -27771.420344 | 0.048949 | -0.004664 | - |
| IM2-I, MP2/tzvpp | -1290.910309 | 0.048624 | -0.005044 | - |
| $C_2H_2$+$BCl_3$+$Cl^-$,CCSD(T)//MP2 | -1941.373657 | 0.047704 | -0.005959 | - |
| TS2-Cl, CCSD(T)//MP2 | -1941.356130 | 0.047099 | -0.001046 | -74.1 |
| IM2-Cl, CCSD(T)//MP2 | -1941.431474 | 0.051002 | 0.006339 | - |
| $C_2H_2$+$BBr_3$+$Br^-$,CCSD(T)//MP2 | -10393.763987 | 0.046209 | -0.014659 | - |
| TS1-Br, CCSD(T)//MP2 | -10393.751954 | 0.045293 | -0.009004 | -194.1 |
| IM1-Br, CCSD(T)//MP2 | -10393.752223 | 0.045477 | -0.005656 | *-10.5* |
| TS2-Br, CCSD(T)//MP2 | -10393.750590 | 0.045818 | -0.006100 | -74.0 |
| IM2-Br, CCSD(T)//MP2 | -10393.825710 | 0.049729 | -0.000085 | - |
| $C_2H_2$+$BI_3$+$I^-$, CCSD(T)//MP2 | -1282.363030 | 0.045471 | -0.018529 | - |
| TS1-I, CCSD(T)//MP2 | -1282.350693 | 0.044769 | -0.013739 | -238.7 |
| IM1-I, CCSD(T)//MP2 | -1282.353189 | 0.046143 | -0.011688 | - |
| TS2-I, CCSD(T)//MP2 | -1282.352832 | 0.045215 | -0.010021 | -44.4 |
| IM2-I, CCSD(T)//MP2 | -1282.427609 | 0.048949 | -0.004664 | - |
| **Figure S4** | | | | |
| IM2+$BCl_3$, MP2/6-31+G* | -3344.382907 | 0.065964 | 0.001446 | - |
| IM2+$BCl_3$, MP2/tzvpp | -3345.129062 | 0.065357 | 0.001077 | - |
| IM2+$BCl_3$, B3LYP | -3348.940781 | 0.064836 | -0.000012 | - |
| TS3-Cl, MP2/6-31+G* | -3344.378319 | 0.064626 | 0.004404 | -191.7 |

| Geometry | E(elec) | H(corr) | G(corr) | Imaginary [a] frequency |
|---|---|---|---|---|
| TS3-Cl, MP2/tzvpp | -3345.126946 | 0.064074 | 0.003539 | -149.6 |
| TS3-Cl, B3LYP | -3348.937590 | 0.063537 | 0.002836 | -144.4 |
| E+$BCl_4^-$, MP2/6-31+G* | -3344.382630 | 0.065581 | 0.005469 | - |
| E+$BCl_4^-$, MP2/tzvpp | -3345.131990 | 0.064964 | 0.004360 | - |
| E+$BCl_4^-$, B3LYP | -3348.949257 | 0.065038 | -0.000181 | - |
| IM2+$BBr_3$, MP2 | -18133.386168 | 0.062567 | -0.007108 | *-8.9* |
| IM2+$BBr_3$, B3LYP | -18144.630670 | 0.062807 | -0.010774 | - |
| TS3-Br, MP2 | -18133.381000 | 0.062162 | -0.007395 | -143.6 |
| TS3-Br, B3LYP | -18144.628408 | 0.061515 | -0.007990 | -109.0 |
| E+$BBr_4^-$, MP2 | -18133.387628 | 0.063177 | -0.006147 | - |
| E+$BBr_4^-$, B3LYP | -18144.636988 | 0.062993 | -0.010468 | - |
| IM2+$BI_3$, MP2 | -48548.214465 | 0.062067 | -0.017663 | - |
| IM2+$BI_3$, B3LYP | -48566.639684 | 0.061519 | -0.019153 | - |
| TS3-I, MP2 | -48548.209211 | 0.060829 | -0.014897 | -148.0 |
| TS3-I, B3LYP | -48566.636391 | 0.060293 | -0.016426 | -104.1 |
| E+$BI_4^-$, MP2 | -48548.218047 | 0.061844 | -0.014023 | - |
| E+$BI_4^-$, B3LYP | -48566.644748 | 0.061651 | -0.018718 | - |
| IM2+$BCl_3$, CCSD(T)//MP2 | -3345.649625 | 0.065964 | 0.001446 | - |
| TS3-Cl, CCSD(T)//MP2 | -3345.645793 | 0.064626 | 0.004404 | -191.7 |
| E+$BCl_4^-$, CCSD(T)//MP2 | -3345.649833 | 0.065581 | 0.005469 | - |
| IM2+$BBr_3$, CCSD(T)//MP2 | -18137.328033 | 0.062567 | -0.007108 | *-8.9* |
| TS3-Br, CCSD(T)//MP2 | -18137.323930 | 0.062162 | -0.007395 | -143.6 |
| E+$BBr_4^-$, CCSD(T)//MP2 | -18137.330475 | 0.063177 | -0.006147 | - |
| IM2+$BI_3$, CCSD(T)//MP2 | -2192.374268 | 0.062067 | -0.017663 | - |
| TS3-I, CCSD(T)//MP2 | -2192.371371 | 0.060829 | -0.014897 | -148.0 |
| E+$BI_4^-$, CCSD(T)//MP2 | -2192.380727 | 0.061844 | -0.014023 | - |
| **Figure S5** | | | | |
| $C_3H_4$+$BCl_3$, MP2 | -1519.988822 | - | - | - |
| TS2-Cl, MP2 | -1519.961506 | - | - | - |

| Geometry | E(elec) | H(corr) | G(corr) | Imaginary [a] frequency |
|---|---|---|---|---|
| Z-Cl, MP2 | -1520.010487 | - | - | - |
| $C_3H_4$+$BCl_3$, CCSD(T)/cc-pVTZ | -1520.551393 | - | - | - |
| TS2-Cl, CCSD(T)/cc-pVTZ | -1520.529556 | - | - | - |
| Z-Cl, CCSD(T)/cc-pVTZ | -1520.573712 | - | - | - |
| $C_3H_4$+$BCl_3$, CCSD(T)/cc-pV5Z | -1520.699350 | - | - | - |
| TS2-Cl, CCSD(T)/cc-pV5Z | -1520.679490 | - | - | - |
| Z-Cl, CCSD(T)/cc-pV5Z | -1520.722564 | - | - | - |
| $C_3H_4$+$BCl_3$, B3LYP | -1522.251559 | - | - | - |
| TS1-Cl | -1522.243410 | - | - | - |
| IM-Cl | -1522.246198 | - | - | - |
| TS2-Cl, B3LYP | -1522.240491 | - | - | - |
| Z-Cl, B3LYP | -1522.281993 | - | - | - |
| $C_3H_4$+$BCl_3$, CCSD(T)//B3LYP | -1520.551772 | - | - | - |
| TS1-Cl, CCSD(T)//B3LYP | -1520.539018 | - | - | - |
| IM-Cl, CCSD(T)//B3LYP | -1520.537509 | - | - | - |
| TS2-Cl, CCSD(T)//B3LYP | -1520.529267 | - | - | - |
| Z-Cl, CCSD(T)//B3LYP | -1520.573971 | - | - | - |
| $C_3H_4$+$BBr_3$, MP2 | -7858.123398 | - | - | - |
| TS1-Br, MP2 | -7858.114556 | - | - | - |
| IM-Br, MP2 | -7858.115449 | - | - | - |
| TS2-Br, MP2 | -7858.107891 | - | - | - |
| Z-Br, MP2 | -7858.150572 | - | - | - |
| $C_3H_4$+$BBr_3$, CCSD(T)/cc-pVTZ | -7859.452197 | - | - | - |
| TS1-Br, CCSD(T)/cc-pVTZ | -7859.441063 | - | - | - |
| IM-Br, CCSD(T)/cc-pVTZ | -7859.442934 | - | - | - |
| TS2-Br, CCSD(T)/cc-pVTZ | -7859.439085 | - | - | - |
| Z-Br, CCSD(T)/cc-pVTZ | -7859.479131 | - | - | - |
| $C_3H_4$+$BBr_3$, CCSD(T)/cc-pV5Z | -7859.982863 | - | - | - |
| TS1-Br, CCSD(T)/cc-pV5Z | -7859.973472 | - | - | - |

| Geometry | E(elec) | H(corr) | G(corr) | Imaginary [a] frequency |
|---|---|---|---|---|
| IM-Br, CCSD(T)/cc-pV5Z | -7859.976334 | - | - | - |
| TS2-Br, CCSD(T)/cc-pV5Z | -7859.972685 | - | - | - |
| Z-Br, CCSD(T)/cc-pV5Z | -7860.010528 | - | - | - |
| $C_3H_4$+$BBr_3$, B3LYP | -7863.255628 | - | - | - |
| TS1-Br, B3LYP | -7863.247596 | - | - | - |
| IM-Br, B3LYP | -7863.253987 | - | - | - |
| TS2-Br, B3LYP | -7863.252195 | - | - | - |
| Z-Br, B3LYP | -7863.290000 | - | - | - |
| $C_3H_4$+$BBr_3$, CCSD(T)//B3LYP | -7859.452313 | - | - | - |
| TS1-Br, CCSD(T)//B3LYP | -7859.441054 | - | - | - |
| IM-Br, CCSD(T)//B3LYP | -7859.442955 | - | - | - |
| TS2-Br, CCSD(T)//B3LYP | -7859.438774 | - | - | - |
| Z-Br, CCSD(T)//B3LYP | -7859.479140 | - | - | - |
| **Figure S6** | | | | |
| $C_3H_4$+$BCl_3$, MP2 | -1519.988822 | 0.075548 | 0.023834 | - |
| $C_3H_4$+$BCl_3$, B3LYP | -1522.251559 | 0.075259 | 0.023135 | - |
| TS1-Cl | -1522.243410 | 0.073876 | 0.027310 | -231.8 |
| IM-Cl | -1522.246198 | 0.074339 | 0.028263 | - |
| TS2-Cl, MP2 | -1519.961506 | 0.074735 | 0.031913 | -354.8 |
| TS2-Cl, B3LYP | -1522.240491 | 0.073802 | 0.030526 | -255.0 |
| Z-Cl, MP2 | -1520.010487 | 0.078523 | 0.034123 | - |
| Z-Cl, B3LYP | -1522.281993 | 0.077419 | 0.032636 | - |
| $C_3H_4$+$BBr_3$, MP2 | -7858.123398 | 0.074334 | 0.018679 | - |
| $C_3H_4$+$BBr_3$, B3LYP | -7863.255628 | 0.074297 | 0.019271 | - |
| TS1-Br, MP2 | -7858.114556 | 0.073858 | 0.023883 | -201.0 |
| TS1-Br, B3LYP | -7863.247596 | 0.072983 | 0.020468 | -250.1 |
| IM-Br, MP2 | -7858.115449 | 0.074793 | 0.024966 | - |
| IM-Br, B3LYP | -7863.253987 | 0.073605 | 0.024068 | - |
| TS2-Br, MP2 | -7858.107891 | 0.073655 | 0.026606 | -278.2 |

| Geometry | E(elec) | H(corr) | G(corr) | Imaginary [a] frequency |
|---|---|---|---|---|
| TS2-Br, B3LYP | -7863.252195 | 0.072861 | 0.025498 | -167.6 |
| Z-Br, MP2 | -7858.150572 | 0.077221 | 0.028296 | - |
| Z-Br, B3LYP | -7863.290000 | 0.075385 | 0.030128 | *-6.5* |
| $C_3H_4$+$BI_3$, MP2 | -20893.041155 | 0.073689 | 0.015233 | - |
| $C_3H_4$+$BI_3$, B3LYP/SVP | -20901.252141 | 0.073686 | 0.015806 | - |
| $C_3H_4$+$BI_3$, B3LYP/ECP46MWB | -175.859620 | 0.073620 | 0.016612 | - |
| TS-I, MP2 | -20893.032828 | 0.073263 | 0.018794 | -231.0 |
| TS-I, B3LYP/SVP | -20901.244352 | 0.072558 | 0.019218 | -252.0 |
| TS-I, B3LYP/ECP46MWB | -175.852583 | 0.072554 | 0.016083 | -247.6 |
| IM-I, MP2 | -20893.037966 | 0.074049 | 0.021853 | - |
| IM-I, B3LYP | -175.8659497 | 0.073102 | 0.020518 | - |
| TS2-I, MP2 | -20893.035497 | 0.072989 | 0.022907 | -207.8 |
| TS2-I, B3LYP | -175.865918 | 0.072169 | 0.021888 | -62.4 |
| Z-I, MP2 | -20893.075549 | 0.075994 | 0.024133 | - |
| Z-I, B3LYP/SVP | -20901.290091 | 0.075187 | 0.022673 | - |
| Z-I, B3LYP/ECP46MWB | -175.897861 | 0.075016 | 0.023043 | - |
| $C_3H_4$+$BCl_3$, CCSD(T)//MP2 | -1520.699350 | 0.075548 | 0.023834 | - |
| TS2-Cl, CCSD(T)//MP2 | -1520.679490 | 0.074735 | 0.031913 | -354.8 |
| Z-Cl, CCSD(T)//MP2 | -1520.722564 | 0.078523 | 0.034123 | - |
| $C_3H_4$+$BBr_3$, CCSD(T)//MP2 | -7859.982863 | 0.074334 | 0.018679 | - |
| TS1-Br, CCSD(T)//MP2 | -7859.973472 | 0.073858 | 0.023883 | -201.0 |
| IM-Br, CCSD(T)//MP2 | -7859.976334 | 0.074793 | 0.024966 | - |
| TS2-Br, CCSD(T)//MP2 | -7859.972685 | 0.073655 | 0.026606 | -278.2 |
| Z-Br, CCSD(T)//MP2 | -7860.010528 | 0.077221 | 0.028296 | - |
| $C_3H_4$+$BI_3$, CCSD(T)//MP2 | -1026.425241 | 0.073689 | 0.015233 | - |
| TS-I, CCSD(T)//MP2 | -1026.415647 | 0.073263 | 0.018794 | -231.0 |
| IM-I, CCSD(T)//MP2 | -1026.422818 | 0.074049 | 0.021853 | - |
| TS2-I, CCSD(T)//MP2 | -1026.421275 | 0.072989 | 0.022907 | -207.8 |

| Geometry | E(elec) | H(corr) | G(corr) | Imaginary [a] frequency |
|---|---|---|---|---|
| Z-I, CCSD(T)//MP2 | -1026.457332 | 0.075994 | 0.024133 | - |
| **Figure S8** | | | | |
| $C_3H_4$+$BCl_3$+$Cl^-$, MP2 | -1979.761288 | 0.078300 | 0.018012 | - |
| $C_3H_4$+$BCl_3$+$Cl^-$,B3LYP | -1982.626524 | 0.078250 | 0.020303 | - |
| TS1-Cl, MP2 | -1979.752674 | 0.077932 | 0.024070 | -171.6 |
| TS1-Cl,B3LYP | -1982.621117 | 0.076783 | 0.022233 | -236.1 |
| IM1+$Cl^-$, MP2 | -1979.752984 | 0.078701 | 0.024870 | - |
| IM1+$Cl^-$,B3LYP | -1982.632252 | 0.075021 | 0.021500 | - |
| TS2-Cl, MP2 | -1979.751856 | 0.077576 | 0.025875 | -60.3 |
| TS2-Cl,B3LYP | -1982.629299 | 0.075604 | 0.023591 | -95.0 |
| IM2-Cl, MP2 | -1979.816737 | 0.080766 | 0.032188 | - |
| IM2-Cl,B3LYP | -1982.681978 | 0.079614 | 0.030996 | - |
| $C_3H_4$+$BBr_3$+$Br^-$ | -10430.612292 | 0.077306 | 0.012894 | - |
| TS1-Br | -10430.606769 | 0.076617 | 0.017146 | -209.5 |
| IM1+$Br^-$ | -10430.613013 | 0.076174 | 0.017131 | - |
| TS2-Br | -10430.612783 | 0.075777 | 0.020278 | -23.7 |
| IM2-Br | -10430.678509 | 0.079641 | 0.026465 | - |
| $C_3H_4$+$BI_3$+$I^-$ | -27810.523542 | 0.076581 | 0.009381 | - |
| TS1-I | -27810.516808 | 0.075961 | 0.012956 | -218.2 |
| IM1+$I^-$ | -27810.528143 | 0.075722 | 0.013329 | - |
| TS2-I | -27810.528092 | 0.075000 | 0.015944 | -13.1 |
| IM2-I | -27810.589461 | 0.078922 | 0.022016 | - |
| **Figure S9** | | | | |
| IM2+$BCl_3$, MP2 | -3383.560195 | 0.095820 | 0.030230 | - |
| IM2+$BCl_3$,B3LYP | -3388.266756 | 0.094372 | 0.027778 | - |
| TS3-Cl, MP2 | -3383.554849 | 0.094503 | 0.032789 | -197.7 |
| TS3-Cl,B3LYP | -3388.263889 | 0.093138 | 0.030729 | -144.6 |
| E+$BCl_4^-$, MP2 | -3383.558903 | 0.095431 | 0.032450 | - |
| E+$BCl_4^-$,B3LYP | -3388.276543 | 0.094509 | 0.027490 | - |

| Geometry | E(elec) | H(corr) | G(corr) | Imaginary [a] frequency |
|---|---|---|---|---|
| IM2+$BBr_3$ | -18172.562633 | 0.093452 | 0.018522 | - |
| TS3-Br | -18172.559118 | 0.092248 | 0.021463 | -142.9 |
| E+$BBr_4^-$ | -18172.564338 | 0.093173 | 0.021839 | - |
| IM2+$BI_3$ | -48587.390507 | 0.092054 | 0.010920 | - |
| TS3-I | -48587.386753 | 0.090875 | 0.013469 | -142.5 |
| E+$BI_4^-$ | -48587.394720 | 0.091866 | 0.014141 | - |
| **Figure S10** | | | | |
| $C_3H_4$+$BCl_3$+$Cl^-$, MP2 | -1979.761288 | 0.078300 | 0.018012 | - |
| $C_3H_4$+$BCl_3$+$Cl^-$,B3LYP | -1982.626524 | 0.078250 | 0.020303 | - |
| TS1-Cl, MP2 | -1979.752674 | 0.077932 | 0.024070 | -171.6 |
| TS1-Cl,B3LYP | -1982.621117 | 0.076783 | 0.022233 | -236.1 |
| IM1+$Cl^-$, MP2 | -1979.752985 | 0.078695 | 0.024780 | - |
| IM1+$Cl^-$,B3LYP | -1982.632252 | 0.075016 | 0.021534 | - |
| TS4-Cl, MP2 | -1979.747106 | 0.072549 | 0.021655 | -648.8 |
| TS4-Cl,B3LYP | -1982.631775 | 0.071475 | 0.020473 | -501.4 |
| IM3+HCl, MP2 | -1979.768163 | 0.075669 | 0.021413 | - |
| IM3+HCl, B3LYP | -1982.640779 | 0.074466 | 0.021011 | - |
| $C_3H_4$+$BBr_3$+$Br^-$, MP2 | -10430.612292 | 0.077306 | 0.012894 | - |
| $C_3H_4$+$BBr_3$+$Br^-$,B3LYP | -10437.294572 | 0.077299 | 0.014750 | - |
| TS1-Br, MP2 | -10430.606769 | 0.076617 | 0.017146 | -209.5 |
| TS1-Br,B3LYP | -10437.288923 | 0.075588 | 0.015781 | -223.7 |
| IM1+$Br^-$ | -10430.613023 | 0.076151 | 0.017359 | - |
| TS4-Br | -10430.612451 | 0.072590 | 0.016111 | -204.6 |
| IM3+HBr, MP2 | -10430.630281 | 0.073939 | 0.015016 | - |
| IM3+HBr,B3LYP | -10437.318606 | 0.072936 | 0.014122 | - |
| $C_3H_4$+$BI_3$+$I^-$, MP2 | -27810.523542 | 0.076581 | 0.009381 | - |
| $C_3H_4$+$BI_3$+$I^-$,B3LYP/ECP46MWB | -187.464180 | 0.076449 | 0.007695 | - |
| $C_3H_4$+$BI_3$+$I^-$, B3LYP/SVP | -27821.308048 | 0.076615 | 0.009892 | - |
| TS1-I, MP2 | -27810.516808 | 0.075961 | 0.012956 | -218.2 |

| Geometry | E(elec) | H(corr) | G(corr) | Imaginary [a] frequency |
|---|---|---|---|---|
| TS1-I, B3LYP/ECP46MWB | -187.458879 | 0.074232 | 0.011984 | -222.1 |
| TS1-I, B3LYP/SVP | -27821.300502 | 0.075220 | 0.012818 | -218.4 |
| IM1+I$^-$, MP2 | -27810.528136 | 0.075731 | 0.013023 | - |
| IM1+I$^-$, B3LYP/ECP46MWB | -187.481534 | 0.074405 | 0.012389 | - |
| IM1+I$^-$, B3LYP/SVP | -27821.324538 | 0.073707 | 0.011798 | - |
| TS4-I, MP2 | -27810.525680 | 0.070588 | 0.010688 | -618.3 |
| TS4-I, B3LYP/ECP46MWB | -187.475360 | 0.069717 | 0.009519 | -186.2 |
| TS4-I, B3LYP/SVP | -27821.322176 | 0.069587 | 0.009252 | -781.7 |
| IM3+HI, MP2 | -27810.535888 | 0.072621 | 0.009777 | - |
| IM3+HI, B3LYP/ECP46MWB | -187.475497 | 0.070379 | 0.011169 | *-9.3* |
| IM3+HI, B3LYP/SVP | -27821.325994 | 0.071955 | 0.008413 | - |
| **Figure S11** | | | | |
| IM3+HI | -27810.537637 | 0.072511 | 0.010489 | - |
| TS5 | -27810.516012 | 0.068816 | 0.007642 | -508.6 |
| IM4+HI+I$^-$ | -27810.517033 | 0.071954 | 0.007867 | - |
| TS6 | -27810.500730 | 0.071232 | 0.009197 | -203.9 |
| IM5 | -27810.584448 | 0.078541 | 0.022068 | - |
| **Figure S12** | | | | |
| IM5 | -27810.584447 | 0.078540 | 0.022094 | - |
| TS7 | -27810.558223 | 0.077504 | 0.021138 | -57.8 |
| IM6+I$^-$ | -27810.559917 | 0.078474 | 0.016953 | - |
| TS-rot | -27810.574610 | 0.077461 | 0.023936 | -65.7 |
| IM5b | -27810.584447 | 0.078540 | 0.022098 | - |
| TS7b | -27810.558223 | 0.077505 | 0.021138 | -57.8 |
| IM6b+I$^-$ | -27810.559942 | 0.078506 | 0.018410 | - |
| **Figure S13** | | | | |
| IM5-Cl, MP2 | -1979.813388 | 0.080550 | 0.032889 | - |
| IM5-Cl, B3LYP | -1982.677691 | 0.079365 | 0.031539 | - |

| Geometry | E(elec) | H(corr) | G(corr) | Imaginary frequency [a] |
|---|---|---|---|---|
| TS-rot-Cl, MP2 | -1979.804926 | 0.079328 | 0.034485 | -84.5 |
| TS-rot-Cl, B3LYP | -1982.669498 | 0.078189 | 0.032950 | -79.8 |
| IM5b-Cl, MP2 | -1979.813387 | 0.080552 | 0.033013 | - |
| IM5b-Cl, B3LYP | -1982.677691 | 0.079365 | 0.031542 | - |
| IM5-Br, MP2 | -10430.677090 | 0.079277 | 0.027088 | - |
| IM5-Br, B3LYP | -10437.359376 | 0.078385 | 0.025355 | - |
| TS-rot-Br, MP2 | -10430.667597 | 0.078195 | 0.028292 | -69.3 |
| TS-rot-Br, B3LYP | -10437.350304 | 0.077207 | 0.026977 | -69.7 |
| IM5b-Br, MP2 | -10430.677090 | 0.079278 | 0.027089 | - |
| IM5b-Br, B3LYP | -10437.359364 | 0.078367 | 0.025511 | - |
| IM5-I, B3LYP/ECP46MWB | -187.516372 | 0.077626 | 0.019896 | - |
| IM5-I, B3LYP/SVP | -27821.368020 | 0.077765 | 0.021056 | - |
| TS-rot-I, B3LYP/ECP46MWB | -187.507229 | 0.076500 | 0.022609 | -65.4 |
| TS-rot-I, B3LYP/SVP | -27821.358887 | 0.076599 | 0.022651 | -64.0 |
| IM5b-I, B3LYP/ECP46MWB | -187.516356 | 0.077620 | 0.020256 | - |
| IM5b-I, B3LYP/SVP | -27821.368026 | 0.077802 | 0.021445 | - |
| **Figure S14** | | | | |
| $C_2H_2$+Cl·, MP2/6-31+G* | -536.635009 | 0.032856 | -0.000402 | - |
| $C_2H_2$+Cl·, MP2/tzvpp | -536.805949 | 0.033678 | 0.001642 | - |
| TS0-Cl, MP2/6-31+G* | -536.621871 | 0.034299 | 0.003335 | -459.8 |
| TS0-Cl, MP2/tzvpp | -536.795639 | 0.033975 | 0.003038 | -481.6 |
| $C_2H_2$Cl·, MP2/6-31+G* | -536.642222 | 0.035387 | 0.005016 | - |
| $C_2H_2$Cl·, MP2/tzvpp | -536.816394 | 0.035255 | 0.004862 | - |
| $C_2H_2$+Br·, MP2/SVP | -2649.367845 | 0.032829 | -0.001579 | - |
| $C_2H_2$+Br·, MP2/tzvpp | -2649.813398 | 0.033282 | -0.000459 | - |
| $C_2H_2$+Br·, B3LYP | -2651.179374 | 0.032998 | -0.002575 | - |
| TS0-Br, MP2/SVP | -2649.349920 | 0.033772 | 0.001685 | -461.6 |
| TS0-Br, MP2/tzvpp | -2649.798256 | 0.033613 | 0.001534 | -475.3 |
| TS0-Br, B3LYP | -2651.179004 | 0.031792 | -0.000963 | -145.9 |

| Geometry | E(elec) | H(corr) | G(corr) | Imaginary [a] frequency |
|---|---|---|---|---|
| $C_2H_2Br\cdot$, MP2/SVP | -2649.359819 | 0.034843 | 0.003104 | - |
| $C_2H_2Br\cdot$, MP2/tzvpp | -2649.809048 | 0.034805 | 0.003081 | - |
| $C_2H_2Br\cdot$, B3LYP | -2651.186714 | 0.032948 | 0.000800 | - |
| $C_2H_2$+I·, MP2/SVP | -6994.367503 | 0.032468 | -0.003374 | - |
| $C_2H_2$+I·, MP2/tzvpp | -374.294876 | 0.032980 | -0.002122 | - |
| $C_2H_2$+I·, B3LYP | -6997.199754 | 0.033010 | -0.002759 | - |
| TS0-I, MP2/SVP | -6994.340452 | 0.033060 | 0.000257 | -456.3 |
| TS0-I, MP2/tzvpp | -374.271678 | 0.033069 | 0.000271 | -480.1 |
| TS0-I, B3LYP | -6997.195695 | 0.031333 | -0.001871 | -223.2 |
| $C_2H_2I\cdot$, MP2/SVP | -6994.342968 | 0.034385 | 0.001641 | - |
| $C_2H_2I\cdot$, MP2/tzvpp | -374.275712 | 0.034371 | 0.001702 | - |
| $C_2H_2I\cdot$, B3LYP | -6997.196485 | 0.032433 | -0.001064 | - |
| **Figure S15** | | | | |
| $C_2H_2Cl\cdot$+$BCl_3$ | -1940.377476 | 0.050396 | -0.003991 | - |
| TS1-Cl | -1940.373213 | 0.049974 | 0.002815 | -104.3 |
| IM-Cl | -1940.404925 | 0.051286 | 0.006366 | - |
| $C_2H_2Br\cdot$+$BBr_3$, MP2/SVP | -10391.234473 | 0.048728 | -0.009743 | - |
| $C_2H_2Br\cdot$+$BBr_3$, MP2/tzvpp | -10392.782002 | 0.048726 | -0.008883 | - |
| TS1-Br, MP2/SVP | -10391.232591 | 0.048132 | -0.004146 | -112.3 |
| TS1-Br, MP2/tzvpp | -10392.781602 | 0.047975 | -0.004572 | -66.5 |
| IM-Br, MP2/SVP | -10391.276724 | 0.049991 | -0.000192 | - |
| IM-Br, MP2/tzvpp | -10392.825868 | 0.049623 | -0.000620 | - |
| $C_2H_2I\cdot$+$BI_3$ | - 27771.136372 | 0.047681 | -0.013894 | - |
| TS1-I | - 27771.135903 | 0.047014 | -0.009527 | -64.9 |
| IM-I | - 27771.205350 | 0.049900 | -0.004264 | - |
| **Figure S16** | | | | |
| IM+$BCl_3$ | -3344.144351 | 0.066396 | 0.001429 | - |
| TS2-Cl | -3344.136767 | 0.066274 | 0.003173 | -211.9 |
| E+$BCl_3$+Cl· | -3344.140371 | 0.071054 | 0.004791 | - |

| Geometry | E(elec) | H(corr) | G(corr) | Imaginary [a] frequency |
|---|---|---|---|---|
| IM+$BBr_3$ | -18133.154925 | 0.063855 | -0.010574 | - |
| TS2-Br | -18133.153026 | 0.062546 | -0.010543 | -151.7 |
| IM2+$BBr_3$ | -18133.155271 | 0.063680 | -0.013108 | - |
| TS3 | -18133.143551 | 0.062492 | -0.008397 | -164.6 |
| E+$BBr_4$· | -18133.147350 | 0.065777 | -0.005163 | - |
| IM+$BI_3$ | -48548.005683 | 0.062665 | -0.017352 | - |
| TS2-I | -48547.999019 | 0.061850 | -0.017106 | -114.3 |
| E+$BI_3$+I· | -48548.001490 | 0.062554 | -0.021565 | - |
| **Figure S17** | | | | |
| IM+$BBr_3$, CCSD(T)//MP2 | -18137.081425 | - | - | - |
| TS2-Br, CCSD(T)//MP2 | -18137.072538 | - | - | - |
| E+$BBr_3$+Br·, CCSD(T)//MP2 | -18137.076444 | - | - | - |
| IM+$BBr_3$, MP2/tzvpp | -18135.803341 | 0.063490 | -0.010377 | - |
| TS2-Br, MP2/tzvpp | -18135.796534 | 0.062988 | -0.008939 | -188.2 |
| E+$BBr_3$+Br· | -18135.801356 | 0.071723 | -0.003085 | - |
| **Figure S18** | | | | |
| $C_3H_4$+Cl· | -575.814360 | 0.064134 | 0.027086 | - |
| TS0-1-Cl | -575.802111 | 0.064491 | 0.029917 | -437.6 |
| $C_3H_4Cl$ (1) | -575.817874 | 0.065519 | 0.031638 | - |
| TS0-2-Cl | -575.806556 | 0.064661 | 0.028198 | -317.9 |
| $C_3H_4Cl$ (2) | -575.821983 | 0.066176 | 0.031267 | - |
| $C_3H_4$+Br· | -2688.545370 | 0.063967 | 0.025272 | - |
| TS0-1-Br | -2688.528128 | 0.064202 | 0.028614 | -424.4 |
| $C_3H_4Br$· (1) | -2688.534846 | 0.065162 | 0.029940 | - |
| TS0-2-Br | -2688.532895 | 0.064317 | 0.027034 | -365.4 |
| $C_3H_4Br$ (2) | -2688.539493 | 0.065622 | 0.029281 | - |
| $C_3H_4$+I· | -7033.544221 | 0.063677 | 0.023469 | - |
| TS0-1-I | -7033.516378 | 0.063644 | 0.027397 | -416.8 |

| Geometry | E(elec) | H(corr) | G(corr) | Imaginary [a] frequency |
|---|---|---|---|---|
| $C_3H_4I$ (1) | -7033.517483 | 0.064805 | 0.028496 | - |
| TS0-2-I | -7033.522898 | 0.063801 | 0.026425 | -340.1 |
| $C_3H_4I$ (2) | -7033.523650 | 0.065067 | 0.027636 | - |
| **Figure S19** | | | | |
| $C_3H_4Cl\cdot+BCl_3$, MP2 | -1979.560105 | 0.081226 | 0.025746 | - |
| $C_3H_4Cl\cdot+BCl_3$, B3LYP | -1982.429061 | 0.077483 | 0.024880 | *-11.5* |
| TS1-Cl, MP2 | -1979.555352 | 0.080639 | 0.031357 | -190.9 |
| TS1-Cl, B3LYP | -1982.427163 | 0.077692 | 0.026642 | -137.5 |
| IM-Cl, MP2 | -1979.585304 | 0.080994 | 0.031267 | - |
| IM-Cl, B3LYP | -1982.456470 | 0.079390 | 0.029562 | - |
| $C_3H_4Br\cdot+BBr_3$, MP2 | -10430.416711 | 0.079463 | 0.018286 | - |
| $C_3H_4Br\cdot+BBr_3$, B3LYP | -10437.111018 | 0.076008 | 0.018160 | *-11.1* |
| TS1-Br, MP2 | -10430.414193 | 0.078843 | 0.023704 | -110.7 |
| TS1-Br, B3LYP | -10437.110059 | 0.076214 | 0.018331 | -82.7 |
| IM-Br, MP2 | -10430.452987 | 0.079752 | 0.026532 | - |
| IM-Br, B3LYP | -10437.146488 | 0.078389 | 0.023875 | - |
| $C_3H_4I\cdot+BI_3$, MP2 | -27810.319770 | 0.078305 | 0.013816 | - |
| $C_3H_4I\cdot+BI_3$, B3LYP | -27821.119065 | 0.075960 | 0.009266 | - |
| TS1-I, MP2 | -27810.317873 | 0.077726 | 0.018323 | -118.7 |
| TS1-I, B3LYP | -27821.118342 | 0.075357 | 0.014613 | -79.4 |
| IM-I, MP2 | -27810.370001 | 0.079204 | 0.022603 | - |
| IM-I, B3LYP | -27821.162060 | 0.078201 | 0.019382 | - |
| **Figure S20** | | | | |
| IM+$BCl_3$, MP2 | -3383.316565 | 0.095472 | 0.032719 | - |
| IM+$BCl_3$, B3LYP | -3388.040146 | 0.094104 | 0.024686 | - |
| TS2-Cl, MP2 | -3383.310657 | 0.094620 | 0.032210 | -59.5 |
| TS2-Cl, B3LYP | -3388.027781 | 0.092867 | 0.029922 | -88.0 |
| E+$BCl_4\cdot$, MP2 | -3383.311361 | 0.096203 | 0.032055 | - |
| E+$BCl_4\cdot$, B3LYP | -3388.029078 | 0.094160 | 0.028893 | - |

| Geometry | E(elec) | H(corr) | G(corr) | Imaginary [a] frequency |
|---|---|---|---|---|
| IM+$BBr_3$, MP2 | -18172.331866 | 0.093536 | 0.016767 | - |
| IM+$BBr_3$, B3LYP | -18183.730542 | 0.091634 | 0.017902 | - |
| TS2-Br, MP2 | -18172.319449 | 0.092842 | 0.019993 | -190.3 |
| TS2-Br, B3LYP | -18183.728757 | 0.090917 | 0.019415 | -72.4 |
| E+$BBr_4$·, MP2 | -18172.321522 | 0.093957 | 0.020792 | - |
| E+$BBr_4$·, B3LYP | -18183.730818 | 0.092229 | 0.016935 | - |
| IM+$BI_3$ | -48587.168159 | 0.092292 | 0.008805 | - |
| TS2 | -48587.156113 | 0.091152 | 0.012481 | -190.8 |
| E+$BI_4$· | -48587.159262 | 0.093386 | 0.014288 | - |
| **Figure S21** | | | | |
| IM+$BI_3$ | -48605.759227 | 0.091529 | 0.009271 | - |
| TS2 | -48605.756977 | 0.090730 | 0.009833 | -64.6 |
| E+$BI_3$+I· | -48605.757944 | 0.091929 | 0.008347 | - |
| TS3 | -48605.750555 | 0.089750 | 0.010207 | -95.2 |
| E+$BI_4$· | -48605.760435 | 0.091376 | 0.009805 | - |

[a] Values for imaginary frequencies given in italics refer to geometry optimizations to minima each of which provided a single small imaginary frequency value.

## Optimized Cartesian Coordinates

**Table S2.** Cartesian coordinates (in Å) for structures referring to Figure 1.

| $C_2H_2$+$BBr_3$ | | x | y | z |
|---|---|---|---|---|
| | B | -0.017288 | 0.000641 | -0.382102 |
| | C | -0.456824 | -0.005675 | 3.221183 |
| | C | 0.755156 | -0.005159 | 3.058727 |
| | Br | -1.916748 | 0.008766 | -0.339362 |
| | Br | 0.936818 | 1.641774 | -0.429015 |
| | Br | 0.922703 | -1.648464 | -0.433240 |
| | H | -1.517309 | -0.006196 | 3.357498 |
| | H | 1.816696 | -0.004680 | 2.930114 |
| TS-Br | | x | y | z |
| | B | -0.099771 | 0.000013 | 0.315346 |
| | C | 1.433060 | -0.000270 | 1.964191 |
| | C | 0.170981 | -0.000024 | 1.951840 |
| | Br | 1.800485 | -0.000527 | -0.539890 |
| | Br | -1.057804 | -1.667275 | -0.157164 |
| | Br | -1.056804 | 1.667858 | -0.157211 |
| | H | 2.493696 | -0.000440 | 2.138876 |
| | H | -0.624764 | 0.000156 | 2.687484 |
| Z-Br | | x | y | z |
| | B | -0.794768 | -0.215223 | 0.210125 |
| | C | 1.480151 | -1.518341 | 0.508128 |
| | C | 0.140667 | -1.369437 | 0.619700 |
| | Br | 2.701922 | -0.285782 | -0.223440 |
| | Br | -2.613248 | -0.676198 | -0.186610 |
| | Br | -0.299661 | 1.621672 | 0.133952 |
| | H | 1.981933 | -2.429564 | 0.819398 |
| | H | -0.348468 | -2.256872 | 1.026440 |
| $C_2H_2$+$BCl_3$ | | x | y | z |
| | C | -2.498380 | -0.305109 | -0.096607 |
| | C | -1.368128 | -0.725936 | 0.098536 |
| | H | -3.487576 | 0.064317 | -0.265676 |
| | H | -0.377271 | -1.094219 | 0.260861 |
| | B | -2.418821 | -0.600629 | 3.400062 |
| | Cl | -1.191000 | 0.616255 | 3.582516 |
| | Cl | -4.088038 | -0.135812 | 3.253605 |
| | Cl | -1.984237 | -2.283886 | 3.415893 |
| TS-Cl | | x | y | z |
| | B | -0.176089 | -0.000001 | 0.117419 |
| | C | 1.773589 | -0.000003 | 1.208495 |
| | C | 0.572814 | -0.000003 | 1.599309 |
| | Cl | 1.322931 | 0.000015 | -1.085374 |
| | Cl | -1.135031 | -1.523400 | -0.076672 |
| | Cl | -1.135050 | 1.523389 | -0.076657 |
| | H | 2.839982 | -0.000002 | 1.068763 |
| | H | 0.063607 | -0.000025 | 2.555266 |

| Z-Cl | | x | y | z |
|---|---|---|---|---|
| | B | -0.849461 | -0.102740 | 0.067706 |
| | C | 1.524028 | -1.242640 | 0.144676 |
| | C | 0.174881 | -1.243604 | 0.219359 |
| | Cl | 2.536421 | 0.117487 | -0.161059 |
| | Cl | -2.535008 | -0.549811 | -0.142477 |
| | Cl | -0.460987 | 1.598487 | 0.116856 |
| | H | 2.102061 | -2.154503 | 0.260161 |
| | H | -0.235455 | -2.239102 | 0.390666 |
| $C_2H_2$+$BI_3$ | | x | y | z |
| | B | 0.006023 | -0.000937 | -0.278725 |
| | C | 0.524056 | 0.011831 | 3.508850 |
| | C | -0.685420 | 0.012381 | 3.328245 |
| | I | 2.141094 | -0.034165 | -0.275925 |
| | I | -1.088944 | -1.833268 | -0.297458 |
| | I | -1.031391 | 1.864320 | -0.303437 |
| | H | 1.583346 | 0.011597 | 3.653931 |
| | H | -1.745473 | 0.012765 | 3.188581 |
| TS-I | | x | y | z |
| | B | -0.000086 | -0.023268 | 0.275256 |
| | C | 0.001081 | 0.585725 | 2.635245 |
| | C | -0.001467 | -0.615424 | 2.369043 |
| | I | -1.875207 | -1.055110 | -0.227462 |
| | I | 0.005511 | 2.126199 | -0.236081 |
| | I | 1.869756 | -1.064667 | -0.227395 |
| | H | 0.003384 | 1.640728 | 2.816444 |
| | H | -0.003808 | -1.686578 | 2.401257 |
| IM | | x | y | z |
| | B | -0.000014 | -0.032896 | 0.422512 |
| | C | -0.000015 | 0.630895 | 2.497686 |
| | C | -0.000069 | -0.576895 | 2.245845 |
| | I | -1.874948 | -1.058438 | -0.217382 |
| | I | 0.000291 | 2.113635 | -0.237978 |
| | I | 1.874670 | -1.058928 | -0.217363 |
| | H | 0.000027 | 1.681561 | 2.707754 |
| | H | -0.000138 | -1.643361 | 2.372823 |
| TS2 | | x | y | z |
| | B | -0.084223 | -0.000117 | 0.459058 |
| | C | 1.184297 | 0.002155 | 2.289154 |
| | C | -0.063848 | -0.000194 | 2.117769 |
| | I | -1.118731 | 1.876152 | -0.155239 |
| | I | 2.088417 | 0.003667 | -0.331867 |
| | I | -1.112059 | -1.880072 | -0.155237 |
| | H | 2.213895 | 0.004186 | 2.595742 |
| | H | -0.969692 | -0.001946 | 2.711613 |
| Z-I | | x | y | z |
| | B | 0.668804 | 0.006050 | 0.613564 |
| | C | -1.712452 | -0.116644 | 1.603030 |
| | C | -0.377649 | -0.072470 | 1.754258 |
| | I | 1.551724 | -1.795193 | -0.126747 |
| | I | -2.642695 | -0.092431 | -0.301311 |
| | I | 1.310215 | 1.913643 | -0.108140 |
| | H | -2.419696 | -0.175127 | 2.423973 |
| | H | -0.003647 | -0.099487 | 2.783014 |

**Table S3.** Cartesian coordinates (in Å) for structures referring to Figure 2

| $C_2H_2$+$BCl_3$+$Cl^-$ | | x | y | z |
|---|---|---|---|---|
| | B | 1.321404 | -0.480864 | 0.029178 |
| | C | -1.089702 | 1.885731 | -0.098651 |
| | C | -0.149311 | 2.667391 | -0.141258 |
| | Cl | 0.170486 | -1.395846 | -0.892082 |
| | Cl | 2.636735 | 0.328862 | -0.777521 |
| | Cl | 1.208565 | -0.424744 | 1.763292 |
| | H | -1.935241 | 1.216799 | -0.063815 |
| | H | 0.682136 | 3.338776 | -0.173645 |
| | Cl | -3.893424 | -0.241801 | -0.003629 |
| TS2-Cl | | x | y | z |
| | B | 1.112909 | 0.011257 | -0.000001 |
| | C | -1.243265 | 0.028483 | -0.000347 |
| | C | -0.567121 | -1.000597 | -0.000268 |
| | Cl | 1.033386 | 1.819778 | 0.001343 |
| | Cl | 1.809567 | -0.664723 | 1.523285 |
| | Cl | 1.809136 | -0.662554 | -1.524423 |
| | H | -1.664572 | 1.010663 | -0.000388 |
| | H | -0.347450 | -2.050296 | -0.000355 |
| | Cl | -4.222102 | -0.091558 | 0.000056 |
| IM2-Cl | | x | y | z |
| | B | -0.789197 | -0.041658 | -0.001084 |
| | C | 1.784614 | 0.329583 | 0.002934 |
| | C | 0.733080 | -0.497026 | -0.014818 |
| | Cl | -1.627504 | -0.806267 | 1.495873 |
| | Cl | -1.001698 | 1.809772 | 0.064866 |
| | Cl | -1.629674 | -0.696443 | -1.548758 |
| | H | 1.718570 | 1.410766 | 0.025239 |
| | H | 0.927676 | -1.571435 | -0.037718 |
| | Cl | 3.446733 | -0.226262 | -0.006735 |
| $C_2H_2$+$BBr_3$+$Br^-$ | | x | y | z |
| | B | 1.581872 | 0.000029 | 0.567142 |
| | C | -1.156825 | 0.000205 | -1.791446 |
| | C | -0.116398 | -0.000064 | -2.437880 |
| | Br | 0.151153 | -0.000123 | 1.809896 |
| | Br | 2.326749 | -1.644624 | -0.029450 |
| | Br | 2.326685 | 1.644760 | -0.029284 |
| | H | -2.087578 | 0.000326 | -1.243889 |
| | H | 0.790029 | -0.000192 | -3.004749 |
| | Br | -4.350284 | -0.000099 | -0.103469 |
| TS1-Br | | x | y | z |
| | B | -0.005131 | 0.000160 | 0.035574 |
| | C | 0.012647 | 0.000246 | 2.483273 |
| | C | 1.111374 | 0.000062 | 1.928593 |
| | Br | -1.955383 | 0.000718 | 0.113758 |
| | Br | 0.786137 | 1.663027 | -0.621326 |
| | Br | 0.785223 | -1.663249 | -0.621044 |
| | H | -0.951595 | 0.000471 | 2.988298 |
| | H | 2.146924 | -0.000137 | 1.656280 |
| | Br | -2.718020 | 0.001188 | 4.589464 |

| IM1-Br | | x | y | z |
|---|---|---|---|---|
| | B | 1.016232 | -0.000160 | -0.112445 |
| | C | -0.899333 | -0.000686 | -1.198473 |
| | C | 0.156513 | -0.001499 | -1.836812 |
| | Br | -0.005040 | 0.006177 | 1.592045 |
| | Br | 2.074331 | -1.668395 | -0.322800 |
| | Br | 2.081459 | 1.662702 | -0.330320 |
| | H | -1.855371 | -0.000179 | -0.667086 |
| | H | 0.878647 | -0.002806 | -2.630037 |
| | Br | -4.140679 | -0.000002 | -0.308323 |
| TS2-Br | | x | y | z |
| | B | -0.020785 | 0.000178 | 0.084157 |
| | C | -0.078709 | 0.000221 | 2.332037 |
| | C | 0.948464 | 0.001579 | 1.641045 |
| | Br | -2.020118 | 0.000408 | 0.127704 |
| | Br | 0.673595 | 1.665903 | -0.761178 |
| | Br | 0.673398 | -1.666733 | -0.758800 |
| | H | -1.019732 | -0.001209 | 2.848805 |
| | H | 2.009559 | 0.002742 | 1.472688 |
| | Br | -0.893111 | -0.003480 | 5.336344 |
| IM2-Br | | x | y | z |
| | B | 0.689872 | 0.029860 | -0.042849 |
| | C | -1.871878 | -0.206476 | 0.287790 |
| | C | -0.833266 | 0.279489 | -0.404471 |
| | Br | 0.963111 | -1.137781 | 1.598658 |
| | Br | 1.593307 | -0.863309 | -1.651202 |
| | Br | 1.580720 | 1.848154 | 0.275826 |
| | H | -1.796360 | -0.827837 | 1.171985 |
| | H | -1.037503 | 0.896801 | -1.281660 |
| | Br | -3.690985 | 0.134184 | -0.194024 |
| $C_2H_2$+$BI_3$+$I^-$ | | x | y | z |
| | B | -1.537748 | -0.000383 | 0.308766 |
| | C | 1.211479 | -0.000055 | -2.253245 |
| | C | 0.162619 | 0.007330 | -2.884987 |
| | I | 0.150051 | -0.001349 | 1.605263 |
| | I | -2.403672 | 1.848160 | -0.326083 |
| | I | -2.403721 | -1.847927 | -0.328975 |
| | H | 2.134103 | -0.004492 | -1.699029 |
| | H | -0.745777 | 0.012302 | -3.448597 |
| | I | 4.620660 | 0.000182 | -0.300522 |
| TS1-I | | x | y | z |
| | B | -1.212438 | 0.002642 | -0.033398 |
| | C | 0.883422 | 0.006040 | -1.507577 |
| | C | -0.188336 | 0.046005 | -2.111098 |
| | I | 0.095764 | -0.166776 | 1.710948 |
| | I | -2.186028 | 1.943712 | -0.301143 |
| | I | -2.388667 | -1.785197 | -0.497061 |
| | H | 1.833307 | -0.017980 | -0.991223 |
| | H | -0.993878 | 0.087050 | -2.814833 |
| | I | 4.498786 | 0.000816 | -0.428120 |

| IM1-I | | x | y | z |
| --- | --- | --- | --- | --- |
| | B | -1.019446 | 0.003335 | -0.222667 |
| | C | 0.878057 | -0.010581 | -1.174408 |
| | C | -0.170674 | 0.021026 | -1.832494 |
| | I | 0.045630 | -0.098286 | 1.758335 |
| | I | -2.178528 | 1.919403 | -0.380579 |
| | I | -2.296772 | -1.823424 | -0.498398 |
| | H | 1.838333 | -0.026739 | -0.661136 |
| | H | -0.813749 | 0.052018 | -2.692907 |
| | I | 4.426432 | 0.000333 | -0.454664 |
| TS2-I | | X | y | z |
| | B | 0.983512 | -0.000023 | -0.158726 |
| | C | -1.160037 | 0.000493 | -0.570641 |
| | C | -0.260320 | -0.000219 | -1.426817 |
| | I | 0.449609 | 0.001386 | 2.036267 |
| | I | 2.108738 | -1.873898 | -0.673805 |
| | I | 2.110151 | 1.872453 | -0.675851 |
| | H | -1.914835 | 0.001018 | 0.195540 |
| | H | 0.094946 | -0.000834 | -2.442469 |
| | I | -4.566149 | 0.000027 | -0.403115 |
| IM2-I | | x | y | z |
| | B | -0.656324 | 0.004797 | -0.043162 |
| | C | 1.908753 | -0.046326 | 0.401464 |
| | C | 0.871713 | 0.048699 | -0.445815 |
| | I | -1.028719 | -0.248275 | 2.184835 |
| | I | -1.606882 | 1.965271 | -0.758679 |
| | I | -1.613735 | -1.742653 | -1.178321 |
| | H | 1.821122 | -0.166738 | 1.475338 |
| | H | 1.079151 | 0.168266 | -1.511636 |
| | I | 3.941762 | 0.024907 | -0.238058 |

**Table S4.** Cartesian coordinates (in Å) for structures referring to Figure 3

| IM2+$BCl_3$ | | x | y | z |
| --- | --- | --- | --- | --- |
| | B | 1.258610 | 0.292941 | 0.081582 |
| | C | 3.791313 | -0.134921 | -0.320683 |
| | C | 2.659745 | -0.409589 | 0.337076 |
| | Cl | 1.315837 | 1.599008 | -1.246508 |
| | Cl | 0.018767 | -1.029555 | -0.421871 |
| | Cl | 0.675903 | 1.073496 | 1.684857 |
| | Cl | 5.312837 | -0.943194 | 0.002152 |
| | H | 2.692127 | -1.178429 | 1.112252 |
| | H | 3.883537 | 0.604933 | -1.106911 |
| | B | -3.107286 | -0.175537 | -0.017952 |
| | Cl | -3.110429 | -0.853822 | 1.584469 |
| | Cl | -2.801255 | 1.519289 | -0.246510 |
| | Cl | -3.531580 | -1.173838 | -1.381404 |

| TS3-Cl | | x | y | z |
|---|---|---|---|---|
| | B | 1.090240 | 0.476285 | 0.072031 |
| | C | 3.541970 | -0.264406 | -0.309757 |
| | C | 2.420811 | -0.310595 | 0.419519 |
| | Cl | 1.198700 | 1.522006 | -1.446657 |
| | Cl | -0.231626 | -0.895142 | -0.236827 |
| | Cl | 0.554677 | 1.492592 | 1.527962 |
| | Cl | 4.997416 | -1.138944 | 0.110661 |
| | H | 2.418332 | -0.925572 | 1.321744 |
| | H | 3.660190 | 0.312248 | -1.219624 |
| | B | -2.557612 | -0.312523 | -0.022985 |
| | Cl | -2.767120 | -0.452912 | 1.729963 |
| | Cl | -2.593833 | 1.307157 | -0.728353 |
| | Cl | -3.188704 | -1.643903 | -1.015921 |
| $E+BCl_4^-$ | | x | y | z |
| | B | 1.089970 | 0.681354 | 0.088252 |
| | C | 3.415925 | -0.332769 | -0.303957 |
| | C | 2.325451 | -0.215215 | 0.466210 |
| | Cl | 1.187386 | 1.599149 | -1.472010 |
| | Cl | -0.315177 | -0.846914 | -0.248929 |
| | Cl | 0.471831 | 1.670632 | 1.476666 |
| | Cl | 4.795016 | -1.302375 | 0.135576 |
| | H | 2.299593 | -0.751516 | 1.415955 |
| | H | 3.545467 | 0.159813 | -1.260902 |
| | B | -2.209279 | -0.408232 | -0.036899 |
| | Cl | -2.591941 | -0.456368 | 1.747075 |
| | Cl | -2.515257 | 1.227027 | -0.773017 |
| | Cl | -3.072844 | -1.743269 | -0.946850 |
| $IM2+BBr_3$ | | x | y | z |
| | B | 1.523920 | 0.281221 | -0.012413 |
| | C | 4.049125 | -0.257004 | -0.267446 |
| | C | 2.925454 | -0.286992 | 0.461162 |
| | Br | 1.563884 | 1.129539 | -1.858873 |
| | Br | 0.185003 | -1.276204 | -0.022345 |
| | Br | 0.900151 | 1.674385 | 1.352570 |
| | Br | 5.711351 | -0.967435 | 0.356372 |
| | H | 2.961580 | -0.738181 | 1.454861 |
| | H | 4.134484 | 0.161879 | -1.263100 |
| | B | -3.144842 | -0.163749 | 0.036787 |
| | Br | -3.149685 | 0.062843 | 1.923312 |
| | Br | -2.773678 | 1.309183 | -1.100400 |
| | Br | -3.648148 | -1.839465 | -0.711858 |
| TS3-Br | | x | y | z |
| | B | 1.329559 | 0.757304 | 0.011718 |
| | C | 3.269982 | -0.979289 | -0.170082 |
| | C | 2.833717 | 0.265463 | 0.065331 |
| | Br | 0.079754 | -0.780280 | -0.618294 |
| | Br | 0.758930 | 1.376022 | 1.853262 |
| | Br | 1.173912 | 2.280960 | -1.323004 |
| | Br | 5.113581 | -1.465033 | -0.062910 |
| | H | 3.568713 | 1.029093 | 0.329789 |
| | H | 2.646151 | -1.824304 | -0.436847 |
| | B | -2.396926 | -0.451465 | -0.004545 |
| | Br | -2.659514 | 1.435597 | -0.309732 |
| | Br | -3.110921 | -1.666846 | -1.345182 |
| | Br | -2.427178 | -1.079021 | 1.825851 |

| E+$BBr_4^-$ | | x | y | z |
|---|---|---|---|---|
| | B | -0.014836 | -0.062109 | -0.011422 |
| | C | 0.008551 | 0.035863 | 2.554427 |
| | C | 0.684373 | -0.042033 | 1.397171 |
| | Br | -1.995897 | 0.017779 | -0.034994 |
| | Br | 0.697917 | 1.948978 | -0.751570 |
| | Br | 0.731856 | -1.450339 | -1.213913 |
| | Br | 0.863270 | 0.022375 | 4.253025 |
| | H | 1.772338 | -0.117841 | 1.427587 |
| | H | -1.070133 | 0.109020 | 2.635205 |
| | B | 0.726675 | 2.154108 | -2.865804 |
| | Br | 2.382110 | 1.281014 | -3.545934 |
| | Br | -0.925587 | 1.345145 | -3.609592 |
| | Br | 0.781329 | 4.136350 | -3.143221 |
| IM2+$BI_3$ | | x | y | z |
| | B | 1.746664 | 0.160805 | 0.104957 |
| | C | 4.298021 | -0.014343 | -0.389937 |
| | C | 3.173194 | -0.519664 | 0.141901 |
| | I | 1.694252 | 2.156743 | -0.980382 |
| | I | 0.325718 | -1.313079 | -0.928675 |
| | I | 1.078407 | 0.453084 | 2.278218 |
| | I | 6.181492 | -1.009043 | -0.283418 |
| | H | 3.226074 | -1.484440 | 0.651687 |
| | H | 4.363246 | 0.931392 | -0.916302 |
| | B | -3.343543 | -0.078672 | -0.023334 |
| | I | -3.331860 | -1.116217 | 1.842380 |
| | I | -2.738040 | 1.960695 | -0.118992 |
| | I | -4.048313 | -1.069042 | -1.783759 |
| TS3-I | | x | y | z |
| | B | 1.575658 | 0.696175 | 0.011171 |
| | C | 3.501470 | -1.054567 | -0.253102 |
| | C | 3.067095 | 0.176405 | 0.061868 |
| | I | 0.131940 | -0.900560 | -0.794266 |
| | I | 0.969274 | 1.289053 | 2.120764 |
| | I | 1.510592 | 2.509543 | -1.372077 |
| | I | 5.549607 | -1.628163 | -0.124679 |
| | H | 3.799738 | 0.913645 | 0.399362 |
| | H | 2.872651 | -1.869295 | -0.594648 |
| | B | -2.625917 | -0.423803 | -0.008642 |
| | I | -2.785525 | 1.724185 | -0.230536 |
| | I | -3.508879 | -1.659743 | -1.576109 |
| | I | -2.637433 | -1.242565 | 2.001998 |
| E+$BI_4^-$ | | x | y | z |
| | B | 1.533807 | 0.614138 | 0.084293 |
| | C | 3.882183 | -0.373717 | -0.367380 |
| | C | 2.766412 | -0.320418 | 0.381972 |
| | I | 1.713933 | 1.962396 | -1.690500 |
| | I | -0.098900 | -1.090861 | -0.451858 |
| | I | 0.901378 | 1.697653 | 1.935189 |
| | I | 5.535285 | -1.623386 | 0.102255 |
| | H | 2.703855 | -0.948239 | 1.273265 |
| | H | 4.045775 | 0.211603 | -1.265796 |
| | B | -2.310051 | -0.425360 | -0.052334 |
| | I | -2.730921 | -0.599652 | 2.139902 |
| | I | -2.607443 | 1.650508 | -0.815360 |
| | I | -3.520123 | -1.921988 | -1.224436 |

**Table S5.** Cartesian coordinates (in Å) for structures referring to Figure 4

| $C_3H_4$+$BBr_3$ | | x | y | z |
|---|---|---|---|---|
| | B | -0.460333 | 0.013163 | 0.323374 |
| | C | 2.212352 | -0.079900 | -1.843773 |
| | C | 1.311028 | -0.134763 | -2.671878 |
| | Br | 0.346206 | 1.622705 | 0.929992 |
| | Br | -1.975966 | 0.084943 | -0.820541 |
| | Br | 0.192586 | -1.665120 | 0.928342 |
| | C | 3.341227 | -0.014454 | -0.912485 |
| | H | 0.500569 | -0.185715 | -3.367325 |
| | H | 3.500871 | 1.011796 | -0.569319 |
| | H | 4.258164 | -0.362198 | -1.397260 |
| | H | 3.155531 | -0.643472 | -0.036902 |
| TS1-Br | | x | y | z |
| | B | 0.190350 | 0.000111 | -0.018510 |
| | C | -1.638792 | 0.001719 | 1.864998 |
| | C | -0.423178 | 0.003745 | 2.075177 |
| | Br | -1.269811 | -0.012636 | -1.306843 |
| | Br | 1.189951 | 1.671533 | 0.081292 |
| | Br | 1.207844 | -1.659886 | 0.093098 |
| | C | -3.052219 | -0.000461 | 1.524086 |
| | H | 0.515160 | 0.006768 | 2.592359 |
| | H | -3.313860 | 0.888731 | 0.944750 |
| | H | -3.634029 | 0.003784 | 2.452567 |
| | H | -3.313296 | -0.895224 | 0.953165 |
| IM-Br | | x | y | z |
| | B | 0.126907 | 0.000091 | 0.121167 |
| | C | -1.589001 | 0.000223 | 1.785214 |
| | C | -0.358187 | 0.000901 | 1.926198 |
| | Br | -1.295132 | -0.004364 | -1.261497 |
| | Br | 1.197832 | 1.671344 | 0.069011 |
| | Br | 1.204335 | -1.667155 | 0.072712 |
| | C | -3.014100 | -0.000310 | 1.542882 |
| | H | 0.531128 | 0.001958 | 2.530115 |
| | H | -3.322280 | 0.896149 | 1.000067 |
| | H | -3.499799 | 0.000866 | 2.528060 |
| | H | -3.322076 | -0.898146 | 1.002249 |
| TS2-Br | | x | y | z |
| | B | 0.219431 | 0.000004 | 0.252909 |
| | C | -1.877693 | -0.000027 | 1.325307 |
| | C | -0.631712 | 0.000007 | 1.626622 |
| | Br | -1.306899 | -0.000257 | -1.176432 |
| | Br | 1.297844 | 1.665798 | 0.104607 |
| | Br | 1.298253 | -1.665540 | 0.104806 |
| | C | -3.319229 | -0.000003 | 1.340591 |
| | H | -0.213997 | 0.000067 | 2.632572 |
| | H | -3.735715 | 0.901864 | 0.887917 |
| | H | -3.561810 | 0.000051 | 2.417490 |
| | H | -3.735742 | -0.901903 | 0.888006 |

| Z-Br | | x | y | z |
|---|---|---|---|---|
| | B | -0.887615 | -0.189982 | 0.167599 |
| | C | 1.559823 | -1.198394 | 0.301860 |
| | C | 0.211087 | -1.227654 | 0.445082 |
| | Br | 2.500143 | 0.312803 | -0.377679 |
| | Br | -2.631212 | -0.881143 | -0.252581 |
| | Br | -0.696652 | 1.702380 | 0.292628 |
| | C | 2.470055 | -2.347018 | 0.609572 |
| | H | -0.155167 | -2.200796 | 0.780342 |
| | H | 3.000376 | -2.669576 | -0.292543 |
| | H | 1.893725 | -3.186696 | 1.007420 |
| | H | 3.223608 | -2.046047 | 1.344809 |
| $C_3H_4$+$BCl_3$ | | x | y | z |
| | B | 0.691762 | -0.091758 | -0.448401 |
| | C | -2.143554 | 0.361065 | 1.312090 |
| | C | -1.361863 | 0.890729 | 2.091343 |
| | Cl | -0.233222 | 0.605514 | -1.745951 |
| | Cl | 1.885626 | 0.865048 | 0.378041 |
| | Cl | 0.475468 | -1.765212 | -0.028641 |
| | C | -3.144912 | -0.256493 | 0.439361 |
| | H | -0.646620 | 1.342911 | 2.745195 |
| | H | -4.077336 | -0.422044 | 0.986419 |
| | H | -2.791257 | -1.221408 | 0.064835 |
| | H | -3.359446 | 0.386339 | -0.418849 |
| TS2-Cl | | x | y | z |
| | B | 0.441641 | 0.042032 | 0.175397 |
| | C | -1.815506 | 0.093497 | 0.678555 |
| | C | -0.705552 | 0.229312 | 1.304642 |
| | Cl | -0.684017 | -0.258936 | -1.366378 |
| | Cl | 1.410998 | 1.565219 | -0.050487 |
| | Cl | 1.460495 | -1.430642 | 0.498522 |
| | C | -3.215056 | 0.003406 | 0.335746 |
| | H | -0.569924 | 0.426635 | 2.365397 |
| | H | -3.726917 | 0.172704 | 1.297130 |
| | H | -3.483124 | -0.986969 | -0.036842 |
| | H | -3.516468 | 0.783292 | -0.365904 |
| Z-Cl | | x | y | z |
| | B | 0.832556 | 0.180417 | 0.587299 |
| | C | -1.732513 | -0.182494 | 0.078025 |
| | C | -0.685257 | 0.286395 | 0.799947 |
| | Cl | -1.532530 | -1.074109 | -1.403082 |
| | Cl | 1.848407 | 1.327086 | 1.453590 |
| | Cl | 1.636897 | -1.016763 | -0.399478 |
| | C | -3.169456 | 0.023248 | 0.440396 |
| | H | -0.992267 | 0.866659 | 1.670961 |
| | H | -3.248033 | 0.578985 | 1.377343 |
| | H | -3.672005 | -0.943129 | 0.551270 |
| | H | -3.687587 | 0.574767 | -0.350851 |

| $C_3H_4$+$BI_3$ | | x | y | z |
|---|---|---|---|---|
| | B | 0.246026 | -0.006987 | -0.336451 |
| | C | -1.691595 | 0.063755 | 2.715876 |
| | C | -0.576568 | 0.116773 | 3.221185 |
| | I | -0.860083 | -1.790019 | -0.731126 |
| | I | -0.635715 | 1.902843 | -0.701336 |
| | I | 2.261933 | -0.136298 | 0.357569 |
| | C | -3.062335 | 0.008347 | 2.202265 |
| | H | 0.416935 | 0.161576 | 3.613509 |
| | H | -3.152207 | 0.589052 | 1.279266 |
| | H | -3.762793 | 0.418867 | 2.935659 |
| | H | -3.354189 | -1.023707 | 1.987187 |

| TS-I | | x | y | z |
|---|---|---|---|---|
| | B | -0.103188 | 0.000036 | 0.043620 |
| | C | 1.215041 | 0.000307 | 2.505183 |
| | C | -0.010739 | 0.000660 | 2.363260 |
| | I | -1.217167 | 1.873032 | -0.069408 |
| | I | 1.857347 | -0.002554 | -0.905551 |
| | I | -1.221308 | -1.870593 | -0.068118 |
| | C | 2.668311 | -0.000088 | 2.574309 |
| | H | -1.056515 | 0.001209 | 2.592800 |
| | H | 3.081550 | -0.891966 | 2.095186 |
| | H | 2.972923 | 0.000781 | 3.626924 |
| | H | 3.082103 | 0.890647 | 2.093535 |

| IM-I | | x | y | z |
|---|---|---|---|---|
| | B | -0.050118 | 0.000073 | 0.323794 |
| | C | 1.289794 | 0.000075 | 2.278011 |
| | C | 0.059253 | 0.000407 | 2.066607 |
| | I | -1.223887 | 1.877051 | -0.054326 |
| | I | 1.839882 | -0.002304 | -0.891684 |
| | I | -1.227639 | -1.874807 | -0.053235 |
| | C | 2.712195 | -0.000112 | 2.490070 |
| | H | -0.897338 | 0.000876 | 2.566827 |
| | H | 3.181611 | -0.902093 | 2.091074 |
| | H | 2.834023 | 0.000774 | 3.585374 |
| | H | 3.181990 | 0.901047 | 2.089646 |

| TS2-I | | x | y | z |
|---|---|---|---|---|
| | B | -0.109807 | -0.000167 | 0.346614 |
| | C | 1.691736 | 0.001064 | 1.933394 |
| | C | 0.413647 | -0.000727 | 1.875795 |
| | I | -1.289548 | 1.872202 | 0.009145 |
| | I | 1.810318 | 0.002578 | -0.931345 |
| | I | -1.285996 | -1.874427 | 0.007428 |
| | C | 3.081460 | -0.001812 | 2.300446 |
| | H | -0.254241 | -0.001494 | 2.738120 |
| | H | 3.597521 | -0.906931 | 1.974518 |
| | H | 3.039685 | -0.001047 | 3.405725 |
| | H | 3.601935 | 0.900459 | 1.973665 |

| Z-I | | x | y | z |
|---|---|---|---|---|
| | B | -0.697760 | 0.000021 | 0.582355 |
| | C | 1.774920 | 0.000380 | 1.333363 |
| | C | 0.457162 | 0.000308 | 1.611045 |
| | I | -1.545187 | 1.857979 | -0.062821 |
| | I | 2.391485 | 0.000121 | -0.722538 |
| | I | -1.544519 | -1.858308 | -0.062630 |
| | C | 2.896120 | 0.000674 | 2.325578 |
| | H | 0.183028 | 0.000498 | 2.672184 |
| | H | 3.529853 | -0.883477 | 2.200064 |
| | H | 2.482778 | 0.000790 | 3.339716 |
| | H | 3.529649 | 0.884930 | 2.199778 |

**Table S6.** Cartesian coordinates (in Å) for structures referring to Figure 5

| $C_3H_4$+$BCl_3$+$Cl^-$ | | x | y | z |
|---|---|---|---|---|
| | B | -2.110489 | 0.210660 | 0.100769 |
| | C | 1.080634 | -0.705082 | -0.347643 |
| | C | 0.521410 | -1.791997 | -0.424330 |
| | Cl | -1.764124 | 1.328626 | -1.185857 |
| | Cl | -3.051237 | -1.217676 | -0.221000 |
| | Cl | -1.599181 | 0.566920 | 1.723644 |
| | C | 1.763924 | 0.587759 | -0.273265 |
| | H | 0.052581 | -2.751566 | -0.475254 |
| | Cl | 5.501605 | -0.067609 | 0.095247 |
| | H | 1.574844 | 1.173130 | -1.178101 |
| | H | 2.843787 | 0.443955 | -0.169834 |
| | H | 1.405349 | 1.162673 | 0.586201 |
| TS1-Cl | | x | y | z |
| | B | -1.635179 | -0.024461 | 0.009947 |
| | C | 0.924893 | -0.171531 | -0.089142 |
| | C | 0.160548 | -1.134525 | -0.180976 |
| | Cl | -1.421408 | 1.748152 | -0.001386 |
| | Cl | -2.411399 | -0.692304 | -1.458212 |
| | Cl | -2.137090 | -0.725759 | 1.576132 |
| | C | 1.736845 | 1.024078 | 0.025651 |
| | H | -0.173155 | -2.149089 | -0.267888 |
| | Cl | 5.119309 | -0.329835 | -0.027192 |
| | H | 1.544718 | 1.705429 | -0.807133 |
| | H | 2.791824 | 0.716963 | 0.002268 |
| | H | 1.538783 | 1.536572 | 0.971014 |
| IM1+$Cl^-$ | | x | y | z |
| | B | -1.518637 | -0.065941 | 0.000744 |
| | C | 0.900528 | -0.113149 | -0.013270 |
| | C | 0.101779 | -1.057697 | -0.020914 |
| | Cl | -1.390259 | 1.737639 | 0.082748 |
| | Cl | -2.238904 | -0.645895 | -1.558351 |
| | Cl | -2.270449 | -0.776543 | 1.489819 |
| | C | 1.747726 | 1.052001 | -0.008250 |
| | H | -0.146225 | -2.102580 | -0.039460 |
| | Cl | 5.033560 | -0.364856 | 0.004266 |
| | H | 2.786567 | 0.680669 | -0.028179 |
| | H | 1.600947 | 1.642219 | 0.899448 |
| | H | 1.574584 | 1.666603 | -0.895109 |

| TS2-Cl | | x | y | z |
|---|---|---|---|---|
| | B | -1.198254 | -0.134571 | 0.001408 |
| | C | 1.150256 | 0.511194 | 0.038377 |
| | C | 0.512498 | -0.560073 | 0.076727 |
| | Cl | -1.582302 | 1.641261 | -0.213553 |
| | Cl | -1.776430 | -1.111379 | -1.434819 |
| | Cl | -1.835880 | -0.768130 | 1.595740 |
| | C | 1.648825 | 1.861764 | 0.004024 |
| | H | 0.660282 | -1.625349 | 0.143803 |
| | Cl | 4.021258 | -0.652114 | 0.001286 |
| | H | 2.732279 | 1.774354 | 0.147332 |
| | H | 1.224802 | 2.450199 | 0.821615 |
| | H | 1.451437 | 2.332491 | -0.961692 |
| IM2-Cl | | x | y | z |
| | B | -0.951454 | -0.125307 | 0.000089 |
| | C | 1.671307 | 0.062820 | 0.000178 |
| | C | 0.547829 | -0.674305 | 0.000118 |
| | Cl | -1.317898 | 0.910275 | -1.524903 |
| | Cl | -2.117790 | -1.594908 | 0.000464 |
| | Cl | -1.318390 | 0.911447 | 1.524117 |
| | C | 1.811220 | 1.551472 | -0.000084 |
| | H | 0.678262 | -1.757112 | 0.000635 |
| | Cl | 3.244756 | -0.758861 | 0.000201 |
| | H | 2.364677 | 1.885063 | 0.884743 |
| | H | 0.826596 | 2.018260 | 0.000191 |
| | H | 2.364067 | 1.885200 | -0.885239 |
| $C_3H_4$+$BBr_3$+$Br^-$ | | x | y | z |
| | B | -1.961158 | -0.054459 | 0.244805 |
| | C | 1.289362 | -0.029299 | -0.883160 |
| | C | 0.656320 | 0.422909 | -1.831203 |
| | Br | -1.345835 | 1.211700 | 1.519357 |
| | Br | -2.995369 | 0.524074 | -1.243552 |
| | Br | -1.647317 | -1.908547 | 0.513914 |
| | C | 1.971017 | -0.603990 | 0.277263 |
| | H | 0.182049 | 0.848525 | -2.689511 |
| | Br | 5.716994 | -0.078051 | 0.253306 |
| | H | 1.573733 | -0.171274 | 1.201326 |
| | H | 3.047481 | -0.409632 | 0.236674 |
| | H | 1.815929 | -1.687216 | 0.311473 |
| TS1-Br | | x | Y | z |
| | B | -1.584383 | 0.020331 | 0.001408 |
| | C | 1.027391 | -0.051874 | -0.472744 |
| | C | 0.288816 | 0.419198 | -1.342442 |
| | Br | -1.331062 | 1.255025 | 1.471605 |
| | Br | -2.829709 | 0.575788 | -1.388546 |
| | Br | -1.558429 | -1.876510 | 0.396460 |
| | C | 1.761413 | -0.634705 | 0.636109 |
| | H | -0.069792 | 0.883740 | -2.236991 |
| | Br | 5.252231 | 0.129993 | -0.292192 |
| | H | 1.481185 | -0.141054 | 1.572841 |
| | H | 2.835343 | -0.484575 | 0.462079 |
| | H | 1.553364 | -1.705855 | 0.713057 |

| IM1+$Br^-$ | | x | y | z |
|---|---|---|---|---|
| | B | 1.290988 | -0.116272 | 0.001532 |
| | C | -1.131936 | 0.251537 | 0.000895 |
| | C | -0.285659 | -0.686924 | 0.012796 |
| | Br | 1.431465 | 1.893304 | -0.075752 |
| | Br | 2.110328 | -0.814077 | 1.700681 |
| | Br | 2.131048 | -0.942304 | -1.628855 |
| | C | -2.047595 | 1.318168 | -0.007923 |
| | H | -0.411829 | -1.764373 | 0.033540 |
| | Br | -5.049821 | -0.352692 | 0.002590 |
| | H | -3.042488 | 0.767902 | -0.001393 |
| | H | -2.009759 | 1.907626 | -0.926988 |
| | H | -2.005423 | 1.925402 | 0.899338 |
| TS2-Br | | x | y | z |
| | B | 1.191203 | -0.107061 | -0.002956 |
| | C | -1.158948 | 0.475371 | -0.007158 |
| | C | -0.446918 | -0.560829 | -0.026908 |
| | Br | 1.532660 | 1.874823 | 0.057150 |
| | Br | 1.919468 | -0.996086 | 1.643611 |
| | Br | 1.943456 | -0.897344 | -1.688267 |
| | C | -1.908967 | 1.679028 | 0.015325 |
| | H | -0.637655 | -1.625927 | -0.054748 |
| | Br | -4.760085 | -0.358651 | -0.009046 |
| | H | -2.952581 | 1.274686 | 0.004586 |
| | H | -1.756477 | 2.283906 | -0.881448 |
| | H | -1.762782 | 2.245238 | 0.938114 |
| IM2-Br | | x | y | z |
| | B | 0.773963 | -0.113512 | 0.002512 |
| | C | -1.825753 | 0.260378 | 0.006689 |
| | C | -0.757705 | -0.557720 | 0.016743 |
| | Br | 1.261702 | 1.050768 | 1.619773 |
| | Br | 1.928805 | -1.802793 | 0.073924 |
| | Br | 1.251231 | 0.909567 | -1.709358 |
| | C | -1.872639 | 1.754193 | -0.009112 |
| | H | -0.956532 | -1.629365 | 0.022271 |
| | Br | -3.599097 | -0.527379 | 0.013294 |
| | H | -2.411983 | 2.111496 | -0.893769 |
| | H | -0.860002 | 2.157707 | -0.020729 |
| | H | -2.397180 | 2.130902 | 0.876565 |
| $C_3H_4$+$BI_3$+$I^-$ | | x | y | z |
| | B | 1.872229 | 0.080306 | -0.435853 |
| | C | -1.188653 | 0.006774 | 1.505732 |
| | C | -0.349404 | -0.291906 | 2.347874 |
| | I | 1.011499 | -1.588910 | -1.448461 |
| | I | 1.254176 | 2.063756 | -0.921978 |
| | I | 3.416586 | -0.229825 | 1.011595 |
| | C | -2.150834 | 0.393496 | 0.472555 |
| | H | 0.340510 | -0.578369 | 3.112523 |
| | I | -5.467180 | 0.746347 | -1.740019 |
| | H | -1.883582 | -0.049269 | -0.491113 |
| | H | -2.178049 | 1.480322 | 0.353400 |
| | H | -3.159515 | 0.058428 | 0.724470 |

| TS1-I | x | y | z |
|---|---|---|---|
| B | 1.634281 | -0.018438 | 0.000826 |
| C | -1.097219 | 0.155132 | 0.440886 |
| C | -0.380900 | -0.285630 | 1.344418 |
| I | 1.324083 | -1.527154 | -1.530189 |
| I | 1.650677 | 2.071707 | -0.594333 |
| I | 2.955428 | -0.553211 | 1.656272 |
| C | -1.806456 | 0.680800 | -0.712671 |
| H | -0.046258 | -0.711691 | 2.266616 |
| I | -5.598656 | -0.084297 | 0.360945 |
| H | -1.464316 | 0.179578 | -1.625337 |
| H | -1.645665 | 1.758506 | -0.808903 |
| H | -2.878928 | 0.490608 | -0.585173 |
| IM1+I$^-$ | x | y | z |
| B | -1.186260 | -0.047115 | -0.015173 |
| C | 1.209221 | 0.529060 | 0.039942 |
| C | 0.405979 | -0.454508 | -0.005979 |
| I | -1.515731 | 2.196094 | 0.063919 |
| I | -2.016153 | -0.900493 | -1.932085 |
| I | -2.073143 | -1.041899 | 1.805788 |
| C | 2.146073 | 1.576144 | 0.089041 |
| H | 0.677648 | -1.508573 | -0.039370 |
| I | 5.299447 | -0.391141 | 0.064228 |
| H | 3.112380 | 0.982615 | 0.081739 |
| H | 2.120400 | 2.142393 | 1.022880 |
| H | 2.145300 | 2.204986 | -0.804228 |
| TS2-I | x | y | z |
| B | -1.152462 | -0.123172 | -0.023615 |
| C | 1.189827 | 0.606575 | -0.003076 |
| C | 0.465321 | -0.434715 | -0.021447 |
| I | -1.629512 | 2.094133 | 0.025571 |
| I | -1.934093 | -1.056220 | -1.922188 |
| I | -1.957788 | -1.144103 | 1.818824 |
| C | 2.042684 | 1.731191 | 0.011627 |
| H | 0.800546 | -1.469728 | -0.037403 |
| I | 4.971558 | -0.639227 | 0.071669 |
| H | 3.036918 | 1.202951 | 0.010644 |
| H | 1.975490 | 2.315160 | 0.932215 |
| H | 1.976771 | 2.337044 | -0.894811 |
| IM2-I | x | y | z |
| B | -0.661014 | -0.039529 | -0.101041 |
| C | 1.889967 | 0.312571 | 0.458314 |
| C | 0.907758 | -0.242147 | -0.280995 |
| I | -1.278782 | 2.153475 | -0.389197 |
| I | -1.711724 | -1.284833 | -1.717108 |
| I | -1.396473 | -0.778421 | 1.945014 |
| C | 1.782036 | 1.243765 | 1.623814 |
| H | 1.213444 | -0.899190 | -1.094629 |
| I | 3.942915 | -0.155336 | -0.049508 |
| H | 2.254196 | 0.807563 | 2.511722 |
| H | 0.733249 | 1.445950 | 1.845726 |
| H | 2.287692 | 2.192342 | 1.408567 |

**Table S7.** Cartesian coordinates (in Å) for structures referring to Figure 6

| IM2+BCl$_3$ | | x | y | z |
|---|---|---|---|---|
| | B | -1.623182 | -0.886792 | 0.036920 |
| | C | -1.189224 | 1.704589 | -0.036359 |
| | C | -1.289673 | 0.549857 | 0.643163 |
| | Cl | -0.277002 | -1.477961 | -1.137254 |
| | Cl | -1.742398 | -2.113632 | 1.449619 |
| | Cl | -3.257547 | -0.901634 | -0.887621 |
| | C | -1.360854 | 1.944632 | -1.502103 |
| | H | -1.137015 | 0.607626 | 1.720990 |
| | Cl | -0.853738 | 3.200554 | 0.855120 |
| | H | -2.288292 | 2.496346 | -1.696358 |
| | H | -1.398458 | 0.993491 | -2.033671 |
| | H | -0.529923 | 2.540713 | -1.892883 |
| | Cl | 2.205095 | 1.385294 | -0.777663 |
| | B | 2.481606 | -0.109337 | 0.067500 |
| | Cl | 1.940649 | -0.295400 | 1.707599 |
| | Cl | 3.402593 | -1.376750 | -0.694999 |
| TS3-Cl | | x | y | z |
| | B | -1.190846 | -1.199156 | 0.050907 |
| | C | -1.723202 | 1.368864 | -0.043879 |
| | C | -1.459900 | 0.246350 | 0.647914 |
| | Cl | 0.478459 | -1.215245 | -0.923614 |
| | Cl | -1.037389 | -2.435286 | 1.426730 |
| | Cl | -2.485399 | -1.782873 | -1.148898 |
| | C | -1.838799 | 1.553919 | -1.523289 |
| | H | -1.418002 | 0.343550 | 1.732630 |
| | Cl | -2.000370 | 2.873378 | 0.846095 |
| | H | -2.885120 | 1.713976 | -1.809812 |
| | H | -1.464367 | 0.672771 | -2.044733 |
| | H | -1.266309 | 2.429399 | -1.843948 |
| | Cl | 1.614630 | 1.817718 | -0.738848 |
| | B | 2.101480 | 0.287231 | 0.009731 |
| | Cl | 1.812460 | 0.103996 | 1.744725 |
| | Cl | 3.535964 | -0.515503 | -0.666295 |
| E+BCl$_4^-$ | | x | y | z |
| | B | -1.112912 | -1.297690 | 0.093777 |
| | C | -1.910875 | 1.191184 | -0.047574 |
| | C | -1.517596 | 0.110892 | 0.653835 |
| | Cl | 0.759989 | -1.068276 | -0.859117 |
| | Cl | -0.811630 | -2.518734 | 1.408574 |
| | Cl | -2.104913 | -2.002618 | -1.264692 |
| | C | -2.037248 | 1.360754 | -1.527330 |
| | H | -1.471786 | 0.225676 | 1.736013 |
| | Cl | -2.350733 | 2.651022 | 0.838882 |
| | H | -3.093331 | 1.379946 | -1.822137 |
| | H | -1.542499 | 0.542813 | -2.050465 |
| | H | -1.584496 | 2.306909 | -1.836561 |
| | Cl | 1.358026 | 1.928949 | -0.714844 |
| | B | 1.889322 | 0.311721 | -0.054762 |
| | Cl | 1.724293 | 0.219596 | 1.755454 |
| | Cl | 3.578167 | -0.121851 | -0.616934 |

| IM2+BBr$_3$ | | x | y | z |
|---|---|---|---|---|
| | B | -1.854531 | -0.634695 | -0.001190 |
| | C | -1.199511 | 1.897330 | -0.242465 |
| | C | -1.411016 | 0.805193 | 0.514121 |
| | Br | -0.465221 | -1.473926 | -1.258284 |
| | Br | -2.053287 | -1.866888 | 1.621901 |
| | Br | -3.661194 | -0.578574 | -0.971509 |
| | C | -1.317293 | 2.061307 | -1.723065 |
| | H | -1.280075 | 0.918965 | 1.589661 |
| | Br | -0.702490 | 3.548904 | 0.644665 |
| | H | -2.130870 | 2.754046 | -1.969148 |
| | H | -1.520327 | 1.095912 | -2.188541 |
| | H | -0.390800 | 2.471383 | -2.139043 |
| | Br | 2.397181 | 1.383623 | -0.960832 |
| | B | 2.493210 | -0.192172 | 0.096204 |
| | Br | 1.802013 | -0.199465 | 1.861644 |
| | Br | 3.417157 | -1.719072 | -0.567858 |
| TS3-Br | | x | y | z |
| | B | -1.612840 | -0.864999 | 0.015059 |
| | C | -1.572463 | 1.744510 | -0.241712 |
| | C | -1.562878 | 0.636992 | 0.523827 |
| | Br | 0.124667 | -1.338050 | -1.052994 |
| | Br | -1.708579 | -2.120939 | 1.608796 |
| | Br | -3.178913 | -1.269671 | -1.221982 |
| | C | -1.636419 | 1.869490 | -1.729732 |
| | H | -1.513433 | 0.782656 | 1.602306 |
| | Br | -1.529125 | 3.467670 | 0.639712 |
| | H | -2.598946 | 2.295288 | -2.037966 |
| | H | -1.519500 | 0.889349 | -2.194052 |
| | H | -0.844617 | 2.533184 | -2.090907 |
| | Br | 1.970705 | 1.704805 | -0.883501 |
| | B | 2.159086 | 0.013334 | 0.025409 |
| | Br | 1.701646 | -0.053718 | 1.893745 |
| | Br | 3.544622 | -1.182900 | -0.606519 |
| E+BBr$_4^-$ | | x | y | z |
| | B | -1.551406 | -0.989977 | 0.062182 |
| | C | -1.804739 | 1.597260 | -0.250542 |
| | C | -1.669238 | 0.505105 | 0.529191 |
| | Br | 0.448204 | -1.240927 | -0.944574 |
| | Br | -1.534654 | -2.259366 | 1.600172 |
| | Br | -2.796043 | -1.630718 | -1.369640 |
| | C | -1.852311 | 1.705183 | -1.740190 |
| | H | -1.638875 | 0.667920 | 1.605533 |
| | Br | -1.990518 | 3.308826 | 0.620491 |
| | H | -2.866980 | 1.954618 | -2.073840 |
| | H | -1.552645 | 0.762557 | -2.199500 |
| | H | -1.179839 | 2.497073 | -2.081782 |
| | Br | 1.703862 | 1.849416 | -0.882616 |
| | B | 1.891819 | 0.045339 | -0.057013 |
| | Br | 1.603917 | 0.090838 | 1.906551 |
| | Br | 3.636489 | -0.803905 | -0.544870 |

| $IM2+BI_3$ | | x | y | z |
|---|---|---|---|---|
| | B | -2.033718 | -0.589788 | -0.055081 |
| | C | -1.286979 | 1.919106 | -0.363693 |
| | C | -1.525874 | 0.842579 | 0.413823 |
| | I | -0.543256 | -1.662270 | -1.429295 |
| | I | -2.316560 | -1.877719 | 1.824018 |
| | I | -4.069000 | -0.447102 | -1.116570 |
| | C | -1.408683 | 2.053643 | -1.847957 |
| | H | -1.402565 | 0.974805 | 1.487668 |
| | I | -0.763530 | 3.779499 | 0.612542 |
| | H | -2.267232 | 2.684954 | -2.108303 |
| | H | -1.544814 | 1.069667 | -2.301124 |
| | H | -0.511421 | 2.517113 | -2.271396 |
| | I | 2.575860 | 1.532768 | -1.188154 |
| | B | 2.704558 | -0.144380 | 0.125761 |
| | I | 1.828152 | -0.061892 | 2.064240 |
| | I | 3.810995 | -1.875882 | -0.471938 |

| TS3-I | | x | y | z |
|---|---|---|---|---|
| | B | -1.882821 | -0.690120 | -0.041578 |
| | C | -1.456681 | 1.889199 | -0.366071 |
| | C | -1.606928 | 0.801769 | 0.419887 |
| | I | -0.041983 | -1.549846 | -1.159082 |
| | I | -2.230292 | -1.972993 | 1.815862 |
| | I | -3.705130 | -0.905297 | -1.403825 |
| | C | -1.515399 | 1.989473 | -1.857514 |
| | H | -1.549765 | 0.960739 | 1.495428 |
| | I | -1.192557 | 3.802196 | 0.605600 |
| | H | -2.468644 | 2.429982 | -2.175815 |
| | H | -1.423060 | 0.996682 | -2.303335 |
| | H | -0.708846 | 2.623558 | -2.236917 |
| | I | 2.312147 | 1.657985 | -1.192155 |
| | B | 2.318957 | -0.127104 | 0.033444 |
| | I | 1.682120 | 0.056067 | 2.088429 |
| | I | 3.768972 | -1.673159 | -0.451365 |

| $E+BI_4^-$ | | x | y | z |
|---|---|---|---|---|
| | B | -1.807227 | -0.782605 | 0.000613 |
| | C | -1.664647 | 1.817994 | -0.379905 |
| | C | -1.693250 | 0.730153 | 0.422465 |
| | I | 0.270235 | -1.451211 | -1.025664 |
| | I | -2.093014 | -2.090853 | 1.815076 |
| | I | -3.361634 | -1.312827 | -1.553691 |
| | C | -1.708486 | 1.892419 | -1.873260 |
| | H | -1.648465 | 0.908431 | 1.495485 |
| | I | -1.628910 | 3.755029 | 0.567697 |
| | H | -2.710390 | 2.183408 | -2.213676 |
| | H | -1.460606 | 0.922538 | -2.309605 |
| | H | -0.996780 | 2.635484 | -2.242541 |
| | I | 2.055126 | 1.815991 | -1.135144 |
| | B | 1.977143 | -0.154618 | -0.072348 |
| | I | 1.606254 | 0.104695 | 2.112795 |
| | I | 3.838075 | -1.360581 | -0.467612 |

**Table S8.** Cartesian coordinates (in Å) for structures referring to Figure 7

| IM1+Cl⁻ | | x | y | z |
|---|---|---|---|---|
| | B | -1.518859 | -0.066011 | -0.000238 |
| | C | 0.900834 | -0.111423 | -0.006621 |
| | C | 0.103584 | -1.057097 | -0.022447 |
| | Cl | -1.392958 | 1.738896 | -0.047396 |
| | Cl | -2.288809 | -0.746374 | -1.494160 |
| | Cl | -2.217672 | -0.679526 | 1.555380 |
| | C | 1.746225 | 1.055042 | 0.013140 |
| | H | -0.144055 | -2.102177 | -0.031545 |
| | Cl | 5.033430 | -0.365143 | -0.009459 |
| | H | 1.586201 | 1.672101 | -0.874243 |
| | H | 2.785803 | 0.685277 | 0.007828 |
| | H | 1.584665 | 1.642200 | 0.920516 |
| TS4-Cl | | x | y | z |
| | B | 1.369241 | -0.110806 | -0.001708 |
| | C | -1.124277 | 0.402548 | -0.001386 |
| | C | -0.214422 | -0.502383 | -0.010436 |
| | Cl | 1.611987 | 1.727727 | -0.056164 |
| | Cl | 2.099895 | -0.807943 | 1.554515 |
| | Cl | 2.142391 | -0.902921 | -1.490030 |
| | C | -2.150997 | 1.298877 | 0.003080 |
| | H | -0.476936 | -1.561998 | -0.012032 |
| | Cl | -4.542494 | -0.559466 | -0.004667 |
| | H | -2.324320 | 1.846346 | 0.930387 |
| | H | -3.081323 | 0.445420 | -0.000255 |
| | H | -2.325691 | 1.854262 | -0.919223 |
| IM3+HCl | | x | y | z |
| | B | 1.271566 | -0.117570 | 0.008413 |
| | C | -1.120865 | 0.890439 | 0.085113 |
| | C | -0.317267 | -0.147845 | 0.154945 |
| | Cl | 1.919916 | 1.580182 | -0.384451 |
| | Cl | 2.033199 | -0.718660 | 1.616629 |
| | Cl | 1.769805 | -1.301751 | -1.360466 |
| | C | -1.933966 | 1.930963 | 0.010740 |
| | H | -0.769154 | -1.127010 | 0.341154 |
| | Cl | -4.395048 | -0.692060 | 0.017693 |
| | H | -2.179689 | 2.520409 | 0.891445 |
| | H | -3.402420 | 0.142344 | 0.041216 |
| | H | -2.347796 | 2.259663 | -0.940553 |
| IM1+Br⁻ | | x | y | z |
| | B | -1.090651 | -0.040024 | -0.004460 |
| | C | 1.238631 | 1.090181 | -0.020335 |
| | C | 0.496689 | 0.014761 | -0.035754 |
| | Br | -1.754511 | -1.022054 | -1.702073 |
| | Br | -1.678206 | -1.146366 | 1.645244 |
| | Br | -1.965136 | 1.805704 | 0.081890 |
| | C | 2.017635 | 2.151948 | -0.005353 |
| | H | 1.016203 | -0.946996 | -0.072909 |
| | Br | 4.648000 | -0.329699 | -0.011391 |
| | H | 2.312084 | 2.654072 | -0.926179 |
| | H | 3.507965 | 0.581235 | -0.018441 |
| | H | 2.344168 | 2.604970 | 0.930054 |

| TS4-Br | | x | y | z |
|---|---|---|---|---|
| | B | -1.234534 | -0.101589 | 0.003391 |
| | C | 1.217301 | 0.481257 | 0.006140 |
| | C | 0.350040 | -0.463573 | 0.017547 |
| | Br | -2.009314 | -0.895058 | -1.688538 |
| | Br | -2.058478 | -0.963008 | 1.638100 |
| | Br | -1.539768 | 1.901433 | 0.038638 |
| | C | 2.219367 | 1.417698 | -0.005789 |
| | H | 0.647646 | -1.513975 | 0.025573 |
| | Br | 4.892019 | -0.363738 | 0.008220 |
| | H | 2.339815 | 1.977255 | -0.935183 |
| | H | 3.165461 | 0.646964 | 0.000628 |
| | H | 2.343461 | 1.998423 | 0.909940 |
| IM3+HBr | | x | y | z |
| | B | 1.103214 | -0.043519 | 0.006470 |
| | C | -1.198380 | 1.146982 | 0.038183 |
| | C | -0.483120 | 0.041769 | 0.054491 |
| | Br | 1.750680 | -1.008796 | 1.696108 |
| | Br | 1.639728 | -1.165207 | -1.624528 |
| | Br | 1.994676 | 1.771859 | -0.109810 |
| | C | -1.946281 | 2.238394 | 0.020516 |
| | H | -1.023215 | -0.908910 | 0.106836 |
| | Br | -4.662866 | -0.324401 | 0.013375 |
| | H | -2.214566 | 2.755433 | 0.939795 |
| | H | -3.518392 | 0.550560 | 0.028485 |
| | H | -2.270824 | 2.686729 | -0.916687 |
| IM1+I$^-$ | | x | y | z |
| | B | -0.386189 | -0.000102 | -1.042295 |
| | C | -0.226781 | -0.000084 | 1.417657 |
| | C | 0.474486 | -0.000106 | 0.357967 |
| | I | -2.627605 | 0.000313 | -0.692325 |
| | I | 0.243007 | 1.870933 | -2.135455 |
| | I | 0.242375 | -1.871577 | -2.135061 |
| | C | -0.949765 | -0.000104 | 2.623321 |
| | H | 1.562126 | -0.000167 | 0.303811 |
| | I | 1.836964 | -0.000066 | 5.083394 |
| | H | -0.097501 | -0.000119 | 3.371400 |
| | H | -1.524754 | -0.914232 | 2.788124 |
| | H | -1.524746 | 0.914020 | 2.788168 |
| TS4-I | | x | y | z |
| | B | -1.117373 | -0.058517 | 0.000005 |
| | C | 1.278472 | 0.830341 | 0.000022 |
| | C | 0.476018 | -0.185823 | 0.000027 |
| | I | -1.758478 | 2.121427 | -0.000042 |
| | I | -1.878950 | -1.108205 | -1.867519 |
| | I | -1.879010 | -1.108145 | 1.867539 |
| | C | 2.284410 | 1.739906 | 0.000007 |
| | H | 0.928211 | -1.184876 | 0.000036 |
| | I | 4.992376 | -0.249515 | 0.000014 |
| | H | 3.243005 | 0.857357 | 0.000016 |
| | H | 2.478775 | 2.284387 | 0.925251 |
| | H | 2.478762 | 2.284365 | -0.925252 |

| IM3+HI | x | y | z |
|---|---|---|---|
| B | -1.005997 | 0.021662 | 0.009766 |
| C | 1.125360 | 1.504399 | 0.084623 |
| C | 0.546764 | 0.320256 | 0.074558 |
| I | -2.276503 | 1.897086 | -0.070706 |
| I | -1.388896 | -1.255622 | -1.859030 |
| I | -1.561284 | -1.208449 | 1.866786 |
| C | 1.750976 | 2.669541 | 0.094257 |
| H | 1.204266 | -0.555290 | 0.112398 |
| I | 4.770060 | -0.071606 | 0.026365 |
| H | 3.489755 | 0.970109 | 0.062399 |
| H | 1.961995 | 3.190841 | 1.026030 |
| H | 2.036445 | 3.166155 | -0.831234 |

**Table S9.** Cartesian coordinates (in Å) for structures referring to Figure 8

| IM3+HI | x | y | z |
|---|---|---|---|
| B | 0.946323 | -0.059480 | 0.294080 |
| C | -0.474009 | -1.424690 | 1.979283 |
| C | 0.239103 | -0.356435 | 1.675576 |
| I | 0.186758 | 1.991007 | -0.448222 |
| I | 3.205160 | 0.113468 | 0.660632 |
| I | 0.539765 | -1.624430 | -1.289463 |
| C | -1.172407 | -2.492744 | 2.314662 |
| H | 0.362802 | 0.401604 | 2.455806 |
| I | -3.769936 | 0.098676 | 0.234990 |
| H | -0.692455 | -3.371981 | 2.739158 |
| H | -2.283489 | 0.774436 | -0.005032 |
| H | -2.247164 | -2.535722 | 2.151945 |
| TS5 | x | y | z |
| B | -1.420579 | 0.582468 | 0.519692 |
| C | 0.275742 | 1.536154 | 2.082597 |
| C | -0.567974 | 0.551622 | 1.788517 |
| I | 0.688566 | -2.321076 | -0.384293 |
| I | -3.233340 | -0.589351 | 0.477596 |
| I | -0.983724 | 1.898205 | -1.124332 |
| C | 1.062816 | 2.528095 | 2.428870 |
| H | -0.696820 | -0.256158 | 2.510821 |
| I | 3.497230 | 0.346697 | 0.126977 |
| H | 0.673199 | 3.388415 | 2.968373 |
| H | 2.045219 | -0.980873 | -0.119225 |
| H | 2.114920 | 2.513911 | 2.156396 |
| IM4+HI+I$^-$ | x | y | z |
| B | 1.606140 | -0.602334 | 0.561725 |
| C | -0.242320 | -1.638736 | 1.881868 |
| C | 0.654085 | -0.663351 | 1.753948 |
| I | -0.801403 | 2.249547 | -0.541968 |
| I | 3.276941 | 0.759736 | 0.658653 |
| I | 1.401085 | -1.888706 | -1.148808 |
| C | -1.112346 | -2.607029 | 2.038694 |
| H | 0.728468 | 0.079920 | 2.549849 |
| I | -3.868251 | -0.417072 | 0.213810 |
| H | -0.835508 | -3.537109 | 2.529958 |
| H | -2.040020 | 1.123229 | -0.221257 |
| H | -2.123832 | -2.485449 | 1.656334 |

| TS6 | | x | y | z |
|---|---|---|---|---|
| | B | 1.151308 | -0.735349 | 0.471281 |
| | C | -1.024906 | -1.816385 | 1.070407 |
| | C | -0.000497 | -0.946321 | 1.378702 |
| | I | -0.651508 | 2.581846 | -0.671821 |
| | I | 2.881199 | 0.397218 | 1.193671 |
| | I | 1.272065 | -1.574132 | -1.536763 |
| | C | -1.324290 | -3.108157 | 0.929996 |
| | H | -0.067378 | -0.394205 | 2.318954 |
| | I | -3.258391 | -0.550624 | 0.514689 |
| | H | -0.574999 | -3.834507 | 1.243993 |
| | H | -1.653045 | 1.327949 | -0.214553 |
| | H | -2.261263 | -3.475660 | 0.532418 |
| IM5 | | x | y | z |
| | B | -0.695564 | 0.013179 | -0.187919 |
| | C | 1.677842 | -0.911545 | -0.977060 |
| | C | 0.460853 | -0.093060 | -1.311753 |
| | I | 0.077309 | 0.795110 | 1.808177 |
| | I | -2.261978 | 1.483810 | -1.010557 |
| | I | -1.735716 | -1.996626 | 0.169210 |
| | C | 1.772571 | -2.251515 | -1.032913 |
| | H | 0.003399 | -0.529208 | -2.212072 |
| | I | 3.461239 | 0.183730 | -0.461618 |
| | H | 0.899185 | -2.828716 | -1.325326 |
| | H | 0.763897 | 0.924666 | -1.581636 |
| | H | 2.678511 | -2.795231 | -0.787239 |

**Table S10.** Cartesian coordinates (in Å) for structures referring to Figure 9

| $C_2H_2$+Cl· | | x | y | z |
|---|---|---|---|---|
| | C | 1.529624 | -0.612698 | 0.000008 |
| | C | 1.529792 | 0.612588 | -0.000001 |
| | H | 1.553534 | 1.682295 | -0.000008 |
| | Cl | -1.262507 | 0.000046 | -0.000003 |
| | H | 1.552592 | -1.682421 | 0.000015 |
| TS0-Cl | | x | y | z |
| | C | -1.000913 | 0.616374 | 0.000005 |
| | C | -1.668786 | -0.395859 | 0.000001 |
| | H | -2.138708 | -1.357074 | -0.000003 |
| | Cl | 1.115293 | -0.096193 | -0.000002 |
| | H | -0.803082 | 1.669269 | 0.000009 |
| $C_2H_2$Cl· | | x | y | z |
| | C | -0.658336 | 0.512851 | 0.000005 |
| | C | -1.693308 | -0.243765 | 0.000000 |
| | H | -1.983859 | -1.282385 | -0.000008 |
| | Cl | 0.986908 | -0.113449 | -0.000002 |
| | H | -0.683708 | 1.596496 | 0.000013 |
| $C_2H_2$+Br· | | x | y | z |
| | C | -2.090161 | -0.613002 | 0.000010 |
| | C | -2.090029 | 0.613043 | -0.000001 |
| | Br | 0.837321 | -0.000008 | -0.000002 |
| | H | -2.112442 | 1.682700 | -0.000010 |
| | H | -2.112659 | -1.682658 | 0.000019 |

| TS0-Br | | x | y | z |
|---|---|---|---|---|
| | C | 1.445941 | 0.599939 | 0.000005 |
| | C | 2.164083 | -0.386907 | -0.000001 |
| | Br | -0.731723 | -0.045517 | -0.000001 |
| | H | 2.627538 | -1.351242 | -0.000008 |
| | H | 1.322614 | 1.666145 | 0.000013 |
| $C_2H_2Br\cdot$ | | x | y | z |
| | C | 1.166158 | 0.536216 | 0.000007 |
| | C | 2.140471 | -0.291347 | 0.000001 |
| | Br | -0.670146 | -0.049763 | -0.000002 |
| | H | 2.371472 | -1.344655 | -0.000008 |
| | H | 1.243854 | 1.617131 | 0.000018 |
| $C_2H_2$+I· | | x | y | z |
| | C | -0.612286 | -2.602092 | 0.000000 |
| | C | 0.612287 | -2.602162 | 0.000000 |
| | I | 0.000000 | 0.687926 | 0.000000 |
| | H | 1.681963 | -2.617334 | 0.000000 |
| | H | -1.681964 | -2.617226 | 0.000000 |
| TS0-I | | x | y | z |
| | C | 0.485447 | -1.731714 | 0.000000 |
| | C | -0.495081 | -2.483675 | 0.000000 |
| | I | 0.000000 | 0.564310 | 0.000000 |
| | H | -1.504124 | -2.842898 | 0.000000 |
| | H | 1.561930 | -1.773213 | 0.000000 |
| $C_2H_2I\cdot$, MP2/6-31+G* | | x | y | z |
| | C | 0.461451 | -1.573136 | 0.000000 |
| | C | -0.461586 | -2.449519 | 0.000000 |
| | I | 0.000000 | 0.537220 | 0.000000 |
| | H | -1.530501 | -2.590550 | 0.000000 |
| | H | 1.531312 | -1.746161 | 0.000000 |

**Table S11.** Cartesian coordinates (in Å) for structures referring to Figure 10

| $C_2H_2Cl\cdot$+$BCl_3$ | | x | y | z |
|---|---|---|---|---|
| | B | -1.649749 | 0.083522 | -0.051364 |
| | C | 2.243672 | 0.201633 | 0.059451 |
| | C | 1.417373 | -0.777166 | 0.086656 |
| | Cl | -1.725555 | -0.107366 | 1.675155 |
| | Cl | -1.248871 | 1.630592 | -0.738623 |
| | Cl | -2.021275 | -1.259445 | -1.092295 |
| | H | 1.965227 | 1.244989 | -0.037738 |
| | Cl | 3.985120 | -0.014589 | 0.174373 |
| | H | 1.374409 | -1.852480 | 0.168564 |
| TS1-Cl | | x | y | z |
| | B | 1.191974 | -0.043753 | -0.000059 |
| | C | -1.826089 | -0.285751 | 0.000062 |
| | C | -0.900373 | 0.599784 | 0.000475 |
| | Cl | 1.616137 | 0.753380 | 1.517766 |
| | Cl | 1.031372 | -1.799529 | -0.000708 |
| | Cl | 1.615766 | 0.754579 | -1.517347 |
| | H | -1.671654 | -1.357297 | -0.000336 |
| | Cl | -3.505642 | 0.174759 | 0.000082 |
| | H | -0.809212 | 1.677649 | 0.000941 |

| IM-Cl | x | y | z |
|---|---|---|---|
| B | -0.912819 | 0.046150 | -0.123482 |
| C | 1.668256 | 0.420978 | -0.072263 |
| C | 0.577717 | -0.378345 | -0.382397 |
| Cl | -1.252440 | 1.772740 | 0.261469 |
| Cl | -2.153508 | -0.763064 | -1.144648 |
| Cl | -0.505932 | -0.994373 | 1.502511 |
| H | 1.566355 | 1.390016 | 0.404054 |
| H | 0.772391 | -1.344764 | -0.846213 |
| Cl | 3.250087 | -0.046586 | -0.396537 |

| $C_2H_2Br\cdot$+$BBr_3$ | x | y | z |
|---|---|---|---|
| Br | -4.211062 | 0.009744 | -0.160998 |
| C | -2.309103 | -0.011548 | 0.142916 |
| C | -1.472640 | 0.051214 | -0.820438 |
| H | -1.401898 | 0.121804 | -1.894898 |
| H | -2.068329 | -0.081012 | 1.197486 |
| B | 1.555684 | -0.005254 | 0.087778 |
| Br | 1.815070 | 1.692006 | -0.726038 |
| Br | 1.815059 | -1.594128 | -0.921384 |
| Br | 1.106142 | -0.114836 | 1.931953 |

| TS1-Br | x | y | z |
|---|---|---|---|
| Br | -3.845576 | 0.173016 | -0.069194 |
| C | -1.987073 | -0.250696 | 0.099693 |
| C | -1.094991 | 0.598028 | -0.239378 |
| H | -1.002804 | 1.597911 | -0.638619 |
| H | -1.816329 | -1.244199 | 0.496293 |
| B | 1.243762 | -0.040917 | 0.016521 |
| Br | 1.621454 | 1.400543 | 1.223857 |
| Br | 1.622165 | 0.172266 | -1.851491 |
| Br | 1.033177 | -1.809629 | 0.722481 |

| IM-Br | x | y | z |
|---|---|---|---|
| B | -0.005744 | -0.002469 | -0.005360 |
| C | -0.002818 | -0.003447 | 2.593935 |
| C | 0.693349 | -0.013487 | 1.401295 |
| Br | -1.923630 | 0.408853 | -0.100921 |
| Br | 1.075590 | 0.692157 | -1.486893 |
| Br | 0.319364 | -2.133857 | 0.301239 |
| H | -1.086839 | -0.028450 | 2.640521 |
| H | 1.782737 | 0.010429 | 1.440830 |
| Br | 0.839057 | 0.041380 | 4.227503 |

| $C_2H_2I\cdot$+$BI_3$ | x | y | z |
|---|---|---|---|
| B | 1.567986 | -0.056498 | 0.075786 |
| C | -1.440539 | 0.452500 | -0.620175 |
| C | -2.287263 | -0.105538 | 0.147929 |
| I | 1.845828 | -0.891914 | -1.871501 |
| I | 1.810441 | 2.044231 | 0.383445 |
| I | 1.160151 | -1.337953 | 1.737301 |
| H | -1.342427 | 1.092601 | -1.483471 |
| H | -2.080014 | -0.742138 | 1.000487 |
| I | -4.406513 | 0.155338 | -0.158486 |

| TS1-I | | x | y | z |
|---|---|---|---|---|
| | B | 1.397688 | -0.103598 | -0.005810 |
| | C | -1.180075 | 0.335457 | -0.586114 |
| | C | -2.061480 | -0.217637 | 0.146514 |
| | I | 1.738931 | -0.968585 | -1.941404 |
| | I | 1.741633 | 1.993904 | 0.299910 |
| | I | 1.139502 | -1.388815 | 1.692773 |
| | H | -1.077503 | 0.983446 | -1.444140 |
| | H | -1.895934 | -0.863180 | 1.001224 |
| | I | -4.148841 | 0.086970 | -0.256222 |
| IM-I | | x | y | z |
| | B | 0.489000 | -0.078524 | 0.104095 |
| | C | -0.990698 | 0.214708 | -0.293521 |
| | C | -2.049849 | -0.273381 | 0.384175 |
| | I | 1.704470 | -0.695883 | -1.787127 |
| | I | 1.714972 | 1.904622 | 0.082370 |
| | I | 0.919860 | -1.339375 | 1.853893 |
| | H | -1.169376 | 0.847256 | -1.164704 |
| | H | -1.971682 | -0.909051 | 1.260065 |
| | I | -4.057070 | 0.128915 | -0.161311 |

**Table S12.** Cartesian coordinates (in Å) for structures referring to Figure 12

| IM+$BCl_3$ | | x | y | z |
|---|---|---|---|---|
| | B | 1.999328 | -0.760994 | 0.029983 |
| | C | 1.687529 | 1.829930 | -0.043283 |
| | C | 1.299177 | 0.586990 | 0.432041 |
| | Cl | 3.604877 | -0.682774 | -0.780842 |
| | Cl | 1.789174 | -2.125877 | 1.182143 |
| | Cl | 0.523735 | -0.683407 | -1.284796 |
| | H | 2.482712 | 1.956745 | -0.770394 |
| | H | 0.473349 | 0.551588 | 1.140980 |
| | Cl | 0.953209 | 3.254052 | 0.459380 |
| | B | -2.633862 | -0.166244 | 0.072587 |
| | Cl | -3.335322 | -1.410922 | -0.912627 |
| | Cl | -2.498215 | 1.458361 | -0.532972 |
| | Cl | -2.078849 | -0.537298 | 1.680539 |
| TS2-Cl | | x | y | z |
| | B | 2.189523 | -0.653006 | 0.194525 |
| | C | 1.517953 | 1.789131 | 0.035037 |
| | C | 1.433518 | 0.609098 | 0.723698 |
| | Cl | 3.685813 | -0.484267 | -0.723914 |
| | Cl | 1.839616 | -2.206388 | 0.939589 |
| | Cl | 0.431758 | -0.200238 | -1.431588 |
| | H | 2.199952 | 1.927530 | -0.798609 |
| | H | 0.714278 | 0.531504 | 1.538090 |
| | Cl | 0.660607 | 3.170926 | 0.450857 |
| | B | -2.555252 | -0.327948 | 0.108486 |
| | Cl | -3.203716 | -1.302054 | -1.175644 |
| | Cl | -2.652078 | 1.406826 | 0.025235 |
| | Cl | -1.867555 | -1.087373 | 1.515057 |

| $E+Cl^{\cdot}+BCl_3$ | x | y | z |
|---|---|---|---|
| B | -2.313844 | -0.686680 | -0.251164 |
| C | -1.596656 | 1.761206 | -0.079434 |
| C | -1.432621 | 0.525476 | -0.656584 |
| Cl | -3.692845 | -0.508994 | 0.805417 |
| Cl | -1.956482 | -2.248382 | -0.935757 |
| Cl | -0.213346 | 0.286133 | 1.616603 |
| H | -2.352164 | 1.961222 | 0.674158 |
| H | -0.667530 | 0.412706 | -1.423449 |
| Cl | -0.733670 | 3.141545 | -0.568405 |
| B | 2.574246 | -0.427296 | -0.152682 |
| Cl | 3.517484 | -0.466309 | 1.306354 |
| Cl | 2.423967 | 1.054789 | -1.052570 |
| Cl | 1.825089 | -1.877849 | -0.749015 |

| $IM+BBr_3$ | x | y | z |
|---|---|---|---|
| B | 2.188323 | -0.632055 | 0.039139 |
| C | 1.782494 | 1.934888 | -0.009572 |
| C | 1.412226 | 0.678067 | 0.424337 |
| Br | 3.953500 | -0.471260 | -0.804281 |
| Br | 1.982410 | -2.136980 | 1.277284 |
| Br | 0.604736 | -0.601481 | -1.467590 |
| H | 2.604733 | 2.100262 | -0.698685 |
| H | 0.567715 | 0.597043 | 1.109137 |
| Br | 0.915400 | 3.459668 | 0.535569 |
| B | -2.666994 | -0.220253 | 0.110430 |
| Br | -2.625817 | 1.592818 | -0.452947 |
| Br | -1.996956 | -0.691644 | 1.826531 |
| Br | -3.403199 | -1.554363 | -1.018763 |

| TS2-Br | x | y | z |
|---|---|---|---|
| B | 2.346314 | -0.604642 | 0.114295 |
| C | 1.855803 | 1.955986 | 0.088091 |
| C | 1.431240 | 0.639490 | 0.346960 |
| Br | 4.047707 | -0.427336 | -0.775303 |
| Br | 2.020410 | -2.185788 | 1.165378 |
| Br | 0.561409 | -0.280859 | -1.476583 |
| H | 2.744113 | 2.186154 | -0.488326 |
| H | 0.592917 | 0.523903 | 1.032742 |
| Br | 0.826733 | 3.412617 | 0.572184 |
| B | -2.676746 | -0.303720 | 0.118349 |
| Br | -2.857709 | 1.524726 | -0.356373 |
| Br | -1.907590 | -0.773057 | 1.794888 |
| Br | -3.302591 | -1.662906 | -1.047560 |

| $IM2+BBr_3$ | x | y | z |
|---|---|---|---|
| B | 2.234625 | -0.848915 | 0.173055 |
| C | 2.352479 | 1.738527 | 0.122914 |
| C | 1.517134 | 0.554828 | 0.175241 |
| Br | 3.889567 | -1.105226 | -0.730319 |
| Br | 1.430507 | -2.281811 | 1.128149 |
| Br | 0.449985 | 0.325273 | -1.562189 |
| H | 3.284640 | 1.775789 | -0.427020 |
| H | 0.724413 | 0.629312 | 0.920327 |
| Br | 1.638092 | 3.393125 | 0.584703 |
| B | -2.689570 | -0.213433 | 0.141530 |
| Br | -2.573442 | -2.075573 | -0.198510 |
| Br | -3.556960 | 0.934850 | -1.095530 |
| Br | -1.990664 | 0.499263 | 1.763549 |

| TS3 | | x | y | z |
|---|---|---|---|---|
| | B | 2.148057 | -0.807568 | 0.122462 |
| | C | 2.182485 | 1.763948 | -0.099715 |
| | C | 1.467712 | 0.608452 | 0.277635 |
| | Br | 3.390649 | -1.140446 | -1.273323 |
| | Br | 1.745200 | -2.124920 | 1.417342 |
| | Br | -0.141602 | 0.252065 | -1.232829 |
| | H | 3.001208 | 1.744789 | -0.810312 |
| | H | 0.796439 | 0.728826 | 1.129444 |
| | Br | 1.670570 | 3.438407 | 0.468740 |
| | B | -2.095781 | -0.181571 | -0.019179 |
| | Br | -2.114030 | -2.126602 | 0.166943 |
| | Br | -3.422250 | 0.554733 | -1.258264 |
| | Br | -1.870256 | 0.810697 | 1.657017 |

| E+$BBr_4^{\cdot}$ | | x | y | z |
|---|---|---|---|---|
| | B | 2.241308 | -0.475699 | 0.032532 |
| | C | 1.518488 | 1.929614 | -0.404145 |
| | C | 1.379855 | 0.785323 | 0.345563 |
| | Br | 2.673353 | -0.991386 | -1.742083 |
| | Br | 3.006066 | -1.375059 | 1.519918 |
| | Br | -0.791750 | 0.563043 | -1.411752 |
| | H | 2.061676 | 1.956528 | -1.346780 |
| | H | 0.843915 | 0.851886 | 1.292859 |
| | Br | 0.907971 | 3.587204 | 0.158585 |
| | B | -1.866425 | -0.481340 | 0.157249 |
| | Br | -0.789923 | -2.072738 | 0.622671 |
| | Br | -3.536685 | -0.894606 | -0.823801 |
| | Br | -2.102462 | 0.774604 | 1.660932 |

| IM+$BI_3$ | | x | y | z |
|---|---|---|---|---|
| | B | 0.327733 | 1.476734 | 1.336521 |
| | C | -1.050084 | 1.376421 | 0.615453 |
| | C | -2.080198 | 0.659202 | 1.106180 |
| | I | 2.064999 | 1.205985 | -0.201791 |
| | I | 0.972239 | 3.713276 | 1.470985 |
| | I | 0.669317 | 0.371015 | 3.207737 |
| | H | -1.187432 | 1.932823 | -0.313421 |
| | H | -2.034239 | 0.078012 | 2.021312 |
| | I | -3.978663 | 0.564577 | 0.175716 |
| | B | 0.154825 | -1.984486 | -1.583203 |
| | I | 2.171498 | -2.477587 | -2.104763 |
| | I | -0.701890 | -2.784242 | 0.194783 |
| | I | -0.986686 | -0.706972 | -2.851548 |

| TS2-I | | x | y | z |
|---|---|---|---|---|
| | B | 0.040311 | 1.685039 | 1.658706 |
| | C | -1.208655 | 1.478648 | 0.784698 |
| | C | -2.230429 | 0.690959 | 1.204999 |
| | I | 2.091648 | 1.086831 | -0.263333 |
| | I | 1.020272 | 3.658913 | 1.472380 |
| | I | 0.464587 | 0.525432 | 3.424241 |
| | H | -1.315633 | 2.044386 | -0.140763 |
| | H | -2.200275 | 0.107476 | 2.120039 |
| | I | -4.066281 | 0.515218 | 0.185452 |
| | B | 0.138100 | -1.885947 | -1.557411 |
| | I | 2.098676 | -2.446894 | -2.211832 |
| | I | -0.596314 | -2.605246 | 0.309548 |
| | I | -1.083177 | -0.656764 | -2.798633 |

| E+I·+BI$_3$ | x | y | z |
|---|---|---|---|
| B | -0.268416 | 1.837643 | 1.618067 |
| C | -1.604571 | 1.573770 | 0.916809 |
| C | -2.456627 | 0.611785 | 1.348262 |
| I | 3.321761 | 0.544174 | -0.002487 |
| I | 1.063643 | 3.274780 | 0.704934 |
| I | 0.326998 | 0.876538 | 3.438507 |
| H | -1.864955 | 2.151124 | 0.031019 |
| H | -2.268626 | -0.009610 | 2.219047 |
| I | -4.268984 | 0.156873 | 0.386968 |
| B | 0.297755 | -1.695879 | -1.611719 |
| I | 1.804354 | -2.764667 | -2.683571 |
| I | -0.360747 | -2.415231 | 0.286659 |
| I | -0.568754 | 0.056748 | -2.464411 |

**Table S13.** Cartesian coordinates (in Å) for structures referring to Figure 13

| $C_3H_4$+Cl· | x | y | z |
|---|---|---|---|
| C | -1.027219 | 0.415361 | 0.000012 |
| C | -0.391172 | 1.471132 | -0.000004 |
| C | -1.883522 | -0.768318 | -0.000001 |
| H | -2.933356 | -0.461857 | -0.001132 |
| H | -1.696939 | -1.376620 | 0.888677 |
| H | -1.695361 | -1.377742 | -0.887576 |
| H | 0.134225 | 2.403758 | -0.000001 |
| Cl | 1.529583 | -0.346858 | 0.000000 |
| **TS0-1-Cl** | **x** | **y** | **z** |
| C | -0.771262 | 0.306561 | -0.000023 |
| C | -0.725233 | 1.530288 | -0.000029 |
| C | -1.470739 | -0.988934 | -0.000018 |
| H | -1.196887 | -1.562553 | -0.887213 |
| H | -2.547442 | -0.803125 | -0.000078 |
| H | -1.196977 | -1.562495 | 0.887243 |
| H | -0.512432 | 2.578903 | -0.000034 |
| Cl | 1.368067 | -0.219895 | 0.000030 |
| **$C_3H_4$Cl· (1)** | **x** | **y** | **z** |
| C | 0.473063 | 0.244774 | 0.000010 |
| C | 0.785988 | 1.490243 | 0.000027 |
| C | 1.399508 | -0.931455 | 0.000014 |
| H | 1.224863 | -1.548979 | 0.885558 |
| H | 2.434457 | -0.583661 | 0.000035 |
| H | 1.224893 | -1.548961 | -0.885549 |
| H | 0.332493 | 2.468888 | 0.000029 |
| Cl | -1.245180 | -0.212274 | -0.000022 |
| **TS0-2-Cl** | **x** | **y** | **z** |
| C | -1.108529 | 0.432376 | 0.000051 |
| C | -0.105703 | 1.127054 | -0.000002 |
| C | -2.192249 | -0.530686 | 0.000013 |
| H | -2.811518 | -0.412718 | -0.891908 |
| H | -2.808625 | -0.416328 | 0.894392 |
| H | -1.765621 | -1.540197 | -0.002796 |
| H | 0.467977 | 2.031680 | -0.000064 |
| Cl | 1.609216 | -0.343230 | 0.000000 |

| $C_3H_4Cl\cdot$ (2) | x | y | z |
|---|---|---|---|
| C | 1.087732 | 0.619428 | 0.000015 |
| C | -0.164653 | 0.901876 | -0.000004 |
| C | 2.060867 | -0.476925 | -0.000003 |
| H | 2.699011 | -0.425824 | 0.886063 |
| H | 2.698883 | -0.425882 | -0.886166 |
| H | 1.538034 | -1.442764 | 0.000064 |
| H | -0.586104 | 1.899748 | -0.000021 |
| Cl | -1.426676 | -0.345385 | 0.000001 |

| $C_3H_4$+Br· | x | y | z |
|---|---|---|---|
| C | -0.199999 | -1.734308 | -0.634216 |
| C | 1.022105 | -1.588805 | -0.675591 |
| C | -1.636497 | -2.010642 | -0.624515 |
| H | -2.036465 | -1.929248 | 0.389744 |
| H | -1.822119 | -3.023614 | -0.993131 |
| H | -2.168194 | -1.302690 | -1.265951 |
| H | 2.085785 | -1.479471 | -0.718588 |
| Br | 0.181245 | 0.917150 | 0.327125 |

| TS0-1-Br | x | y | z |
|---|---|---|---|
| C | 0.087889 | 0.370648 | -0.020883 |
| C | 1.240951 | 0.006631 | -0.250694 |
| C | -1.355645 | 0.045418 | -0.026635 |
| H | -1.760670 | 0.122190 | 0.984217 |
| H | -1.482709 | -0.974213 | -0.398547 |
| H | -1.893840 | 0.742733 | -0.671539 |
| H | 2.299401 | -0.092930 | -0.373413 |
| Br | 0.248141 | 2.477802 | 0.757484 |

| $C_3H_4Br\cdot$ (1) | x | y | z |
|---|---|---|---|
| C | -0.029298 | -1.086557 | -0.405157 |
| C | 1.058182 | -1.682598 | -0.719218 |
| C | -1.440463 | -1.582477 | -0.477659 |
| H | -1.898271 | -1.565023 | 0.515360 |
| H | -1.447855 | -2.604891 | -0.862831 |
| H | -2.032419 | -0.941570 | -1.137063 |
| H | 2.127555 | -1.550077 | -0.756242 |
| Br | 0.105698 | 0.739130 | 0.273804 |

| TS0-2-Br | x | y | z |
|---|---|---|---|
| C | -0.048519 | 0.048545 | 0.041162 |
| C | 1.114330 | 0.440018 | 0.098747 |
| C | -1.483570 | -0.145417 | -0.050549 |
| H | -1.862931 | -0.643772 | 0.844839 |
| H | -1.736821 | -0.738673 | -0.932435 |
| H | -1.970419 | 0.833832 | -0.136958 |
| H | 2.157484 | 0.204991 | 0.192690 |
| Br | 1.213966 | 2.698755 | -0.057508 |

| $C_3H_4Br\cdot$ (2) | x | y | z |
|---|---|---|---|
| C | -0.656709 | -1.824073 | 0.080493 |
| C | 0.426791 | -1.144310 | 0.112454 |
| C | -2.115986 | -1.759228 | -0.029073 |
| H | -2.588663 | -2.188404 | 0.858532 |
| H | -2.461799 | -2.313417 | -0.905836 |
| H | -2.434415 | -0.712412 | -0.126192 |
| H | 1.436622 | -1.522641 | 0.210796 |
| Br | 0.411482 | 0.796594 | -0.020455 |

| $C_3H_4$+I· | | x | y | z |
|---|---|---|---|---|
| | C | -2.195771 | 0.348718 | 0.000000 |
| | C | -1.786514 | 1.507347 | -0.000001 |
| | C | -2.773399 | -0.996829 | 0.000001 |
| | H | -2.457010 | -1.552702 | -0.886957 |
| | H | -3.865503 | -0.935438 | 0.000001 |
| | H | -2.457009 | -1.552701 | 0.886960 |
| | H | -1.432219 | 2.516987 | -0.000002 |
| | I | 0.957469 | -0.068520 | 0.000000 |
| TS0-1-I | | x | y | z |
| | C | -1.521304 | 0.240255 | -0.000002 |
| | C | -1.836163 | 1.445497 | 0.000000 |
| | C | -2.176849 | -1.099472 | 0.000000 |
| | H | -1.878728 | -1.664790 | -0.885808 |
| | H | -3.261017 | -0.961283 | -0.000005 |
| | H | -1.878737 | -1.664782 | 0.885816 |
| | H | -1.697793 | 2.508856 | 0.000001 |
| | I | 0.790984 | -0.032749 | 0.000000 |
| $C_3H_4$I· (1) | | x | y | z |
| | C | -1.402860 | 0.231064 | -0.000018 |
| | C | -1.822005 | 1.432394 | -0.000028 |
| | C | -2.158083 | -1.064195 | -0.000020 |
| | H | -1.902314 | -1.652830 | -0.885277 |
| | H | -3.231175 | -0.855853 | -0.000032 |
| | H | -1.902332 | -1.652823 | 0.885247 |
| | H | -1.524738 | 2.468130 | -0.000030 |
| | I | 0.770910 | -0.035891 | 0.000009 |
| TS0-2-I | | x | y | z |
| | C | -2.122555 | 0.504088 | 0.000012 |
| | C | -1.052575 | 1.144200 | 0.000002 |
| | C | -3.016642 | -0.642803 | -0.000006 |
| | H | -3.651250 | -0.638033 | -0.889761 |
| | H | -3.651548 | -0.637812 | 0.889535 |
| | H | -2.419549 | -1.565223 | 0.000219 |
| | H | -0.740680 | 2.176297 | -0.000033 |
| | I | 0.898371 | -0.101286 | 0.000000 |
| $C_3H_4$I· (2) | | x | y | z |
| | C | 2.101048 | 0.572802 | 0.000007 |
| | C | 0.953818 | 1.120762 | -0.000002 |
| | C | 2.886450 | -0.660192 | -0.000002 |
| | H | 3.523287 | -0.711175 | 0.887182 |
| | H | 3.523120 | -0.711266 | -0.887300 |
| | H | 2.211155 | -1.527409 | 0.000107 |
| | H | 0.699960 | 2.172937 | -0.000010 |
| | I | -0.860480 | -0.102327 | 0.000000 |

**Table S14.** Cartesian coordinates (in Å) for structures referring to Figure 14

| $C_2H_2Cl\cdot{}+BCl_3$ | | x | y | z |
|---|---|---|---|---|
| | B | 1.714185 | -0.206072 | 0.035192 |
| | C | -1.276208 | 0.583221 | -0.079709 |
| | C | -2.005493 | -0.472516 | -0.066100 |
| | Cl | 1.432779 | -1.828677 | -0.526298 |
| | Cl | 2.140224 | 1.042006 | -1.100404 |
| | Cl | 1.647268 | 0.151729 | 1.736288 |
| | C | -1.308216 | 2.048378 | -0.076969 |
| | Cl | -3.774652 | -0.407407 | -0.023265 |
| | H | -1.639661 | -1.492121 | -0.080103 |
| | H | -0.884801 | 2.446046 | -1.003070 |
| | H | -0.735407 | 2.447179 | 0.764980 |
| | H | -2.347077 | 2.394686 | 0.011445 |
| TS1 | | x | y | z |
| | B | -1.215477 | -0.166083 | -0.033480 |
| | C | 0.852711 | 0.501217 | 0.149953 |
| | C | 1.669231 | -0.490382 | 0.203945 |
| | Cl | -1.147842 | -1.900597 | 0.315132 |
| | Cl | -1.853950 | 0.872832 | 1.255262 |
| | Cl | -1.489220 | 0.320443 | -1.713980 |
| | C | 0.914059 | 1.971110 | 0.078509 |
| | Cl | 3.384584 | -0.267035 | -0.031814 |
| | H | 1.402292 | -1.524351 | 0.369915 |
| | H | 0.661972 | 2.416974 | 1.043116 |
| | H | 0.254617 | 2.376681 | -0.690404 |
| | H | 1.951752 | 2.233522 | -0.167848 |
| IM | | x | y | z |
| | B | 0.930309 | -0.084168 | -0.033683 |
| | C | -0.589132 | 0.440176 | -0.109035 |
| | C | -1.617338 | -0.516870 | -0.013660 |
| | Cl | 1.131487 | -1.805678 | -0.648994 |
| | Cl | 2.100922 | 1.047405 | -0.870450 |
| | Cl | 1.081031 | -0.002145 | 1.834266 |
| | C | -0.904539 | 1.879019 | -0.221013 |
| | Cl | -3.235286 | -0.155759 | -0.139968 |
| | H | -1.399008 | -1.567922 | 0.141474 |
| | H | -0.956265 | 2.109076 | -1.298884 |
| | H | -0.097205 | 2.487419 | 0.188987 |
| | H | -1.861633 | 2.153326 | 0.226553 |
| $C_3H_4Br\cdot{}+BBr_3$ | | x | y | z |
| | B | -1.596538 | 0.160337 | 0.035287 |
| | C | 1.407236 | -0.754677 | -0.151085 |
| | C | 2.160842 | 0.278539 | -0.125651 |
| | C | 1.378506 | -2.218931 | -0.140567 |
| | H | 1.851428 | 1.315983 | -0.151227 |
| | H | 0.784456 | -2.587582 | 0.700374 |
| | H | 2.403017 | -2.604371 | -0.044840 |
| | H | 0.942923 | -2.603149 | -1.066973 |
| | Br | -1.216114 | 1.998905 | -0.264111 |
| | Br | 4.093119 | 0.118128 | -0.028902 |
| | Br | -2.029680 | -0.968205 | -1.432202 |
| | Br | -1.638143 | -0.524603 | 1.807788 |

| TS1 | x | y | z |
|---|---|---|---|
| B | -1.220310 | 0.112367 | 0.018955 |
| C | 1.043179 | -0.658041 | -0.084812 |
| C | 1.854261 | 0.333042 | -0.057595 |
| C | 1.042549 | -2.127570 | -0.123948 |
| H | 1.605651 | 1.385140 | -0.036688 |
| H | 0.355133 | -2.546946 | 0.614052 |
| H | 2.060345 | -2.465422 | 0.112111 |
| H | 0.766137 | -2.492596 | -1.115676 |
| Br | -1.044736 | 2.007147 | -0.260710 |
| Br | 3.750682 | 0.037874 | -0.047291 |
| Br | -1.850416 | -0.951008 | -1.455165 |
| Br | -1.493406 | -0.514773 | 1.818296 |

| IM | x | y | z |
|---|---|---|---|
| B | -0.872120 | 0.065141 | -0.134232 |
| C | 0.611633 | -0.444163 | -0.355078 |
| C | 1.646754 | 0.454499 | -0.104162 |
| C | 0.897321 | -1.804244 | -0.909791 |
| H | 1.470393 | 1.446960 | 0.294136 |
| H | 0.043918 | -2.470045 | -0.784445 |
| H | 1.785860 | -2.253166 | -0.459151 |
| H | 1.081524 | -1.692909 | -1.987992 |
| Br | -1.197436 | 2.010889 | -0.038421 |
| Br | 3.424133 | 0.079535 | -0.400564 |
| Br | -2.307412 | -0.914437 | -1.054357 |
| Br | -0.460866 | -0.735789 | 1.831136 |

| $C_2H_2I\cdot$+$BI_3$ | x | y | z |
|---|---|---|---|
| B | -1.602493 | 0.146379 | 0.066855 |
| C | 1.469222 | -0.866699 | -0.278097 |
| C | 2.200828 | 0.167767 | -0.163429 |
| C | 1.422210 | -2.322153 | -0.412336 |
| H | 1.900221 | 1.204787 | -0.085694 |
| H | 0.780281 | -2.761426 | 0.356514 |
| H | 2.436781 | -2.730876 | -0.301700 |
| H | 1.032566 | -2.605168 | -1.393958 |
| I | 4.371247 | -0.012466 | -0.094564 |
| I | -1.186007 | 2.227945 | -0.181986 |
| I | -2.174879 | -1.030678 | -1.622156 |
| I | -1.551700 | -0.726550 | 2.015947 |

| TS1 | x | y | z |
|---|---|---|---|
| B | -1.273465 | 0.095674 | 0.039072 |
| C | 1.145347 | -0.753904 | -0.180518 |
| C | 1.941560 | 0.233784 | -0.048289 |
| C | 1.126723 | -2.207314 | -0.390523 |
| H | 1.694898 | 1.276286 | 0.100741 |
| H | 0.394417 | -2.696705 | 0.256068 |
| H | 2.124049 | -2.598446 | -0.147996 |
| H | 0.894425 | -2.445266 | -1.431432 |
| I | 4.070869 | -0.068964 | -0.109491 |
| I | -1.046684 | 2.234668 | -0.117040 |
| I | -2.006193 | -0.968231 | -1.685497 |
| I | -1.471242 | -0.775769 | 2.001523 |

| IM | | x | y | z |
|---|---|---|---|---|
| | B | -0.837914 | 0.055045 | -0.153615 |
| | C | 0.640131 | -0.420741 | -0.500663 |
| | C | 1.681045 | 0.386066 | -0.095310 |
| | C | 0.882059 | -1.646624 | -1.334081 |
| | H | 1.518946 | 1.278851 | 0.498913 |
| | H | 0.057667 | -2.355656 | -1.252458 |
| | H | 1.814602 | -2.145442 | -1.058524 |
| | H | 0.953144 | -1.336806 | -2.385923 |
| | I | 3.680920 | 0.083963 | -0.541448 |
| | I | -1.181167 | 2.195274 | 0.335711 |
| | I | -2.489400 | -0.813006 | -1.337100 |
| | I | -0.375904 | -1.195068 | 1.855033 |

**Table S15.** Cartesian coordinates (in Å) for structures referring to Figure 15

| $IM+BCl_3$ | | x | y | z |
|---|---|---|---|---|
| | B | -1.296061 | -1.052098 | 0.069485 |
| | C | -1.450867 | 0.488947 | -0.399136 |
| | C | -1.399714 | 1.506651 | 0.584680 |
| | Cl | -2.354189 | -2.141008 | -0.931329 |
| | Cl | 0.543711 | -1.513474 | -0.421738 |
| | Cl | -1.532759 | -1.314084 | 1.849640 |
| | C | -1.731300 | 0.815818 | -1.809756 |
| | Cl | -1.473169 | 3.119035 | 0.248341 |
| | H | -1.323321 | 1.264421 | 1.638962 |
| | H | -1.272467 | 0.088427 | -2.482627 |
| | H | -1.445132 | 1.831786 | -2.085225 |
| | H | -2.822899 | 0.724404 | -1.946914 |
| | B | 1.911410 | -0.008964 | -0.037238 |
| | Cl | 1.604667 | 1.247100 | -1.303297 |
| | Cl | 1.581052 | 0.577817 | 1.637258 |
| | Cl | 3.470590 | -0.885517 | -0.228296 |
| TS2-Cl | | x | y | z |
| | B | -1.975948 | -0.677455 | 0.101292 |
| | C | -1.417610 | 0.728199 | -0.394171 |
| | C | -1.017944 | 1.655095 | 0.604012 |
| | Cl | -2.928680 | -1.624288 | -1.014613 |
| | Cl | 0.354055 | -1.423798 | -0.591342 |
| | Cl | -2.065127 | -1.039688 | 1.808173 |
| | C | -1.491161 | 1.129073 | -1.813175 |
| | Cl | -0.582310 | 3.208208 | 0.277990 |
| | H | -1.032883 | 1.396431 | 1.656466 |
| | H | -1.405307 | 0.267782 | -2.475817 |
| | H | -0.754332 | 1.888158 | -2.079313 |
| | H | -2.494520 | 1.563180 | -1.971695 |
| | B | 1.884320 | -0.410609 | -0.041384 |
| | Cl | 2.075796 | 0.989114 | -1.214251 |
| | Cl | 1.580170 | 0.213797 | 1.662737 |
| | Cl | 3.313478 | -1.543900 | -0.093940 |

| $E+BCl_4^-$ | | x | y | z |
|---|---|---|---|---|
| | B | -2.283184 | -0.468966 | 0.100308 |
| | C | -1.331222 | 0.692158 | -0.410494 |
| | C | -0.826931 | 1.538933 | 0.593319 |
| | Cl | -3.282652 | -1.305894 | -1.044933 |
| | Cl | 0.255220 | -1.144715 | -0.660325 |
| | Cl | -2.412193 | -0.854940 | 1.791111 |
| | C | -1.396300 | 1.174988 | -1.822451 |
| | Cl | -0.223897 | 3.056223 | 0.296787 |
| | H | -0.834900 | 1.258497 | 1.639552 |
| | H | -1.595879 | 0.357156 | -2.514119 |
| | H | -0.477889 | 1.685597 | -2.120483 |
| | H | -2.225991 | 1.893455 | -1.898840 |
| | B | 1.982550 | -0.488331 | -0.012267 |
| | Cl | 2.429390 | 0.933904 | -1.062333 |
| | Cl | 1.769551 | -0.010416 | 1.737729 |
| | Cl | 3.109554 | -1.900321 | -0.217363 |

| $IM+BBr_3$ | | x | y | z |
|---|---|---|---|---|
| | C | -0.795270 | -0.183923 | -1.738541 |
| | C | 0.021274 | 0.942542 | -1.663034 |
| | C | -0.571812 | 2.314864 | -1.619517 |
| | H | -0.881575 | 2.580255 | -2.640843 |
| | H | 0.160674 | 3.051809 | -1.291005 |
| | H | -1.454214 | 2.357723 | -0.975796 |
| | B | 1.587588 | 0.720429 | -1.765677 |
| | Br | 1.403177 | 0.687198 | 0.373718 |
| | Br | 2.247588 | -1.020793 | -2.432920 |
| | Br | 2.658095 | 2.244655 | -2.402544 |
| | Br | -2.623270 | -0.102499 | -1.893433 |
| | H | -0.398441 | -1.192554 | -1.740016 |
| | B | -1.164341 | -0.761907 | 2.364887 |
| | Br | 0.058111 | -0.688852 | 3.813916 |
| | Br | -2.307107 | 0.714101 | 2.011708 |
| | Br | -1.270755 | -2.327686 | 1.292243 |

| TS2-Br | | x | y | z |
|---|---|---|---|---|
| | C | -2.385501 | -2.683501 | -0.118041 |
| | C | -1.479067 | -1.638569 | 0.004557 |
| | C | -1.946836 | -0.213645 | -0.047474 |
| | H | -2.192613 | 0.032894 | -1.089581 |
| | H | -1.169532 | 0.471044 | 0.290174 |
| | H | -2.843965 | -0.067911 | 0.559336 |
| | B | 0.043229 | -2.013709 | -0.209492 |
| | Br | -0.529024 | -1.774584 | 2.345232 |
| | Br | 0.609044 | -3.829661 | -0.475896 |
| | Br | 1.298328 | -0.625783 | -0.611769 |
| | Br | -4.192703 | -2.451625 | -0.289614 |
| | H | -2.076612 | -3.722150 | -0.129894 |
| | B | -2.138782 | -3.148732 | 3.660071 |
| | Br | -1.153743 | -3.152088 | 5.332910 |
| | Br | -3.735712 | -2.047420 | 3.585997 |
| | Br | -2.191202 | -4.825388 | 2.676494 |

| $E+BBr_4^-$ | x | y | z |
|---|---|---|---|
| C | -0.890262 | -0.158091 | -1.788631 |
| C | 0.002579 | 0.893600 | -1.616293 |
| C | -0.498996 | 2.311141 | -1.573736 |
| H | -0.774086 | 2.625478 | -2.589887 |
| H | 0.269180 | 2.992162 | -1.206699 |
| H | -1.382801 | 2.397050 | -0.936537 |
| B | 1.488572 | 0.569445 | -2.038733 |
| Br | 0.908632 | 0.686049 | 0.777872 |
| Br | 2.101342 | -1.214966 | -2.331333 |
| Br | 2.689102 | 2.005739 | -2.378877 |
| Br | -2.697185 | 0.052605 | -1.928521 |
| H | -0.566782 | -1.188370 | -1.881102 |
| B | -0.633855 | -0.386161 | 1.869968 |
| Br | 0.181230 | -0.506130 | 3.670297 |
| Br | -2.256656 | 0.741692 | 1.809784 |
| Br | -0.862007 | -2.145568 | 0.989153 |
| $IM+BI_3$ | x | y | z |
| B | -2.401559 | -0.440871 | -0.378956 |
| C | -1.769121 | 0.915735 | -0.918823 |
| C | -0.954625 | 1.622013 | -0.061605 |
| C | -2.162876 | 1.470972 | -2.257992 |
| H | -0.651533 | 1.229338 | 0.902965 |
| H | -2.479226 | 0.681813 | -2.940985 |
| H | -1.346721 | 2.036300 | -2.715391 |
| H | -3.015162 | 2.149332 | -2.113619 |
| I | -0.211140 | 3.519493 | -0.412603 |
| I | -2.441413 | -0.797365 | 1.814170 |
| I | -4.229754 | -1.171810 | -1.383893 |
| I | -0.389449 | -1.343532 | -1.335413 |
| B | 2.928029 | -0.327035 | 0.687577 |
| I | 1.987480 | 0.464675 | 2.435088 |
| I | 3.456066 | -2.392570 | 0.611581 |
| I | 3.369955 | 0.951933 | -0.961721 |
| TS2 | x | y | z |
| B | -2.520516 | -0.250179 | -0.179196 |
| C | -1.687408 | 0.990928 | -0.698135 |
| C | -1.178440 | 1.829563 | 0.272004 |
| C | -1.680813 | 1.385832 | -2.147437 |
| H | -1.144004 | 1.546763 | 1.318424 |
| H | -1.842812 | 0.526570 | -2.798965 |
| H | -0.738745 | 1.869249 | -2.421389 |
| H | -2.498626 | 2.099134 | -2.321142 |
| I | -0.503668 | 3.737501 | -0.056376 |
| I | -2.969752 | -0.502070 | 1.953259 |
| I | -4.001948 | -1.122143 | -1.527678 |
| I | -0.262923 | -1.399785 | -0.690545 |
| B | 2.099662 | -0.405348 | 0.163181 |
| I | 1.688971 | 0.360011 | 2.169357 |
| I | 3.212823 | -2.290575 | 0.054005 |
| I | 2.569680 | 1.078728 | -1.372378 |

| $E+BI_4^{\cdot}$ | | x | y | z |
|---|---|---|---|---|
| | B | -2.527065 | -0.172494 | -0.158870 |
| | C | -1.594453 | 0.992825 | -0.693269 |
| | C | -1.075270 | 1.822968 | 0.278651 |
| | C | -1.547745 | 1.362871 | -2.146470 |
| | H | -1.065758 | 1.539555 | 1.326108 |
| | H | -1.691643 | 0.490525 | -2.784532 |
| | H | -0.597248 | 1.838522 | -2.402470 |
| | H | -2.360973 | 2.071885 | -2.357344 |
| | I | -0.357303 | 3.699029 | -0.046423 |
| | I | -2.935458 | -0.455775 | 1.947219 |
| | I | -3.856996 | -1.156055 | -1.542894 |
| | I | -0.025723 | -1.474531 | -0.706467 |
| | B | 1.974201 | -0.540276 | 0.020010 |
| | I | 1.696392 | 0.290959 | 2.079876 |
| | I | 3.360727 | -2.304350 | 0.004016 |
| | I | 2.595448 | 1.028372 | -1.449671 |

**Table S16.** Cartesian coordinates (in Å) for structures referring to Figure S1

| $C_2H_2+BBr_3$, MP2/SVP | | x | y | z |
|---|---|---|---|---|
| | B | -0.017288 | 0.000641 | -0.382102 |
| | C | -0.456824 | -0.005675 | 3.221183 |
| | C | 0.755156 | -0.005159 | 3.058727 |
| | Br | -1.916748 | 0.008766 | -0.339362 |
| | Br | 0.936818 | 1.641774 | -0.429015 |
| | Br | 0.922703 | -1.648464 | -0.433240 |
| | H | -1.517309 | -0.006196 | 3.357498 |
| | H | 1.816696 | -0.004680 | 2.930114 |
| $C_2H_2+BBr_3$, MP2/tzvpp | | x | y | z |
| | B | 0.015450 | -0.000269 | -0.369669 |
| | C | 0.472427 | 0.002404 | 3.109813 |
| | C | -0.732109 | 0.002136 | 2.964137 |
| | Br | 1.907447 | -0.004461 | -0.336603 |
| | Br | -0.932479 | -1.636920 | -0.411901 |
| | Br | -0.925218 | 1.640511 | -0.413611 |
| | H | 1.528241 | 0.002669 | 3.238417 |
| | H | -1.788623 | 0.001895 | 2.840275 |
| $C_2H_2+BBr_3$, B3LYP | | x | y | z |
| | B | 0.001811 | 0.001691 | -0.375190 |
| | C | -0.613506 | -0.015699 | 3.142782 |
| | C | 0.589329 | -0.013835 | 3.023368 |
| | Br | -1.903737 | 0.121201 | -0.408274 |
| | Br | 1.057362 | 1.591990 | -0.378450 |
| | Br | 0.850983 | -1.707506 | -0.392602 |
| | H | -1.677390 | -0.017400 | 3.241538 |
| | H | 1.652119 | -0.012818 | 2.913955 |

| TS-Br, MP2/SVP | x | y | z |
|---|---|---|---|
| B | -0.099771 | 0.000013 | 0.315346 |
| C | 1.433060 | -0.000270 | 1.964191 |
| C | 0.170981 | -0.000024 | 1.951840 |
| Br | 1.800485 | -0.000527 | -0.539890 |
| Br | -1.057804 | -1.667275 | -0.157164 |
| Br | -1.056804 | 1.667858 | -0.157211 |
| H | 2.493696 | -0.000440 | 2.138876 |
| H | -0.624764 | 0.000156 | 2.687484 |

| TS-Br, MP2/tzvpp | x | y | z |
|---|---|---|---|
| B | -0.093652 | 0.000001 | 0.306035 |
| C | 1.389911 | -0.000014 | 1.932488 |
| C | 0.139466 | -0.000001 | 1.962085 |
| Br | 1.791222 | -0.000041 | -0.522943 |
| Br | -1.045321 | -1.659719 | -0.162070 |
| Br | -1.045247 | 1.659762 | -0.162075 |
| H | 2.447135 | -0.000021 | 2.090251 |
| H | -0.678019 | 0.000010 | 2.660221 |

| TS-Br, B3LYP | x | y | z |
|---|---|---|---|
| B | -0.068178 | -0.000001 | 0.290121 |
| C | 1.291619 | 0.000055 | 2.178810 |
| C | 0.054059 | -0.000002 | 1.985256 |
| Br | 1.825408 | 0.000134 | -0.532251 |
| Br | -1.044660 | -1.677643 | -0.181714 |
| Br | -1.044908 | 1.677498 | -0.181701 |
| H | 2.354539 | 0.000103 | 2.322231 |
| H | -0.842126 | -0.000043 | 2.591085 |

| Z-Br, MP2/SVP | x | y | z |
|---|---|---|---|
| B | -0.794768 | -0.215223 | 0.210125 |
| C | 1.480151 | -1.518341 | 0.508128 |
| C | 0.140667 | -1.369437 | 0.619700 |
| Br | 2.701922 | -0.285782 | -0.223440 |
| Br | -2.613248 | -0.676198 | -0.186610 |
| Br | -0.299661 | 1.621672 | 0.133952 |
| H | 1.981933 | -2.429564 | 0.819398 |
| H | -0.348468 | -2.256872 | 1.026440 |

| Z-Br, MP2/tzvpp | x | y | z |
|---|---|---|---|
| B | 0.668350 | -0.000011 | 0.510849 |
| C | -1.736075 | 0.002354 | 1.340370 |
| C | -0.428105 | 0.001705 | 1.602725 |
| Br | -2.380445 | 0.001216 | -0.427572 |
| Br | 1.368038 | 1.637537 | -0.142966 |
| Br | 1.363857 | -1.639627 | -0.142232 |
| H | -2.517834 | 0.003652 | 2.083986 |
| H | -0.139563 | 0.002629 | 2.650169 |

| Z-Br, B3LYP | x | y | z |
|---|---|---|---|
| B | -0.806076 | -0.256039 | 0.113425 |
| C | 1.450923 | -1.620085 | 0.295542 |
| C | 0.113334 | -1.465788 | 0.327245 |
| Br | 2.771780 | -0.314272 | -0.046099 |
| Br | -0.245223 | 1.524122 | -0.322899 |
| Br | -2.706350 | -0.558487 | 0.302534 |
| H | 1.915314 | -2.582686 | 0.479404 |
| H | -0.401311 | -2.401486 | 0.553659 |

| $C_2H_2$+$BCl_3$, MP2/6-31+G* | x | y | z |
|---|---|---|---|
| C | -2.498380 | -0.305109 | -0.096607 |
| C | -1.368128 | -0.725936 | 0.098536 |
| H | -3.487576 | 0.064317 | -0.265676 |
| H | -0.377271 | -1.094219 | 0.260861 |
| B | -2.418821 | -0.600629 | 3.400062 |
| Cl | -1.191000 | 0.616255 | 3.582516 |
| Cl | -4.088038 | -0.135812 | 3.253605 |
| Cl | -1.984237 | -2.283886 | 3.415893 |
| $C_2H_2$+$BCl_3$, MP2/tzvpp[a] | x | y | z |
| B | -0.655470 | -0.004956 | 0.004275 |
| C | 2.723200 | 0.625562 | -0.016818 |
| C | 2.653004 | -0.585576 | -0.016746 |
| Cl | -0.709396 | 1.724526 | -0.164590 |
| Cl | -0.668344 | -1.016010 | -1.409568 |
| Cl | -0.643460 | -0.723503 | 1.586680 |
| H | 2.785175 | 1.687370 | -0.016591 |
| H | 2.595365 | -1.647749 | -0.016274 |
| $C_2H_2$+$BCl_3$, MP2/tzvpp[b] | x | y | z |
| B | 0.655342 | -0.008512 | 0.001038 |
| C | -2.720682 | 0.636973 | -0.000527 |
| C | -2.655833 | -0.574458 | -0.003354 |
| Cl | 0.715452 | 1.717840 | 0.197535 |
| Cl | 0.653464 | -1.041766 | 1.398829 |
| Cl | 0.651803 | -0.702028 | -1.592526 |
| H | -2.777933 | 1.699044 | 0.001634 |
| H | -2.602962 | -1.636874 | -0.006260 |
| $C_2H_2$+$BCl_3$, B3LYP | x | y | z |
| C | -2.470457 | -0.713269 | -0.036001 |
| C | -1.332210 | -0.327959 | 0.093111 |
| H | -3.477452 | -1.053935 | -0.146227 |
| H | -0.326175 | 0.012650 | 0.211418 |
| B | -2.449399 | -0.595271 | 3.366318 |
| Cl | -0.803199 | -0.084247 | 3.687044 |
| Cl | -3.720018 | 0.599903 | 3.196083 |
| Cl | -2.834541 | -2.302892 | 3.277444 |
| TS-Cl, MP2/6-31+G* | x | y | z |
| B | -0.176089 | -0.000001 | 0.117419 |
| C | 1.773589 | -0.000003 | 1.208495 |
| C | 0.572814 | -0.000003 | 1.599309 |
| Cl | 1.322931 | 0.000015 | -1.085374 |
| Cl | -1.135031 | -1.523400 | -0.076672 |
| Cl | -1.135050 | 1.523389 | -0.076657 |
| H | 2.839982 | -0.000002 | 1.068763 |
| H | 0.063607 | -0.000025 | 2.555266 |
| TS-Cl, MP2/tzvpp* | x | y | z |
| B | -0.174899 | -0.000001 | 0.111817 |
| C | 1.727984 | -0.000018 | 1.218258 |
| C | 0.543326 | -0.000021 | 1.625059 |
| Cl | 1.337930 | 0.000016 | -1.066999 |
| Cl | -1.125915 | -1.524299 | -0.091016 |
| Cl | -1.125915 | 1.524301 | -0.090985 |
| H | 2.786104 | -0.000016 | 1.063345 |
| H | -0.003179 | -0.000057 | 2.550676 |

| TS-Cl, B3LYP | x | y | z |
|---|---|---|---|
| B | -0.168403 | -0.000001 | 0.108546 |
| C | 1.815882 | -0.000011 | 1.294055 |
| C | 0.591146 | -0.000010 | 1.594914 |
| Cl | 1.316648 | -0.000002 | -1.139660 |
| Cl | -1.144669 | -1.538342 | -0.063578 |
| Cl | -1.144650 | 1.538354 | -0.063570 |
| H | 2.875374 | -0.000014 | 1.119184 |
| H | 0.059881 | -0.000026 | 2.540013 |

| Z-Cl, MP2/6-31+G* | x | y | z |
|---|---|---|---|
| B | -0.849461 | -0.102740 | 0.067706 |
| C | 1.524028 | -1.242640 | 0.144676 |
| C | 0.174881 | -1.243604 | 0.219359 |
| Cl | 2.536421 | 0.117487 | -0.161059 |
| Cl | -2.535008 | -0.549811 | -0.142477 |
| Cl | -0.460987 | 1.598487 | 0.116856 |
| H | 2.102061 | -2.154503 | 0.260161 |
| H | -0.235455 | -2.239102 | 0.390666 |

| Z-Cl, MP2/tzvpp* | x | y | z |
|---|---|---|---|
| B | -0.844888 | -0.106734 | 0.000017 |
| C | 1.513969 | -1.256857 | 0.000503 |
| C | 0.168328 | -1.260778 | 0.000371 |
| Cl | 2.532974 | 0.119101 | -0.000420 |
| Cl | -2.550303 | -0.527166 | -0.000313 |
| Cl | -0.435750 | 1.589270 | 0.000316 |
| H | 2.079130 | -2.178056 | 0.001037 |
| H | -0.246119 | -2.262940 | 0.000717 |

| Z-Cl, B3LYP | x | y | z |
|---|---|---|---|
| B | -0.848530 | -0.110379 | 0.000369 |
| C | 1.515611 | -1.266088 | 0.000787 |
| C | 0.170126 | -1.259481 | 0.001179 |
| Cl | 2.566811 | 0.116308 | -0.000876 |
| Cl | -2.572138 | -0.537023 | -0.000776 |
| Cl | -0.447899 | 1.606539 | 0.000643 |
| H | 2.085927 | -2.189180 | 0.001413 |
| H | -0.252844 | -2.264516 | 0.002090 |

| $C_2H_2$+$BI_3$, MP2/SVP | x | y | z |
|---|---|---|---|
| B | 0.006023 | -0.000937 | -0.278725 |
| C | 0.524056 | 0.011831 | 3.508850 |
| C | -0.685420 | 0.012381 | 3.328245 |
| I | 2.141094 | -0.034165 | -0.275925 |
| I | -1.088944 | -1.833268 | -0.297458 |
| I | -1.031391 | 1.864320 | -0.303437 |
| H | 1.583346 | 0.011597 | 3.653931 |
| H | -1.745473 | 0.012765 | 3.188581 |

| $C_2H_2$+$BI_3$, MP2/tzvpp | x | y | z |
|---|---|---|---|
| B | 0.003225 | -0.000126 | -0.266650 |
| C | 0.555413 | 0.001882 | 3.344841 |
| C | -0.648256 | 0.001587 | 3.191109 |
| I | -1.039223 | 1.819370 | -0.283021 |
| I | 2.100589 | -0.006032 | -0.273017 |
| I | -1.049393 | -1.813786 | -0.282153 |
| H | 1.610513 | 0.002203 | 3.479009 |
| H | -1.704165 | 0.001301 | 3.062639 |

| $C_2H_2$+$BI_3$, B3LYP/SVP | x | y | z |
|---|---|---|---|
| B | 0.000212 | -0.000048 | -0.267240 |
| C | 0.602609 | 0.000533 | 3.361329 |
| C | -0.597076 | 0.000254 | 3.213633 |
| I | 2.139977 | 0.001106 | -0.288879 |
| I | -1.069382 | -1.852863 | -0.276915 |
| I | -1.071380 | 1.851657 | -0.277111 |
| H | 1.664102 | 0.000814 | 3.481918 |
| H | -1.656782 | -0.000021 | 3.078494 |

| $C_2H_2$+$BI_3$, B3LYP/ECP46MWB | x | y | z |
|---|---|---|---|
| B | -0.047909 | 0.001143 | -0.207421 |
| C | 0.351298 | 0.010879 | 3.462146 |
| C | 1.476578 | -0.030935 | 3.022822 |
| I | 1.053606 | -1.802810 | -0.529642 |
| I | -2.114056 | -0.090994 | 0.335413 |
| I | 0.903826 | 1.896783 | -0.471189 |
| H | -0.646761 | 0.048381 | 3.841600 |
| H | 2.469434 | -0.068131 | 2.629991 |

| TS-I, MP2/SVP | x | y | z |
|---|---|---|---|
| B | -0.000086 | -0.023268 | 0.275256 |
| C | 0.001081 | 0.585725 | 2.635245 |
| C | -0.001467 | -0.615424 | 2.369043 |
| I | -1.875207 | -1.055110 | -0.227462 |
| I | 0.005511 | 2.126199 | -0.236081 |
| I | 1.869756 | -1.064667 | -0.227395 |
| H | 0.003384 | 1.640728 | 2.816444 |
| H | -0.003808 | -1.686578 | 2.401257 |

| TS-I, MP2/tzvpp | x | y | z |
|---|---|---|---|
| B | -0.000375 | -0.022943 | 0.315527 |
| C | 0.006349 | 0.561532 | 2.528722 |
| C | -0.008127 | -0.641264 | 2.305059 |
| I | -1.851502 | -1.014614 | -0.224484 |
| I | 0.025644 | 2.086910 | -0.223315 |
| I | 1.826134 | -1.059247 | -0.224323 |
| H | 0.018971 | 1.608766 | 2.722705 |
| H | -0.021065 | -1.707261 | 2.319433 |

| TS-I, B3LYP/SVP | x | y | z |
|---|---|---|---|
| B | 0.004054 | -0.024382 | 0.282039 |
| C | -0.077441 | 0.654467 | 2.656594 |
| C | 0.068532 | -0.518505 | 2.351311 |
| I | -1.718475 | -1.308726 | -0.217188 |
| I | -0.282022 | 2.108799 | -0.256254 |
| I | 2.001210 | -0.815446 | -0.219209 |
| H | -0.206511 | 1.699727 | 2.840555 |
| H | 0.201918 | -1.578877 | 2.412316 |

| TS-I, B3LYP/ECP46MWB | x | y | z |
|---|---|---|---|
| B | 0.003717 | -0.020346 | 0.265982 |
| C | -0.079795 | 0.651935 | 2.665746 |
| C | 0.071573 | -0.520948 | 2.367430 |
| I | -1.722551 | -1.305327 | -0.217092 |
| I | -0.276878 | 2.113553 | -0.272256 |
| I | 2.000976 | -0.817246 | -0.224179 |
| H | -0.214377 | 1.696875 | 2.847588 |
| H | 0.208604 | -1.581437 | 2.416951 |

| IM, MP2/SVP | x | y | z |
|---|---|---|---|
| B | -0.000014 | -0.032896 | 0.422512 |
| C | -0.000015 | 0.630895 | 2.497686 |
| C | -0.000069 | -0.576895 | 2.245845 |
| I | -1.874948 | -1.058438 | -0.217382 |
| I | 0.000291 | 2.113635 | -0.237978 |
| I | 1.874670 | -1.058928 | -0.217363 |
| H | 0.000027 | 1.681561 | 2.707754 |
| H | -0.000138 | -1.643361 | 2.372823 |

| IM, MP2/tzvpp | x | y | z |
|---|---|---|---|
| B | 0.000023 | -0.024867 | 0.349390 |
| C | 0.000096 | 0.573103 | 2.497448 |
| C | -0.000003 | -0.631465 | 2.277059 |
| I | -1.838954 | -1.036800 | -0.221954 |
| I | -0.000229 | 2.083647 | -0.224075 |
| I | 1.839168 | -1.036430 | -0.221975 |
| H | 0.000216 | 1.619807 | 2.695927 |
| H | -0.000103 | -1.697413 | 2.314296 |

| TS2, MP2/SVP | x | y | z |
|---|---|---|---|
| B | -0.084223 | -0.000117 | 0.459058 |
| C | 1.184297 | 0.002155 | 2.289154 |
| C | -0.063848 | -0.000194 | 2.117769 |
| I | -1.118731 | 1.876152 | -0.155239 |
| I | 2.088417 | 0.003667 | -0.331867 |
| I | -1.112059 | -1.880072 | -0.155237 |
| H | 2.213895 | 0.004186 | 2.595742 |
| H | -0.969692 | -0.001946 | 2.711613 |

| TS2, MP2/tzvpp | x | y | z |
|---|---|---|---|
| B | -0.004117 | 0.081313 | 0.445334 |
| C | 0.056443 | -1.149953 | 2.214948 |
| C | -0.005097 | 0.094770 | 2.117609 |
| I | 1.787612 | 1.178572 | -0.155203 |
| I | 0.102471 | -2.038701 | -0.319560 |
| I | -1.896564 | 0.993990 | -0.155522 |
| H | 0.106262 | -2.177806 | 2.500901 |
| H | -0.050234 | 1.007686 | 2.682189 |

| Z-I, MP2/SVP | x | y | z |
|---|---|---|---|
| B | 0.668804 | 0.006050 | 0.613564 |
| C | -1.712452 | -0.116644 | 1.603030 |
| C | -0.377649 | -0.072470 | 1.754258 |
| I | 1.551724 | -1.795193 | -0.126747 |
| I | -2.642695 | -0.092431 | -0.301311 |
| I | 1.310215 | 1.913643 | -0.108140 |
| H | -2.419696 | -0.175127 | 2.423973 |
| H | -0.003647 | -0.099487 | 2.783014 |

| Z-I, MP2/tzvpp | x | y | z |
|---|---|---|---|
| B | 0.654046 | 0.000010 | 0.621405 |
| C | -1.710679 | -0.000155 | 1.587280 |
| C | -0.385383 | -0.000093 | 1.763964 |
| I | 1.387891 | -1.823866 | -0.115581 |
| I | -2.553466 | -0.000125 | -0.304628 |
| I | 1.387569 | 1.824025 | -0.115557 |
| H | -2.433378 | -0.000231 | 2.389562 |
| H | -0.026149 | -0.000126 | 2.791582 |

| Z-I, B3LYP/SVP | x | y | z |
| --- | --- | --- | --- |
| B | 0.096007 | -0.711576 | 0.599559 |
| C | -0.198963 | 1.539819 | 1.814636 |
| C | -0.023767 | 0.216879 | 1.830627 |
| I | -1.660914 | -1.634681 | -0.215614 |
| I | -0.351767 | 2.653625 | 0.010107 |
| I | 2.028130 | -1.141666 | -0.228426 |
| H | -0.279338 | 2.164814 | 2.696142 |
| H | 0.040972 | -0.253013 | 2.817068 |
| Z-I, B3LYP/ECP46MWB | x | y | z |
| B | 0.859530 | 0.001665 | 0.375911 |
| C | -0.995322 | -0.000531 | 2.166580 |
| C | 0.289010 | 0.000940 | 1.810518 |
| I | 1.300931 | -1.855514 | -0.600498 |
| I | -2.592731 | -0.002370 | 0.745693 |
| I | 1.292379 | 1.859832 | -0.602426 |
| H | -1.360101 | -0.001145 | 3.186786 |
| H | 1.015321 | 0.001480 | 2.629907 |

**Table S17.** Cartesian coordinates (in Å) for structures referring to Figure S3

| $C_2H_2$+$BBr_3$+$Cl^-$, MP2 | x | y | z |
| --- | --- | --- | --- |
| B | 1.321404 | -0.480864 | 0.029178 |
| C | -1.089702 | 1.885731 | -0.098651 |
| C | -0.149311 | 2.667391 | -0.141258 |
| Cl | 0.170486 | -1.395846 | -0.892082 |
| Cl | 2.636735 | 0.328862 | -0.777521 |
| Cl | 1.208565 | -0.424744 | 1.763292 |
| H | -1.935241 | 1.216799 | -0.063815 |
| H | 0.682136 | 3.338776 | -0.173645 |
| Cl | -3.893424 | -0.241801 | -0.003629 |
| $C_2H_2$+$BCl_3$+$Cl^-$, B3LYP | x | y | z |
| B | 1.381772 | -0.457804 | 0.000001 |
| C | -1.183663 | 1.817862 | 0.000037 |
| C | -0.197975 | 2.520787 | -0.000045 |
| Cl | 2.094246 | 0.064937 | 1.517042 |
| Cl | 0.021839 | -1.556357 | 0.000840 |
| Cl | 2.093128 | 0.064023 | -1.517868 |
| H | -2.061341 | 1.187245 | 0.000081 |
| H | 0.673841 | 3.138520 | -0.000099 |
| Cl | -4.046362 | -0.223700 | -0.000010 |
| TS1-Cl | x | y | z |
| B | -1.054225 | -0.000073 | -0.032043 |
| C | 0.976969 | 0.003347 | 1.219659 |
| C | -0.173982 | 0.004162 | 1.655272 |
| Cl | -2.030451 | 1.543534 | 0.120977 |
| Cl | 0.016334 | -0.013650 | -1.519240 |
| Cl | -2.044399 | -1.532989 | 0.138705 |
| H | 1.964741 | -0.000326 | 0.735237 |
| H | -0.896947 | 0.009062 | 2.449198 |
| Cl | 4.022364 | -0.000038 | 0.066982 |

| IM1-Cl | x | y | z |
|---|---|---|---|
| B | 1.053217 | 0.000007 | 0.013315 |
| C | -0.995721 | -0.000357 | -1.195435 |
| C | 0.165458 | -0.000504 | -1.613605 |
| Cl | 2.046776 | 1.538715 | -0.137961 |
| Cl | -0.006534 | 0.001136 | 1.517028 |
| Cl | 2.045772 | -1.539497 | -0.136410 |
| H | -1.990489 | -0.000121 | -0.721223 |
| H | 0.854104 | -0.000796 | -2.439195 |
| Cl | -4.035903 | 0.000002 | -0.069240 |
| TS2-Cl, MP2 | x | y | z |
| B | 1.112909 | 0.011257 | -0.000001 |
| C | -1.243265 | 0.028483 | -0.000347 |
| C | -0.567121 | -1.000597 | -0.000268 |
| Cl | 1.033386 | 1.819778 | 0.001343 |
| Cl | 1.809567 | -0.664723 | 1.523285 |
| Cl | 1.809136 | -0.662554 | -1.524423 |
| H | -1.664572 | 1.010663 | -0.000388 |
| H | -0.347450 | -2.050296 | -0.000355 |
| Cl | -4.222102 | -0.091558 | 0.000056 |
| TS2-Cl, B3LYP | x | y | z |
| B | 1.108540 | -0.000002 | -0.031216 |
| C | -1.229760 | -0.000028 | -0.549026 |
| C | -0.182124 | 0.000022 | -1.222143 |
| Cl | 0.506089 | -0.000148 | 1.729490 |
| Cl | 2.051765 | -1.539830 | -0.418445 |
| Cl | 2.051602 | 1.539979 | -0.418229 |
| H | -2.127263 | -0.000061 | 0.070031 |
| H | 0.146030 | 0.000079 | -2.250672 |
| Cl | -4.320643 | 0.000000 | -0.130243 |
| IM2-Cl, MP2 | x | y | z |
| B | -0.789197 | -0.041658 | -0.001084 |
| C | 1.784614 | 0.329583 | 0.002934 |
| C | 0.733080 | -0.497026 | -0.014818 |
| Cl | -1.627504 | -0.806267 | 1.495873 |
| Cl | -1.001698 | 1.809772 | 0.064866 |
| Cl | -1.629674 | -0.696443 | -1.548758 |
| H | 1.718570 | 1.410766 | 0.025239 |
| H | 0.927676 | -1.571435 | -0.037718 |
| Cl | 3.446733 | -0.226262 | -0.006735 |
| IM2-Cl, B3LYP | x | y | z |
| B | 0.779276 | 0.042855 | -0.000708 |
| C | -1.787686 | -0.333568 | 0.003544 |
| C | -0.744900 | 0.491922 | -0.007806 |
| Cl | 1.017799 | -1.832319 | 0.016670 |
| Cl | 1.642298 | 0.749503 | -1.547781 |
| Cl | 1.634834 | 0.778632 | 1.536956 |
| H | -1.731962 | -1.414565 | 0.016485 |
| H | -0.936510 | 1.565796 | -0.022893 |
| Cl | -3.473306 | 0.226794 | -0.003756 |

| $C_2H_2$+$BBr_3$+$Br^-$, MP2/SVP | x | y | z |
|---|---|---|---|
| B | 1.581872 | 0.000029 | 0.567142 |
| C | -1.156825 | 0.000205 | -1.791446 |
| C | -0.116398 | -0.000064 | -2.437880 |
| Br | 0.151153 | -0.000123 | 1.809896 |
| Br | 2.326749 | -1.644624 | -0.029450 |
| Br | 2.326685 | 1.644760 | -0.029284 |
| H | -2.087578 | 0.000326 | -1.243889 |
| H | 0.790029 | -0.000192 | -3.004749 |
| Br | -4.350284 | -0.000099 | -0.103469 |

| $C_2H_2$+$BBr_3$+$Br^-$, MP2/tzvpp | x | y | z |
|---|---|---|---|
| B | -1.413783 | 0.000082 | -0.329095 |
| C | 1.148547 | -0.000385 | 2.007143 |
| C | 0.132988 | -0.000837 | 2.675409 |
| Br | 0.070106 | -0.000851 | -1.494973 |
| Br | -2.183498 | -1.637322 | 0.233655 |
| Br | -2.181857 | 1.638435 | 0.233114 |
| H | 2.054568 | -0.000017 | 1.427161 |
| H | -0.754732 | -0.001290 | 3.260962 |
| Br | 4.240388 | -0.000027 | 0.138548 |

| TS1-Br | x | y | z |
|---|---|---|---|
| B | -0.005131 | 0.000160 | 0.035574 |
| C | 0.012647 | 0.000246 | 2.483273 |
| C | 1.111374 | 0.000062 | 1.928593 |
| Br | -1.955383 | 0.000718 | 0.113758 |
| Br | 0.786137 | 1.663027 | -0.621326 |
| Br | 0.785223 | -1.663249 | -0.621044 |
| H | -0.951595 | 0.000471 | 2.988298 |
| H | 2.146924 | -0.000137 | 1.656280 |
| Br | -2.718020 | 0.001188 | 4.589464 |

| IM1-Br | x | y | z |
|---|---|---|---|
| B | 1.016232 | -0.000160 | -0.112445 |
| C | -0.899333 | -0.000686 | -1.198473 |
| C | 0.156513 | -0.001499 | -1.836812 |
| Br | -0.005040 | 0.006177 | 1.592045 |
| Br | 2.074331 | -1.668395 | -0.322800 |
| Br | 2.081459 | 1.662702 | -0.330320 |
| H | -1.855371 | -0.000179 | -0.667086 |
| H | 0.878647 | -0.002806 | -2.630037 |
| Br | -4.140679 | -0.000002 | -0.308323 |

| TS2-Br, MP2/SVP | x | y | z |
|---|---|---|---|
| B | -0.020785 | 0.000178 | 0.084157 |
| C | -0.078709 | 0.000221 | 2.332037 |
| C | 0.948464 | 0.001579 | 1.641045 |
| Br | -2.020118 | 0.000408 | 0.127704 |
| Br | 0.673595 | 1.665903 | -0.761178 |
| Br | 0.673398 | -1.666733 | -0.758800 |
| H | -1.019732 | -0.001209 | 2.848805 |
| H | 2.009559 | 0.002742 | 1.472688 |
| Br | -0.893111 | -0.003480 | 5.336344 |

| TS2-Br, MP2/tzvpp | x | y | z |
|---|---|---|---|
| B | 1.020344 | 0.000646 | -0.054820 |
| C | -1.209114 | -0.002791 | -0.310449 |
| C | -0.451098 | 0.011934 | -1.275412 |
| Br | 0.723875 | -0.039459 | 1.900487 |
| Br | 1.913875 | -1.638097 | -0.698494 |
| Br | 1.890078 | 1.674959 | -0.635577 |
| H | -1.813683 | -0.015573 | 0.567826 |
| H | -0.095370 | 0.027339 | -2.280980 |
| Br | -4.334438 | 0.000602 | -0.237776 |
| IM2-Br, MP2/SVP | x | y | z |
| B | 0.689872 | 0.029860 | -0.042849 |
| C | -1.871878 | -0.206476 | 0.287790 |
| C | -0.833266 | 0.279489 | -0.404471 |
| Br | 0.963111 | -1.137781 | 1.598658 |
| Br | 1.593307 | -0.863309 | -1.651202 |
| Br | 1.580720 | 1.848154 | 0.275826 |
| H | -1.796360 | -0.827837 | 1.171985 |
| H | -1.037503 | 0.896801 | -1.281660 |
| Br | -3.690985 | 0.134184 | -0.194024 |
| IM2-Br, MP2/tzvpp | x | y | z |
| B | -0.686650 | -0.000639 | 0.053681 |
| C | 1.867420 | 0.005249 | -0.341616 |
| C | 0.829985 | -0.006185 | 0.494432 |
| Br | -0.942020 | 0.034823 | -1.950430 |
| Br | -1.582920 | -1.668046 | 0.806221 |
| Br | -1.588120 | 1.636294 | 0.865371 |
| Br | 3.668361 | -0.002781 | 0.240835 |
| H | 1.032228 | -0.021351 | 1.561490 |
| H | 1.781060 | 0.020034 | -1.416663 |
| $C_2H_2$+$BI_3$+$I^-$, MP2/SVP | x | y | z |
| B | -1.537748 | -0.000383 | 0.308766 |
| C | 1.211479 | -0.000055 | -2.253245 |
| C | 0.162619 | 0.007330 | -2.884987 |
| I | 0.150051 | -0.001349 | 1.605263 |
| I | -2.403672 | 1.848160 | -0.326083 |
| I | -2.403721 | -1.847927 | -0.328975 |
| H | 2.134103 | -0.004492 | -1.699029 |
| H | -0.745777 | 0.012302 | -3.448597 |
| I | 4.620660 | 0.000182 | -0.300522 |
| $C_2H_2$+$BI_3$+$I^-$, MP2/tzvpp | x | y | z |
| B | 1.409116 | 0.000019 | -0.324040 |
| C | -0.962903 | -0.000004 | 2.405611 |
| C | 0.096805 | -0.000506 | 3.000544 |
| I | -0.445382 | -0.000685 | -1.277684 |
| I | 2.349198 | 1.815554 | 0.169060 |
| I | 2.350399 | -1.814780 | 0.169486 |
| H | -1.895560 | 0.000416 | 1.877469 |
| H | 1.023960 | -0.001000 | 3.521613 |
| I | -4.272656 | -0.000022 | 0.255822 |

| TS1-I, MP2/SVP | x | y | z |
|---|---|---|---|
| B | -1.212438 | 0.002642 | -0.033398 |
| C | 0.883422 | 0.006040 | -1.507577 |
| C | -0.188336 | 0.046005 | -2.111098 |
| I | 0.095764 | -0.166776 | 1.710948 |
| I | -2.186028 | 1.943712 | -0.301143 |
| I | -2.388667 | -1.785197 | -0.497061 |
| H | 1.833307 | -0.017980 | -0.991223 |
| H | -0.993878 | 0.087050 | -2.814833 |
| I | 4.498786 | 0.000816 | -0.428120 |
| **TS1-I, MP2/tzvpp** | **x** | **y** | **z** |
| B | -1.155600 | 0.000002 | -0.050679 |
| C | 0.787332 | -0.000049 | -1.560828 |
| C | -0.290768 | 0.000138 | -2.139333 |
| I | 0.204576 | -0.000517 | 1.613939 |
| I | -2.241559 | 1.836859 | -0.345415 |
| I | -2.242257 | -1.836351 | -0.345985 |
| H | 1.749881 | -0.000171 | -1.071729 |
| H | -1.142482 | 0.000291 | -2.778302 |
| I | 4.320583 | -0.000004 | -0.426229 |
| **IM1-I, MP2/SVP** | **x** | **y** | **z** |
| B | -1.019446 | 0.003335 | -0.222667 |
| C | 0.878057 | -0.010581 | -1.174408 |
| C | -0.170674 | 0.021026 | -1.832494 |
| I | 0.045630 | -0.098286 | 1.758335 |
| I | -2.178528 | 1.919403 | -0.380579 |
| I | -2.296772 | -1.823424 | -0.498398 |
| H | 1.838333 | -0.026739 | -0.661136 |
| H | -0.813749 | 0.052018 | -2.692907 |
| I | 4.426432 | 0.000333 | -0.454664 |
| **IM1-I, MP2/tzvpp** | **x** | **y** | **z** |
| B | -1.009815 | 0.000924 | -0.207053 |
| C | 0.786084 | -0.000688 | -1.316479 |
| C | -0.275529 | 0.006058 | -1.939606 |
| I | 0.169684 | -0.029002 | 1.643852 |
| I | -2.184400 | 1.851127 | -0.354821 |
| I | -2.221371 | -1.822954 | -0.387478 |
| H | 1.763936 | -0.004199 | -0.840065 |
| H | -1.018070 | 0.013724 | -2.706323 |
| I | 4.259482 | -0.000045 | -0.446494 |
| **TS2-I, MP2/SVP** | **X** | **y** | **z** |
| B | 0.983512 | -0.000023 | -0.158726 |
| C | -1.160037 | 0.000493 | -0.570641 |
| C | -0.260320 | -0.000219 | -1.426817 |
| I | 0.449609 | 0.001386 | 2.036267 |
| I | 2.108738 | -1.873898 | -0.673805 |
| I | 2.110151 | 1.872453 | -0.675851 |
| H | -1.914835 | 0.001018 | 0.195540 |
| H | 0.094946 | -0.000834 | -2.442469 |
| I | -4.566149 | 0.000027 | -0.403115 |

| TS2-I, MP2/tzvpp | X | y | z |
|---|---|---|---|
| B | -0.974320 | 0.001288 | -0.125137 |
| C | 1.164910 | -0.018542 | -0.451404 |
| C | 0.354220 | 0.015961 | -1.379441 |
| I | -0.564222 | -0.072339 | 2.039554 |
| I | -1.994975 | 1.873310 | -0.654020 |
| I | -2.057497 | -1.800032 | -0.764723 |
| H | 1.853445 | -0.047469 | 0.365453 |
| H | 0.004240 | 0.048977 | -2.388282 |
| I | 4.501583 | -0.000797 | -0.363573 |

| IM2-I, MP2/SVP | x | y | z |
|---|---|---|---|
| B | -0.656324 | 0.004797 | -0.043162 |
| C | 1.908753 | -0.046326 | 0.401464 |
| C | 0.871713 | 0.048699 | -0.445815 |
| I | -1.028719 | -0.248275 | 2.184835 |
| I | -1.606882 | 1.965271 | -0.758679 |
| I | -1.613735 | -1.742653 | -1.178321 |
| H | 1.821122 | -0.166738 | 1.475338 |
| H | 1.079151 | 0.168266 | -1.511636 |
| I | 3.941762 | 0.024907 | -0.238058 |

| IM2-I, MP2/tzvpp | x | y | z |
|---|---|---|---|
| B | -0.653695 | 0.002023 | -0.047944 |
| C | 1.902119 | -0.019411 | 0.379648 |
| C | 0.866090 | 0.019911 | -0.463243 |
| I | -0.990280 | -0.098044 | 2.151014 |
| I | -1.598226 | 1.870207 | -0.862178 |
| I | -1.604225 | -1.781863 | -1.027840 |
| H | 1.801014 | -0.069427 | 1.453717 |
| H | 1.071171 | 0.069699 | -1.529722 |
| I | 3.886826 | 0.009447 | -0.245575 |

**Table S18.** Cartesian coordinates (in Å) for structures referring to Figure S4

| IM2+$BCl_3$, MP2/6-31+G* | x | y | z |
|---|---|---|---|
| B | 1.258610 | 0.292941 | 0.081582 |
| C | 3.791313 | -0.134921 | -0.320683 |
| C | 2.659745 | -0.409589 | 0.337076 |
| Cl | 1.315837 | 1.599008 | -1.246508 |
| Cl | 0.018767 | -1.029555 | -0.421871 |
| Cl | 0.675903 | 1.073496 | 1.684857 |
| Cl | 5.312837 | -0.943194 | 0.002152 |
| H | 2.692127 | -1.178429 | 1.112252 |
| H | 3.883537 | 0.604933 | -1.106911 |
| B | -3.107286 | -0.175537 | -0.017952 |
| Cl | -3.110429 | -0.853822 | 1.584469 |
| Cl | -2.801255 | 1.519289 | -0.246510 |
| Cl | -3.531580 | -1.173838 | -1.381404 |

| IM2+$BCl_3$, MP2/tzvpp | x | y | z |
|---|---|---|---|
| B | 1.213841 | 0.256605 | 0.066092 |
| C | 3.739560 | -0.174940 | -0.288143 |
| C | 2.638361 | -0.328138 | 0.445106 |
| Cl | 1.221918 | 1.217408 | -1.531851 |
| Cl | 0.002745 | -1.179450 | -0.096051 |
| Cl | 0.613414 | 1.373093 | 1.449042 |
| Cl | 5.289250 | -0.836683 | 0.159281 |
| H | 2.719491 | -0.896284 | 1.367159 |
| H | 3.773955 | 0.368828 | -1.219053 |
| B | -3.028337 | -0.142162 | -0.019493 |
| Cl | -3.087439 | -0.088758 | 1.718608 |
| Cl | -2.635939 | 1.286807 | -0.923717 |
| Cl | -3.503272 | -1.597491 | -0.853130 |

| IM2+$BCl_3$, B3LYP | x | y | z |
|---|---|---|---|
| B | 1.242775 | 0.235296 | 0.100824 |
| C | 3.786005 | -0.063591 | -0.317957 |
| C | 2.663907 | -0.452088 | 0.280858 |
| Cl | 1.250561 | 1.701804 | -1.088892 |
| Cl | 0.017000 | -1.073839 | -0.560198 |
| Cl | 0.616514 | 0.827066 | 1.797629 |
| Cl | 5.349233 | -0.872212 | -0.074655 |
| H | 2.707006 | -1.310074 | 0.953254 |
| H | 3.875691 | 0.767961 | -1.005706 |
| B | -3.075228 | -0.139384 | -0.030210 |
| Cl | -3.094167 | -0.867711 | 1.565785 |
| Cl | -2.690881 | 1.557451 | -0.219510 |
| Cl | -3.572961 | -1.086875 | -1.424749 |

| TS3-Cl, MP2/6-31+G* | x | y | z |
|---|---|---|---|
| B | 1.090240 | 0.476285 | 0.072031 |
| C | 3.541970 | -0.264406 | -0.309757 |
| C | 2.420811 | -0.310595 | 0.419519 |
| Cl | 1.198700 | 1.522006 | -1.446657 |
| Cl | -0.231626 | -0.895142 | -0.236827 |
| Cl | 0.554677 | 1.492592 | 1.527962 |
| Cl | 4.997416 | -1.138944 | 0.110661 |
| H | 2.418332 | -0.925572 | 1.321744 |
| H | 3.660190 | 0.312248 | -1.219624 |
| B | -2.557612 | -0.312523 | -0.022985 |
| Cl | -2.767120 | -0.452912 | 1.729963 |
| Cl | -2.593833 | 1.307157 | -0.728353 |
| Cl | -3.188704 | -1.643903 | -1.015921 |

| TS3-Cl, MP2/tzvpp | x | y | z |
|---|---|---|---|
| B | 1.092553 | 0.423730 | 0.076741 |
| C | 3.555643 | -0.241116 | -0.297489 |
| C | 2.439402 | -0.331496 | 0.424499 |
| Cl | 1.195222 | 1.473206 | -1.445546 |
| Cl | -0.216946 | -0.950405 | -0.233367 |
| Cl | 0.530380 | 1.441420 | 1.526559 |
| Cl | 5.029364 | -1.072502 | 0.110423 |
| H | 2.456320 | -0.957421 | 1.311593 |
| H | 3.646619 | 0.353073 | -1.193333 |
| B | -2.609014 | -0.279748 | -0.023189 |
| Cl | -2.771661 | -0.345529 | 1.731138 |
| Cl | -2.562336 | 1.297695 | -0.801227 |
| Cl | -3.232900 | -1.648585 | -0.955514 |

| TS3-Cl, B3LYP | x | y | z |
|---|---|---|---|
| B | 1.110220 | 0.462027 | 0.080011 |
| C | 3.562809 | -0.244005 | -0.312305 |
| C | 2.445595 | -0.325195 | 0.406053 |
| Cl | 1.189228 | 1.552240 | -1.433239 |
| Cl | -0.235236 | -0.946180 | -0.272349 |
| Cl | 0.534507 | 1.443253 | 1.567974 |
| Cl | 5.045600 | -1.122249 | 0.096237 |
| H | 2.440359 | -0.964927 | 1.289033 |
| H | 3.689842 | 0.356841 | -1.204425 |
| B | -2.562385 | -0.303942 | -0.026865 |
| Cl | -2.756172 | -0.401176 | 1.747741 |
| Cl | -2.577079 | 1.316818 | -0.771370 |
| Cl | -3.254955 | -1.652538 | -0.988690 |

| E+$BCl_4^-$, MP2/6-31+G* | x | y | z |
|---|---|---|---|
| B | 1.089970 | 0.681354 | 0.088252 |
| C | 3.415925 | -0.332769 | -0.303957 |
| C | 2.325451 | -0.215215 | 0.466210 |
| Cl | 1.187386 | 1.599149 | -1.472010 |
| Cl | -0.315177 | -0.846914 | -0.248929 |
| Cl | 0.471831 | 1.670632 | 1.476666 |
| Cl | 4.795016 | -1.302375 | 0.135576 |
| H | 2.299593 | -0.751516 | 1.415955 |
| H | 3.545467 | 0.159813 | -1.260902 |
| B | -2.209279 | -0.408232 | -0.036899 |
| Cl | -2.591941 | -0.456368 | 1.747075 |
| Cl | -2.515257 | 1.227027 | -0.773017 |
| Cl | -3.072844 | -1.743269 | -0.946850 |

| E+$BCl_4^-$, MP2/tzvpp | x | y | z |
|---|---|---|---|
| B | 1.080966 | 0.673957 | 0.093746 |
| C | 3.397749 | -0.333909 | -0.289384 |
| C | 2.328319 | -0.192200 | 0.496220 |
| Cl | 1.174420 | 1.541280 | -1.497367 |
| Cl | -0.299120 | -0.876707 | -0.209853 |
| Cl | 0.425624 | 1.693489 | 1.442309 |
| Cl | 4.787591 | -1.268958 | 0.153796 |
| H | 2.328526 | -0.688076 | 1.461098 |
| H | 3.489958 | 0.122764 | -1.262863 |
| B | -2.197427 | -0.404788 | -0.033783 |
| Cl | -2.593170 | -0.377066 | 1.746505 |
| Cl | -2.471323 | 1.200481 | -0.840909 |
| Cl | -3.058880 | -1.772746 | -0.896779 |

| E+$BCl_4^-$, B3LYP | x | y | z |
|---|---|---|---|
| B | 1.736126 | 1.189416 | 0.140259 |
| C | 3.400172 | -0.651622 | -0.277754 |
| C | 2.623700 | 0.017254 | 0.585901 |
| Cl | 1.647981 | 1.728902 | -1.545125 |
| Cl | -0.573176 | -1.151879 | -0.210522 |
| Cl | 0.831293 | 2.109038 | 1.348935 |
| Cl | 4.421244 | -1.985767 | 0.183676 |
| H | 2.621179 | -0.279833 | 1.632575 |
| H | 3.463629 | -0.433606 | -1.337912 |
| B | -2.306615 | -0.447516 | -0.035325 |
| Cl | -2.630537 | -0.028988 | 1.763821 |
| Cl | -2.456826 | 1.100079 | -1.081897 |
| Cl | -3.556190 | -1.723729 | -0.615842 |

| IM2+BBr$_3$, MP2 | x | y | z |
|---|---|---|---|
| B | 1.523920 | 0.281221 | -0.012413 |
| C | 4.049125 | -0.257004 | -0.267446 |
| C | 2.925454 | -0.286992 | 0.461162 |
| Br | 1.563884 | 1.129539 | -1.858873 |
| Br | 0.185003 | -1.276204 | -0.022345 |
| Br | 0.900151 | 1.674385 | 1.352570 |
| Br | 5.711351 | -0.967435 | 0.356372 |
| H | 2.961580 | -0.738181 | 1.454861 |
| H | 4.134484 | 0.161879 | -1.263100 |
| B | -3.144842 | -0.163749 | 0.036787 |
| Br | -3.149685 | 0.062843 | 1.923312 |
| Br | -2.773678 | 1.309183 | -1.100400 |
| Br | -3.648148 | -1.839465 | -0.711858 |

| IM2+BBr$_3$, B3LYP | x | y | z |
|---|---|---|---|
| B | 1.492586 | 0.565917 | 0.022383 |
| C | 3.612019 | -0.910309 | -0.162460 |
| C | 3.042515 | 0.257346 | 0.125969 |
| Br | 0.367200 | -1.041378 | -0.581815 |
| Br | 0.819923 | 1.196299 | 1.873605 |
| Br | 1.220395 | 2.111820 | -1.328768 |
| Br | 5.508716 | -1.227139 | -0.026019 |
| H | 3.677049 | 1.080238 | 0.456995 |
| H | 3.090544 | -1.797056 | -0.498755 |
| B | -2.969593 | -0.316548 | 0.017922 |
| Br | -2.769674 | 1.461099 | -0.637987 |
| Br | -3.431399 | -1.732692 | -1.186176 |
| Br | -2.838298 | -0.671216 | 1.888851 |

| TS3-Br, MP2 | x | y | z |
|---|---|---|---|
| B | 1.329559 | 0.757304 | 0.011718 |
| C | 3.269982 | -0.979289 | -0.170082 |
| C | 2.833717 | 0.265463 | 0.065331 |
| Br | 0.079754 | -0.780280 | -0.618294 |
| Br | 0.758930 | 1.376022 | 1.853262 |
| Br | 1.173912 | 2.280960 | -1.323004 |
| Br | 5.113581 | -1.465033 | -0.062910 |
| H | 3.568713 | 1.029093 | 0.329789 |
| H | 2.646151 | -1.824304 | -0.436847 |
| B | -2.396926 | -0.451465 | -0.004545 |
| Br | -2.659514 | 1.435597 | -0.309732 |
| Br | -3.110921 | -1.666846 | -1.345182 |
| Br | -2.427178 | -1.079021 | 1.825851 |

| TS3-Br, B3LYP | x | y | z |
|---|---|---|---|
| B | 1.359476 | 0.411329 | 0.071468 |
| C | 3.819286 | -0.267143 | -0.324359 |
| C | 2.703376 | -0.355040 | 0.396906 |
| Br | 1.425464 | 1.606965 | -1.570629 |
| Br | -0.071830 | -1.119482 | -0.292416 |
| Br | 0.750193 | 1.473827 | 1.704855 |
| Br | 5.439748 | -1.204572 | 0.115652 |
| H | 2.701353 | -0.990142 | 1.283053 |
| H | 3.940443 | 0.330598 | -1.218763 |
| B | -2.592335 | -0.287272 | -0.015787 |
| Br | -2.769233 | -0.339651 | 1.916188 |
| Br | -2.511240 | 1.445738 | -0.875101 |
| Br | -3.394916 | -1.755044 | -1.020777 |

| E+BBr$_4^-$, MP2 | x | y | z |
| --- | --- | --- | --- |
| B | -0.014836 | -0.062109 | -0.011422 |
| C | 0.008551 | 0.035863 | 2.554427 |
| C | 0.684373 | -0.042033 | 1.397171 |
| Br | -1.995897 | 0.017779 | -0.034994 |
| Br | 0.697917 | 1.948978 | -0.751570 |
| Br | 0.731856 | -1.450339 | -1.213913 |
| Br | 0.863270 | 0.022375 | 4.253025 |
| H | 1.772338 | -0.117841 | 1.427587 |
| H | -1.070133 | 0.109020 | 2.635205 |
| B | 0.726675 | 2.154108 | -2.865804 |
| Br | 2.382110 | 1.281014 | -3.545934 |
| Br | -0.925587 | 1.345145 | -3.609592 |
| Br | 0.781329 | 4.136350 | -3.143221 |

| E+BBr$_4^-$, B3LYP | x | y | z |
| --- | --- | --- | --- |
| B | 1.914017 | 1.182104 | 0.213039 |
| C | 3.635137 | -0.600078 | -0.248470 |
| C | 2.740229 | -0.058120 | 0.589553 |
| Br | 1.981657 | 1.980935 | -1.543964 |
| Br | -0.343709 | -1.182599 | -0.530105 |
| Br | 0.883535 | 2.067157 | 1.572863 |
| Br | 4.684277 | -2.119367 | 0.178287 |
| H | 2.600717 | -0.498507 | 1.574541 |
| H | 3.838659 | -0.231210 | -1.246967 |
| B | -2.202221 | -0.467136 | -0.069189 |
| Br | -2.409462 | -0.385620 | 1.952502 |
| Br | -2.424570 | 1.392280 | -0.864650 |
| Br | -3.607457 | -1.721241 | -0.853314 |

| IM2+BI$_3$, MP2 | x | y | z |
| --- | --- | --- | --- |
| B | 1.746664 | 0.160805 | 0.104957 |
| C | 4.298021 | -0.014343 | -0.389937 |
| C | 3.173194 | -0.519664 | 0.141901 |
| I | 1.694252 | 2.156743 | -0.980382 |
| I | 0.325718 | -1.313079 | -0.928675 |
| I | 1.078407 | 0.453084 | 2.278218 |
| I | 6.181492 | -1.009043 | -0.283418 |
| H | 3.226074 | -1.484440 | 0.651687 |
| H | 4.363246 | 0.931392 | -0.916302 |
| B | -3.343543 | -0.078672 | -0.023334 |
| I | -3.331860 | -1.116217 | 1.842380 |
| I | -2.738040 | 1.960695 | -0.118992 |
| I | -4.048313 | -1.069042 | -1.783759 |

| IM2+BI$_3$, B3LYP | x | y | z |
| --- | --- | --- | --- |
| B | 1.679285 | 0.134863 | 0.132664 |
| C | 4.228076 | 0.085046 | -0.365107 |
| C | 3.092167 | -0.544400 | -0.062170 |
| I | 1.669713 | 2.403329 | -0.145710 |
| I | 0.227221 | -0.817194 | -1.396046 |
| I | 0.944086 | -0.370401 | 2.260891 |
| I | 6.108308 | -0.911820 | -0.600695 |
| H | 3.114119 | -1.626565 | 0.072554 |
| H | 4.326313 | 1.152293 | -0.520224 |
| B | -3.220225 | -0.085712 | -0.030419 |
| I | -3.160035 | -1.720108 | 1.349851 |
| I | -2.579712 | 1.857209 | 0.573409 |
| I | -4.033301 | -0.384701 | -1.994528 |

| TS3-I, MP2 | x | y | z |
|---|---|---|---|
| B | 1.575658 | 0.696175 | 0.011171 |
| C | 3.501470 | -1.054567 | -0.253102 |
| C | 3.067095 | 0.176405 | 0.061868 |
| I | 0.131940 | -0.900560 | -0.794266 |
| I | 0.969274 | 1.289053 | 2.120764 |
| I | 1.510592 | 2.509543 | -1.372077 |
| I | 5.549607 | -1.628163 | -0.124679 |
| H | 3.799738 | 0.913645 | 0.399362 |
| H | 2.872651 | -1.869295 | -0.594648 |
| B | -2.625917 | -0.423803 | -0.008642 |
| I | -2.785525 | 1.724185 | -0.230536 |
| I | -3.508879 | -1.659743 | -1.576109 |
| I | -2.637433 | -1.242565 | 2.001998 |
| TS3-I, B3LYP | x | y | z |
| B | 1.560089 | 0.681054 | 0.026479 |
| C | 3.520244 | -1.015616 | -0.260628 |
| C | 3.059254 | 0.189466 | 0.076311 |
| I | 0.135771 | -0.951683 | -0.813393 |
| I | 0.894696 | 1.234004 | 2.140367 |
| I | 1.438877 | 2.516335 | -1.347332 |
| I | 5.582387 | -1.569015 | -0.139285 |
| H | 3.769358 | 0.937720 | 0.431404 |
| H | 2.916065 | -1.838001 | -0.622749 |
| B | -2.575140 | -0.423133 | -0.019031 |
| I | -2.692455 | 1.735165 | -0.231463 |
| I | -3.523320 | -1.632508 | -1.583388 |
| I | -2.611185 | -1.246118 | 1.998267 |
| E+$BI_4^-$, MP2 | x | y | z |
| B | 1.533807 | 0.614138 | 0.084293 |
| C | 3.882183 | -0.373717 | -0.367380 |
| C | 2.766412 | -0.320418 | 0.381972 |
| I | 1.713933 | 1.962396 | -1.690500 |
| I | -0.098900 | -1.090861 | -0.451858 |
| I | 0.901378 | 1.697653 | 1.935189 |
| I | 5.535285 | -1.623386 | 0.102255 |
| H | 2.703855 | -0.948239 | 1.273265 |
| H | 4.045775 | 0.211603 | -1.265796 |
| B | -2.310051 | -0.425360 | -0.052334 |
| I | -2.730921 | -0.599652 | 2.139902 |
| I | -2.607443 | 1.650508 | -0.815360 |
| I | -3.520123 | -1.921988 | -1.224436 |
| E+$BI_4^-$, B3LYP | x | y | z |
| B | 2.128417 | 1.226155 | 0.215429 |
| C | 3.859071 | -0.556591 | -0.254013 |
| C | 2.999801 | 0.019819 | 0.602020 |
| I | 2.076309 | 2.026201 | -1.787807 |
| I | -0.208916 | -1.465802 | -0.206353 |
| I | 1.030866 | 2.241931 | 1.763578 |
| I | 5.071074 | -2.217893 | 0.227687 |
| H | 2.917283 | -0.370827 | 1.614442 |
| H | 3.998901 | -0.229925 | -1.278395 |
| B | -2.273414 | -0.503576 | -0.064203 |
| I | -2.749570 | -0.063978 | 2.117097 |
| I | -2.288463 | 1.426505 | -1.263536 |
| I | -3.824591 | -1.943030 | -0.910669 |

**Table S19.** Cartesian coordinates (in Å) for structures referring to Figures S5 and S6

| $C_3H_4$+$BBr_3$, MP2 | x | y | z |
|---|---|---|---|
| B | -0.460333 | 0.013163 | 0.323374 |
| C | 2.212352 | -0.079900 | -1.843773 |
| C | 1.311028 | -0.134763 | -2.671878 |
| Br | 0.346206 | 1.622705 | 0.929992 |
| Br | -1.975966 | 0.084943 | -0.820541 |
| Br | 0.192586 | -1.665120 | 0.928342 |
| C | 3.341227 | -0.014454 | -0.912485 |
| H | 0.500569 | -0.185715 | -3.367325 |
| H | 3.500871 | 1.011796 | -0.569319 |
| H | 4.258164 | -0.362198 | -1.397260 |
| H | 3.155531 | -0.643472 | -0.036902 |
| $C_3H_4$+$BBr_3$, B3LYP | x | y | z |
| B | -0.439684 | -0.004411 | -0.333071 |
| C | 2.117107 | 0.025039 | 1.926036 |
| C | 1.174958 | 0.044219 | 2.686799 |
| Br | 0.386926 | -1.613694 | -0.944394 |
| Br | -2.008240 | -0.089506 | 0.753079 |
| Br | 0.250027 | 1.689361 | -0.882991 |
| C | 3.244415 | 0.005058 | 0.998118 |
| H | 0.342594 | 0.059681 | 3.355097 |
| H | 3.415694 | -1.006725 | 0.614170 |
| H | 4.163749 | 0.344839 | 1.488303 |
| H | 3.052515 | 0.662736 | 0.142779 |
| TS1-Br, MP2 | x | y | z |
| B | 0.190350 | 0.000111 | -0.018510 |
| C | -1.638792 | 0.001719 | 1.864998 |
| C | -0.423178 | 0.003745 | 2.075177 |
| Br | -1.269811 | -0.012636 | -1.306843 |
| Br | 1.189951 | 1.671533 | 0.081292 |
| Br | 1.207844 | -1.659886 | 0.093098 |
| C | -3.052219 | -0.000461 | 1.524086 |
| H | 0.515160 | 0.006768 | 2.592359 |
| H | -3.313860 | 0.888731 | 0.944750 |
| H | -3.634029 | 0.003784 | 2.452567 |
| H | -3.313296 | -0.895224 | 0.953165 |
| TS1-Br, B3LYP | x | y | z |
| B | 0.205725 | -0.000030 | -0.042245 |
| C | -1.712578 | -0.001816 | 1.869916 |
| C | -0.510064 | -0.004100 | 2.087619 |
| Br | -1.251341 | 0.014894 | -1.345844 |
| Br | 1.222836 | 1.665123 | 0.115605 |
| Br | 1.201752 | -1.678990 | 0.101349 |
| C | -3.117511 | 0.000664 | 1.511842 |
| H | 0.420830 | -0.007356 | 2.613324 |
| H | -3.376720 | 0.897743 | 0.940243 |
| H | -3.720279 | -0.008518 | 2.428831 |
| H | -3.375174 | -0.886149 | 0.923708 |

| IM-Br, MP2 | x | y | z |
|---|---|---|---|
| B | 0.126907 | 0.000091 | 0.121167 |
| C | -1.589001 | 0.000223 | 1.785214 |
| C | -0.358187 | 0.000901 | 1.926198 |
| Br | -1.295132 | -0.004364 | -1.261497 |
| Br | 1.197832 | 1.671344 | 0.069011 |
| Br | 1.204335 | -1.667155 | 0.072712 |
| C | -3.014100 | -0.000310 | 1.542882 |
| H | 0.531128 | 0.001958 | 2.530115 |
| H | -3.322280 | 0.896149 | 1.000067 |
| H | -3.499799 | 0.000866 | 2.528060 |
| H | -3.322076 | -0.898146 | 1.002249 |
| IM-Br, B3LYP | x | y | z |
| B | -0.131302 | -0.000006 | 0.179811 |
| C | 1.854064 | -0.000040 | 1.631723 |
| C | 0.598280 | -0.000086 | 1.704553 |
| Br | 1.224333 | 0.000391 | -1.336068 |
| Br | -1.249041 | -1.678562 | 0.141016 |
| Br | -1.249567 | 1.678205 | 0.141393 |
| C | 3.275311 | -0.000019 | 1.484850 |
| H | -0.061872 | -0.000198 | 2.563665 |
| H | 3.637341 | -0.905179 | 0.987101 |
| H | 3.677356 | -0.000221 | 2.514061 |
| H | 3.637366 | 0.905308 | 0.987427 |
| TS2-Br, MP2 | x | y | z |
| B | 0.219431 | 0.000004 | 0.252909 |
| C | -1.877693 | -0.000027 | 1.325307 |
| C | -0.631712 | 0.000007 | 1.626622 |
| Br | -1.306899 | -0.000257 | -1.176432 |
| Br | 1.297844 | 1.665798 | 0.104607 |
| Br | 1.298253 | -1.665540 | 0.104806 |
| C | -3.319229 | -0.000003 | 1.340591 |
| H | -0.213997 | 0.000067 | 2.632572 |
| H | -3.735715 | 0.901864 | 0.887917 |
| H | -3.561810 | 0.000051 | 2.417490 |
| H | -3.735742 | -0.901903 | 0.888006 |
| TS2-Br, B3LYP | x | y | z |
| B | 0.219054 | -0.000018 | 0.258080 |
| C | -1.912075 | 0.000019 | 1.360339 |
| C | -0.657472 | -0.000013 | 1.613857 |
| Br | -1.307890 | 0.000490 | -1.209914 |
| Br | 1.308758 | 1.674789 | 0.114676 |
| Br | 1.308043 | -1.675256 | 0.114271 |
| C | -3.349526 | -0.000055 | 1.378577 |
| H | -0.238243 | -0.000079 | 2.619144 |
| H | -3.772864 | 0.901787 | 0.927631 |
| H | -3.608861 | -0.000508 | 2.453137 |
| H | -3.772763 | -0.901600 | 0.926942 |

| Z-Br, MP2 | x | y | z |
|---|---|---|---|
| B | -0.887615 | -0.189982 | 0.167599 |
| C | 1.559823 | -1.198394 | 0.301860 |
| C | 0.211087 | -1.227654 | 0.445082 |
| Br | 2.500143 | 0.312803 | -0.377679 |
| Br | -2.631212 | -0.881143 | -0.252581 |
| Br | -0.696652 | 1.702380 | 0.292628 |
| C | 2.470055 | -2.347018 | 0.609572 |
| H | -0.155167 | -2.200796 | 0.780342 |
| H | 3.000376 | -2.669576 | -0.292543 |
| H | 1.893725 | -3.186696 | 1.007420 |
| H | 3.223608 | -2.046047 | 1.344809 |

| Z-Br, B3LYP | x | y | z |
|---|---|---|---|
| B | 0.898632 | -0.200511 | 0.000409 |
| C | -1.530826 | -1.276939 | 0.000768 |
| C | -0.178533 | -1.285853 | 0.001070 |
| Br | -2.584922 | 0.318402 | -0.000973 |
| Br | 0.635817 | 1.700176 | 0.000805 |
| Br | 2.738996 | -0.812611 | -0.000674 |
| C | -2.381071 | -2.510661 | 0.001558 |
| H | 0.220690 | -2.301863 | 0.001833 |
| H | -3.032369 | -2.527642 | 0.883085 |
| H | -1.753864 | -3.406548 | 0.002895 |
| H | -3.031220 | -2.529516 | -0.880786 |

| $C_3H_4$+$BCl_3$, MP2 | x | y | z |
|---|---|---|---|
| B | 0.691762 | -0.091758 | -0.448401 |
| C | -2.143554 | 0.361065 | 1.312090 |
| C | -1.361863 | 0.890729 | 2.091343 |
| Cl | -0.233222 | 0.605514 | -1.745951 |
| Cl | 1.885626 | 0.865048 | 0.378041 |
| Cl | 0.475468 | -1.765212 | -0.028641 |
| C | -3.144912 | -0.256493 | 0.439361 |
| H | -0.646620 | 1.342911 | 2.745195 |
| H | -4.077336 | -0.422044 | 0.986419 |
| H | -2.791257 | -1.221408 | 0.064835 |
| H | -3.359446 | 0.386339 | -0.418849 |

| $C_3H_4$+$BCl_3$, B3LYP | x | y | z |
|---|---|---|---|
| B | -0.829783 | -0.244352 | 0.115734 |
| C | 2.333470 | 0.751693 | -0.263749 |
| C | 1.767570 | 1.822432 | -0.251083 |
| Cl | -1.741079 | 1.243127 | 0.294806 |
| Cl | -0.632077 | -0.965217 | -1.470712 |
| Cl | -0.199322 | -1.062425 | 1.532954 |
| C | 3.006528 | -0.544240 | -0.278446 |
| H | 1.269411 | 2.767045 | -0.240348 |
| H | 4.042323 | -0.444432 | -0.621723 |
| H | 2.492155 | -1.242160 | -0.948780 |
| H | 3.021014 | -0.987941 | 0.723227 |

| TS1-Cl | | x | y | z |
|---|---|---|---|---|
| | B | 0.538178 | 0.000288 | -0.112117 |
| | C | -1.842717 | -0.002651 | 1.005858 |
| | C | -0.771717 | -0.004266 | 1.597541 |
| | Cl | 1.394288 | 1.534453 | 0.294834 |
| | Cl | 1.392858 | -1.537090 | 0.285574 |
| | Cl | -0.345099 | 0.005475 | -1.683497 |
| | C | -3.050343 | -0.000374 | 0.206605 |
| | H | -0.088465 | -0.006564 | 2.421686 |
| | H | -3.917669 | -0.001654 | 0.879141 |
| | H | -3.105748 | -0.892065 | -0.425981 |
| | H | -3.105134 | 0.894333 | -0.421767 |
| IM-Cl | | x | y | z |
| | B | 0.452140 | 0.000000 | -0.022386 |
| | C | -1.861453 | 0.000000 | -0.903595 |
| | C | -0.686076 | 0.000000 | -1.332068 |
| | Cl | 1.438628 | -1.541206 | -0.289027 |
| | Cl | 1.438617 | 1.541215 | -0.289017 |
| | Cl | -0.361708 | -0.000008 | 1.649230 |
| | C | -3.158969 | -0.000001 | -0.302111 |
| | H | -0.238637 | 0.000000 | -2.316564 |
| | H | -3.882675 | -0.000011 | -1.135256 |
| | H | -3.332266 | 0.904356 | 0.290108 |
| | H | -3.332258 | -0.904351 | 0.290121 |
| TS2-Cl, MP2 | | x | y | z |
| | B | 0.441641 | 0.042032 | 0.175397 |
| | C | -1.815506 | 0.093497 | 0.678555 |
| | C | -0.705552 | 0.229312 | 1.304642 |
| | Cl | -0.684017 | -0.258936 | -1.366378 |
| | Cl | 1.410998 | 1.565219 | -0.050487 |
| | Cl | 1.460495 | -1.430642 | 0.498522 |
| | C | -3.215056 | 0.003406 | 0.335746 |
| | H | -0.569924 | 0.426635 | 2.365397 |
| | H | -3.726917 | 0.172704 | 1.297130 |
| | H | -3.483124 | -0.986969 | -0.036842 |
| | H | -3.516468 | 0.783292 | -0.365904 |
| TS2-Cl, B3LYP | | x | y | z |
| | B | -0.543845 | -0.000005 | -0.157384 |
| | C | 1.749211 | 0.000004 | -0.699266 |
| | C | 0.606179 | -0.000007 | -1.284968 |
| | Cl | -1.548706 | 1.535958 | -0.165212 |
| | Cl | -1.548557 | -1.536068 | -0.165080 |
| | Cl | 0.659256 | 0.000123 | 1.428252 |
| | C | 3.161254 | -0.000014 | -0.409897 |
| | H | 0.469619 | -0.000032 | -2.364677 |
| | H | 3.646321 | -0.000004 | -1.401499 |
| | H | 3.474762 | -0.900126 | 0.126278 |
| | H | 3.474780 | 0.900079 | 0.126300 |

| Z-Cl, MP2 | x | y | z |
|---|---|---|---|
| B | 0.832556 | 0.180417 | 0.587299 |
| C | -1.732513 | -0.182494 | 0.078025 |
| C | -0.685257 | 0.286395 | 0.799947 |
| Cl | -1.532530 | -1.074109 | -1.403082 |
| Cl | 1.848407 | 1.327086 | 1.453590 |
| Cl | 1.636897 | -1.016763 | -0.399478 |
| C | -3.169456 | 0.023248 | 0.440396 |
| H | -0.992267 | 0.866659 | 1.670961 |
| H | -3.248033 | 0.578985 | 1.377343 |
| H | -3.672005 | -0.943129 | 0.551270 |
| H | -3.687587 | 0.574767 | -0.350851 |
| Z-Cl, B3LYP | x | y | z |
| B | -1.083159 | -0.127837 | 0.000052 |
| C | 1.506806 | -0.649094 | 0.000026 |
| C | 0.193845 | -0.969641 | 0.000151 |
| Cl | -1.176259 | 1.635153 | 0.000020 |
| Cl | -2.635055 | -1.002027 | -0.000057 |
| Cl | 2.086163 | 1.009213 | -0.000033 |
| C | 2.626246 | -1.642733 | -0.000034 |
| H | 0.026169 | -2.046817 | 0.000321 |
| H | 2.233868 | -2.662649 | 0.000103 |
| H | 3.261137 | -1.501062 | 0.882297 |
| H | 3.260815 | -1.501238 | -0.882633 |
| $C_3H_4$+$BI_3$, MP2 | x | y | z |
| B | 0.246026 | -0.006987 | -0.336451 |
| C | -1.691595 | 0.063755 | 2.715876 |
| C | -0.576568 | 0.116773 | 3.221185 |
| I | -0.860083 | -1.790019 | -0.731126 |
| I | -0.635715 | 1.902843 | -0.701336 |
| I | 2.261933 | -0.136298 | 0.357569 |
| C | -3.062335 | 0.008347 | 2.202265 |
| H | 0.416935 | 0.161576 | 3.613509 |
| H | -3.152207 | 0.589052 | 1.279266 |
| H | -3.762793 | 0.418867 | 2.935659 |
| H | -3.354189 | -1.023707 | 1.987187 |
| $C_3H_4$+$BI_3$, B3LYP/SVP | x | y | z |
| B | 0.229466 | -0.003016 | -0.326139 |
| C | -1.570118 | 0.018356 | 2.702125 |
| C | -0.430741 | 0.033487 | 3.112500 |
| I | -0.890833 | -1.786206 | -0.704016 |
| I | -0.669828 | 1.913045 | -0.647944 |
| I | 2.274710 | -0.133647 | 0.293470 |
| C | -2.940482 | 0.003172 | 2.198303 |
| H | 0.575321 | 0.045517 | 3.469267 |
| H | -3.007498 | 0.538152 | 1.243978 |
| H | -3.624629 | 0.485272 | 2.905902 |
| H | -3.287097 | -1.023109 | 2.033989 |

| $C_3H_4$+$BI_3$, B3LYP/ECP46MWB | x | y | z |
|---|---|---|---|
| B | -0.107069 | -0.063654 | -0.190595 |
| C | 0.260294 | 0.200290 | 3.333868 |
| C | 1.280976 | 0.730764 | 2.955006 |
| I | 0.049831 | -2.168346 | 0.153280 |
| I | -1.956477 | 0.945843 | 0.172780 |
| I | 1.562817 | 1.017201 | -0.978129 |
| C | -0.972002 | -0.442233 | 3.781976 |
| H | 2.179901 | 1.197555 | 2.617647 |
| H | -1.826804 | 0.235183 | 3.677451 |
| H | -0.899987 | -0.740651 | 4.833990 |
| H | -1.180128 | -1.339906 | 3.188258 |

| TS-I, MP2 | x | y | z |
|---|---|---|---|
| B | -0.103188 | 0.000036 | 0.043620 |
| C | 1.215041 | 0.000307 | 2.505183 |
| C | -0.010739 | 0.000660 | 2.363260 |
| I | -1.217167 | 1.873032 | -0.069408 |
| I | 1.857347 | -0.002554 | -0.905551 |
| I | -1.221308 | -1.870593 | -0.068118 |
| C | 2.668311 | -0.000088 | 2.574309 |
| H | -1.056515 | 0.001209 | 2.592800 |
| H | 3.081550 | -0.891966 | 2.095186 |
| H | 2.972923 | 0.000781 | 3.626924 |
| H | 3.082103 | 0.890647 | 2.093535 |

| TS-I, B3LYP/SVP | x | y | z |
|---|---|---|---|
| B | -0.102246 | -0.001388 | 0.047090 |
| C | 1.227395 | -0.013234 | 2.506400 |
| C | 0.013239 | -0.028291 | 2.372883 |
| I | -1.316729 | 1.816636 | -0.038622 |
| I | 1.858386 | 0.115407 | -0.910057 |
| I | -1.129006 | -1.927292 | -0.096696 |
| C | 2.675760 | 0.004366 | 2.577778 |
| H | -1.030950 | -0.049163 | 2.598458 |
| H | 3.105339 | -0.881194 | 2.097724 |
| H | 2.984412 | 0.007230 | 3.630939 |
| H | 3.083560 | 0.901236 | 2.099899 |

| TS-I, B3LYP/ECP46MWB | x | y | z |
|---|---|---|---|
| B | -0.003709 | 0.005002 | -0.026113 |
| C | 0.000806 | 0.007223 | 2.804744 |
| C | 1.007914 | 0.025475 | 2.115438 |
| I | 0.963971 | -1.855764 | -0.641621 |
| I | -2.184438 | -0.028814 | 0.078265 |
| I | 0.905122 | 1.892911 | -0.648916 |
| C | -1.240309 | -0.015925 | 3.555643 |
| H | 2.030891 | 0.043809 | 1.807895 |
| H | -1.850528 | 0.866935 | 3.337738 |
| H | -1.013166 | -0.018453 | 4.629150 |
| H | -1.822804 | -0.914936 | 3.327941 |

| IM-I, MP2 | x | y | z |
|---|---|---|---|
| B | -0.050118 | 0.000073 | 0.323794 |
| C | 1.289794 | 0.000075 | 2.278011 |
| C | 0.059253 | 0.000407 | 2.066607 |
| I | -1.223887 | 1.877051 | -0.054326 |
| I | 1.839882 | -0.002304 | -0.891684 |
| I | -1.227639 | -1.874807 | -0.053235 |
| C | 2.712195 | -0.000112 | 2.490070 |
| H | -0.897338 | 0.000876 | 2.566827 |
| H | 3.181611 | -0.902093 | 2.091074 |
| H | 2.834023 | 0.000774 | 3.585374 |
| H | 3.181990 | 0.901047 | 2.089646 |
| **IM-I, B3LYP** | **x** | **y** | **z** |
| B | 0.234292 | 0.000661 | 0.351090 |
| C | -0.241767 | 0.003230 | 2.764662 |
| C | 0.701980 | -0.005913 | 1.914187 |
| I | 1.044258 | -1.887518 | -0.586298 |
| I | -2.071070 | 0.024054 | 0.257700 |
| I | 1.082526 | 1.874301 | -0.581686 |
| C | -1.231079 | 0.013626 | 3.794214 |
| H | 1.761037 | -0.017191 | 2.167292 |
| H | -1.834610 | 0.926213 | 3.789115 |
| H | -0.642004 | 0.006098 | 4.732116 |
| H | -1.855202 | -0.884994 | 3.787696 |
| **TS2-I, MP2** | **x** | **y** | **z** |
| B | -0.109807 | -0.000167 | 0.346614 |
| C | 1.691736 | 0.001064 | 1.933394 |
| C | 0.413647 | -0.000727 | 1.875795 |
| I | -1.289548 | 1.872202 | 0.009145 |
| I | 1.810318 | 0.002578 | -0.931345 |
| I | -1.285996 | -1.874427 | 0.007428 |
| C | 3.081460 | -0.001812 | 2.300446 |
| H | -0.254241 | -0.001494 | 2.738120 |
| H | 3.597521 | -0.906931 | 1.974518 |
| H | 3.039685 | -0.001047 | 3.405725 |
| H | 3.601935 | 0.900459 | 1.973665 |
| **TS2-I, B3LYP** | **x** | **y** | **z** |
| B | -0.017181 | 0.000293 | -0.002915 |
| C | 0.054849 | 0.002399 | 2.448321 |
| C | 0.786416 | 0.000690 | 1.405387 |
| I | 0.534831 | -1.881548 | -1.115008 |
| I | -2.280672 | 0.003425 | 0.512651 |
| I | 0.539228 | 1.878685 | -1.118637 |
| C | -0.625672 | 0.004103 | 3.705842 |
| H | 1.876023 | -0.000480 | 1.432562 |
| H | -1.218609 | 0.909724 | 3.863741 |
| H | 0.193925 | 0.003796 | 4.450901 |
| H | -1.220517 | -0.900027 | 3.865117 |

| Z-I, MP2 | x | y | z |
|---|---|---|---|
| B | -0.697760 | 0.000021 | 0.582355 |
| C | 1.774920 | 0.000380 | 1.333363 |
| C | 0.457162 | 0.000308 | 1.611045 |
| I | -1.545187 | 1.857979 | -0.062821 |
| I | 2.391485 | 0.000121 | -0.722538 |
| I | -1.544519 | -1.858308 | -0.062630 |
| C | 2.896120 | 0.000674 | 2.325578 |
| H | 0.183028 | 0.000498 | 2.672184 |
| H | 3.529853 | -0.883477 | 2.200064 |
| H | 2.482778 | 0.000790 | 3.339716 |
| H | 3.529649 | 0.884930 | 2.199778 |
| **Z-I, B3LYP/SVP** | **x** | **y** | **z** |
| C | -1.774457 | 0.000747 | 1.341286 |
| C | -0.465719 | 0.000592 | 1.612501 |
| C | -2.904854 | 0.001345 | 2.323235 |
| H | -0.190367 | 0.000952 | 2.672472 |
| B | 0.696092 | 0.000054 | 0.596130 |
| I | -2.395861 | 0.000191 | -0.724096 |
| I | 1.547953 | -1.860369 | -0.062706 |
| I | 1.549177 | 1.859766 | -0.063102 |
| H | -2.506922 | 0.001597 | 3.344212 |
| H | -3.539913 | 0.884936 | 2.192418 |
| H | -3.540334 | -0.882032 | 2.193013 |
| **Z-I, B3LYP/ECP46MWB** | **x** | **y** | **z** |
| B | 0.917781 | -0.000239 | 0.079131 |
| C | -0.449816 | -0.000144 | 2.268758 |
| C | 0.709281 | -0.000304 | 1.605698 |
| I | 1.134729 | -1.856914 | -0.980807 |
| I | -2.295738 | 0.000244 | 1.122329 |
| I | 1.135417 | 1.856508 | -0.980540 |
| C | -0.656840 | -0.000191 | 3.749612 |
| H | 1.615996 | -0.000494 | 2.220403 |
| H | -1.220760 | 0.883766 | 4.068521 |
| H | 0.314984 | -0.000390 | 4.255744 |
| H | -1.221071 | -0.883985 | 4.068423 |

**Table S20.** Cartesian coordinates (in Å) for structures referring to Figure S8

| $C_3H_4$+$BCl_3$+$Cl^-$, MP2 | x | y | z |
|---|---|---|---|
| B | -2.110489 | 0.210660 | 0.100769 |
| C | 1.080634 | -0.705082 | -0.347643 |
| C | 0.521410 | -1.791997 | -0.424330 |
| Cl | -1.764124 | 1.328626 | -1.185857 |
| Cl | -3.051237 | -1.217676 | -0.221000 |
| Cl | -1.599181 | 0.566920 | 1.723644 |
| C | 1.763924 | 0.587759 | -0.273265 |
| H | 0.052581 | -2.751566 | -0.475254 |
| Cl | 5.501605 | -0.067609 | 0.095247 |
| H | 1.574844 | 1.173130 | -1.178101 |
| H | 2.843787 | 0.443955 | -0.169834 |
| H | 1.405349 | 1.162673 | 0.586201 |

| $C_3H_4$+$BCl_3$+$Cl^-$, B3LYP | x | y | z |
|---|---|---|---|
| B | -2.107445 | 0.283384 | -0.000030 |
| C | 0.967846 | -0.975105 | 0.000125 |
| C | 0.303841 | -1.988881 | 0.000090 |
| Cl | -1.628817 | 1.018554 | 1.518125 |
| Cl | -1.629105 | 1.017927 | -1.518580 |
| Cl | -3.162292 | -1.119638 | 0.000357 |
| C | 1.758884 | 0.251725 | 0.000138 |
| H | -0.274714 | -2.886203 | 0.000027 |
| Cl | 5.639399 | 0.024024 | -0.000045 |
| H | 1.533839 | 0.856984 | -0.885670 |
| H | 2.834422 | 0.034317 | -0.000029 |
| H | 1.534104 | 0.856795 | 0.886142 |

| TS1-Cl, MP2 | x | y | z |
|---|---|---|---|
| B | -1.635179 | -0.024461 | 0.009947 |
| C | 0.924893 | -0.171531 | -0.089142 |
| C | 0.160548 | -1.134525 | -0.180976 |
| Cl | -1.421408 | 1.748152 | -0.001386 |
| Cl | -2.411399 | -0.692304 | -1.458212 |
| Cl | -2.137090 | -0.725759 | 1.576132 |
| C | 1.736845 | 1.024078 | 0.025651 |
| H | -0.173155 | -2.149089 | -0.267888 |
| Cl | 5.119309 | -0.329835 | -0.027192 |
| H | 1.544718 | 1.705429 | -0.807133 |
| H | 2.791824 | 0.716963 | 0.002268 |
| H | 1.538783 | 1.536572 | 0.971014 |

| TS1-Cl, B3LYP | x | y | z |
|---|---|---|---|
| B | -1.755568 | 0.018959 | -0.000132 |
| C | 0.948283 | -0.301472 | 0.000859 |
| C | 0.164935 | -1.237963 | 0.001381 |
| Cl | -1.396146 | 1.772522 | -0.001719 |
| Cl | -2.381909 | -0.668278 | -1.533362 |
| Cl | -2.382443 | -0.665371 | 1.534178 |
| C | 1.811376 | 0.860902 | 0.000183 |
| H | -0.249690 | -2.223403 | 0.002086 |
| Cl | 5.297624 | -0.278737 | -0.000035 |
| H | 1.642694 | 1.473416 | -0.892065 |
| H | 2.863324 | 0.529709 | 0.000263 |
| H | 1.642817 | 1.474383 | 0.891788 |

| IM1+$Cl^-$, MP2 | x | y | z |
|---|---|---|---|
| B | -1.518637 | -0.065941 | 0.000744 |
| C | 0.900528 | -0.113149 | -0.013270 |
| C | 0.101779 | -1.057697 | -0.020914 |
| Cl | -1.390259 | 1.737639 | 0.082748 |
| Cl | -2.238904 | -0.645895 | -1.558351 |
| Cl | -2.270449 | -0.776543 | 1.489819 |
| C | 1.747726 | 1.052001 | -0.008250 |
| H | -0.146225 | -2.102580 | -0.039460 |
| Cl | 5.033560 | -0.364856 | 0.004266 |
| H | 2.786567 | 0.680669 | -0.028179 |
| H | 1.600947 | 1.642219 | 0.899448 |
| H | 1.574584 | 1.666603 | -0.895109 |

| IM1+Cl$^-$, B3LYP | x | y | z |
|---|---|---|---|
| B | -1.436782 | -0.114829 | 0.000348 |
| C | 1.081130 | 0.238887 | 0.000843 |
| C | 0.131839 | -0.610203 | 0.001051 |
| Cl | -1.561935 | 1.758771 | 0.015388 |
| Cl | -2.233778 | -0.802228 | -1.549106 |
| Cl | -2.244389 | -0.828189 | 1.532439 |
| C | 2.140272 | 1.133120 | -0.000197 |
| H | 0.318294 | -1.684120 | 0.000195 |
| Cl | 4.815073 | -0.492631 | 0.000646 |
| H | 3.094813 | 0.431810 | -0.000084 |
| H | 2.238958 | 1.724756 | 0.915107 |
| H | 2.237899 | 1.723588 | -0.916383 |
| **TS2-Cl, MP2** | **x** | **y** | **z** |
| B | -1.198254 | -0.134571 | 0.001408 |
| C | 1.150256 | 0.511194 | 0.038377 |
| C | 0.512498 | -0.560073 | 0.076727 |
| Cl | -1.582302 | 1.641261 | -0.213553 |
| Cl | -1.776430 | -1.111379 | -1.434819 |
| Cl | -1.835880 | -0.768130 | 1.595740 |
| C | 1.648825 | 1.861764 | 0.004024 |
| H | 0.660282 | -1.625349 | 0.143803 |
| Cl | 4.021258 | -0.652114 | 0.001286 |
| H | 2.732279 | 1.774354 | 0.147332 |
| H | 1.224802 | 2.450199 | 0.821615 |
| H | 1.451437 | 2.332491 | -0.961692 |
| **TS2-Cl, B3LYP** | **x** | **y** | **z** |
| B | 1.198482 | -0.171211 | -0.000946 |
| C | -1.190391 | 0.555694 | 0.009054 |
| C | -0.453289 | -0.468492 | -0.001441 |
| Cl | 1.581700 | 1.661574 | 0.046348 |
| Cl | 1.872656 | -1.018832 | 1.513367 |
| Cl | 1.854747 | -0.935298 | -1.566929 |
| C | -1.947668 | 1.753964 | 0.016804 |
| H | -0.768635 | -1.506144 | -0.015282 |
| Cl | -4.345309 | -0.636003 | -0.061855 |
| H | -2.992900 | 1.361729 | -0.001368 |
| H | -1.793604 | 2.355848 | -0.884558 |
| H | -1.813419 | 2.334332 | 0.935046 |
| **IM2-Cl, MP2** | **x** | **y** | **z** |
| B | -0.951454 | -0.125307 | 0.000089 |
| C | 1.671307 | 0.062820 | 0.000178 |
| C | 0.547829 | -0.674305 | 0.000118 |
| Cl | -1.317898 | 0.910275 | -1.524903 |
| Cl | -2.117790 | -1.594908 | 0.000464 |
| Cl | -1.318390 | 0.911447 | 1.524117 |
| C | 1.811220 | 1.551472 | -0.000084 |
| H | 0.678262 | -1.757112 | 0.000635 |
| Cl | 3.244756 | -0.758861 | 0.000201 |
| H | 2.364677 | 1.885063 | 0.884743 |
| H | 0.826596 | 2.018260 | 0.000191 |
| H | 2.364067 | 1.885200 | -0.885239 |

| IM2-Cl, B3LYP | x | y | z |
|---|---|---|---|
| B | -0.943452 | -0.131389 | -0.000151 |
| C | 1.670793 | 0.070445 | -0.000476 |
| C | 0.560296 | -0.669700 | -0.000867 |
| Cl | -1.332381 | 0.924444 | -1.540996 |
| Cl | -2.132593 | -1.618786 | -0.001178 |
| Cl | -1.331099 | 0.921325 | 1.543072 |
| C | 1.826989 | 1.557669 | 0.000291 |
| H | 0.688790 | -1.750995 | -0.001289 |
| Cl | 3.270566 | -0.765804 | -0.000491 |
| H | 2.382837 | 1.889612 | 0.885610 |
| H | 0.847149 | 2.037351 | 0.000058 |
| H | 2.383629 | 1.890446 | -0.884219 |

| $C_3H_4$+$BBr_3$+$Br^-$ | x | y | z |
|---|---|---|---|
| B | -1.961158 | -0.054459 | 0.244805 |
| C | 1.289362 | -0.029299 | -0.883160 |
| C | 0.656320 | 0.422909 | -1.831203 |
| Br | -1.345835 | 1.211700 | 1.519357 |
| Br | -2.995369 | 0.524074 | -1.243552 |
| Br | -1.647317 | -1.908547 | 0.513914 |
| C | 1.971017 | -0.603990 | 0.277263 |
| H | 0.182049 | 0.848525 | -2.689511 |
| Br | 5.716994 | -0.078051 | 0.253306 |
| H | 1.573733 | -0.171274 | 1.201326 |
| H | 3.047481 | -0.409632 | 0.236674 |
| H | 1.815929 | -1.687216 | 0.311473 |

| TS1-Br | x | Y | z |
|---|---|---|---|
| B | -1.584383 | 0.020331 | 0.001408 |
| C | 1.027391 | -0.051874 | -0.472744 |
| C | 0.288816 | 0.419198 | -1.342442 |
| Br | -1.331062 | 1.255025 | 1.471605 |
| Br | -2.829709 | 0.575788 | -1.388546 |
| Br | -1.558429 | -1.876510 | 0.396460 |
| C | 1.761413 | -0.634705 | 0.636109 |
| H | -0.069792 | 0.883740 | -2.236991 |
| Br | 5.252231 | 0.129993 | -0.292192 |
| H | 1.481185 | -0.141054 | 1.572841 |
| H | 2.835343 | -0.484575 | 0.462079 |
| H | 1.553364 | -1.705855 | 0.713057 |

| IM1+$Br^-$ | x | y | z |
|---|---|---|---|
| B | 1.290988 | -0.116272 | 0.001532 |
| C | -1.131936 | 0.251537 | 0.000895 |
| C | -0.285659 | -0.686924 | 0.012796 |
| Br | 1.431465 | 1.893304 | -0.075752 |
| Br | 2.110328 | -0.814077 | 1.700681 |
| Br | 2.131048 | -0.942304 | -1.628855 |
| C | -2.047595 | 1.318168 | -0.007923 |
| H | -0.411829 | -1.764373 | 0.033540 |
| Br | -5.049821 | -0.352692 | 0.002590 |
| H | -3.042488 | 0.767902 | -0.001393 |
| H | -2.009759 | 1.907626 | -0.926988 |
| H | -2.005423 | 1.925402 | 0.899338 |

| TS2-Br | | x | y | z |
| --- | --- | --- | --- | --- |
| | B | 1.191203 | -0.107061 | -0.002956 |
| | C | -1.158948 | 0.475371 | -0.007158 |
| | C | -0.446918 | -0.560829 | -0.026908 |
| | Br | 1.532660 | 1.874823 | 0.057150 |
| | Br | 1.919468 | -0.996086 | 1.643611 |
| | Br | 1.943456 | -0.897344 | -1.688267 |
| | C | -1.908967 | 1.679028 | 0.015325 |
| | H | -0.637655 | -1.625927 | -0.054748 |
| | Br | -4.760085 | -0.358651 | -0.009046 |
| | H | -2.952581 | 1.274686 | 0.004586 |
| | H | -1.756477 | 2.283906 | -0.881448 |
| | H | -1.762782 | 2.245238 | 0.938114 |
| IM2-Br | | x | y | z |
| | B | 0.773963 | -0.113512 | 0.002512 |
| | C | -1.825753 | 0.260378 | 0.006689 |
| | C | -0.757705 | -0.557720 | 0.016743 |
| | Br | 1.261702 | 1.050768 | 1.619773 |
| | Br | 1.928805 | -1.802793 | 0.073924 |
| | Br | 1.251231 | 0.909567 | -1.709358 |
| | C | -1.872639 | 1.754193 | -0.009112 |
| | H | -0.956532 | -1.629365 | 0.022271 |
| | Br | -3.599097 | -0.527379 | 0.013294 |
| | H | -2.411983 | 2.111496 | -0.893769 |
| | H | -0.860002 | 2.157707 | -0.020729 |
| | H | -2.397180 | 2.130902 | 0.876565 |
| $C_3H_4$+$BI_3$+$I^-$ | | x | y | z |
| | B | 1.872229 | 0.080306 | -0.435853 |
| | C | -1.188653 | 0.006774 | 1.505732 |
| | C | -0.349404 | -0.291906 | 2.347874 |
| | I | 1.011499 | -1.588910 | -1.448461 |
| | I | 1.254176 | 2.063756 | -0.921978 |
| | I | 3.416586 | -0.229825 | 1.011595 |
| | C | -2.150834 | 0.393496 | 0.472555 |
| | H | 0.340510 | -0.578369 | 3.112523 |
| | I | -5.467180 | 0.746347 | -1.740019 |
| | H | -1.883582 | -0.049269 | -0.491113 |
| | H | -2.178049 | 1.480322 | 0.353400 |
| | H | -3.159515 | 0.058428 | 0.724470 |
| TS1-I | | x | y | z |
| | B | 1.634281 | -0.018438 | 0.000826 |
| | C | -1.097219 | 0.155132 | 0.440886 |
| | C | -0.380900 | -0.285630 | 1.344418 |
| | I | 1.324083 | -1.527154 | -1.530189 |
| | I | 1.650677 | 2.071707 | -0.594333 |
| | I | 2.955428 | -0.553211 | 1.656272 |
| | C | -1.806456 | 0.680800 | -0.712671 |
| | H | -0.046258 | -0.711691 | 2.266616 |
| | I | -5.598656 | -0.084297 | 0.360945 |
| | H | -1.464316 | 0.179578 | -1.625337 |
| | H | -1.645665 | 1.758506 | -0.808903 |
| | H | -2.878928 | 0.490608 | -0.585173 |

| $IM1+I^-$ | | x | y | z |
|---|---|---|---|---|
| | B | -1.186260 | -0.047115 | -0.015173 |
| | C | 1.209221 | 0.529060 | 0.039942 |
| | C | 0.405979 | -0.454508 | -0.005979 |
| | I | -1.515731 | 2.196094 | 0.063919 |
| | I | -2.016153 | -0.900493 | -1.932085 |
| | I | -2.073143 | -1.041899 | 1.805788 |
| | C | 2.146073 | 1.576144 | 0.089041 |
| | H | 0.677648 | -1.508573 | -0.039370 |
| | I | 5.299447 | -0.391141 | 0.064228 |
| | H | 3.112380 | 0.982615 | 0.081739 |
| | H | 2.120400 | 2.142393 | 1.022880 |
| | H | 2.145300 | 2.204986 | -0.804228 |

| TS2-I | | x | y | z |
|---|---|---|---|---|
| | B | -1.152462 | -0.123172 | -0.023615 |
| | C | 1.189827 | 0.606575 | -0.003076 |
| | C | 0.465321 | -0.434715 | -0.021447 |
| | I | -1.629512 | 2.094133 | 0.025571 |
| | I | -1.934093 | -1.056220 | -1.922188 |
| | I | -1.957788 | -1.144103 | 1.818824 |
| | C | 2.042684 | 1.731191 | 0.011627 |
| | H | 0.800546 | -1.469728 | -0.037403 |
| | I | 4.971558 | -0.639227 | 0.071669 |
| | H | 3.036918 | 1.202951 | 0.010644 |
| | H | 1.975490 | 2.315160 | 0.932215 |
| | H | 1.976771 | 2.337044 | -0.894811 |

| IM2-I | | x | y | z |
|---|---|---|---|---|
| | B | -0.661014 | -0.039529 | -0.101041 |
| | C | 1.889967 | 0.312571 | 0.458314 |
| | C | 0.907758 | -0.242147 | -0.280995 |
| | I | -1.278782 | 2.153475 | -0.389197 |
| | I | -1.711724 | -1.284833 | -1.717108 |
| | I | -1.396473 | -0.778421 | 1.945014 |
| | C | 1.782036 | 1.243765 | 1.623814 |
| | H | 1.213444 | -0.899190 | -1.094629 |
| | I | 3.942915 | -0.155336 | -0.049508 |
| | H | 2.254196 | 0.807563 | 2.511722 |
| | H | 0.733249 | 1.445950 | 1.845726 |
| | H | 2.287692 | 2.192342 | 1.408567 |

**Table S21.** Cartesian coordinates (in Å) for structures referring to Figure S9

| IM2+$BCl_3$, MP2 | x | y | z |
|---|---|---|---|
| B | -1.623182 | -0.886792 | 0.036920 |
| C | -1.189224 | 1.704589 | -0.036359 |
| C | -1.289673 | 0.549857 | 0.643163 |
| Cl | -0.277002 | -1.477961 | -1.137254 |
| Cl | -1.742398 | -2.113632 | 1.449619 |
| Cl | -3.257547 | -0.901634 | -0.887621 |
| C | -1.360854 | 1.944632 | -1.502103 |
| H | -1.137015 | 0.607626 | 1.720990 |
| Cl | -0.853738 | 3.200554 | 0.855120 |
| H | -2.288292 | 2.496346 | -1.696358 |
| H | -1.398458 | 0.993491 | -2.033671 |
| H | -0.529923 | 2.540713 | -1.892883 |
| Cl | 2.205095 | 1.385294 | -0.777663 |
| B | 2.481606 | -0.109337 | 0.067500 |
| Cl | 1.940649 | -0.295400 | 1.707599 |
| Cl | 3.402593 | -1.376750 | -0.694999 |
| **IM2+$BCl_3$, B3LYP** | **x** | **y** | **z** |
| B | -1.585614 | -0.919710 | 0.039634 |
| C | -1.306883 | 1.684932 | -0.044059 |
| C | -1.354778 | 0.540620 | 0.639677 |
| Cl | -0.217293 | -1.409268 | -1.202271 |
| Cl | -1.557030 | -2.185951 | 1.457545 |
| Cl | -3.262482 | -1.081790 | -0.853694 |
| C | -1.453424 | 1.926905 | -1.511752 |
| H | -1.213082 | 0.603658 | 1.716709 |
| Cl | -1.018753 | 3.220364 | 0.857260 |
| H | -2.308793 | 2.582969 | -1.714382 |
| H | -1.603715 | 0.981195 | -2.034407 |
| H | -0.558058 | 2.414635 | -1.914662 |
| Cl | 2.185106 | 1.494070 | -0.694303 |
| B | 2.485149 | -0.053066 | 0.073634 |
| Cl | 1.943746 | -0.338112 | 1.713957 |
| Cl | 3.448850 | -1.265979 | -0.756306 |
| **TS3-Cl, MP2** | **x** | **y** | **z** |
| B | -1.190846 | -1.199156 | 0.050907 |
| C | -1.723202 | 1.368864 | -0.043879 |
| C | -1.459900 | 0.246350 | 0.647914 |
| Cl | 0.478459 | -1.215245 | -0.923614 |
| Cl | -1.037389 | -2.435286 | 1.426730 |
| Cl | -2.485399 | -1.782873 | -1.148898 |
| C | -1.838799 | 1.553919 | -1.523289 |
| H | -1.418002 | 0.343550 | 1.732630 |
| Cl | -2.000370 | 2.873378 | 0.846095 |
| H | -2.885120 | 1.713976 | -1.809812 |
| H | -1.464367 | 0.672771 | -2.044733 |
| H | -1.266309 | 2.429399 | -1.843948 |
| Cl | 1.614630 | 1.817718 | -0.738848 |
| B | 2.101480 | 0.287231 | 0.009731 |
| Cl | 1.812460 | 0.103996 | 1.744725 |
| Cl | 3.535964 | -0.515503 | -0.666295 |

| TS3-Cl, B3LYP | x | y | z |
|---|---|---|---|
| B | -1.219832 | -1.196962 | 0.059941 |
| C | -1.771910 | 1.358843 | -0.053112 |
| C | -1.513561 | 0.249645 | 0.643608 |
| Cl | 0.464362 | -1.188775 | -0.976047 |
| Cl | -0.992242 | -2.439327 | 1.447570 |
| Cl | -2.521212 | -1.844416 | -1.138170 |
| C | -1.857580 | 1.563253 | -1.531366 |
| H | -1.482381 | 0.343834 | 1.727057 |
| Cl | -2.059531 | 2.889751 | 0.846205 |
| H | -2.866212 | 1.882939 | -1.820636 |
| H | -1.618844 | 0.637859 | -2.056915 |
| H | -1.156331 | 2.341627 | -1.851495 |
| Cl | 1.652708 | 1.862171 | -0.698130 |
| B | 2.139575 | 0.298816 | 0.015003 |
| Cl | 1.841411 | 0.062671 | 1.756187 |
| Cl | 3.578231 | -0.503603 | -0.692175 |
| $E+BCl_4^-$, MP2 | x | y | z |
| B | -1.112912 | -1.297690 | 0.093777 |
| C | -1.910875 | 1.191184 | -0.047574 |
| C | -1.517596 | 0.110892 | 0.653835 |
| Cl | 0.759989 | -1.068276 | -0.859117 |
| Cl | -0.811630 | -2.518734 | 1.408574 |
| Cl | -2.104913 | -2.002618 | -1.264692 |
| C | -2.037248 | 1.360754 | -1.527330 |
| H | -1.471786 | 0.225676 | 1.736013 |
| Cl | -2.350733 | 2.651022 | 0.838882 |
| H | -3.093331 | 1.379946 | -1.822137 |
| H | -1.542499 | 0.542813 | -2.050465 |
| H | -1.584496 | 2.306909 | -1.836561 |
| Cl | 1.358026 | 1.928949 | -0.714844 |
| B | 1.889322 | 0.311721 | -0.054762 |
| Cl | 1.724293 | 0.219596 | 1.755454 |
| Cl | 3.578167 | -0.121851 | -0.616934 |
| $E+BCl_4^-$, B3LYP | x | y | z |
| B | -1.949138 | -1.249061 | 0.387483 |
| C | -1.937792 | 1.326539 | 0.026067 |
| C | -1.852966 | 0.222673 | 0.795424 |
| Cl | 0.986542 | -1.114931 | -1.156390 |
| Cl | -1.468037 | -2.469415 | 1.588760 |
| Cl | -2.641581 | -1.876536 | -1.122076 |
| C | -2.088820 | 1.470780 | -1.450467 |
| H | -1.660276 | 0.390114 | 1.852886 |
| Cl | -1.813184 | 2.898346 | 0.837370 |
| H | -3.012173 | 2.012109 | -1.688522 |
| H | -2.103728 | 0.499847 | -1.941936 |
| H | -1.248163 | 2.052296 | -1.843219 |
| Cl | 1.591780 | 1.870866 | -0.839256 |
| B | 2.037193 | 0.147979 | -0.246251 |
| Cl | 1.726996 | 0.014645 | 1.595370 |
| Cl | 3.852262 | -0.186395 | -0.602763 |

| IM2+BBr$_3$ | | x | y | z |
|---|---|---|---|---|
| | B | -1.854531 | -0.634695 | -0.001190 |
| | C | -1.199511 | 1.897330 | -0.242465 |
| | C | -1.411016 | 0.805193 | 0.514121 |
| | Br | -0.465221 | -1.473926 | -1.258284 |
| | Br | -2.053287 | -1.866888 | 1.621901 |
| | Br | -3.661194 | -0.578574 | -0.971509 |
| | C | -1.317293 | 2.061307 | -1.723065 |
| | H | -1.280075 | 0.918965 | 1.589661 |
| | Br | -0.702490 | 3.548904 | 0.644665 |
| | H | -2.130870 | 2.754046 | -1.969148 |
| | H | -1.520327 | 1.095912 | -2.188541 |
| | H | -0.390800 | 2.471383 | -2.139043 |
| | Br | 2.397181 | 1.383623 | -0.960832 |
| | B | 2.493210 | -0.192172 | 0.096204 |
| | Br | 1.802013 | -0.199465 | 1.861644 |
| | Br | 3.417157 | -1.719072 | -0.567858 |
| TS3-Br | | x | y | z |
| | B | -1.612840 | -0.864999 | 0.015059 |
| | C | -1.572463 | 1.744510 | -0.241712 |
| | C | -1.562878 | 0.636992 | 0.523827 |
| | Br | 0.124667 | -1.338050 | -1.052994 |
| | Br | -1.708579 | -2.120939 | 1.608796 |
| | Br | -3.178913 | -1.269671 | -1.221982 |
| | C | -1.636419 | 1.869490 | -1.729732 |
| | H | -1.513433 | 0.782656 | 1.602306 |
| | Br | -1.529125 | 3.467670 | 0.639712 |
| | H | -2.598946 | 2.295288 | -2.037966 |
| | H | -1.519500 | 0.889349 | -2.194052 |
| | H | -0.844617 | 2.533184 | -2.090907 |
| | Br | 1.970705 | 1.704805 | -0.883501 |
| | B | 2.159086 | 0.013334 | 0.025409 |
| | Br | 1.701646 | -0.053718 | 1.893745 |
| | Br | 3.544622 | -1.182900 | -0.606519 |
| E+BBr$_4^-$ | | x | y | z |
| | B | -1.551406 | -0.989977 | 0.062182 |
| | C | -1.804739 | 1.597260 | -0.250542 |
| | C | -1.669238 | 0.505105 | 0.529191 |
| | Br | 0.448204 | -1.240927 | -0.944574 |
| | Br | -1.534654 | -2.259366 | 1.600172 |
| | Br | -2.796043 | -1.630718 | -1.369640 |
| | C | -1.852311 | 1.705183 | -1.740190 |
| | H | -1.638875 | 0.667920 | 1.605533 |
| | Br | -1.990518 | 3.308826 | 0.620491 |
| | H | -2.866980 | 1.954618 | -2.073840 |
| | H | -1.552645 | 0.762557 | -2.199500 |
| | H | -1.179839 | 2.497073 | -2.081782 |
| | Br | 1.703862 | 1.849416 | -0.882616 |
| | B | 1.891819 | 0.045339 | -0.057013 |
| | Br | 1.603917 | 0.090838 | 1.906551 |
| | Br | 3.636489 | -0.803905 | -0.544870 |

| $IM2+BI_3$ | | x | y | z |
|---|---|---|---|---|
| | B | -2.033718 | -0.589788 | -0.055081 |
| | C | -1.286979 | 1.919106 | -0.363693 |
| | C | -1.525874 | 0.842579 | 0.413823 |
| | I | -0.543256 | -1.662270 | -1.429295 |
| | I | -2.316560 | -1.877719 | 1.824018 |
| | I | -4.069000 | -0.447102 | -1.116570 |
| | C | -1.408683 | 2.053643 | -1.847957 |
| | H | -1.402565 | 0.974805 | 1.487668 |
| | I | -0.763530 | 3.779499 | 0.612542 |
| | H | -2.267232 | 2.684954 | -2.108303 |
| | H | -1.544814 | 1.069667 | -2.301124 |
| | H | -0.511421 | 2.517113 | -2.271396 |
| | I | 2.575860 | 1.532768 | -1.188154 |
| | B | 2.704558 | -0.144380 | 0.125761 |
| | I | 1.828152 | -0.061892 | 2.064240 |
| | I | 3.810995 | -1.875882 | -0.471938 |

| TS3-I | | x | y | z |
|---|---|---|---|---|
| | B | -1.882821 | -0.690120 | -0.041578 |
| | C | -1.456681 | 1.889199 | -0.366071 |
| | C | -1.606928 | 0.801769 | 0.419887 |
| | I | -0.041983 | -1.549846 | -1.159082 |
| | I | -2.230292 | -1.972993 | 1.815862 |
| | I | -3.705130 | -0.905297 | -1.403825 |
| | C | -1.515399 | 1.989473 | -1.857514 |
| | H | -1.549765 | 0.960739 | 1.495428 |
| | I | -1.192557 | 3.802196 | 0.605600 |
| | H | -2.468644 | 2.429982 | -2.175815 |
| | H | -1.423060 | 0.996682 | -2.303335 |
| | H | -0.708846 | 2.623558 | -2.236917 |
| | I | 2.312147 | 1.657985 | -1.192155 |
| | B | 2.318957 | -0.127104 | 0.033444 |
| | I | 1.682120 | 0.056067 | 2.088429 |
| | I | 3.768972 | -1.673159 | -0.451365 |

| $E+BI_4^-$ | | x | y | z |
|---|---|---|---|---|
| | B | -1.807227 | -0.782605 | 0.000613 |
| | C | -1.664647 | 1.817994 | -0.379905 |
| | C | -1.693250 | 0.730153 | 0.422465 |
| | I | 0.270235 | -1.451211 | -1.025664 |
| | I | -2.093014 | -2.090853 | 1.815076 |
| | I | -3.361634 | -1.312827 | -1.553691 |
| | C | -1.708486 | 1.892419 | -1.873260 |
| | H | -1.648465 | 0.908431 | 1.495485 |
| | I | -1.628910 | 3.755029 | 0.567697 |
| | H | -2.710390 | 2.183408 | -2.213676 |
| | H | -1.460606 | 0.922538 | -2.309605 |
| | H | -0.996780 | 2.635484 | -2.242541 |
| | I | 2.055126 | 1.815991 | -1.135144 |
| | B | 1.977143 | -0.154618 | -0.072348 |
| | I | 1.606254 | 0.104695 | 2.112795 |
| | I | 3.838075 | -1.360581 | -0.467612 |

**Table S22.** Cartesian coordinates (in Å) for structures referring to Figure S10

| $C_3H_4$+$BCl_3$+$Cl^-$, MP2 | x | y | z |
|---|---|---|---|
| B | -2.110489 | 0.210660 | 0.100769 |
| C | 1.080634 | -0.705082 | -0.347643 |
| C | 0.521410 | -1.791997 | -0.424330 |
| Cl | -1.764124 | 1.328626 | -1.185857 |
| Cl | -3.051237 | -1.217676 | -0.221000 |
| Cl | -1.599181 | 0.566920 | 1.723644 |
| C | 1.763924 | 0.587759 | -0.273265 |
| H | 0.052581 | -2.751566 | -0.475254 |
| Cl | 5.501605 | -0.067609 | 0.095247 |
| H | 1.574844 | 1.173130 | -1.178101 |
| H | 2.843787 | 0.443955 | -0.169834 |
| H | 1.405349 | 1.162673 | 0.586201 |
| **$C_3H_4$+$BCl_3$+$Cl^-$, B3LYP** | **x** | **y** | **z** |
| B | -2.107445 | 0.283384 | -0.000030 |
| C | 0.967846 | -0.975105 | 0.000125 |
| C | 0.303841 | -1.988881 | 0.000090 |
| Cl | -1.628817 | 1.018554 | 1.518125 |
| Cl | -1.629105 | 1.017927 | -1.518580 |
| Cl | -3.162292 | -1.119638 | 0.000357 |
| C | 1.758884 | 0.251725 | 0.000138 |
| H | -0.274714 | -2.886203 | 0.000027 |
| Cl | 5.639399 | 0.024024 | -0.000045 |
| H | 1.533839 | 0.856984 | -0.885670 |
| H | 2.834422 | 0.034317 | -0.000029 |
| H | 1.534104 | 0.856795 | 0.886142 |
| **TS1-Cl, MP2** | **x** | **y** | **z** |
| B | -1.635179 | -0.024461 | 0.009947 |
| C | 0.924893 | -0.171531 | -0.089142 |
| C | 0.160548 | -1.134525 | -0.180976 |
| Cl | -1.421408 | 1.748152 | -0.001386 |
| Cl | -2.411399 | -0.692304 | -1.458212 |
| Cl | -2.137090 | -0.725759 | 1.576132 |
| C | 1.736845 | 1.024078 | 0.025651 |
| H | -0.173155 | -2.149089 | -0.267888 |
| Cl | 5.119309 | -0.329835 | -0.027192 |
| H | 1.544718 | 1.705429 | -0.807133 |
| H | 2.791824 | 0.716963 | 0.002268 |
| H | 1.538783 | 1.536572 | 0.971014 |
| **TS1-Cl, B3LYP** | **x** | **y** | **z** |
| B | -1.755568 | 0.018959 | -0.000132 |
| C | 0.948283 | -0.301472 | 0.000859 |
| C | 0.164935 | -1.237963 | 0.001381 |
| Cl | -1.396146 | 1.772522 | -0.001719 |
| Cl | -2.381909 | -0.668278 | -1.533362 |
| Cl | -2.382443 | -0.665371 | 1.534178 |
| C | 1.811376 | 0.860902 | 0.000183 |
| H | -0.249690 | -2.223403 | 0.002086 |
| Cl | 5.297624 | -0.278737 | -0.000035 |
| H | 1.642694 | 1.473416 | -0.892065 |
| H | 2.863324 | 0.529709 | 0.000263 |
| H | 1.642817 | 1.474383 | 0.891788 |

| IM1+Cl⁻, MP2 | x | y | z |
|---|---|---|---|
| B | -1.518859 | -0.066011 | -0.000238 |
| C | 0.900834 | -0.111423 | -0.006621 |
| C | 0.103584 | -1.057097 | -0.022447 |
| Cl | -1.392958 | 1.738896 | -0.047396 |
| Cl | -2.288809 | -0.746374 | -1.494160 |
| Cl | -2.217672 | -0.679526 | 1.555380 |
| C | 1.746225 | 1.055042 | 0.013140 |
| H | -0.144055 | -2.102177 | -0.031545 |
| Cl | 5.033430 | -0.365143 | -0.009459 |
| H | 1.586201 | 1.672101 | -0.874243 |
| H | 2.785803 | 0.685277 | 0.007828 |
| H | 1.584665 | 1.642200 | 0.920516 |

| IM1+Cl⁻, B3LYP | x | y | z |
|---|---|---|---|
| B | 1.436566 | -0.114799 | -0.000535 |
| C | -1.081386 | 0.239591 | -0.001273 |
| C | -0.132149 | -0.609607 | -0.001743 |
| Cl | 1.562415 | 1.758685 | -0.022911 |
| Cl | 2.230658 | -0.796120 | 1.553150 |
| Cl | 2.246743 | -0.834719 | -1.528225 |
| C | -2.140596 | 1.133635 | 0.000395 |
| H | -0.318976 | -1.683470 | -0.000731 |
| Cl | -4.814278 | -0.492866 | -0.001030 |
| H | -2.238292 | 1.723588 | 0.916904 |
| H | -3.095102 | 0.431890 | 0.000111 |
| H | -2.239835 | 1.725598 | -0.914630 |

| TS4-Cl, MP2 | x | y | z |
|---|---|---|---|
| B | 1.369241 | -0.110806 | -0.001708 |
| C | -1.124277 | 0.402548 | -0.001386 |
| C | -0.214422 | -0.502383 | -0.010436 |
| Cl | 1.611987 | 1.727727 | -0.056164 |
| Cl | 2.099895 | -0.807943 | 1.554515 |
| Cl | 2.142391 | -0.902921 | -1.490030 |
| C | -2.150997 | 1.298877 | 0.003080 |
| H | -0.476936 | -1.561998 | -0.012032 |
| Cl | -4.542494 | -0.559466 | -0.004667 |
| H | -2.324320 | 1.846346 | 0.930387 |
| H | -3.081323 | 0.445420 | -0.000255 |
| H | -2.325691 | 1.854262 | -0.919223 |

| TS4-Cl, B3LYP | x | y | z |
|---|---|---|---|
| B | 1.382817 | -0.107953 | -0.001526 |
| C | -1.139388 | 0.371795 | -0.003083 |
| C | -0.192090 | -0.495872 | -0.005151 |
| Cl | 1.638522 | 1.755407 | -0.048341 |
| Cl | 2.151360 | -0.815237 | 1.566933 |
| Cl | 2.182871 | -0.897609 | -1.513371 |
| C | -2.207142 | 1.214351 | 0.001951 |
| H | -0.448341 | -1.557696 | -0.004331 |
| Cl | -4.633019 | -0.533109 | -0.002842 |
| H | -2.404206 | 1.762525 | 0.926673 |
| H | -3.196575 | 0.363124 | 0.000101 |
| H | -2.408714 | 1.769469 | -0.917566 |

| IM3+HCl, MP2 | x | y | z |
|---|---|---|---|
| B | 1.271566 | -0.117570 | 0.008413 |
| C | -1.120865 | 0.890439 | 0.085113 |
| C | -0.317267 | -0.147845 | 0.154945 |
| Cl | 1.919916 | 1.580182 | -0.384451 |
| Cl | 2.033199 | -0.718660 | 1.616629 |
| Cl | 1.769805 | -1.301751 | -1.360466 |
| C | -1.933966 | 1.930963 | 0.010740 |
| H | -0.769154 | -1.127010 | 0.341154 |
| Cl | -4.395048 | -0.692060 | 0.017693 |
| H | -2.179689 | 2.520409 | 0.891445 |
| H | -3.402420 | 0.142344 | 0.041216 |
| H | -2.347796 | 2.259663 | -0.940553 |

| IM3+HCl, B3LYP | x | y | z |
|---|---|---|---|
| B | 1.291001 | -0.110069 | -0.001330 |
| C | -1.154552 | 0.749675 | -0.002109 |
| C | -0.300317 | -0.237493 | 0.002276 |
| Cl | 1.885983 | 1.676771 | -0.076360 |
| Cl | 1.981612 | -0.921368 | 1.578512 |
| Cl | 1.984852 | -1.056857 | -1.502296 |
| C | -2.030895 | 1.734030 | -0.003634 |
| H | -0.706100 | -1.252758 | 0.014886 |
| Cl | -4.478324 | -0.651526 | 0.001282 |
| H | -2.374042 | 2.189545 | 0.924581 |
| H | -3.434296 | 0.159469 | 0.001785 |
| H | -2.386070 | 2.177464 | -0.933131 |

| $C_3H_4$+$BBr_3$+$Br^-$, MP2 | x | y | z |
|---|---|---|---|
| B | -1.961158 | -0.054459 | 0.244805 |
| C | 1.289362 | -0.029299 | -0.883160 |
| C | 0.656320 | 0.422909 | -1.831203 |
| Br | -1.345835 | 1.211700 | 1.519357 |
| Br | -2.995369 | 0.524074 | -1.243552 |
| Br | -1.647317 | -1.908547 | 0.513914 |
| C | 1.971017 | -0.603990 | 0.277263 |
| H | 0.182049 | 0.848525 | -2.689511 |
| Br | 5.716994 | -0.078051 | 0.253306 |
| H | 1.573733 | -0.171274 | 1.201326 |
| H | 3.047481 | -0.409632 | 0.236674 |
| H | 1.815929 | -1.687216 | 0.311473 |

| $C_3H_4$+$BBr_3$+$Br^-$, B3LYP | x | Y | z |
|---|---|---|---|
| B | -1.844824 | -0.036601 | -0.440232 |
| C | 1.170047 | 0.019939 | 1.115007 |
| C | 0.417544 | -0.001613 | 2.065227 |
| Br | -1.259114 | -1.716250 | -1.132702 |
| Br | -3.119782 | 0.015006 | 0.985629 |
| Br | -1.248229 | 1.589809 | -1.242210 |
| C | 2.060385 | 0.045663 | -0.039858 |
| H | -0.241264 | -0.021065 | 2.904749 |
| Br | 5.424670 | -0.046382 | -1.455605 |
| H | 1.821872 | -0.770624 | -0.731403 |
| H | 3.115523 | -0.055967 | 0.231680 |
| H | 1.955841 | 0.987482 | -0.590145 |

| TS1-Br, MP2 | x | Y | z |
|---|---|---|---|
| B | -1.584383 | 0.020331 | 0.001408 |
| C | 1.027391 | -0.051874 | -0.472744 |
| C | 0.288816 | 0.419198 | -1.342442 |
| Br | -1.331062 | 1.255025 | 1.471605 |
| Br | -2.829709 | 0.575788 | -1.388546 |
| Br | -1.558429 | -1.876510 | 0.396460 |
| C | 1.761413 | -0.634705 | 0.636109 |
| H | -0.069792 | 0.883740 | -2.236991 |
| Br | 5.252231 | 0.129993 | -0.292192 |
| H | 1.481185 | -0.141054 | 1.572841 |
| H | 2.835343 | -0.484575 | 0.462079 |
| H | 1.553364 | -1.705855 | 0.713057 |

| TS1-Br, B3LYP | x | Y | z |
|---|---|---|---|
| B | -1.610392 | -0.000001 | -0.000214 |
| C | 1.051065 | -0.000010 | 0.460944 |
| C | 0.306622 | -0.000253 | 1.428623 |
| Br | -1.451435 | -1.666505 | -0.994866 |
| Br | -2.866824 | -0.000467 | 1.500727 |
| Br | -1.451612 | 1.667023 | -0.994028 |
| C | 1.834317 | 0.000313 | -0.753530 |
| H | -0.100746 | -0.000364 | 2.415197 |
| Br | 5.279990 | -0.000083 | 0.315731 |
| H | 1.618704 | -0.893144 | -1.351103 |
| H | 2.909113 | 0.000231 | -0.493351 |
| H | 1.618698 | 0.894086 | -1.350626 |

| IM1+$Br^-$ | x | y | z |
|---|---|---|---|
| B | -1.090651 | -0.040024 | -0.004460 |
| C | 1.238631 | 1.090181 | -0.020335 |
| C | 0.496689 | 0.014761 | -0.035754 |
| Br | -1.754511 | -1.022054 | -1.702073 |
| Br | -1.678206 | -1.146366 | 1.645244 |
| Br | -1.965136 | 1.805704 | 0.081890 |
| C | 2.017635 | 2.151948 | -0.005353 |
| H | 1.016203 | -0.946996 | -0.072909 |
| Br | 4.648000 | -0.329699 | -0.011391 |
| H | 2.312084 | 2.654072 | -0.926179 |
| H | 3.507965 | 0.581235 | -0.018441 |
| H | 2.344168 | 2.604970 | 0.930054 |

| TS4-Br | x | y | z |
|---|---|---|---|
| B | -1.234534 | -0.101589 | 0.003391 |
| C | 1.217301 | 0.481257 | 0.006140 |
| C | 0.350040 | -0.463573 | 0.017547 |
| Br | -2.009314 | -0.895058 | -1.688538 |
| Br | -2.058478 | -0.963008 | 1.638100 |
| Br | -1.539768 | 1.901433 | 0.038638 |
| C | 2.219367 | 1.417698 | -0.005789 |
| H | 0.647646 | -1.513975 | 0.025573 |
| Br | 4.892019 | -0.363738 | 0.008220 |
| H | 2.339815 | 1.977255 | -0.935183 |
| H | 3.165461 | 0.646964 | 0.000628 |
| H | 2.343461 | 1.998423 | 0.909940 |

| IM3+HBr, MP2 | x | y | z |
|---|---|---|---|
| B | 1.103214 | -0.043519 | 0.006470 |
| C | -1.198380 | 1.146982 | 0.038183 |
| C | -0.483120 | 0.041769 | 0.054491 |
| Br | 1.750680 | -1.008796 | 1.696108 |
| Br | 1.639728 | -1.165207 | -1.624528 |
| Br | 1.994676 | 1.771859 | -0.109810 |
| C | -1.946281 | 2.238394 | 0.020516 |
| H | -1.023215 | -0.908910 | 0.106836 |
| Br | -4.662866 | -0.324401 | 0.013375 |
| H | -2.214566 | 2.755433 | 0.939795 |
| H | -3.518392 | 0.550560 | 0.028485 |
| H | -2.270824 | 2.686729 | -0.916687 |

| IM3+HBr, B3LYP | x | y | z |
|---|---|---|---|
| B | -1.294404 | -0.116386 | -0.006933 |
| C | 1.130711 | 0.241045 | -0.004316 |
| C | 0.279546 | -0.692431 | -0.049057 |
| Br | -2.138916 | -0.778599 | -1.707988 |
| Br | -2.116382 | -0.970487 | 1.618242 |
| Br | -1.425499 | 1.891984 | 0.109520 |
| C | 2.050904 | 1.302391 | 0.043549 |
| H | 0.400916 | -1.769074 | -0.107324 |
| Br | 5.058863 | -0.351628 | -0.018662 |
| H | 2.013852 | 1.941402 | -0.841860 |
| H | 3.044645 | 0.749725 | 0.021095 |
| H | 2.013322 | 1.859427 | 0.982753 |

| $C_3H_4$+$BI_3$+$I^-$, MP2 | x | y | z |
|---|---|---|---|
| B | 1.872229 | 0.080306 | -0.435853 |
| C | -1.188653 | 0.006774 | 1.505732 |
| C | -0.349404 | -0.291906 | 2.347874 |
| I | 1.011499 | -1.588910 | -1.448461 |
| I | 1.254176 | 2.063756 | -0.921978 |
| I | 3.416586 | -0.229825 | 1.011595 |
| C | -2.150834 | 0.393496 | 0.472555 |
| H | 0.340510 | -0.578369 | 3.112523 |
| I | -5.467180 | 0.746347 | -1.740019 |
| H | -1.883582 | -0.049269 | -0.491113 |
| H | -2.178049 | 1.480322 | 0.353400 |
| H | -3.159515 | 0.058428 | 0.724470 |

| $C_3H_4$+$BI_3$+$I^-$,B3LYP/ECP46MWB | x | y | z |
|---|---|---|---|
| B | 2.124950 | -0.006360 | -0.206416 |
| C | -1.115954 | 0.101588 | 1.217229 |
| C | -0.431958 | -0.141013 | 2.187524 |
| I | 1.387897 | -1.717752 | -1.253246 |
| I | 1.624461 | 1.960933 | -0.876256 |
| I | 3.446829 | -0.261846 | 1.459085 |
| C | -1.931647 | 0.391910 | 0.042290 |
| H | 0.167148 | -0.357009 | 3.044129 |
| I | -6.147331 | -0.040514 | 0.257796 |
| H | -1.630185 | -0.238061 | -0.803070 |
| H | -1.817436 | 1.438587 | -0.262091 |
| H | -2.995308 | 0.209818 | 0.239778 |

| $C_3H_4$+$BI_3$+$I^-$, B3LYP/SVP | x | y | z |
|---|---|---|---|
| B | -1.878785 | -0.052267 | -0.397340 |
| C | 1.150071 | -0.122460 | 1.358209 |
| C | 0.381409 | 0.226051 | 2.227979 |
| I | -0.948344 | 1.492908 | -1.546385 |
| I | -1.417953 | -2.103781 | -0.790936 |
| I | -3.342554 | 0.450557 | 1.086634 |
| C | 2.067360 | -0.536941 | 0.302135 |
| H | -0.295198 | 0.533430 | 2.993953 |
| I | 5.646355 | -0.377604 | -1.432927 |
| H | 1.662715 | -0.284786 | -0.684801 |
| H | 2.234738 | -1.619432 | 0.328507 |
| H | 3.044479 | -0.050894 | 0.386872 |
| TS1-I, MP2 | x | y | z |
| B | 1.634281 | -0.018438 | 0.000826 |
| C | -1.097219 | 0.155132 | 0.440886 |
| C | -0.380900 | -0.285630 | 1.344418 |
| I | 1.324083 | -1.527154 | -1.530189 |
| I | 1.650677 | 2.071707 | -0.594333 |
| I | 2.955428 | -0.553211 | 1.656272 |
| C | -1.806456 | 0.680800 | -0.712671 |
| H | -0.046258 | -0.711691 | 2.266616 |
| I | -5.598656 | -0.084297 | 0.360945 |
| H | -1.464316 | 0.179578 | -1.625337 |
| H | -1.645665 | 1.758506 | -0.808903 |
| H | -2.878928 | 0.490608 | -0.585173 |
| TS1-I, B3LYP/ECP46MWB | x | y | z |
| B | 1.662411 | -0.012556 | 0.011390 |
| C | -1.050826 | 0.135388 | 0.471096 |
| C | -0.337070 | -0.174752 | 1.410359 |
| I | 1.359484 | -1.578605 | -1.475412 |
| I | 1.671181 | 2.060124 | -0.668776 |
| I | 3.024723 | -0.492519 | 1.663942 |
| C | -1.820815 | 0.501122 | -0.699006 |
| H | 0.041687 | -0.461190 | 2.366156 |
| I | -5.734733 | -0.064873 | 0.357414 |
| H | -1.582637 | -0.162766 | -1.538460 |
| H | -1.619972 | 1.537003 | -0.993492 |
| H | -2.893592 | 0.400455 | -0.475773 |
| TS1-I, B3LYP/SVP | x | y | z |
| B | -1.592323 | 0.016026 | 0.006408 |
| C | 1.106759 | -0.188409 | 0.452546 |
| C | 0.424768 | 0.253669 | 1.362744 |
| I | -1.250269 | 1.405646 | -1.637662 |
| I | -1.685873 | -2.113792 | -0.453910 |
| I | -2.909787 | 0.717349 | 1.612227 |
| C | 1.833572 | -0.706661 | -0.685047 |
| H | 0.069151 | 0.662128 | 2.282059 |
| I | 5.498842 | 0.096596 | 0.363163 |
| H | 1.591678 | -0.129050 | -1.585554 |
| H | 1.590822 | -1.760809 | -0.858855 |
| H | 2.915006 | -0.611330 | -0.493528 |

| IM1+I$^-$, MP2 | x | y | z |
|---|---|---|---|
| B | -0.386189 | -0.000102 | -1.042295 |
| C | -0.226781 | -0.000084 | 1.417657 |
| C | 0.474486 | -0.000106 | 0.357967 |
| I | -2.627605 | 0.000313 | -0.692325 |
| I | 0.243007 | 1.870933 | -2.135455 |
| I | 0.242375 | -1.871577 | -2.135061 |
| C | -0.949765 | -0.000104 | 2.623321 |
| H | 1.562126 | -0.000167 | 0.303811 |
| I | 1.836964 | -0.000066 | 5.083394 |
| H | -0.097501 | -0.000119 | 3.371400 |
| H | -1.524754 | -0.914232 | 2.788124 |
| H | -1.524746 | 0.914020 | 2.788168 |
| IM1+I$^-$, B3LYP/ECP46MWB | x | y | z |
| B | -0.317450 | -0.000388 | -1.126429 |
| C | -0.216999 | -0.000973 | 1.396336 |
| C | 0.437873 | -0.000281 | 0.297599 |
| I | -2.595201 | -0.003112 | -0.829267 |
| I | 0.330382 | 1.881307 | -2.237418 |
| I | 0.334644 | -1.879329 | -2.239565 |
| C | -0.823278 | -0.001547 | 2.650931 |
| H | 1.530552 | 0.000490 | 0.315393 |
| I | 1.632423 | -0.001382 | 5.210959 |
| H | 0.076968 | -0.001315 | 3.387667 |
| H | -1.378833 | -0.914876 | 2.884380 |
| H | -1.379727 | 0.911140 | 2.884765 |
| IM1+I$^-$, B3LYP/SVP | x | y | z |
| B | 1.220034 | -0.099011 | 0.005517 |
| C | -1.240670 | 0.508218 | -0.012427 |
| C | -0.356383 | -0.420078 | 0.025795 |
| I | 2.100202 | -1.114056 | -1.836711 |
| I | 1.568580 | 2.161694 | -0.086904 |
| I | 2.122387 | -0.960321 | 1.912701 |
| C | -2.304677 | 1.393026 | -0.050199 |
| H | -0.682985 | -1.462681 | 0.070762 |
| I | -5.299053 | -0.305754 | 0.016508 |
| H | -2.412582 | 1.947147 | -0.987243 |
| H | -2.418118 | 2.019606 | 0.839264 |
| H | -3.258294 | 0.681089 | -0.025908 |
| TS4-I, MP2 | x | y | z |
| B | -1.117373 | -0.058517 | 0.000005 |
| C | 1.278472 | 0.830341 | 0.000022 |
| C | 0.476018 | -0.185823 | 0.000027 |
| I | -1.758478 | 2.121427 | -0.000042 |
| I | -1.878950 | -1.108205 | -1.867519 |
| I | -1.879010 | -1.108145 | 1.867539 |
| C | 2.284410 | 1.739906 | 0.000007 |
| H | 0.928211 | -1.184876 | 0.000036 |
| I | 4.992376 | -0.249515 | 0.000014 |
| H | 3.243005 | 0.857357 | 0.000016 |
| H | 2.478775 | 2.284387 | 0.925251 |
| H | 2.478762 | 2.284365 | -0.925252 |

| TS4-I, B3LYP/ECP46MWB | x | y | z |
|---|---|---|---|
| B | 0.004700 | 0.000337 | 0.010177 |
| C | -0.043413 | 0.000359 | 2.588331 |
| C | 0.619280 | 0.000511 | 1.468389 |
| I | -2.284738 | -0.001715 | 0.008316 |
| I | 0.774166 | 1.882533 | -1.089885 |
| I | 0.777721 | -1.879851 | -1.090833 |
| C | -0.616922 | 0.000260 | 3.787019 |
| H | 1.711276 | 0.000831 | 1.543687 |
| I | 2.177190 | -0.000035 | 5.792929 |
| H | 0.811495 | 0.000234 | 4.650602 |
| H | -0.988385 | -0.927896 | 4.221382 |
| H | -0.988690 | 0.928302 | 4.221370 |

| TS4-I, B3LYP/SVP | x | y | z |
|---|---|---|---|
| B | 1.131116 | -0.055317 | -0.000205 |
| C | -1.317187 | 0.730689 | -0.000360 |
| C | -0.451424 | -0.232530 | -0.000964 |
| I | 1.743983 | 2.143167 | -0.000441 |
| I | 1.951657 | -1.095241 | 1.876120 |
| I | 1.953739 | -1.096255 | -1.875029 |
| C | -2.338737 | 1.606865 | 0.000264 |
| H | -0.845751 | -1.253818 | -0.001735 |
| I | -5.112884 | -0.254777 | -0.000500 |
| H | -3.451038 | 0.688361 | -0.000160 |
| H | -2.574525 | 2.138879 | -0.923320 |
| H | -2.574416 | 2.137614 | 0.924607 |

| IM3+HI, MP2 | x | y | z |
|---|---|---|---|
| B | -1.005997 | 0.021662 | 0.009766 |
| C | 1.125360 | 1.504399 | 0.084623 |
| C | 0.546764 | 0.320256 | 0.074558 |
| I | -2.276503 | 1.897086 | -0.070706 |
| I | -1.388896 | -1.255622 | -1.859030 |
| I | -1.561284 | -1.208449 | 1.866786 |
| C | 1.750976 | 2.669541 | 0.094257 |
| H | 1.204266 | -0.555290 | 0.112398 |
| I | 4.770060 | -0.071606 | 0.026365 |
| H | 3.489755 | 0.970109 | 0.062399 |
| H | 1.961995 | 3.190841 | 1.026030 |
| H | 2.036445 | 3.166155 | -0.831234 |

| IM3+HI, B3LYP/ECP46MWB | x | y | z |
|---|---|---|---|
| B | -0.329024 | -0.000853 | -0.987033 |
| C | -0.455052 | -0.000387 | 1.594308 |
| C | 0.236375 | -0.000663 | 0.487141 |
| I | -2.615753 | -0.006357 | -1.082812 |
| I | 0.479072 | 1.883374 | -2.068792 |
| I | 0.488709 | -1.879156 | -2.071877 |
| C | -1.085582 | 0.000032 | 2.754198 |
| H | 1.325986 | -0.000468 | 0.590215 |
| I | 1.980123 | -0.004693 | 4.663917 |
| H | 0.602688 | -0.001885 | 3.652466 |
| H | -1.418970 | -0.928743 | 3.215605 |
| H | -1.418490 | 0.929245 | 3.215084 |

| IM3+HI, B3LYP/SVP | x | y | z |
|---|---|---|---|
| B | 1.002479 | 0.018178 | 0.000876 |
| C | -1.204415 | 1.375253 | 0.022165 |
| C | -0.563600 | 0.235107 | 0.023514 |
| I | 2.192218 | 1.960574 | -0.111708 |
| I | 1.592975 | -1.133000 | 1.920516 |
| I | 1.517753 | -1.305055 | -1.825321 |
| C | -1.892323 | 2.496751 | 0.023568 |
| H | -1.175267 | -0.671974 | 0.047900 |
| I | -4.812971 | -0.106829 | 0.006414 |
| H | -3.505719 | 0.918216 | 0.019566 |
| H | -2.186637 | 2.985230 | -0.904452 |
| H | -2.151411 | 3.003431 | 0.952366 |

**Table S23.** Cartesian coordinates (in Å) for structures referring to Figure S11

| IM3+HI | x | y | z |
|---|---|---|---|
| B | 0.946323 | -0.059480 | 0.294080 |
| C | -0.474009 | -1.424690 | 1.979283 |
| C | 0.239103 | -0.356435 | 1.675576 |
| I | 0.186758 | 1.991007 | -0.448222 |
| I | 3.205160 | 0.113468 | 0.660632 |
| I | 0.539765 | -1.624430 | -1.289463 |
| C | -1.172407 | -2.492744 | 2.314662 |
| H | 0.362802 | 0.401604 | 2.455806 |
| I | -3.769936 | 0.098676 | 0.234990 |
| H | -0.692455 | -3.371981 | 2.739158 |
| H | -2.283489 | 0.774436 | -0.005032 |
| H | -2.247164 | -2.535722 | 2.151945 |
| TS5 | x | y | z |
| B | -1.420579 | 0.582468 | 0.519692 |
| C | 0.275742 | 1.536154 | 2.082597 |
| C | -0.567974 | 0.551622 | 1.788517 |
| I | 0.688566 | -2.321076 | -0.384293 |
| I | -3.233340 | -0.589351 | 0.477596 |
| I | -0.983724 | 1.898205 | -1.124332 |
| C | 1.062816 | 2.528095 | 2.428870 |
| H | -0.696820 | -0.256158 | 2.510821 |
| I | 3.497230 | 0.346697 | 0.126977 |
| H | 0.673199 | 3.388415 | 2.968373 |
| H | 2.045219 | -0.980873 | -0.119225 |
| H | 2.114920 | 2.513911 | 2.156396 |
| IM4+HI+I$^-$ | x | y | z |
| B | 1.606140 | -0.602334 | 0.561725 |
| C | -0.242320 | -1.638736 | 1.881868 |
| C | 0.654085 | -0.663351 | 1.753948 |
| I | -0.801403 | 2.249547 | -0.541968 |
| I | 3.276941 | 0.759736 | 0.658653 |
| I | 1.401085 | -1.888706 | -1.148808 |
| C | -1.112346 | -2.607029 | 2.038694 |
| H | 0.728468 | 0.079920 | 2.549849 |
| I | -3.868251 | -0.417072 | 0.213810 |
| H | -0.835508 | -3.537109 | 2.529958 |
| H | -2.040020 | 1.123229 | -0.221257 |
| H | -2.123832 | -2.485449 | 1.656334 |

| TS6 | | x | y | z |
|---|---|---|---|---|
| | B | 1.151308 | -0.735349 | 0.471281 |
| | C | -1.024906 | -1.816385 | 1.070407 |
| | C | -0.000497 | -0.946321 | 1.378702 |
| | I | -0.651508 | 2.581846 | -0.671821 |
| | I | 2.881199 | 0.397218 | 1.193671 |
| | I | 1.272065 | -1.574132 | -1.536763 |
| | C | -1.324290 | -3.108157 | 0.929996 |
| | H | -0.067378 | -0.394205 | 2.318954 |
| | I | -3.258391 | -0.550624 | 0.514689 |
| | H | -0.574999 | -3.834507 | 1.243993 |
| | H | -1.653045 | 1.327949 | -0.214553 |
| | H | -2.261263 | -3.475660 | 0.532418 |
| IM5 | | x | y | z |
| | B | -0.695564 | 0.013179 | -0.187919 |
| | C | 1.677842 | -0.911545 | -0.977060 |
| | C | 0.460853 | -0.093060 | -1.311753 |
| | I | 0.077309 | 0.795110 | 1.808177 |
| | I | -2.261978 | 1.483810 | -1.010557 |
| | I | -1.735716 | -1.996626 | 0.169210 |
| | C | 1.772571 | -2.251515 | -1.032913 |
| | H | 0.003399 | -0.529208 | -2.212072 |
| | I | 3.461239 | 0.183730 | -0.461618 |
| | H | 0.899185 | -2.828716 | -1.325326 |
| | H | 0.763897 | 0.924666 | -1.581636 |
| | H | 2.678511 | -2.795231 | -0.787239 |

**Table S24.** Cartesian coordinates (in Å) for structures referring to Figure S12

| IM5 | | x | y | z |
|---|---|---|---|---|
| | B | 0.695691 | 0.013531 | 0.187722 |
| | C | -1.677840 | -0.910694 | 0.976032 |
| | C | -0.461541 | -0.091170 | 1.310815 |
| | I | -0.077641 | 0.786473 | -1.811765 |
| | I | 2.258303 | 1.490198 | 1.006721 |
| | I | 1.740449 | -1.995298 | -0.161563 |
| | C | -1.771521 | -2.250782 | 1.030538 |
| | H | -0.004769 | -0.525195 | 2.212509 |
| | I | -3.462018 | 0.184080 | 0.462072 |
| | H | -0.897529 | -2.827612 | 1.321850 |
| | H | -0.765443 | 0.926899 | 1.578312 |
| | H | -2.677192 | -2.794907 | 0.784791 |
| TS7 | | x | y | z |
| | B | 0.260992 | -0.621570 | -0.147788 |
| | C | -1.594522 | 1.134174 | -0.760037 |
| | C | -0.532325 | 0.192782 | -1.242616 |
| | I | -0.200444 | -0.561267 | 1.952015 |
| | I | 1.594533 | -2.170524 | -0.863883 |
| | I | 2.598160 | 1.966090 | -0.176055 |
| | C | -1.401783 | 2.426056 | -0.452074 |
| | H | 0.224089 | 0.771782 | -1.789922 |
| | I | -3.554881 | 0.283400 | -0.537522 |
| | H | -0.397407 | 2.833802 | -0.547380 |
| | H | -0.948413 | -0.526601 | -1.961239 |
| | H | -2.191956 | 3.072763 | -0.085535 |

| IM6+I$^-$ | | x | y | z |
|---|---|---|---|---|
| | B | 0.184861 | 0.833012 | -0.083859 |
| | C | 0.990920 | -1.628654 | -0.413798 |
| | C | 0.194048 | -0.478773 | -0.938977 |
| | I | 0.496686 | 0.816725 | 2.035041 |
| | I | -0.304428 | 2.686780 | -1.058407 |
| | I | -3.520499 | -1.476359 | -0.180032 |
| | C | 0.481309 | -2.730422 | 0.158023 |
| | H | -0.873688 | -0.782245 | -0.940554 |
| | I | 3.120621 | -1.418674 | -0.613222 |
| | H | -0.600679 | -2.812083 | 0.246987 |
| | H | 0.459750 | -0.255292 | -1.979910 |
| | H | 1.096524 | -3.537304 | 0.542139 |
| TS-rot | | x | y | z |
| | B | 0.662976 | -0.000053 | 0.247088 |
| | C | -1.647760 | -0.000498 | 1.802988 |
| | C | -0.138841 | -0.001039 | 1.638739 |
| | I | 0.303894 | 1.887174 | -0.985418 |
| | I | 2.898615 | -0.000995 | 0.848650 |
| | I | 0.302658 | -1.885895 | -0.986834 |
| | C | -2.218228 | -0.000302 | 3.022748 |
| | H | 0.223005 | -0.879608 | 2.192181 |
| | I | -3.030886 | 0.000011 | 0.151968 |
| | H | -1.578774 | -0.000651 | 3.904694 |
| | H | 0.223730 | 0.875943 | 2.194205 |
| | H | -3.290774 | -0.000019 | 3.183228 |
| IM5b | | x | y | z |
| | B | -0.695720 | -0.013558 | -0.187724 |
| | C | 1.678113 | 0.910431 | -0.975393 |
| | C | 0.461914 | 0.090820 | -1.310457 |
| | I | -1.741859 | 1.994952 | 0.159386 |
| | I | -2.257235 | -1.491863 | -1.005840 |
| | I | 0.077295 | -0.784296 | 1.812724 |
| | C | 1.771440 | 2.250577 | -1.028948 |
| | H | 0.765871 | -0.927339 | -1.577511 |
| | I | 3.462630 | -0.184125 | -0.462113 |
| | H | 0.897306 | 2.827373 | -1.319871 |
| | H | 0.005570 | 0.524584 | -2.212486 |
| | H | 2.677039 | 2.794745 | -0.783017 |
| TS7b | | x | y | z |
| | B | -0.260645 | -0.621648 | -0.147772 |
| | C | 1.594443 | 1.134441 | -0.760247 |
| | C | 0.532233 | 0.193004 | -1.242647 |
| | I | -2.598682 | 1.965759 | -0.176150 |
| | I | -1.593945 | -2.170851 | -0.863776 |
| | I | 0.200497 | -0.560955 | 1.952049 |
| | C | 1.401809 | 2.426454 | -0.452774 |
| | H | 0.948151 | -0.526107 | -1.961638 |
| | I | 3.554756 | 0.283621 | -0.537430 |
| | H | 0.397496 | 2.834308 | -0.548317 |
| | H | -0.224511 | 0.772052 | -1.789466 |
| | H | 2.192018 | 3.073204 | -0.086391 |

| IM6b+I$^-$ | x | y | z |
|---|---|---|---|
| B | -0.210995 | 0.839457 | -0.085578 |
| C | -0.942981 | -1.642722 | -0.425164 |
| C | -0.179768 | -0.468439 | -0.946530 |
| I | 3.565243 | -1.418435 | -0.187049 |
| I | 0.212874 | 2.714141 | -1.049569 |
| I | -0.509589 | 0.797536 | 2.034587 |
| C | -0.401825 | -2.735198 | 0.135200 |
| H | -0.452634 | -0.249027 | -1.986449 |
| I | -3.078535 | -1.485327 | -0.608367 |
| H | 0.682477 | -2.789639 | 0.217172 |
| H | 0.896850 | -0.739292 | -0.949026 |
| H | -0.993887 | -3.560677 | 0.516241 |

**Table S25.** Cartesian coordinates (in Å) for structures referring to Figure S13

| IM5-Cl, MP2 | x | y | z |
|---|---|---|---|
| B | -0.877094 | 0.002443 | -0.061393 |
| C | 1.693401 | -0.578516 | -0.467162 |
| C | 0.341550 | -0.360797 | -1.063868 |
| Cl | -0.726485 | 1.707002 | 0.687288 |
| Cl | -2.463157 | -0.051316 | -1.073768 |
| Cl | -1.001525 | -1.255757 | 1.316161 |
| C | 2.264964 | -1.768805 | -0.222401 |
| H | 0.070481 | -1.297131 | -1.568980 |
| Cl | 2.611456 | 0.874033 | -0.078307 |
| H | 1.723019 | -2.677146 | -0.466642 |
| H | 0.400410 | 0.415425 | -1.838544 |
| H | 3.247149 | -1.862009 | 0.228364 |
| IM5-Cl, B3LYP | x | y | z |
| B | -0.876880 | -0.025602 | 0.069617 |
| C | 1.684736 | 0.563690 | 0.475655 |
| C | 0.360953 | 0.216311 | 1.085322 |
| Cl | -0.591266 | -1.464740 | -1.126197 |
| Cl | -2.421536 | -0.424350 | 1.122101 |
| Cl | -1.246378 | 1.534148 | -0.948664 |
| C | 2.145363 | 1.782575 | 0.189146 |
| H | 0.072141 | 1.046319 | 1.742370 |
| Cl | 2.733415 | -0.834532 | 0.102838 |
| H | 1.524645 | 2.646099 | 0.409420 |
| H | 0.467270 | -0.670100 | 1.720845 |
| H | 3.112026 | 1.951299 | -0.272789 |
| TS-rot-Cl, MP2 | x | y | z |
| B | -0.851553 | 0.096247 | 0.000000 |
| C | 1.803366 | 0.792196 | 0.000216 |
| C | 0.340214 | 1.176417 | 0.000590 |
| Cl | -0.843646 | -0.975905 | 1.532828 |
| Cl | -2.463600 | 1.080280 | 0.000311 |
| Cl | -0.843580 | -0.974836 | -1.533558 |
| C | 2.802409 | 1.690074 | 0.000081 |
| H | 0.181605 | 1.820214 | -0.875300 |
| Cl | 2.257114 | -0.907433 | -0.000032 |
| H | 2.563600 | 2.749919 | 0.000377 |
| H | 0.181611 | 1.818858 | 0.877480 |
| H | 3.848116 | 1.401849 | -0.000193 |

| TS-rot-Cl, B3LYP | x | y | z |
|---|---|---|---|
| B | -0.844416 | 0.101928 | 0.000096 |
| C | 1.816861 | 0.813559 | 0.000352 |
| C | 0.347434 | 1.183228 | 0.000626 |
| Cl | -0.848283 | -0.992193 | 1.549037 |
| Cl | -2.488895 | 1.095053 | 0.000805 |
| Cl | -0.848811 | -0.990110 | -1.550210 |
| C | 2.814900 | 1.701014 | 0.000440 |
| H | 0.184399 | 1.827532 | -0.873794 |
| Cl | 2.276317 | -0.908295 | -0.000305 |
| H | 2.582415 | 2.763283 | 0.000706 |
| H | 0.184579 | 1.827109 | 0.875395 |
| H | 3.859934 | 1.409898 | 0.000154 |
| IM5b-Cl, MP2 | x | y | z |
| B | 0.876773 | 0.001324 | -0.061268 |
| C | -1.694260 | -0.580064 | -0.466705 |
| C | -0.341258 | -0.367536 | -1.062473 |
| Cl | 0.992764 | -1.242283 | 1.330080 |
| Cl | 2.464637 | -0.070228 | -1.069998 |
| Cl | 0.732515 | 1.714661 | 0.668626 |
| C | -2.271483 | -1.768025 | -0.223936 |
| H | -0.398365 | 0.403525 | -1.842493 |
| Cl | -2.606667 | 0.875902 | -0.077458 |
| H | -1.733497 | -2.678654 | -0.468423 |
| H | -0.070594 | -1.307559 | -1.560887 |
| H | -3.254633 | -1.857072 | 0.225569 |
| IM5b-Cl, B3LYP | x | y | z |
| B | 0.876879 | -0.025634 | 0.069648 |
| C | -1.684706 | 0.563651 | 0.475686 |
| C | -0.360959 | 0.216171 | 1.085377 |
| Cl | 1.246394 | 1.534240 | -0.948471 |
| Cl | 2.421560 | -0.424518 | 1.122014 |
| Cl | 0.591212 | -1.464622 | -1.126345 |
| C | -2.145191 | 1.782576 | 0.189106 |
| H | -0.467311 | -0.670307 | 1.720802 |
| Cl | -2.733495 | -0.834479 | 0.102857 |
| H | -1.524379 | 2.646033 | 0.409380 |
| H | -0.072149 | 1.046102 | 1.742523 |
| H | -3.111803 | 1.951408 | -0.272897 |
| IM5-Br, MP2 | x | y | z |
| B | -0.738102 | 0.019622 | -0.145582 |
| C | 1.698125 | -0.825754 | -0.749598 |
| C | 0.443648 | -0.160905 | -1.232319 |
| Br | -0.133871 | 1.043284 | 1.506987 |
| Br | -2.248360 | 1.071777 | -1.056632 |
| Br | -1.489934 | -1.786478 | 0.448344 |
| C | 1.909938 | -2.146092 | -0.623962 |
| H | 0.038281 | -0.763799 | -2.056841 |
| Br | 3.149397 | 0.358081 | -0.292596 |
| H | 1.111614 | -2.833299 | -0.887640 |
| H | 0.684770 | 0.823359 | -1.649153 |
| H | 2.842472 | -2.561090 | -0.256783 |

| IM5-Br, B3LYP | x | y | z |
| --- | --- | --- | --- |
| B | -0.723265 | -0.005065 | 0.150160 |
| C | 1.707119 | 0.876513 | 0.796289 |
| C | 0.428834 | 0.241184 | 1.257440 |
| Br | -0.149322 | -1.273798 | -1.359517 |
| Br | -2.341512 | -0.862002 | 1.130581 |
| Br | -1.357495 | 1.769073 | -0.690007 |
| C | 1.970062 | 2.181186 | 0.712353 |
| H | -0.004495 | 0.899215 | 2.021820 |
| Br | 3.111938 | -0.359554 | 0.276801 |
| H | 1.201370 | 2.892230 | 1.003607 |
| H | 0.642453 | -0.713680 | 1.749840 |
| H | 2.914579 | 2.574122 | 0.352450 |

| TS-rot-Br, MP2 | x | y | z |
| --- | --- | --- | --- |
| B | 0.689673 | 0.000746 | 0.178707 |
| C | -1.763823 | 0.006292 | 1.447738 |
| C | -0.249690 | 0.004953 | 1.482239 |
| Br | 0.460271 | 1.670076 | -0.969138 |
| Br | 2.630817 | 0.005492 | 0.886948 |
| Br | 0.463032 | -1.679382 | -0.953975 |
| C | -2.519842 | 0.012029 | 2.559688 |
| H | 0.048190 | -0.871391 | 2.074882 |
| Br | -2.717297 | -0.001428 | -0.222333 |
| H | -2.031007 | 0.016218 | 3.531457 |
| H | 0.050023 | 0.882732 | 2.071756 |
| H | -3.604255 | 0.012540 | 2.537826 |

| TS-rot-Br, B3LYP | x | y | z |
| --- | --- | --- | --- |
| B | 0.683701 | 0.005714 | 0.186416 |
| C | -1.772328 | 0.046390 | 1.467228 |
| C | -0.256046 | 0.036337 | 1.488920 |
| Br | 0.457078 | 1.655495 | -1.021992 |
| Br | 2.648277 | 0.029873 | 0.894334 |
| Br | 0.463081 | -1.713998 | -0.920897 |
| C | -2.526352 | 0.090239 | 2.568610 |
| H | 0.038162 | -0.831129 | 2.095393 |
| Br | -2.726274 | -0.010480 | -0.218121 |
| H | -2.042367 | 0.121448 | 3.543261 |
| H | 0.048974 | 0.917792 | 2.069118 |
| H | -3.610610 | 0.094338 | 2.545272 |

| IM5b-Br, MP2 | x | y | z |
| --- | --- | --- | --- |
| B | 0.738226 | 0.019592 | -0.145561 |
| C | -1.697981 | -0.825423 | -0.749253 |
| C | -0.443675 | -0.160217 | -1.232155 |
| Br | 1.489800 | -1.786626 | 0.447984 |
| Br | 2.248724 | 1.071713 | -1.056317 |
| Br | 0.133887 | 1.043387 | 1.506925 |
| C | -1.909132 | -2.145792 | -0.622807 |
| H | -0.684912 | 0.824476 | -1.647900 |
| Br | -3.149862 | 0.357878 | -0.292879 |
| H | -1.110463 | -2.832779 | -0.885950 |
| H | -0.038710 | -0.762401 | -2.057401 |
| H | -2.841564 | -2.560970 | -0.255568 |

| IM5b-Br, B3LYP | x | y | z |
|---|---|---|---|
| B | 0.728494 | 0.016490 | -0.149335 |
| C | -1.706959 | -0.846272 | -0.764235 |
| C | -0.449834 | -0.175595 | -1.238383 |
| Br | 1.491930 | -1.799735 | 0.468260 |
| Br | 2.260492 | 1.069545 | -1.073251 |
| Br | 0.133614 | 1.066634 | 1.511837 |
| C | -1.925163 | -2.155490 | -0.637309 |
| H | -0.688504 | 0.804020 | -1.664204 |
| Br | -3.155733 | 0.359971 | -0.293355 |
| H | -1.128752 | -2.847997 | -0.896118 |
| H | -0.035559 | -0.780722 | -2.055422 |
| H | -2.858517 | -2.568151 | -0.270227 |
| IM5-I, B3LYP/ECP46MWB | x | y | z |
| B | 0.702056 | -0.148025 | -0.326989 |
| C | 0.462796 | -0.448756 | 2.317966 |
| C | 1.295158 | 0.031205 | 1.159537 |
| I | -1.315099 | 0.896498 | -0.648303 |
| I | 2.265424 | 0.807086 | -1.770162 |
| I | 0.479240 | -2.373737 | -0.909581 |
| C | 0.349957 | -1.698229 | 2.765569 |
| H | 2.245589 | -0.519215 | 1.191241 |
| I | -0.612462 | 1.104907 | 3.409478 |
| H | 0.893796 | -2.489371 | 2.253679 |
| H | 1.550607 | 1.086546 | 1.293975 |
| H | -0.267551 | -1.979276 | 3.611124 |
| IM5-I, B3LYP/SVP | x | y | z |
| B | 0.682123 | 0.011521 | 0.194134 |
| C | -1.679427 | -0.929676 | 1.006359 |
| C | -0.460599 | -0.105357 | 1.328450 |
| I | -0.093612 | 0.819992 | -1.802502 |
| I | 2.280853 | 1.476311 | 1.017036 |
| I | 1.724614 | -2.009917 | -0.194717 |
| C | -1.776786 | -2.259127 | 1.054161 |
| H | 0.012525 | -0.545302 | 2.216878 |
| I | -3.451050 | 0.185058 | 0.465437 |
| H | -0.904052 | -2.839989 | 1.343226 |
| H | -0.759332 | 0.908488 | 1.611217 |
| H | -2.681570 | -2.802389 | 0.805719 |
| TS-rot-I, B3LYP/ECP46MWB | x | y | z |
| B | -0.002105 | -0.000052 | 0.002036 |
| C | 0.004205 | 0.000001 | 2.793336 |
| C | 0.696554 | -0.000153 | 1.441951 |
| I | -1.235626 | 1.893887 | -0.416050 |
| I | 1.780533 | -0.000376 | -1.532520 |
| I | -1.236295 | -1.893569 | -0.416006 |
| C | 0.678986 | -0.000095 | 3.945062 |
| H | 1.360453 | -0.875537 | 1.449401 |
| I | -2.164836 | 0.000400 | 2.991033 |
| H | 1.768700 | -0.000292 | 3.919311 |
| H | 1.360768 | 0.874992 | 1.449367 |
| H | 0.204865 | 0.000014 | 4.920051 |

| TS-rot-I, B3LYP/SVP | x | y | z |
|---|---|---|---|
| B | 0.652080 | -0.000004 | 0.256656 |
| C | -1.653190 | -0.000067 | 1.827080 |
| C | -0.143817 | -0.000050 | 1.649505 |
| I | 0.293677 | 1.892507 | -0.990067 |
| I | 2.912075 | -0.000164 | 0.846780 |
| I | 0.293488 | -1.892308 | -0.990294 |
| C | -2.227372 | -0.000133 | 3.033807 |
| H | 0.223309 | -0.874580 | 2.203821 |
| I | -3.021184 | 0.000000 | 0.155032 |
| H | -1.596843 | -0.000177 | 3.922916 |
| H | 0.223253 | 0.874513 | 2.203809 |
| H | -3.300833 | -0.000129 | 3.186904 |
| IM5b-I, B3LYP/ECP46MWB | x | y | z |
| B | 0.716230 | 0.125760 | -0.318366 |
| C | 0.494137 | 0.407471 | 2.330781 |
| C | 1.311196 | -0.078543 | 1.164318 |
| I | 0.522988 | 2.360246 | -0.876535 |
| I | 2.263762 | -0.835702 | -1.774842 |
| I | -1.316433 | -0.886272 | -0.647599 |
| C | 0.412806 | 1.654305 | 2.792459 |
| H | 1.546839 | -1.139824 | 1.288251 |
| I | -0.616835 | -1.131008 | 3.407666 |
| H | 0.974687 | 2.437613 | 2.288065 |
| H | 2.272166 | 0.453248 | 1.196715 |
| H | -0.195952 | 1.941024 | 3.642434 |
| IM5b-I, B3LYP/SVP | x | y | z |
| B | -0.681617 | 0.006290 | 0.195154 |
| C | 1.675536 | -0.943280 | 1.018298 |
| C | 0.450013 | -0.129156 | 1.338519 |
| I | -1.661156 | -2.021783 | -0.295672 |
| I | -2.329290 | 1.388784 | 1.061809 |
| I | 0.093157 | 0.933928 | -1.750502 |
| C | 1.783853 | -2.271358 | 1.076169 |
| H | 0.739917 | 0.880362 | 1.646260 |
| I | 3.437564 | 0.178466 | 0.462976 |
| H | 0.917053 | -2.857772 | 1.372515 |
| H | -0.032489 | -0.590154 | 2.211141 |
| H | 2.692622 | -2.809083 | 0.830016 |

**Table S26.** Cartesian coordinates (in Å) for structures referring to Figure S14

| $C_2H_2$+Cl·, MP2/6-31+G* | x | y | z |
|---|---|---|---|
| C | 1.529624 | -0.612698 | 0.000008 |
| C | 1.529792 | 0.612588 | -0.000001 |
| H | 1.553534 | 1.682295 | -0.000008 |
| Cl | -1.262507 | 0.000046 | -0.000003 |
| H | 1.552592 | -1.682421 | 0.000015 |
| $C_2H_2$+Cl·[a], MP2/tzvpp | x | y | z |
| C | -1.394097 | 0.610444 | 0.000008 |
| C | -1.394675 | -0.610330 | -0.000001 |
| H | -1.430884 | -1.674469 | -0.000008 |
| Cl | 1.152618 | -0.000045 | -0.000003 |
| H | -1.430995 | 1.674545 | 0.000016 |

| TS0-Cl, MP2/6-31+G* | x | y | z |
|---|---|---|---|
| C | -1.000913 | 0.616374 | 0.000005 |
| C | -1.668786 | -0.395859 | 0.000001 |
| H | -2.138708 | -1.357074 | -0.000003 |
| Cl | 1.115293 | -0.096193 | -0.000002 |
| H | -0.803082 | 1.669269 | 0.000009 |
| **TS0-Cl, MP2/tzvpp** | **x** | **y** | **z** |
| C | -1.000419 | 0.625854 | 0.000005 |
| C | -1.615468 | -0.411320 | 0.000001 |
| H | -2.058664 | -1.379218 | -0.000003 |
| Cl | 1.091580 | -0.093015 | -0.000002 |
| H | -0.802884 | 1.673268 | 0.000009 |
| **$C_2H_2Cl\cdot$, MP2/6-31+G*** | **x** | **y** | **z** |
| C | -0.658336 | 0.512851 | 0.000005 |
| C | -1.693308 | -0.243765 | 0.000000 |
| H | -1.983859 | -1.282385 | -0.000008 |
| Cl | 0.986908 | -0.113449 | -0.000002 |
| H | -0.683708 | 1.596496 | 0.000013 |
| **$C_2H_2Cl\cdot$, MP2/tzvpp** | **x** | **y** | **z** |
| C | -0.656463 | 0.511162 | 0.000005 |
| C | -1.680982 | -0.244442 | 0.000000 |
| H | -1.976562 | -1.275799 | -0.000009 |
| Cl | 0.981754 | -0.112595 | -0.000002 |
| H | -0.688586 | 1.589592 | 0.000014 |
| **$C_2H_2$+Br·, MP2/6-31+G*** | **x** | **y** | **z** |
| C | -2.090161 | -0.613002 | 0.000010 |
| C | -2.090029 | 0.613043 | -0.000001 |
| Br | 0.837321 | -0.000008 | -0.000002 |
| H | -2.112442 | 1.682700 | -0.000010 |
| H | -2.112659 | -1.682658 | 0.000019 |
| **$C_2H_2$+Br·, MP2/tzvpp[a]** | **x** | **y** | **z** |
| C | -1.991617 | -0.609020 | 0.000010 |
| C | -1.991440 | 0.609083 | -0.000001 |
| Br | 0.798196 | -0.000013 | -0.000002 |
| H | -2.019075 | 1.673322 | -0.000011 |
| H | -2.019443 | -1.673249 | 0.000020 |
| **$C_2H_2$+Br·, B3LYP** | **x** | **y** | **z** |
| C | 1.978726 | 0.609819 | 0.000009 |
| C | 1.985532 | -0.607311 | 0.000000 |
| Br | -0.794593 | -0.000508 | -0.000002 |
| H | 2.019934 | -1.676159 | -0.000009 |
| H | 2.005276 | 1.678882 | 0.000018 |
| **TS0-Br, MP2/6-31+G*** | **x** | **y** | **z** |
| C | 1.445941 | 0.599939 | 0.000005 |
| C | 2.164083 | -0.386907 | -0.000001 |
| Br | -0.731723 | -0.045517 | -0.000001 |
| H | 2.627538 | -1.351242 | -0.000008 |
| H | 1.322614 | 1.666145 | 0.000013 |
| **TS0-Br, MP2/tzvpp** | **x** | **y** | **z** |
| C | 1.442419 | 0.604365 | 0.000005 |
| C | 2.127530 | -0.394333 | -0.000002 |
| Br | -0.723403 | -0.044908 | -0.000001 |
| H | 2.593669 | -1.351504 | -0.000009 |
| H | 1.305754 | 1.663086 | 0.000012 |

| TS0-Br, B3LYP | x | y | z |
|---|---|---|---|
| C | 1.636813 | 0.633705 | 0.000007 |
| C | 2.210681 | -0.452107 | 0.000000 |
| Br | -0.775346 | -0.037810 | -0.000001 |
| H | 2.573972 | -1.458656 | -0.000008 |
| H | 1.478182 | 1.692433 | 0.000015 |

| $C_2H_2Br\cdot$, MP2/6-31+G* | x | y | z |
|---|---|---|---|
| C | 1.166158 | 0.536216 | 0.000007 |
| C | 2.140471 | -0.291347 | 0.000001 |
| Br | -0.670146 | -0.049763 | -0.000002 |
| H | 2.371472 | -1.344655 | -0.000008 |
| H | 1.243854 | 1.617131 | 0.000018 |

| $C_2H_2Br\cdot$, MP2/tzvpp | x | y | z |
|---|---|---|---|
| C | -1.156319 | 0.532088 | -0.000003 |
| C | -2.123166 | -0.288819 | 0.000004 |
| Br | 0.665214 | -0.049530 | 0.000000 |
| H | -2.367035 | -1.333440 | 0.000013 |
| H | -1.238557 | 1.607366 | -0.000012 |

| $C_2H_2Br\cdot$, B3LYP | x | y | z |
|---|---|---|---|
| C | 1.192419 | 0.547304 | 0.000006 |
| C | 2.174911 | -0.297913 | -0.000001 |
| Br | -0.682493 | -0.050908 | -0.000001 |
| H | 2.436880 | -1.343905 | -0.000010 |
| H | 1.246386 | 1.629341 | 0.000016 |

| $C_2H_2$+I·, MP2/6-31+G* | x | y | z |
|---|---|---|---|
| C | -0.612286 | -2.602092 | 0.000000 |
| C | 0.612287 | -2.602162 | 0.000000 |
| I | 0.000000 | 0.687926 | 0.000000 |
| H | 1.681963 | -2.617334 | 0.000000 |
| H | -1.681964 | -2.617226 | 0.000000 |

| $C_2H_2$+I·, MP2/tzvpp[a] | x | y | z |
|---|---|---|---|
| C | -0.608045 | -2.452931 | 0.000000 |
| C | 0.608049 | -2.453889 | 0.000000 |
| I | 0.000000 | 0.648814 | 0.000000 |
| H | 1.672286 | -2.474287 | 0.000000 |
| H | -1.672309 | -2.471964 | 0.000000 |

| $C_2H_2$+I·, B3LYP | x | y | z |
|---|---|---|---|
| C | 0.607412 | -2.419548 | 0.000000 |
| C | -0.607417 | -2.421067 | 0.000000 |
| I | 0.000000 | 0.640149 | 0.000000 |
| H | -1.676512 | -2.443766 | 0.000000 |
| H | 1.676543 | -2.440440 | 0.000000 |

| TS0-I, MP2/6-31+G* | x | y | z |
|---|---|---|---|
| C | 0.485447 | -1.731714 | 0.000000 |
| C | -0.495081 | -2.483675 | 0.000000 |
| I | 0.000000 | 0.564310 | 0.000000 |
| H | -1.504124 | -2.842898 | 0.000000 |
| H | 1.561930 | -1.773213 | 0.000000 |

| TS0-I, MP2/tzvpp | x | y | z |
|---|---|---|---|
| C | 0.489985 | -1.717675 | 0.000000 |
| C | -0.502072 | -2.430093 | 0.000000 |
| I | 0.000000 | 0.555643 | 0.000000 |
| H | -1.488367 | -2.832195 | 0.000000 |
| H | 1.560890 | -1.730275 | 0.000000 |

| TS0-I, B3LYP | x | y | z |
|---|---|---|---|
| C | 0.504025 | -1.819638 | 0.000000 |
| C | -0.511793 | -2.548330 | 0.000000 |
| I | 0.000000 | 0.583215 | 0.000000 |
| H | -1.532845 | -2.872924 | 0.000000 |
| H | 1.579457 | -1.829670 | 0.000000 |
| $C_2H_2I\cdot$, MP2/6-31+G* | x | y | z |
| C | 0.461451 | -1.573136 | 0.000000 |
| C | -0.461586 | -2.449519 | 0.000000 |
| I | 0.000000 | 0.537220 | 0.000000 |
| H | -1.530501 | -2.590550 | 0.000000 |
| H | 1.531312 | -1.746161 | 0.000000 |
| $C_2H_2I\cdot$, MP2/tzvpp | x | y | z |
| C | 0.457251 | -1.531168 | 0.000000 |
| C | -0.457354 | -2.403921 | 0.000000 |
| I | 0.000000 | 0.525884 | 0.000000 |
| H | -1.520322 | -2.549939 | 0.000000 |
| H | 1.520943 | -1.711401 | 0.000000 |
| $C_2H_2I\cdot$, B3LYP | x | y | z |
| C | 0.473172 | -1.635658 | 0.000000 |
| C | -0.475450 | -2.500582 | 0.000000 |
| I | 0.000000 | 0.552414 | 0.000000 |
| H | -1.534073 | -2.697224 | 0.000000 |
| H | 1.547741 | -1.763300 | 0.000000 |

**Table S27.** Cartesian coordinates (in Å) for structures referring to Figure S15

| $C_2H_2Cl\cdot$+$BCl_3$ | x | y | z |
|---|---|---|---|
| Br | -1.649749 | 0.083522 | -0.051364 |
| C | 2.243672 | 0.201633 | 0.059451 |
| C | 1.417373 | -0.777166 | 0.086656 |
| Cl | -1.725555 | -0.107366 | 1.675155 |
| Cl | -1.248871 | 1.630592 | -0.738623 |
| Cl | -2.021275 | -1.259445 | -1.092295 |
| H | 1.965227 | 1.244989 | -0.037738 |
| Cl | 3.985120 | -0.014589 | 0.174373 |
| H | 1.374409 | -1.852480 | 0.168564 |
| TS1-Cl | x | y | z |
| Br | 1.191974 | -0.043753 | -0.000059 |
| C | -1.826089 | -0.285751 | 0.000062 |
| C | -0.900373 | 0.599784 | 0.000475 |
| Cl | 1.616137 | 0.753380 | 1.517766 |
| Cl | 1.031372 | -1.799529 | -0.000708 |
| Cl | 1.615766 | 0.754579 | -1.517347 |
| H | -1.671654 | -1.357297 | -0.000336 |
| Cl | -3.505642 | 0.174759 | 0.000082 |
| H | -0.809212 | 1.677649 | 0.000941 |

| IM-Cl | x | y | z |
|---|---|---|---|
| B | -0.912819 | 0.046150 | -0.123482 |
| C | 1.668256 | 0.420978 | -0.072263 |
| C | 0.577717 | -0.378345 | -0.382397 |
| Cl | -1.252440 | 1.772740 | 0.261469 |
| Cl | -2.153508 | -0.763064 | -1.144648 |
| Cl | -0.505932 | -0.994373 | 1.502511 |
| H | 1.566355 | 1.390016 | 0.404054 |
| H | 0.772391 | -1.344764 | -0.846213 |
| Cl | 3.250087 | -0.046586 | -0.396537 |

| $C_2H_2Br\cdot{}+BBr_3$, MP2/SVP | x | y | z |
|---|---|---|---|
| Br | -4.211062 | 0.009744 | -0.160998 |
| C | -2.309103 | -0.011548 | 0.142916 |
| C | -1.472640 | 0.051214 | -0.820438 |
| H | -1.401898 | 0.121804 | -1.894898 |
| H | -2.068329 | -0.081012 | 1.197486 |
| B | 1.555684 | -0.005254 | 0.087778 |
| Br | 1.815070 | 1.692006 | -0.726038 |
| Br | 1.815059 | -1.594128 | -0.921384 |
| Br | 1.106142 | -0.114836 | 1.931953 |

| $C_2H_2Br\cdot{}+BBr_3$, MP2/tzvpp | x | y | z |
|---|---|---|---|
| Br | 4.090361 | -0.022316 | -0.173854 |
| C | 2.210798 | 0.027879 | 0.156522 |
| C | 1.363705 | -0.104498 | -0.777415 |
| H | 1.260855 | -0.255608 | -1.835290 |
| H | 1.981598 | 0.177319 | 1.200148 |
| B | -1.492606 | 0.010802 | 0.085855 |
| Br | -1.750162 | -1.752601 | -0.555337 |
| Br | -1.776238 | 1.488064 | -1.064932 |
| Br | -1.056145 | 0.300681 | 1.906443 |

| TS1-Br, MP2/SVP | x | y | z |
|---|---|---|---|
| Br | -3.845576 | 0.173016 | -0.069194 |
| C | -1.987073 | -0.250696 | 0.099693 |
| C | -1.094991 | 0.598028 | -0.239378 |
| H | -1.002804 | 1.597911 | -0.638619 |
| H | -1.816329 | -1.244199 | 0.496293 |
| B | 1.243762 | -0.040917 | 0.016521 |
| Br | 1.621454 | 1.400543 | 1.223857 |
| Br | 1.622165 | 0.172266 | -1.851491 |
| Br | 1.033177 | -1.809629 | 0.722481 |

| TS1-Br, MP2/tzvpp | x | y | z |
|---|---|---|---|
| Br | -3.874691 | -0.003235 | 0.193137 |
| C | -2.021122 | 0.004221 | -0.231508 |
| C | -1.138493 | -0.011989 | 0.679076 |
| H | -1.019767 | -0.031053 | 1.746687 |
| H | -1.831904 | 0.023082 | -1.293181 |
| B | 1.310421 | 0.001031 | -0.061537 |
| Br | 1.649991 | -1.662911 | 0.798853 |
| Br | 1.652768 | 1.627862 | 0.866164 |
| Br | 1.007853 | 0.039697 | -1.939047 |

| IM-Br, MP2/SVP | x | y | z |
|---|---|---|---|
| B | -0.005744 | -0.002469 | -0.005360 |
| C | -0.002818 | -0.003447 | 2.593935 |
| C | 0.693349 | -0.013487 | 1.401295 |
| Br | -1.923630 | 0.408853 | -0.100921 |
| Br | 1.075590 | 0.692157 | -1.486893 |
| Br | 0.319364 | -2.133857 | 0.301239 |
| H | -1.086839 | -0.028450 | 2.640521 |
| H | 1.782737 | 0.010429 | 1.440830 |
| Br | 0.839057 | 0.041380 | 4.227503 |

| IM-Br, MP2/tzvpp | x | y | z |
|---|---|---|---|
| Br | 3.429945 | 0.016358 | -0.512733 |
| C | 1.702830 | 0.484329 | -0.148306 |
| C | 0.636154 | -0.337287 | -0.435651 |
| H | 0.838357 | -1.283369 | -0.924626 |
| H | 1.574421 | 1.444937 | 0.327411 |
| B | -0.855027 | 0.059525 | -0.168171 |
| Br | -0.311408 | -1.111587 | 1.563357 |
| Br | -2.206021 | -0.843921 | -1.244558 |
| Br | -1.260274 | 1.900823 | 0.335128 |

| $C_2H_2I\cdot+BI_3$ | x | y | z |
|---|---|---|---|
| Br | 1.567986 | -0.056498 | 0.075786 |
| C | -1.440539 | 0.452500 | -0.620175 |
| C | -2.287263 | -0.105538 | 0.147929 |
| I | 1.845828 | -0.891914 | -1.871501 |
| I | 1.810441 | 2.044231 | 0.383445 |
| I | 1.160151 | -1.337953 | 1.737301 |
| H | -1.342427 | 1.092601 | -1.483471 |
| H | -2.080014 | -0.742138 | 1.000487 |
| I | -4.406513 | 0.155338 | -0.158486 |

| TS1-I | x | y | z |
|---|---|---|---|
| Br | 1.397688 | -0.103598 | -0.005810 |
| C | -1.180075 | 0.335457 | -0.586114 |
| C | -2.061480 | -0.217637 | 0.146514 |
| I | 1.738931 | -0.968585 | -1.941404 |
| I | 1.741633 | 1.993904 | 0.299910 |
| I | 1.139502 | -1.388815 | 1.692773 |
| H | -1.077503 | 0.983446 | -1.444140 |
| H | -1.895934 | -0.863180 | 1.001224 |
| I | -4.148841 | 0.086970 | -0.256222 |

| IM-I | x | y | z |
|---|---|---|---|
| B | 0.489000 | -0.078524 | 0.104095 |
| C | -0.990698 | 0.214708 | -0.293521 |
| C | -2.049849 | -0.273381 | 0.384175 |
| I | 1.704470 | -0.695883 | -1.787127 |
| I | 1.714972 | 1.904622 | 0.082370 |
| I | 0.919860 | -1.339375 | 1.853893 |
| H | -1.169376 | 0.847256 | -1.164704 |
| H | -1.971682 | -0.909051 | 1.260065 |
| I | -4.057070 | 0.128915 | -0.161311 |

**Table S28.** Cartesian coordinates (in Å) for structures referring to Figure S16

| $IM+BCl_3$ | x | y | z |
|---|---|---|---|
| B | 1.999328 | -0.760994 | 0.029983 |
| C | 1.687529 | 1.829930 | -0.043283 |
| C | 1.299177 | 0.586990 | 0.432041 |
| Cl | 3.604877 | -0.682774 | -0.780842 |
| Cl | 1.789174 | -2.125877 | 1.182143 |
| Cl | 0.523735 | -0.683407 | -1.284796 |
| H | 2.482712 | 1.956745 | -0.770394 |
| H | 0.473349 | 0.551588 | 1.140980 |
| Cl | 0.953209 | 3.254052 | 0.459380 |
| B | -2.633862 | -0.166244 | 0.072587 |
| Cl | -3.335322 | -1.410922 | -0.912627 |
| Cl | -2.498215 | 1.458361 | -0.532972 |
| Cl | -2.078849 | -0.537298 | 1.680539 |
| TS2-Cl | x | y | z |
| B | 2.189523 | -0.653006 | 0.194525 |
| C | 1.517953 | 1.789131 | 0.035037 |
| C | 1.433518 | 0.609098 | 0.723698 |
| Cl | 3.685813 | -0.484267 | -0.723914 |
| Cl | 1.839616 | -2.206388 | 0.939589 |
| Cl | 0.431758 | -0.200238 | -1.431588 |
| H | 2.199952 | 1.927530 | -0.798609 |
| H | 0.714278 | 0.531504 | 1.538090 |
| Cl | 0.660607 | 3.170926 | 0.450857 |
| B | -2.555252 | -0.327948 | 0.108486 |
| Cl | -3.203716 | -1.302054 | -1.175644 |
| Cl | -2.652078 | 1.406826 | 0.025235 |
| Cl | -1.867555 | -1.087373 | 1.515057 |
| $E+Cl^{\cdot}+BCl_3$ | x | y | z |
| B | -2.313844 | -0.686680 | -0.251164 |
| C | -1.596656 | 1.761206 | -0.079434 |
| C | -1.432621 | 0.525476 | -0.656584 |
| Cl | -3.692845 | -0.508994 | 0.805417 |
| Cl | -1.956482 | -2.248382 | -0.935757 |
| Cl | -0.213346 | 0.286133 | 1.616603 |
| H | -2.352164 | 1.961222 | 0.674158 |
| H | -0.667530 | 0.412706 | -1.423449 |
| Cl | -0.733670 | 3.141545 | -0.568405 |
| B | 2.574246 | -0.427296 | -0.152682 |
| Cl | 3.517484 | -0.466309 | 1.306354 |
| Cl | 2.423967 | 1.054789 | -1.052570 |
| Cl | 1.825089 | -1.877849 | -0.749015 |

| IM+$BBr_3$, MP2/SVP | x | y | z |
|---|---|---|---|
| B | 2.188323 | -0.632055 | 0.039139 |
| C | 1.782494 | 1.934888 | -0.009572 |
| C | 1.412226 | 0.678067 | 0.424337 |
| Br | 3.953500 | -0.471260 | -0.804281 |
| Br | 1.982410 | -2.136980 | 1.277284 |
| Br | 0.604736 | -0.601481 | -1.467590 |
| H | 2.604733 | 2.100262 | -0.698685 |
| H | 0.567715 | 0.597043 | 1.109137 |
| Br | 0.915400 | 3.459668 | 0.535569 |
| B | -2.666994 | -0.220253 | 0.110430 |
| Br | -2.625817 | 1.592818 | -0.452947 |
| Br | -1.996956 | -0.691644 | 1.826531 |
| Br | -3.403199 | -1.554363 | -1.018763 |

| TS2-Br, MP2/SVP | x | y | z |
|---|---|---|---|
| B | 2.346314 | -0.604642 | 0.114295 |
| C | 1.855803 | 1.955986 | 0.088091 |
| C | 1.431240 | 0.639490 | 0.346960 |
| Br | 4.047707 | -0.427336 | -0.775303 |
| Br | 2.020410 | -2.185788 | 1.165378 |
| Br | 0.561409 | -0.280859 | -1.476583 |
| H | 2.744113 | 2.186154 | -0.488326 |
| H | 0.592917 | 0.523903 | 1.032742 |
| Br | 0.826733 | 3.412617 | 0.572184 |
| B | -2.676746 | -0.303720 | 0.118349 |
| Br | -2.857709 | 1.524726 | -0.356373 |
| Br | -1.907590 | -0.773057 | 1.794888 |
| Br | -3.302591 | -1.662906 | -1.047560 |

| IM2+$BBr_3$ | x | y | Z |
|---|---|---|---|
| B | 2.234625 | -0.848915 | 0.173055 |
| C | 2.352479 | 1.738527 | 0.122914 |
| C | 1.517134 | 0.554828 | 0.175241 |
| Br | 3.889567 | -1.105226 | -0.730319 |
| Br | 1.430507 | -2.281811 | 1.128149 |
| Br | 0.449985 | 0.325273 | -1.562189 |
| H | 3.284640 | 1.775789 | -0.427020 |
| H | 0.724413 | 0.629312 | 0.920327 |
| Br | 1.638092 | 3.393125 | 0.584703 |
| B | -2.689570 | -0.213433 | 0.141530 |
| Br | -2.573442 | -2.075573 | -0.198510 |
| Br | -3.556960 | 0.934850 | -1.095530 |
| Br | -1.990664 | 0.499263 | 1.763549 |

| TS3 | x | y | z |
|---|---|---|---|
| B | 2.148057 | -0.807568 | 0.122462 |
| C | 2.182485 | 1.763948 | -0.099715 |
| C | 1.467712 | 0.608452 | 0.277635 |
| Br | 3.390649 | -1.140446 | -1.273323 |
| Br | 1.745200 | -2.124920 | 1.417342 |
| Br | -0.141602 | 0.252065 | -1.232829 |
| H | 3.001208 | 1.744789 | -0.810312 |
| H | 0.796439 | 0.728826 | 1.129444 |
| Br | 1.670570 | 3.438407 | 0.468740 |
| B | -2.095781 | -0.181571 | -0.019179 |
| Br | -2.114030 | -2.126602 | 0.166943 |
| Br | -3.422250 | 0.554733 | -1.258264 |
| Br | -1.870256 | 0.810697 | 1.657017 |

| E+BBr4˙ | x | y | z |
|---|---|---|---|
| B | 2.241308 | -0.475699 | 0.032532 |
| C | 1.518488 | 1.929614 | -0.404145 |
| C | 1.379855 | 0.785323 | 0.345563 |
| Br | 2.673353 | -0.991386 | -1.742083 |
| Br | 3.006066 | -1.375059 | 1.519918 |
| Br | -0.791750 | 0.563043 | -1.411752 |
| H | 2.061676 | 1.956528 | -1.346780 |
| H | 0.843915 | 0.851886 | 1.292859 |
| Br | 0.907971 | 3.587204 | 0.158585 |
| B | -1.866425 | -0.481340 | 0.157249 |
| Br | -0.789923 | -2.072738 | 0.622671 |
| Br | -3.536685 | -0.894606 | -0.823801 |
| Br | -2.102462 | 0.774604 | 1.660932 |
| IM+BI3 | x | y | z |
| B | 0.327733 | 1.476734 | 1.336521 |
| C | -1.050084 | 1.376421 | 0.615453 |
| C | -2.080198 | 0.659202 | 1.106180 |
| I | 2.064999 | 1.205985 | -0.201791 |
| I | 0.972239 | 3.713276 | 1.470985 |
| I | 0.669317 | 0.371015 | 3.207737 |
| H | -1.187432 | 1.932823 | -0.313421 |
| H | -2.034239 | 0.078012 | 2.021312 |
| I | -3.978663 | 0.564577 | 0.175716 |
| B | 0.154825 | -1.984486 | -1.583203 |
| I | 2.171498 | -2.477587 | -2.104763 |
| I | -0.701890 | -2.784242 | 0.194783 |
| I | -0.986686 | -0.706972 | -2.851548 |
| TS2-I | x | y | z |
| B | 0.040311 | 1.685039 | 1.658706 |
| C | -1.208655 | 1.478648 | 0.784698 |
| C | -2.230429 | 0.690959 | 1.204999 |
| I | 2.091648 | 1.086831 | -0.263333 |
| I | 1.020272 | 3.658913 | 1.472380 |
| I | 0.464587 | 0.525432 | 3.424241 |
| H | -1.315633 | 2.044386 | -0.140763 |
| H | -2.200275 | 0.107476 | 2.120039 |
| I | -4.066281 | 0.515218 | 0.185452 |
| B | 0.138100 | -1.885947 | -1.557411 |
| I | 2.098676 | -2.446894 | -2.211832 |
| I | -0.596314 | -2.605246 | 0.309548 |
| I | -1.083177 | -0.656764 | -2.798633 |
| E+I˙+BI3 | x | y | z |
| B | -0.268416 | 1.837643 | 1.618067 |
| C | -1.604571 | 1.573770 | 0.916809 |
| C | -2.456627 | 0.611785 | 1.348262 |
| I | 3.321761 | 0.544174 | -0.002487 |
| I | 1.063643 | 3.274780 | 0.704934 |
| I | 0.326998 | 0.876538 | 3.438507 |
| H | -1.864955 | 2.151124 | 0.031019 |
| H | -2.268626 | -0.009610 | 2.219047 |
| I | -4.268984 | 0.156873 | 0.386968 |
| B | 0.297755 | -1.695879 | -1.611719 |
| I | 1.804354 | -2.764667 | -2.683571 |
| I | -0.360747 | -2.415231 | 0.286659 |
| I | -0.568754 | 0.056748 | -2.464411 |

**Table S29.** Cartesian coordinates (in Å) for structures referring to Figure 17

| IM+$BBr_3$, CCSD(T)/MP2 | x | y | z |
|---|---|---|---|
| C | 0.477344 | -1.115405 | -1.134778 |
| C | 1.217569 | -2.218250 | -0.759098 |
| B | 1.020308 | 0.010991 | -2.084400 |
| H | -0.553883 | -1.044519 | -0.788690 |
| Br | 0.527145 | -3.552514 | 0.298467 |
| H | 2.251180 | -2.361874 | -1.058056 |
| Br | 2.679816 | -0.310077 | -3.080706 |
| Br | -0.343523 | 1.052867 | -3.030254 |
| Br | 1.450497 | 0.849933 | -0.108983 |
| B | -1.504465 | 0.806933 | 2.073815 |
| Br | -1.129569 | 2.579853 | 2.633328 |
| Br | -2.668845 | 0.502123 | 0.601027 |
| Br | -0.747101 | -0.667706 | 3.000223 |
| TS2-Br, CCSD(T)/MP2 | x | y | z |
| C | 0.447847 | 0.057045 | 0.077237 |
| C | 1.290131 | -0.751262 | 0.787744 |
| B | 1.054713 | 1.264330 | -0.723083 |
| H | -0.625465 | -0.018973 | 0.248138 |
| Br | 0.695859 | -2.084128 | 1.913650 |
| H | 2.368707 | -0.738980 | 0.655984 |
| Br | 2.724186 | 1.025428 | -1.691735 |
| Br | -0.184246 | 2.583079 | -1.412352 |
| Br | 1.698085 | 1.879330 | 1.554758 |
| B | -1.315565 | 2.047376 | 3.521332 |
| Br | -0.920006 | 3.856367 | 3.936350 |
| Br | -2.472013 | 1.640950 | 2.067768 |
| Br | -0.616092 | 0.645565 | 4.593897 |
| E+$Br^{\cdot}$+$BBr_3$, CCSD(T)/MP2 | x | y | z |
| C | 0.391930 | -1.244862 | -1.295996 |
| C | 1.427789 | -1.967617 | -0.772324 |
| B | 0.577914 | -0.348133 | -2.545092 |
| H | -0.594438 | -1.342721 | -0.843815 |
| Br | 1.217509 | -3.207997 | 0.606557 |
| H | 2.433803 | -1.944303 | -1.181819 |
| Br | 2.239550 | -0.238953 | -3.481067 |
| Br | -0.931479 | 0.602060 | -3.212582 |
| Br | 1.687702 | 0.628902 | 0.328382 |
| B | -1.538226 | 0.950383 | 1.982313 |
| Br | -0.738995 | 2.060543 | 3.299564 |
| Br | -2.293173 | 1.716854 | 0.419268 |
| Br | -1.632195 | -0.927703 | 2.254212 |

| IM+$BBr_3$, MP2/tzvpp | x | y | z |
|---|---|---|---|
| B | 2.117311 | -0.641067 | 0.040522 |
| C | 1.773793 | 1.925980 | -0.004138 |
| C | 1.366779 | 0.681988 | 0.417193 |
| Br | 3.855187 | -0.516166 | -0.838740 |
| Br | 1.885501 | -2.151429 | 1.249318 |
| Br | 0.519316 | -0.537116 | -1.414556 |
| H | 2.605565 | 2.057927 | -0.680117 |
| H | 0.521610 | 0.620288 | 1.094437 |
| Br | 0.946535 | 3.464751 | 0.521299 |
| B | -2.581886 | -0.223461 | 0.119285 |
| Br | -2.489944 | 1.648139 | -0.151685 |
| Br | -1.917773 | -0.972681 | 1.724971 |
| Br | -3.360186 | -1.335596 | -1.196084 |
| TS2-Br, MP2/tzvpp | x | y | z |
| B | 2.273270 | -0.496640 | 0.213948 |
| C | 1.534409 | 1.895867 | 0.100456 |
| C | 1.532344 | 0.741767 | 0.821282 |
| Br | 3.950417 | -0.238261 | -0.718005 |
| Br | 1.977112 | -2.218630 | 1.025288 |
| Br | 0.560038 | -0.105739 | -1.453560 |
| H | 2.206642 | 2.046990 | -0.733284 |
| H | 0.840306 | 0.640388 | 1.649143 |
| Br | 0.547963 | 3.375055 | 0.542047 |
| B | -2.520542 | -0.402969 | 0.115108 |
| Br | -2.788633 | 1.459036 | -0.091192 |
| Br | -1.760818 | -1.075441 | 1.712323 |
| Br | -3.063538 | -1.596452 | -1.248088 |
| E+$Br^{\cdot}$+$BBr_3$, MP2/tzvpp | x | y | z |
| B | -2.517407 | -0.506793 | -0.293315 |
| C | -1.627327 | 1.868647 | -0.156176 |
| C | -1.524482 | 0.610193 | -0.681512 |
| Br | -4.008308 | -0.169287 | 0.836274 |
| Br | -2.281412 | -2.236483 | -1.032255 |
| Br | -0.262232 | 0.379276 | 1.671557 |
| H | -2.404188 | 2.135306 | 0.546967 |
| H | -0.734204 | 0.412862 | -1.396210 |
| Br | -0.574113 | 3.289315 | -0.692376 |
| B | 2.568961 | -0.532272 | -0.171484 |
| Br | 2.770323 | 1.220089 | -0.855326 |
| Br | 1.568930 | -1.810596 | -1.144714 |
| Br | 3.409425 | -1.021624 | 1.451108 |

**Table S29.** Cartesian coordinates (in Å) for structures referring to Figure S17

| $C_3H_4$+Cl· | x | y | z |
|---|---|---|---|
| C | -1.027219 | 0.415361 | 0.000012 |
| C | -0.391172 | 1.471132 | -0.000004 |
| C | -1.883522 | -0.768318 | -0.000001 |
| H | -2.933356 | -0.461857 | -0.001132 |
| H | -1.696939 | -1.376620 | 0.888677 |
| H | -1.695361 | -1.377742 | -0.887576 |
| H | 0.134225 | 2.403758 | -0.000001 |
| Cl | 1.529583 | -0.346858 | 0.000000 |

| TS0-1-Cl | x | y | z |
|---|---|---|---|
| C | -0.771262 | 0.306561 | -0.000023 |
| C | -0.725233 | 1.530288 | -0.000029 |
| C | -1.470739 | -0.988934 | -0.000018 |
| H | -1.196887 | -1.562553 | -0.887213 |
| H | -2.547442 | -0.803125 | -0.000078 |
| H | -1.196977 | -1.562495 | 0.887243 |
| H | -0.512432 | 2.578903 | -0.000034 |
| Cl | 1.368067 | -0.219895 | 0.000030 |

| $C_3H_4Cl$· (1) | x | y | z |
|---|---|---|---|
| C | 0.473063 | 0.244774 | 0.000010 |
| C | 0.785988 | 1.490243 | 0.000027 |
| C | 1.399508 | -0.931455 | 0.000014 |
| H | 1.224863 | -1.548979 | 0.885558 |
| H | 2.434457 | -0.583661 | 0.000035 |
| H | 1.224893 | -1.548961 | -0.885549 |
| H | 0.332493 | 2.468888 | 0.000029 |
| Cl | -1.245180 | -0.212274 | -0.000022 |

| TS0-2-Cl | x | y | z |
|---|---|---|---|
| C | -1.108529 | 0.432376 | 0.000051 |
| C | -0.105703 | 1.127054 | -0.000002 |
| C | -2.192249 | -0.530686 | 0.000013 |
| H | -2.811518 | -0.412718 | -0.891908 |
| H | -2.808625 | -0.416328 | 0.894392 |
| H | -1.765621 | -1.540197 | -0.002796 |
| H | 0.467977 | 2.031680 | -0.000064 |
| Cl | 1.609216 | -0.343230 | 0.000000 |

| $C_3H_4Cl$· (2) | x | y | z |
|---|---|---|---|
| C | 1.087732 | 0.619428 | 0.000015 |
| C | -0.164653 | 0.901876 | -0.000004 |
| C | 2.060867 | -0.476925 | -0.000003 |
| H | 2.699011 | -0.425824 | 0.886063 |
| H | 2.698883 | -0.425882 | -0.886166 |
| H | 1.538034 | -1.442764 | 0.000064 |
| H | -0.586104 | 1.899748 | -0.000021 |
| Cl | -1.426676 | -0.345385 | 0.000001 |

| $C_3H_4$+Br· | x | y | z |
|---|---|---|---|
| C | -0.199999 | -1.734308 | -0.634216 |
| C | 1.022105 | -1.588805 | -0.675591 |
| C | -1.636497 | -2.010642 | -0.624515 |
| H | -2.036465 | -1.929248 | 0.389744 |
| H | -1.822119 | -3.023614 | -0.993131 |
| H | -2.168194 | -1.302690 | -1.265951 |
| H | 2.085785 | -1.479471 | -0.718588 |
| Br | 0.181245 | 0.917150 | 0.327125 |

| TS0-1-Br | x | y | z |
|---|---|---|---|
| C | 0.087889 | 0.370648 | -0.020883 |
| C | 1.240951 | 0.006631 | -0.250694 |
| C | -1.355645 | 0.045418 | -0.026635 |
| H | -1.760670 | 0.122190 | 0.984217 |
| H | -1.482709 | -0.974213 | -0.398547 |
| H | -1.893840 | 0.742733 | -0.671539 |
| H | 2.299401 | -0.092930 | -0.373413 |
| Br | 0.248141 | 2.477802 | 0.757484 |

| $C_3H_4Br\cdot$ (1) | | x | y | z |
|---|---|---|---|---|
| | C | -0.029298 | -1.086557 | -0.405157 |
| | C | 1.058182 | -1.682598 | -0.719218 |
| | C | -1.440463 | -1.582477 | -0.477659 |
| | H | -1.898271 | -1.565023 | 0.515360 |
| | H | -1.447855 | -2.604891 | -0.862831 |
| | H | -2.032419 | -0.941570 | -1.137063 |
| | H | 2.127555 | -1.550077 | -0.756242 |
| | Br | 0.105698 | 0.739130 | 0.273804 |

| TS0-2-Br | | x | y | z |
|---|---|---|---|---|
| | C | -0.048519 | 0.048545 | 0.041162 |
| | C | 1.114330 | 0.440018 | 0.098747 |
| | C | -1.483570 | -0.145417 | -0.050549 |
| | H | -1.862931 | -0.643772 | 0.844839 |
| | H | -1.736821 | -0.738673 | -0.932435 |
| | H | -1.970419 | 0.833832 | -0.136958 |
| | H | 2.157484 | 0.204991 | 0.192690 |
| | Br | 1.213966 | 2.698755 | -0.057508 |

| $C_3H_4Br\cdot$ (2) | | x | y | z |
|---|---|---|---|---|
| | C | -0.656709 | -1.824073 | 0.080493 |
| | C | 0.426791 | -1.144310 | 0.112454 |
| | C | -2.115986 | -1.759228 | -0.029073 |
| | H | -2.588663 | -2.188404 | 0.858532 |
| | H | -2.461799 | -2.313417 | -0.905836 |
| | H | -2.434415 | -0.712412 | -0.126192 |
| | H | 1.436622 | -1.522641 | 0.210796 |
| | Br | 0.411482 | 0.796594 | -0.020455 |

| $C_3H_4$+$I\cdot$ | | x | y | z |
|---|---|---|---|---|
| | C | -2.195771 | 0.348718 | 0.000000 |
| | C | -1.786514 | 1.507347 | -0.000001 |
| | C | -2.773399 | -0.996829 | 0.000001 |
| | H | -2.457010 | -1.552702 | -0.886957 |
| | H | -3.865503 | -0.935438 | 0.000001 |
| | H | -2.457009 | -1.552701 | 0.886960 |
| | H | -1.432219 | 2.516987 | -0.000002 |
| | I | 0.957469 | -0.068520 | 0.000000 |

| TS0-1-I | | x | y | z |
|---|---|---|---|---|
| | C | -1.521304 | 0.240255 | -0.000002 |
| | C | -1.836163 | 1.445497 | 0.000000 |
| | C | -2.176849 | -1.099472 | 0.000000 |
| | H | -1.878728 | -1.664790 | -0.885808 |
| | H | -3.261017 | -0.961283 | -0.000005 |
| | H | -1.878737 | -1.664782 | 0.885816 |
| | H | -1.697793 | 2.508856 | 0.000001 |
| | I | 0.790984 | -0.032749 | 0.000000 |

| $C_3H_4I\cdot$ (1) | | x | y | z |
|---|---|---|---|---|
| | C | -1.402860 | 0.231064 | -0.000018 |
| | C | -1.822005 | 1.432394 | -0.000028 |
| | C | -2.158083 | -1.064195 | -0.000020 |
| | H | -1.902314 | -1.652830 | -0.885277 |
| | H | -3.231175 | -0.855853 | -0.000032 |
| | H | -1.902332 | -1.652823 | 0.885247 |
| | H | -1.524738 | 2.468130 | -0.000030 |
| | I | 0.770910 | -0.035891 | 0.000009 |

| TS0-2-I | | x | y | z |
|---|---|---|---|---|
| | C | -2.122555 | 0.504088 | 0.000012 |
| | C | -1.052575 | 1.144200 | 0.000002 |
| | C | -3.016642 | -0.642803 | -0.000006 |
| | H | -3.651250 | -0.638033 | -0.889761 |
| | H | -3.651548 | -0.637812 | 0.889535 |
| | H | -2.419549 | -1.565223 | 0.000219 |
| | H | -0.740680 | 2.176297 | -0.000033 |
| | I | 0.898371 | -0.101286 | 0.000000 |
| $C_3H_4I\cdot$ (2) | | x | y | z |
| | C | 2.101048 | 0.572802 | 0.000007 |
| | C | 0.953818 | 1.120762 | -0.000002 |
| | C | 2.886450 | -0.660192 | -0.000002 |
| | H | 3.523287 | -0.711175 | 0.887182 |
| | H | 3.523120 | -0.711266 | -0.887300 |
| | H | 2.211155 | -1.527409 | 0.000107 |
| | H | 0.699960 | 2.172937 | -0.000010 |
| | I | -0.860480 | -0.102327 | 0.000000 |

**Table S29.** Cartesian coordinates (in Å) for structures referring to Figure S18

| $C_3H_4Cl\cdot+BCl_3$, MP2 | | x | y | z |
|---|---|---|---|---|
| | B | 1.714185 | -0.206072 | 0.035192 |
| | C | -1.276208 | 0.583221 | -0.079709 |
| | C | -2.005493 | -0.472516 | -0.066100 |
| | Cl | 1.432779 | -1.828677 | -0.526298 |
| | Cl | 2.140224 | 1.042006 | -1.100404 |
| | Cl | 1.647268 | 0.151729 | 1.736288 |
| | C | -1.308216 | 2.048378 | -0.076969 |
| | Cl | -3.774652 | -0.407407 | -0.023265 |
| | H | -1.639661 | -1.492121 | -0.080103 |
| | H | -0.884801 | 2.446046 | -1.003070 |
| | H | -0.735407 | 2.447179 | 0.764980 |
| | H | -2.347077 | 2.394686 | 0.011445 |
| $C_3H_4Cl\cdot+BCl_3$, B3LYP | | x | y | z |
| | B | -1.657210 | 0.216241 | 0.022352 |
| | C | 1.216752 | -0.606779 | -0.079970 |
| | C | 1.953716 | 0.466578 | -0.078387 |
| | Cl | -2.030288 | -0.859920 | -1.313702 |
| | Cl | -1.746500 | -0.372965 | 1.673688 |
| | Cl | -1.331647 | 1.913610 | -0.286020 |
| | C | 1.182361 | -2.067128 | -0.041499 |
| | Cl | 3.756530 | 0.380478 | 0.002475 |
| | H | 1.609563 | 1.492204 | -0.121219 |
| | H | 0.599969 | -2.428049 | 0.813946 |
| | H | 2.205792 | -2.464110 | 0.049361 |
| | H | 0.736148 | -2.477718 | -0.954180 |

| TS1-Cl, MP2 | x | y | z |
|---|---|---|---|
| B | -1.215477 | -0.166083 | -0.033480 |
| C | 0.852711 | 0.501217 | 0.149953 |
| C | 1.669231 | -0.490382 | 0.203945 |
| Cl | -1.147842 | -1.900597 | 0.315132 |
| Cl | -1.853950 | 0.872832 | 1.255262 |
| Cl | -1.489220 | 0.320443 | -1.713980 |
| C | 0.914059 | 1.971110 | 0.078509 |
| Cl | 3.384584 | -0.267035 | -0.031814 |
| H | 1.402292 | -1.524351 | 0.369915 |
| H | 0.661972 | 2.416974 | 1.043116 |
| H | 0.254617 | 2.376681 | -0.690404 |
| H | 1.951752 | 2.233522 | -0.167848 |

| TS1-Cl, B3LYP | x | y | z |
|---|---|---|---|
| B | -1.263383 | -0.170813 | -0.027914 |
| C | 0.884849 | 0.511472 | 0.126668 |
| C | 1.712879 | -0.507760 | 0.172726 |
| Cl | -1.874083 | 0.847244 | 1.307128 |
| Cl | -1.549977 | 0.366784 | -1.704619 |
| Cl | -1.162576 | -1.925322 | 0.274034 |
| C | 0.932309 | 1.979983 | 0.067463 |
| Cl | 3.456604 | -0.264186 | -0.024664 |
| H | 1.449022 | -1.547588 | 0.299186 |
| H | 0.279329 | 2.382698 | -0.711313 |
| H | 1.967679 | 2.274504 | -0.158209 |
| H | 0.651185 | 2.425443 | 1.026820 |

| IM-Cl, MP2 | x | y | z |
|---|---|---|---|
| B | 0.930309 | -0.084168 | -0.033683 |
| C | -0.589132 | 0.440176 | -0.109035 |
| C | -1.617338 | -0.516870 | -0.013660 |
| Cl | 1.131487 | -1.805678 | -0.648994 |
| Cl | 2.100922 | 1.047405 | -0.870450 |
| Cl | 1.081031 | -0.002145 | 1.834266 |
| C | -0.904539 | 1.879019 | -0.221013 |
| Cl | -3.235286 | -0.155759 | -0.139968 |
| H | -1.399008 | -1.567922 | 0.141474 |
| H | -0.956265 | 2.109076 | -1.298884 |
| H | -0.097205 | 2.487419 | 0.188987 |
| H | -1.861633 | 2.153326 | 0.226553 |

| IM-Cl, B3LYP | x | y | z |
|---|---|---|---|
| B | -0.931492 | 0.079931 | -0.061111 |
| C | 0.585198 | -0.456711 | -0.141007 |
| C | 1.602162 | 0.498916 | -0.024259 |
| Cl | -2.181431 | -1.094839 | -0.733845 |
| Cl | -0.951462 | 0.104886 | 1.864476 |
| Cl | -1.176624 | 1.788393 | -0.737529 |
| C | 0.881114 | -1.902703 | -0.275927 |
| Cl | 3.254445 | 0.145895 | -0.166097 |
| H | 1.388246 | 1.547463 | 0.147106 |
| H | 0.118505 | -2.503376 | 0.226108 |
| H | 1.878096 | -2.183055 | 0.067498 |
| H | 0.797999 | -2.151397 | -1.347103 |

| $C_3H_4Br^{\cdot+}BBr_3$, MP2 | x | y | z |
|---|---|---|---|
| B | -1.596538 | 0.160337 | 0.035287 |
| C | 1.407236 | -0.754677 | -0.151085 |
| C | 2.160842 | 0.278539 | -0.125651 |
| C | 1.378506 | -2.218931 | -0.140567 |
| H | 1.851428 | 1.315983 | -0.151227 |
| H | 0.784456 | -2.587582 | 0.700374 |
| H | 2.403017 | -2.604371 | -0.044840 |
| H | 0.942923 | -2.603149 | -1.066973 |
| Br | -1.216114 | 1.998905 | -0.264111 |
| Br | 4.093119 | 0.118128 | -0.028902 |
| Br | -2.029680 | -0.968205 | -1.432202 |
| Br | -1.638143 | -0.524603 | 1.807788 |

| $C_3H_4Br^{\cdot+}BBr_3$, B3LYP | x | y | z |
|---|---|---|---|
| B | -1.507147 | 0.134874 | 0.024630 |
| C | 1.351446 | -0.864311 | -0.152796 |
| C | 2.113809 | 0.182903 | -0.156573 |
| C | 1.254911 | -2.320557 | -0.097743 |
| H | 1.827858 | 1.224478 | -0.208370 |
| H | 0.649352 | -2.644837 | 0.756109 |
| H | 2.260324 | -2.757010 | 0.008443 |
| H | 0.800050 | -2.723458 | -1.009404 |
| Br | -1.106400 | 1.978106 | -0.293032 |
| Br | 4.084513 | 0.011810 | -0.057530 |
| Br | -1.988370 | -0.999485 | -1.438294 |
| Br | -1.578516 | -0.530313 | 1.816046 |

| TS1-Br, MP2 | x | y | z |
|---|---|---|---|
| B | -1.220310 | 0.112367 | 0.018955 |
| C | 1.043179 | -0.658041 | -0.084812 |
| C | 1.854261 | 0.333042 | -0.057595 |
| C | 1.042549 | -2.127570 | -0.123948 |
| H | 1.605651 | 1.385140 | -0.036688 |
| H | 0.355133 | -2.546946 | 0.614052 |
| H | 2.060345 | -2.465422 | 0.112111 |
| H | 0.766137 | -2.492596 | -1.115676 |
| Br | -1.044736 | 2.007147 | -0.260710 |
| Br | 3.750682 | 0.037874 | -0.047291 |
| Br | -1.850416 | -0.951008 | -1.455165 |
| Br | -1.493406 | -0.514773 | 1.818296 |

| TS1-Br, B3LYP | x | y | z |
|---|---|---|---|
| B | -1.270563 | 0.125060 | 0.023277 |
| C | 1.070686 | -0.693936 | -0.104161 |
| C | 1.888746 | 0.320593 | -0.055893 |
| C | 1.048226 | -2.159817 | -0.155333 |
| H | 1.639524 | 1.370697 | -0.012779 |
| H | 0.368937 | -2.578164 | 0.593837 |
| H | 2.061517 | -2.532056 | 0.054651 |
| H | 0.739686 | -2.520649 | -1.141395 |
| Br | -1.051108 | 2.019893 | -0.274970 |
| Br | 3.813993 | 0.036275 | -0.058033 |
| Br | -1.915005 | -0.958459 | -1.439327 |
| Br | -1.490818 | -0.502457 | 1.837520 |

| IM-Br, MP2 | x | y | z |
|---|---|---|---|
| B | -0.872120 | 0.065141 | -0.134232 |
| C | 0.611633 | -0.444163 | -0.355078 |
| C | 1.646754 | 0.454499 | -0.104162 |
| C | 0.897321 | -1.804244 | -0.909791 |
| H | 1.470393 | 1.446960 | 0.294136 |
| H | 0.043918 | -2.470045 | -0.784445 |
| H | 1.785860 | -2.253166 | -0.459151 |
| H | 1.081524 | -1.692909 | -1.987992 |
| Br | -1.197436 | 2.010889 | -0.038421 |
| Br | 3.424133 | 0.079535 | -0.400564 |
| Br | -2.307412 | -0.914437 | -1.054357 |
| Br | -0.460866 | -0.735789 | 1.831136 |
| IM-Br, B3LYP | x | y | z |
| B | -0.828391 | 0.031623 | -0.107918 |
| C | 0.659999 | -0.501720 | -0.361301 |
| C | 1.704055 | 0.350414 | -0.007861 |
| C | 0.907308 | -1.856245 | -0.929272 |
| H | 1.529468 | 1.315325 | 0.451117 |
| H | 0.108491 | -2.548148 | -0.656242 |
| H | 1.878448 | -2.267221 | -0.646766 |
| H | 0.877310 | -1.766637 | -2.026497 |
| Br | -1.068089 | 2.024452 | -0.209054 |
| Br | 3.498155 | -0.008163 | -0.302387 |
| Br | -2.269963 | -0.935807 | -1.091427 |
| Br | -0.728101 | -0.590371 | 1.923113 |
| $C_2H_2I\cdot+BI_3$, MP2 | x | y | z |
| B | -1.602493 | 0.146379 | 0.066855 |
| C | 1.469222 | -0.866699 | -0.278097 |
| C | 2.200828 | 0.167767 | -0.163429 |
| C | 1.422210 | -2.322153 | -0.412336 |
| H | 1.900221 | 1.204787 | -0.085694 |
| H | 0.780281 | -2.761426 | 0.356514 |
| H | 2.436781 | -2.730876 | -0.301700 |
| H | 1.032566 | -2.605168 | -1.393958 |
| I | 4.371247 | -0.012466 | -0.094564 |
| I | -1.186007 | 2.227945 | -0.181986 |
| I | -2.174879 | -1.030678 | -1.622156 |
| I | -1.551700 | -0.726550 | 2.015947 |
| $C_2H_2I\cdot+BI_3$, B3LYP | x | y | z |
| B | -1.567098 | 0.157953 | 0.088038 |
| C | 1.388953 | -1.019784 | -0.458894 |
| C | 2.042937 | 0.076325 | -0.415225 |
| C | 1.277745 | -2.470761 | -0.422607 |
| H | 1.749627 | 1.112058 | -0.487513 |
| H | 0.591463 | -2.794406 | 0.367912 |
| H | 2.266181 | -2.912741 | -0.221344 |
| H | 0.912147 | -2.864093 | -1.377349 |
| I | 4.302816 | 0.010014 | -0.105677 |
| I | -1.175447 | 2.244247 | -0.203859 |
| I | -2.187644 | -1.051007 | -1.567969 |
| I | -1.429191 | -0.690901 | 2.048419 |

| TS1-I, MP2 | x | y | z |
|---|---|---|---|
| B | -1.273465 | 0.095674 | 0.039072 |
| C | 1.145347 | -0.753904 | -0.180518 |
| C | 1.941560 | 0.233784 | -0.048289 |
| C | 1.126723 | -2.207314 | -0.390523 |
| H | 1.694898 | 1.276286 | 0.100741 |
| H | 0.394417 | -2.696705 | 0.256068 |
| H | 2.124049 | -2.598446 | -0.147996 |
| H | 0.894425 | -2.445266 | -1.431432 |
| I | 4.070869 | -0.068964 | -0.109491 |
| I | -1.046684 | 2.234668 | -0.117040 |
| I | -2.006193 | -0.968231 | -1.685497 |
| I | -1.471242 | -0.775769 | 2.001523 |

| TS1-I, B3LYP | x | y | z |
|---|---|---|---|
| B | -1.322093 | 0.108482 | 0.036010 |
| C | 1.162162 | -0.800922 | -0.152054 |
| C | 1.952583 | 0.216389 | -0.020418 |
| C | 1.123178 | -2.254139 | -0.335228 |
| H | 1.709204 | 1.257895 | 0.127889 |
| H | 0.382239 | -2.722571 | 0.320232 |
| H | 2.111030 | -2.668010 | -0.085693 |
| H | 0.882596 | -2.518651 | -1.369821 |
| I | 4.125733 | -0.064789 | -0.102697 |
| I | -1.058466 | 2.243310 | -0.150191 |
| I | -2.010750 | -0.995189 | -1.684178 |
| I | -1.507501 | -0.746710 | 2.010152 |

| IM-I, MP2 | x | y | z |
|---|---|---|---|
| B | -0.837914 | 0.055045 | -0.153615 |
| C | 0.640131 | -0.420741 | -0.500663 |
| C | 1.681045 | 0.386066 | -0.095310 |
| C | 0.882059 | -1.646624 | -1.334081 |
| H | 1.518946 | 1.278851 | 0.498913 |
| H | 0.057667 | -2.355656 | -1.252458 |
| H | 1.814602 | -2.145442 | -1.058524 |
| H | 0.953144 | -1.336806 | -2.385923 |
| I | 3.680920 | 0.083963 | -0.541448 |
| I | -1.181167 | 2.195274 | 0.335711 |
| I | -2.489400 | -0.813006 | -1.337100 |
| I | -0.375904 | -1.195068 | 1.855033 |

| IM-I, B3LYP | x | y | z |
|---|---|---|---|
| B | -0.573655 | 0.264779 | -0.003295 |
| C | 0.933926 | 0.368897 | 0.410796 |
| C | 1.889219 | -0.186387 | -0.346339 |
| C | 1.240096 | 1.178724 | 1.654148 |
| H | 1.706628 | -0.760606 | -1.244588 |
| H | 0.338560 | 1.387376 | 2.235901 |
| H | 1.690890 | 2.141289 | 1.383953 |
| H | 1.949544 | 0.646249 | 2.296828 |
| I | 3.976284 | -0.031173 | 0.036239 |
| I | -1.143681 | -1.347378 | -1.562843 |
| I | -1.751192 | -1.060662 | 1.609749 |
| I | -1.594558 | 2.195710 | -0.365546 |

**Table S30.** Cartesian coordinates (in Å) for structures referring to Figure S19

| IM+BCl$_3$, MP2 | x | y | z |
|---|---|---|---|
| B | -1.296061 | -1.052098 | 0.069485 |
| C | -1.450867 | 0.488947 | -0.399136 |
| C | -1.399714 | 1.506651 | 0.584680 |
| Cl | -2.354189 | -2.141008 | -0.931329 |
| Cl | 0.543711 | -1.513474 | -0.421738 |
| Cl | -1.532759 | -1.314084 | 1.849640 |
| C | -1.731300 | 0.815818 | -1.809756 |
| Cl | -1.473169 | 3.119035 | 0.248341 |
| H | -1.323321 | 1.264421 | 1.638962 |
| H | -1.272467 | 0.088427 | -2.482627 |
| H | -1.445132 | 1.831786 | -2.085225 |
| H | -2.822899 | 0.724404 | -1.946914 |
| B | 1.911410 | -0.008964 | -0.037238 |
| Cl | 1.604667 | 1.247100 | -1.303297 |
| Cl | 1.581052 | 0.577817 | 1.637258 |
| Cl | 3.470590 | -0.885517 | -0.228296 |
| IM+BCl$_3$, B3LYP | x | y | z |
| B | -2.093563 | -0.552084 | 0.095469 |
| C | -1.363909 | 0.773771 | -0.461651 |
| C | -0.731576 | 1.592640 | 0.482592 |
| Cl | -3.340645 | -1.254959 | -1.065556 |
| Cl | -0.497436 | -1.621670 | 0.133926 |
| Cl | -2.789274 | -0.365405 | 1.805204 |
| C | -1.334270 | 1.077033 | -1.910333 |
| Cl | -0.037217 | 3.093248 | 0.118807 |
| H | -0.673094 | 1.320075 | 1.529578 |
| H | -1.232993 | 0.156393 | -2.492262 |
| H | -0.566527 | 1.795999 | -2.197881 |
| H | -2.322945 | 1.484920 | -2.177937 |
| B | 2.654733 | -0.366590 | -0.011346 |
| Cl | 2.277541 | 0.482337 | -1.499533 |
| Cl | 2.456082 | 0.448233 | 1.528997 |
| Cl | 3.258491 | -2.006766 | -0.065713 |
| TS2-Cl, MP2 | x | y | z |
| B | -1.975948 | -0.677455 | 0.101292 |
| C | -1.417610 | 0.728199 | -0.394171 |
| C | -1.017944 | 1.655095 | 0.604012 |
| Cl | -2.928680 | -1.624288 | -1.014613 |
| Cl | 0.354055 | -1.423798 | -0.591342 |
| Cl | -2.065127 | -1.039688 | 1.808173 |
| C | -1.491161 | 1.129073 | -1.813175 |
| Cl | -0.582310 | 3.208208 | 0.277990 |
| H | -1.032883 | 1.396431 | 1.656466 |
| H | -1.405307 | 0.267782 | -2.475817 |
| H | -0.754332 | 1.888158 | -2.079313 |
| H | -2.494520 | 1.563180 | -1.971695 |
| B | 1.884320 | -0.410609 | -0.041384 |
| Cl | 2.075796 | 0.989114 | -1.214251 |
| Cl | 1.580170 | 0.213797 | 1.662737 |
| Cl | 3.313478 | -1.543900 | -0.093940 |

| TS2-Cl, B3LYP | x | y | z |
|---|---|---|---|
| B | -1.627481 | -0.948732 | 0.069800 |
| C | -1.437643 | 0.568014 | -0.388276 |
| C | -1.408575 | 1.562994 | 0.603887 |
| Cl | -2.527655 | -1.998762 | -1.060058 |
| Cl | 0.593357 | -1.569284 | -0.448780 |
| Cl | -1.770757 | -1.326317 | 1.806903 |
| C | -1.381234 | 0.921085 | -1.824766 |
| Cl | -1.325520 | 3.213299 | 0.259993 |
| H | -1.553812 | 1.332156 | 1.651837 |
| H | -1.064957 | 0.078226 | -2.439289 |
| H | -0.752137 | 1.793060 | -2.018046 |
| H | -2.405660 | 1.187891 | -2.140850 |
| B | 1.903154 | -0.179119 | -0.050776 |
| Cl | 2.095497 | 0.881003 | -1.537099 |
| Cl | 1.232061 | 0.818421 | 1.393100 |
| Cl | 3.453777 | -1.022162 | 0.439240 |

| E+$BCl_4^{\cdot}$, MP2 | x | y | z |
|---|---|---|---|
| B | -2.283184 | -0.468966 | 0.100308 |
| C | -1.331222 | 0.692158 | -0.410494 |
| C | -0.826931 | 1.538933 | 0.593319 |
| Cl | -3.282652 | -1.305894 | -1.044933 |
| Cl | 0.255220 | -1.144715 | -0.660325 |
| Cl | -2.412193 | -0.854940 | 1.791111 |
| C | -1.396300 | 1.174988 | -1.822451 |
| Cl | -0.223897 | 3.056223 | 0.296787 |
| H | -0.834900 | 1.258497 | 1.639552 |
| H | -1.595879 | 0.357156 | -2.514119 |
| H | -0.477889 | 1.685597 | -2.120483 |
| H | -2.225991 | 1.893455 | -1.898840 |
| B | 1.982550 | -0.488331 | -0.012267 |
| Cl | 2.429390 | 0.933904 | -1.062333 |
| Cl | 1.769551 | -0.010416 | 1.737729 |
| Cl | 3.109554 | -1.900321 | -0.217363 |

| E+$BCl_4^{\cdot}$, B3LYP | x | y | z |
|---|---|---|---|
| B | -2.069073 | -0.691091 | 0.091661 |
| C | -1.463825 | 0.700011 | -0.361649 |
| C | -1.218012 | 1.658095 | 0.634329 |
| Cl | -2.951378 | -1.649060 | -1.081258 |
| Cl | 0.650545 | -1.655901 | -0.683057 |
| Cl | -2.066194 | -1.180927 | 1.774412 |
| C | -1.264739 | 1.033952 | -1.793198 |
| Cl | -0.807929 | 3.262303 | 0.281459 |
| H | -1.441879 | 1.476273 | 1.678126 |
| H | -1.325577 | 0.155271 | -2.432526 |
| H | -0.305759 | 1.540061 | -1.950962 |
| H | -2.046074 | 1.746788 | -2.105372 |
| B | 1.922106 | -0.416733 | -0.091545 |
| Cl | 2.331750 | 0.791676 | -1.427043 |
| Cl | 1.158142 | 0.538689 | 1.395432 |
| Cl | 3.422334 | -1.267463 | 0.559659 |

| IM+BBr$_3$, MP2 | x | y | z |
|---|---|---|---|
| C | -0.795270 | -0.183923 | -1.738541 |
| C | 0.021274 | 0.942542 | -1.663034 |
| C | -0.571812 | 2.314864 | -1.619517 |
| H | -0.881575 | 2.580255 | -2.640843 |
| H | 0.160674 | 3.051809 | -1.291005 |
| H | -1.454214 | 2.357723 | -0.975796 |
| B | 1.587588 | 0.720429 | -1.765677 |
| Br | 1.403177 | 0.687198 | 0.373718 |
| Br | 2.247588 | -1.020793 | -2.432920 |
| Br | 2.658095 | 2.244655 | -2.402544 |
| Br | -2.623270 | -0.102499 | -1.893433 |
| H | -0.398441 | -1.192554 | -1.740016 |
| B | -1.164341 | -0.761907 | 2.364887 |
| Br | 0.058111 | -0.688852 | 3.813916 |
| Br | -2.307107 | 0.714101 | 2.011708 |
| Br | -1.270755 | -2.327686 | 1.292243 |
| IM+BBr$_3$, B3LYP | x | y | z |
| B | -1.097362 | -1.137317 | -0.089432 |
| C | -1.723269 | 0.287844 | -0.493083 |
| C | -2.408997 | 0.996865 | 0.497494 |
| C | -1.777816 | 0.727615 | -1.919981 |
| H | -2.405602 | 0.682659 | 1.533924 |
| H | -1.048048 | 0.202148 | -2.535181 |
| H | -1.628699 | 1.806658 | -2.013668 |
| H | -2.779074 | 0.505427 | -2.315036 |
| Br | -0.911437 | -1.529730 | 1.849612 |
| Br | -3.415672 | 2.512201 | 0.181561 |
| Br | -2.184295 | -2.522823 | -1.069092 |
| Br | 0.866361 | -1.243121 | -0.985748 |
| Br | 2.786784 | -0.325322 | 1.626783 |
| B | 1.997711 | 0.368092 | -0.040828 |
| Br | 3.317867 | 0.923665 | -1.411929 |
| Br | 0.649540 | 1.858710 | 0.308090 |
| TS2-Br, MP2 | x | y | z |
| C | -2.385501 | -2.683501 | -0.118041 |
| C | -1.479067 | -1.638569 | 0.004557 |
| C | -1.946836 | -0.213645 | -0.047474 |
| H | -2.192613 | 0.032894 | -1.089581 |
| H | -1.169532 | 0.471044 | 0.290174 |
| H | -2.843965 | -0.067911 | 0.559336 |
| B | 0.043229 | -2.013709 | -0.209492 |
| Br | -0.529024 | -1.774584 | 2.345232 |
| Br | 0.609044 | -3.829661 | -0.475896 |
| Br | 1.298328 | -0.625783 | -0.611769 |
| Br | -4.192703 | -2.451625 | -0.289614 |
| H | -2.076612 | -3.722150 | -0.129894 |
| B | -2.138782 | -3.148732 | 3.660071 |
| Br | -1.153743 | -3.152088 | 5.332910 |
| Br | -3.735712 | -2.047420 | 3.585997 |
| Br | -2.191202 | -4.825388 | 2.676494 |

| TS2-Br, B3LYP | x | y | z |
|---|---|---|---|
| B | -1.559599 | -1.111272 | -0.012458 |
| C | -1.830660 | 0.382208 | -0.483257 |
| C | -2.079852 | 1.326650 | 0.504858 |
| C | -2.002125 | 0.703852 | -1.930229 |
| H | -1.969094 | 1.104984 | 1.558897 |
| H | -1.449561 | 0.007727 | -2.562150 |
| H | -1.702016 | 1.729587 | -2.157552 |
| H | -3.068304 | 0.607253 | -2.185856 |
| Br | -1.214438 | -1.509717 | 1.848110 |
| Br | -2.790731 | 3.011623 | 0.180164 |
| Br | -2.446192 | -2.484646 | -1.068060 |
| Br | 0.846316 | -1.291751 | -1.092958 |
| Br | 2.444129 | -0.545615 | 1.738573 |
| B | 1.963960 | 0.105322 | -0.074750 |
| Br | 3.553794 | 0.625160 | -1.172846 |
| Br | 0.796923 | 1.826487 | 0.059431 |

| E+$BBr_4^{\cdot}$, MP2 | x | y | z |
|---|---|---|---|
| C | -0.890262 | -0.158091 | -1.788631 |
| C | 0.002579 | 0.893600 | -1.616293 |
| C | -0.498996 | 2.311141 | -1.573736 |
| H | -0.774086 | 2.625478 | -2.589887 |
| H | 0.269180 | 2.992162 | -1.206699 |
| H | -1.382801 | 2.397050 | -0.936537 |
| B | 1.488572 | 0.569445 | -2.038733 |
| Br | 0.908632 | 0.686049 | 0.777872 |
| Br | 2.101342 | -1.214966 | -2.331333 |
| Br | 2.689102 | 2.005739 | -2.378877 |
| Br | -2.697185 | 0.052605 | -1.928521 |
| H | -0.566782 | -1.188370 | -1.881102 |
| B | -0.633855 | -0.386161 | 1.869968 |
| Br | 0.181230 | -0.506130 | 3.670297 |
| Br | -2.256656 | 0.741692 | 1.809784 |
| Br | -0.862007 | -2.145568 | 0.989153 |

| E+$BBr_4^{\cdot}$, B3LYP | x | y | z |
|---|---|---|---|
| B | -1.878191 | -1.112130 | 0.068270 |
| C | -2.031363 | 0.370482 | -0.434399 |
| C | -2.332330 | 1.311232 | 0.526304 |
| C | -2.051517 | 0.666843 | -1.900809 |
| H | -2.319051 | 1.087598 | 1.586069 |
| H | -1.271025 | 0.098486 | -2.414168 |
| H | -1.922292 | 1.730579 | -2.107244 |
| H | -3.013835 | 0.346059 | -2.323267 |
| Br | -1.286364 | -1.528698 | 1.832798 |
| Br | -2.899433 | 3.049370 | 0.166139 |
| Br | -2.460844 | -2.524941 | -1.082497 |
| Br | 1.263822 | -1.303129 | -1.357983 |
| Br | 2.462972 | -0.722821 | 1.686448 |
| B | 2.037765 | 0.043117 | -0.097597 |
| Br | 3.589988 | 1.060803 | -0.889700 |
| Br | 0.650419 | 1.626302 | 0.109329 |

**Table S31.** Cartesian coordinates (in Å) for structures referring to Figure S20

| IM+BI$_3$ | | x | y | z |
|---|---|---|---|---|
| | B | -1.816368 | 0.341814 | 0.252650 |
| | C | -0.904971 | 1.479214 | -0.335069 |
| | C | 0.028537 | 2.012877 | 0.468861 |
| | C | -1.135323 | 1.988612 | -1.740127 |
| | H | 0.212153 | 1.692057 | 1.484616 |
| | H | -1.662447 | 1.261291 | -2.360748 |
| | H | -0.185965 | 2.228454 | -2.227605 |
| | H | -1.737974 | 2.905052 | -1.715945 |
| | I | 1.325009 | 3.610238 | -0.060254 |
| | I | -1.527629 | -0.378315 | 2.325177 |
| | I | -4.087517 | 0.783975 | -0.195934 |
| | I | -1.976249 | -1.505435 | -1.227686 |
| | B | 2.180773 | -1.057832 | -0.195584 |
| | I | 3.128224 | -0.075016 | 1.443170 |
| | I | 1.251172 | -2.967450 | 0.117837 |
| | I | 2.144022 | -0.173487 | -2.134907 |
| TS2 | | x | y | z |
| | B | -2.170641 | 0.050493 | 0.072303 |
| | C | -1.433062 | 1.242188 | -0.600854 |
| | C | -0.938886 | 2.182304 | 0.227574 |
| | C | -1.368919 | 1.345531 | -2.105722 |
| | H | -1.000997 | 2.119157 | 1.305090 |
| | H | -1.141105 | 0.375775 | -2.556710 |
| | H | -0.606826 | 2.059815 | -2.424705 |
| | H | -2.335174 | 1.675677 | -2.506284 |
| | I | 0.017113 | 3.982590 | -0.343707 |
| | I | -2.383323 | -0.190900 | 2.226335 |
| | I | -3.786875 | -0.948214 | -1.129695 |
| | I | -0.704201 | -2.156585 | -0.767962 |
| | B | 2.446017 | -0.421407 | 0.130486 |
| | I | 1.912911 | 0.509417 | 1.970438 |
| | I | 2.957847 | -2.513566 | 0.108565 |
| | I | 2.479970 | 0.694692 | -1.685810 |
| E+BI$_3$+I· | | x | y | z |
| | B | -2.424907 | 0.038912 | 0.000143 |
| | C | -1.664371 | 1.194603 | -0.681996 |
| | C | -1.276196 | 2.203739 | 0.128959 |
| | C | -1.451035 | 1.194713 | -2.176422 |
| | H | -1.438672 | 2.195755 | 1.198388 |
| | H | -0.949975 | 0.274028 | -2.491341 |
| | H | -0.841809 | 2.043213 | -2.494321 |
| | H | -2.410458 | 1.241924 | -2.703881 |
| | I | -0.332563 | 3.999070 | -0.450418 |
| | I | -2.659118 | -0.208074 | 2.127051 |
| | I | -3.501175 | -1.413714 | -1.248727 |
| | I | -0.239740 | -2.423572 | -0.734893 |
| | B | 2.424221 | -0.181312 | 0.232628 |
| | I | 1.573268 | 0.653851 | 2.000129 |
| | I | 3.310455 | -2.145319 | 0.307982 |
| | I | 2.452532 | 0.922640 | -1.591614 |

| TS3 | | x | y | z |
|---|---|---|---|---|
| | B | -2.998388 | 0.163114 | 0.083250 |
| | C | -2.301976 | 1.446132 | -0.435457 |
| | C | -1.279786 | 1.916742 | 0.314609 |
| | C | -2.722086 | 2.064406 | -1.747064 |
| | H | -0.978488 | 1.461391 | 1.248664 |
| | H | -2.465839 | 1.406125 | -2.585227 |
| | H | -2.235249 | 3.028869 | -1.908769 |
| | H | -3.806463 | 2.212000 | -1.776995 |
| | I | -0.010147 | 3.529920 | -0.157230 |
| | I | -2.745250 | -0.607116 | 2.086025 |
| | I | -4.302621 | -0.953509 | -1.228022 |
| | I | 0.068692 | -1.086902 | -1.405499 |
| | B | 2.431573 | -0.484230 | 0.217685 |
| | I | 1.627803 | -0.037148 | 2.159801 |
| | I | 2.816981 | -2.600788 | -0.287209 |
| | I | 3.490641 | 1.018439 | -0.890033 |

| $E+BI_4^{\cdot}$ | | x | y | z |
|---|---|---|---|---|
| | B | -2.793534 | 0.077509 | -0.087566 |
| | C | -1.864994 | 1.203095 | -0.640017 |
| | C | -1.234336 | 1.963140 | 0.277644 |
| | C | -1.721186 | 1.390004 | -2.128168 |
| | H | -1.326486 | 1.832702 | 1.346906 |
| | H | -1.369431 | 0.464196 | -2.594573 |
| | H | -1.018556 | 2.188285 | -2.374502 |
| | H | -2.690927 | 1.630556 | -2.577173 |
| | I | 0.117303 | 3.532411 | -0.153824 |
| | I | -2.993710 | -0.373659 | 2.012036 |
| | I | -4.041646 | -1.046632 | -1.443972 |
| | I | 0.097990 | -1.862233 | -0.785342 |
| | B | 1.800663 | -0.725245 | 0.096321 |
| | I | 1.420436 | -0.031893 | 2.168170 |
| | I | 3.753836 | -1.865012 | -0.142150 |
| | I | 2.406033 | 1.076932 | -1.256827 |

## References


(1) Hanwell, M. D.; Curtis, D. E.; Lonie, D. C.; Vandermeersch, T.; Zurek, E.; Hutchison, G. R. Avogadro: An Advanced Semantic Chemical Editor, Visualization, and Analysis Platform. *J. Cheminformatics* **2012**, *4* (1), 17. https://doi.org/10.1186/1758-2946-4-17.

(2) Møller, Chr.; Plesset, M. S. Note on an Approximation Treatment for Many-Electron Systems. *Phys. Rev.* **1934**, *46* (7), 618–622. https://doi.org/10.1103/PhysRev.46.618.

(3) Frisch, M. J.; Head-Gordon, M.; Pople, J. A. A Direct MP2 Gradient Method. *Chem. Phys. Lett.* **1990**, *166* (3), 275–280. https://doi.org/10.1016/0009-2614(90)80029-D.

(4) Frisch, M. J.; Head-Gordon, M.; Pople, J. A. Semi-Direct Algorithms for the MP2 Energy and Gradient. *Chem. Phys. Lett.* **1990**, *166* (3), 281–289. https://doi.org/10.1016/0009-2614(90)80030-H.

(5) Head-Gordon, M.; Pople, J. A.; Frisch, M. J. MP2 Energy Evaluation by Direct Methods. *Chem. Phys. Lett.* **1988**, *153* (6), 503–506. https://doi.org/10.1016/0009-2614(88)85250-3.

(6) Sæbø, S.; Almlöf, J. Avoiding the Integral Storage Bottleneck in LCAO Calculations of Electron Correlation. *Chem. Phys. Lett.* **1989**, *154* (1), 83–89. https://doi.org/10.1016/0009-2614(89)87442-1.

(7) Head-Gordon, M.; Head-Gordon, T. Analytic MP2 Frequencies without Fifth-Order Storage. Theory and Application to Bifurcated Hydrogen Bonds in the Water Hexamer. *Chem. Phys. Lett.* **1994**, *220* (1), 122–128. https://doi.org/10.1016/0009-2614(94)00116-2.

(8) Frisch, M. J.; Trucks, G. W.; Schlegel, H. B.; Scuseria, G. E.; Robb, M. A.; Cheeseman, J. R.; Scalmani, G.; Barone, V.; Mennucci, B.; Petersson, G. A.; Nakatsuji, H.; Caricato, M.; Li, X.; Hratchian, H. P.; Izmaylov, A. F.; Bloino, J.; Zheng, G.; Sonnenberg, J. L.; Hada, M.; Ehara, M.; Toyota, K.; Fukuda, R.; Hasegawa, J.; Ishida, M.; Nakajima, T.; Honda, Y.; Kitao, O.; Nakai, H.; Vreven, T.; Montgomery, J. A. Jr.; Peralta, J. E.; Ogliaro, F.; Beapark, M.; Heyd, J. J.; Brothers, E.; Kudin, K. N.; Staroverov, V. N.; Kobayashi, R.; Normand, J.; Raghavachari, K.; Rendell, A.; Burant, J. C.; Iyengar, S. S.; Tomasi, J.; Cossi, M.; Rega, N.; Millam, J. M.; Kleine, M.; Knox, J. E.; Cross, J. B.; Bakken, V.; Adamo, C.; Jaramillo, J.; Gomperts, R.; Stratmann, R. E.; Yazyev, O.; Austin, A. J.; Cammi, R.; Pomelli, C.; Ochterski, J. W.; Martin, R. L.; Morokuma, K.; Zakrzewski, V. G.; Voth, G. A.; Salvador, P.; Dannenberg, J. J.; Dapprich, S.; Daniels, A. D.; Farkas, Ö.; Foresman, J. B.; Ortiz, J. V.; Cioslowski, J.; Fox, D. J. Gaussian 09, Revision D.01, 2009.

(9) Frisch, M. J.; Trucks, G. W.; Schlegel, H. B.; Scuseria, G. E.; Robb, M. A.; Cheeseman, J. R.; Scalmani, G.; Barone, V.; Petersson, G. A.; Nakatsuji, H.; Li, X.; Caricato, M.; Marenich, A. V.; Bloino, J.; Janesco, B. G.; Gomperts, R.; Mennucci, B.; Hratchian, H. P.; Ortiz, J. V.; Izmaylov, A. F.; Sonnenberg, J. L.; Williams-Young, D.; Ding, F.; Lipparini, F.; Egidi, F.; Goings, J.; Peng, B.; Petrone, A.; Henderson, T.; Ranasinghe, D.; Zakrzewski, V. G.; Gao, J.; Rega, N.; Zheng, G.; Liang, W.; Hada, M.; Ehara, M.; Toyota, K.; Fukuda, R.; Hasegawa, J.; Ishida, M.; Nakajima, T.; Honda, Y.; Kitao, O.; Nakai, H.; Vreven, T.; Throssell, K.; Montgomery, J. A. Jr.; Peralta, J. E.; Ogliaro, F.; Beapark, M. J.; Heyd, J. J.; Brothers, E. N.; Kudin, K. N.; Raghavachari, K.; Rendell, A. P.; Burant, J. C.; Iyengar, S. S.; Tomasi, J.; Cossi, M.; Millam, J. M.; Klene, M.; Adamo, C.; Cammi, R.; Ochterski, J. W.; Martin, R. L.; Morokuma, K.; Farkas, Ö.; Foresman, J. B.; Fox, D. J. Gaussian 16, Revision C.01, 2019.

(10) Riplinger, C.; Neese, F. An Efficient and near Linear Scaling Pair Natural Orbital Based Local Coupled Cluster Method. *J. Chem. Phys.* **2013**, *138* (3), 034106. https://doi.org/10.1063/1.4773581.

(11) Riplinger, C.; Sandhoefer, B.; Hansen, A.; Neese, F. Natural Triple Excitations in Local Coupled Cluster Calculations with Pair Natural Orbitals. *J. Chem. Phys.* **2013**, *139* (13), 134101. https://doi.org/10.1063/1.4821834.

(12) Riplinger, C.; Pinski, P.; Becker, U.; Valeev, E. F.; Neese, F. Sparse Maps—A Systematic Infrastructure for Reduced-Scaling Electronic Structure Methods. II. Linear Scaling Domain Based

Pair Natural Orbital Coupled Cluster Theory. *J. Chem. Phys.* **2016**, *144* (2), 024109. https://doi.org/10.1063/1.4939030.
(13) Saitow, M.; Becker, U.; Riplinger, C.; Valeev, E. F.; Neese, F. A New Near-Linear Scaling, Efficient and Accurate, Open-Shell Domain-Based Local Pair Natural Orbital Coupled Cluster Singles and Doubles Theory. *J. Chem. Phys.* **2017**, *146* (16), 164105. https://doi.org/10.1063/1.4981521.
(14) Neese, F. Software Update: The ORCA Program System—Version 5.0. *WIREs Comput. Mol. Sci.* **2022**, *12* (5), e1606. https://doi.org/10.1002/wcms.1606.
(15) Becke, A. D. Density-functional Thermochemistry. III. The Role of Exact Exchange. *J. Chem. Phys.* **1993**, *98* (7), 5648–5652. https://doi.org/10.1063/1.464913.
(16) Lee, C.; Yang, W.; Parr, R. G. Development of the Colle-Salvetti Correlation-Energy Formula into a Functional of the Electron Density. *Phys. Rev. B* **1988**, *37* (2), 785–789. https://doi.org/10.1103/PhysRevB.37.785.
(17) Miehlich, B.; Savin, A.; Stoll, H.; Preuss, H. Results Obtained with the Correlation Energy Density Functionals of Becke and Lee, Yang and Parr. *Chem. Phys. Lett.* **1989**, *157* (3), 200–206. https://doi.org/10.1016/0009-2614(89)87234-3.
(18) Grimme, S.; Ehrlich, S.; Goerigk, L. Effect of the Damping Function in Dispersion Corrected Density Functional Theory. *J. Comput. Chem.* **2011**, *32* (7), 1456–1465. https://doi.org/10.1002/jcc.21759.
(19) Schäfer, A.; Horn, H.; Alrichs, R. Fully Optimized Contracted Gaussian Basis Sets for Atoms Li to Kr. *J. Chem. Phys.* **1992**, *97* (4), 2571–2577. https://doi.org/10.1063/1.463096.
(20) Ahlrichs, R.; May, K. Contracted All-Electron Gaussian Basis Sets for Atoms Rb to Xe. *Phys. Chem. Chem. Phys.* **2000**, *2* (5), 943–945. https://doi.org/10.1039/A908859H.
(21) Clark, T.; Chandrasekhar, J.; Spitznagel, G. W.; Schleyer, P. V. R. Efficient Diffuse Function-Augmented Basis Sets for Anion Calculations. III. The 3-21+G Basis Set for First-Row Elements, Li–F. *J. Comput. Chem.* **1983**, *4* (3), 294–301. https://doi.org/10.1002/jcc.540040303.
(22) Dill, J. D.; Pople, J. A. Self-consistent Molecular Orbital Methods. XV. Extended Gaussian-type Basis Sets for Lithium, Beryllium, and Boron. *J. Chem. Phys.* **1975**, *62* (7), 2921–2923. https://doi.org/10.1063/1.430801.
(23) Ditchfield, R.; Hehre, W. J.; Pople, J. A. Self-Consistent Molecular-Orbital Methods. IX. An Extended Gaussian-Type Basis for Molecular-Orbital Studies of Organic Molecules. *J. Chem. Phys.* **1971**, *54* (2), 724–728. https://doi.org/10.1063/1.1674902.
(24) Francl, M. M.; Pietro, W. J.; Hehre, W. J.; Binkley, J. S.; Gordon, M. S.; DeFrees, D. J.; Pople, J. A. Self-consistent Molecular Orbital Methods. XXIII. A Polarization-type Basis Set for Second-row Elements. *J. Chem. Phys.* **1982**, *77* (7), 3654–3665. https://doi.org/10.1063/1.444267.
(25) Gordon, M. S.; Binkley, J. S.; Pople, J. A.; Pietro, W. J.; Hehre, W. J. Self-Consistent Molecular-Orbital Methods. 22. Small Split-Valence Basis Sets for Second-Row Elements. *J. Am. Chem. Soc.* **1982**, *104* (10), 2797–2803. https://doi.org/10.1021/ja00374a017.
(26) Hariharan, P. C.; Pople, J. A. The Influence of Polarization Functions on Molecular Orbital Hydrogenation Energies. *Theor. Chim. Acta* **1973**, *28* (3), 213–222. https://doi.org/10.1007/BF00533485.
(27) Hehre, W. J.; Ditchfield, R.; Pople, J. A. Self-Consistent Molecular Orbital Methods. XII. Further Extensions of Gaussian—Type Basis Sets for Use in Molecular Orbital Studies of Organic Molecules. *J. Chem. Phys.* **1972**, *56* (5), 2257–2261. https://doi.org/10.1063/1.1677527.
(28) Spitznagel, G. W.; Clark, T.; Schleyer, P. von R.; Hehre, W. J. An Evaluation of the Performance of Diffuse Function-Augmented Basis Sets for Second Row Elements, Na-Cl. *J. Comput. Chem.* **1987**, *8* (8), 1109–1116. https://doi.org/10.1002/jcc.540080807.
(29) Weigend, F.; Ahlrichs, R. Balanced Basis Sets of Split Valence, Triple Zeta Valence and Quadruple Zeta Valence Quality for H to Rn: Design and Assessment of Accuracy. *Phys. Chem. Chem. Phys.* **2005**, *7* (18), 3297–3305. https://doi.org/10.1039/B508541A.

(30) Weigend, F. Accurate Coulomb-Fitting Basis Sets for H to Rn. *Phys. Chem. Chem. Phys.* **2006**, *8* (9), 1057–1065. https://doi.org/10.1039/B515623H.

(31) Bergner, A.; Dolg, M.; Küchle, W.; Stoll, H.; Preuß, H. Ab Initio Energy-Adjusted Pseudopotentials for Elements of Groups 13–17. *Mol. Phys.* **1993**, *80* (6), 1431–1441. https://doi.org/10.1080/00268979300103121.

(32) Dunning, T. H. Gaussian Basis Sets for Use in Correlated Molecular Calculations. I. The Atoms Boron through Neon and Hydrogen. *J. Chem. Phys.* **1989**, *90* (2), 1007–1023. https://doi.org/10.1063/1.456153.

(33) Woon, D. E.; Dunning, T. H. Gaussian Basis Sets for Use in Correlated Molecular Calculations. III. The Atoms Aluminum through Argon. *J. Chem. Phys.* **1993**, *98* (2), 1358–1371. https://doi.org/10.1063/1.464303.

(34) Wilson, A. K.; Woon, D. E.; Peterson, K. A.; Dunning, T. H. Gaussian Basis Sets for Use in Correlated Molecular Calculations. IX. The Atoms Gallium through Krypton. *J. Chem. Phys.* **1999**, *110* (16), 7667–7676. https://doi.org/10.1063/1.478678.

(35) Peterson, K. A.; Shepler, B. C.; Figgen, D.; Stoll, H. On the Spectroscopic and Thermochemical Properties of ClO, BrO, IO, and Their Anions. *J. Phys. Chem. A* **2006**, *110* (51), 13877–13883. https://doi.org/10.1021/jp065887l.

(36) *MetaCentrum (MetaVO) - Virtual Organization*. https://metavo.metacentrum.cz/cs/ (accessed 2022-02-10).
